# Supplementary material for: H-bonded reusable template assisted para-selective ketonisation using soft electrophilic vinyl ethers
Source: Nat Commun. 2018 Sep 4;9:3582. doi: 10.1038/s41467-018-06018-2 (PMC6123475; doi:10.1038/s41467-018-06018-2)
Supplement: Supplementary file 1 — Supplementary Data 1 [file 41467_2018_6018_MOESM1_ESM.pdf]

Supplementary Information

H-Bonded Reusable Template-Assisted *Para*-Selective Ketonisation Using Soft Electrophilic Vinyl Ethers

Maji et al.

General considerations:

Reagent Information: Unless otherwise stated, all reactions were carried out under atmospheric condition in screw cap reaction tubes. All solvents were bought from Merck/Aldrich/ TCI in sure-seal bottle and were used as received. For column chromatography, silica gel (100–200 mesh) obtained from SRL Co. and neutral activated alumina from Spectrochem was used. A gradient elution using pet ether and ethyl acetate was performed, based on Merck aluminum TLC sheets (silica gel $60F_{254}$). Benzyl halides were obtained from Aldrich/ TCI/Alfa Aeser and Spectrochem. HFIP was purchased from TCI.

Analytical Information: All isolated compounds were characterized by ^{1}H, ^{13}C NMR spectroscopy, mass analysis and IR spectroscopy. Copies of the ^{1}H NMR, ^{13}C NMR and few NOE spectra can be found in the supporting information. Unless otherwise stated, all Nuclear Magnetic Resonance spectra were recorded on a Bruker 400 MHz instrument. Some Nuclear Magnetic Resonance was taken on a Bruker 500 MHz instrument. All ^{1}H NMR experiments are reported in units, parts per million (ppm), and were measured relative to the signals for residual chloroform (7.26 ppm) in the deuterated solvent, unless otherwise stated. All ^{13}C NMR spectra were reported in ppm relative to deuterochloroform (77.23 ppm), unless otherwise stated, and all were obtained with ^{1}H decoupling. The NMR yields were calculated using 1,3,5-trimethoxybenzene as the reference. High-resolution mass spectra (HRMS) were recorded on a micro-mass ESI TOF (time of flight) mass spectrometer. Neat infrared spectra were recorded on a Perkin-Elmer spectrum one FT-IR spectrometer. The data was recorded in transmittance mode (%T, cm^{-1}).

Description of Reaction Tube:

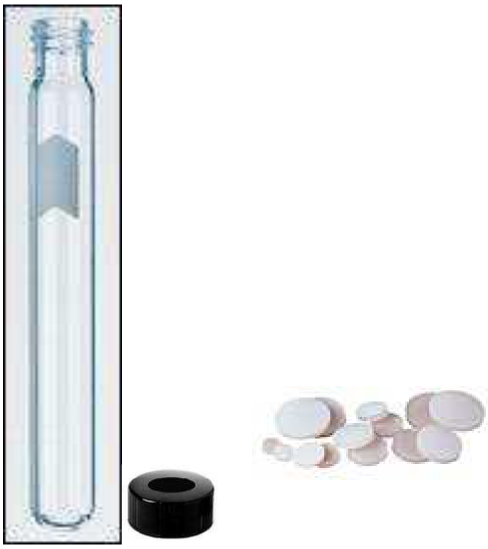

Supplementary Figure 1. Pictorial description of reaction tube for ketonisation: Fisher brand Disposable Borosilicate Glass Tubes (16*125mm) with Threaded End (Fisher Scientific Order No.1495935A) [left]; Kimble Black Phenolic Screw Thread Closures with Open Tops (Fisher Scientific Order No. 033407E) [middle]; Thermo Scientific National PTFE/Silicone Septa for Sample Screw Thread Caps (Fisher Scientific Order No. 03394A) [right].

Optimizations:

Supplementary Table 1: Acylating Agent Optimization:

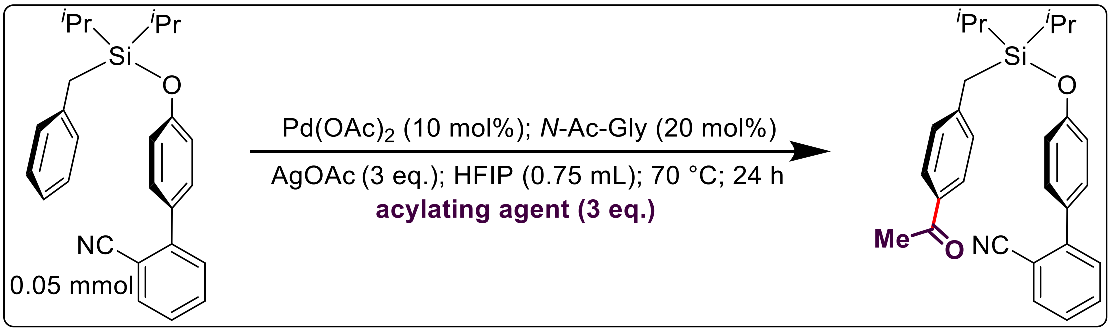

Entry	Acylating Agent	Yield %	Selectivity (*para*: others)
1	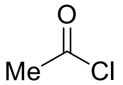	multiple products	-
**2**	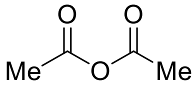	multiple products	**-**
3	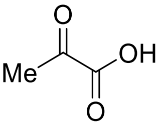	n.d	-
4	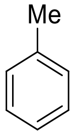	n.d	-
5	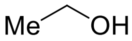 + $K_2S_2O_8$	n.d	-
6	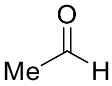	n.d	-
7	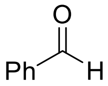	multiple products	
**8**	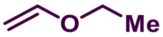	**11**	**2:1**

Supplementary Table 2: Directing Group Optimization:

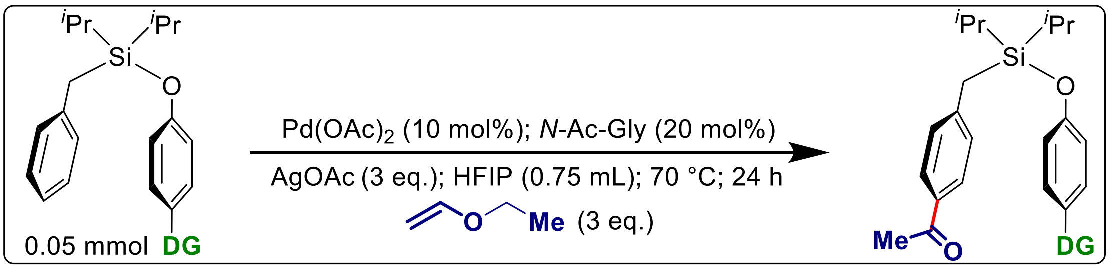

**Directing group**	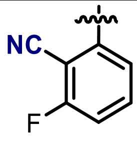	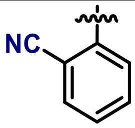	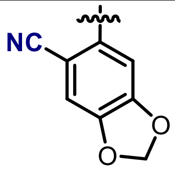	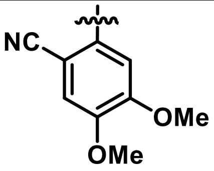
**Yield (*para*:others)**	**DG1**: 21% (11:1)	**DG2**:11% (2:1)	**DG3**: 31% (6:1)	**DG4: 52% (11:1)**
	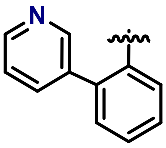	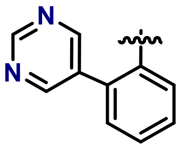	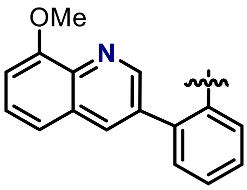	
**Yield (*para*:others)**	**DG5**: 22% (3:1)	**DG6**: 29% (5:1)	**DG7**: 53% (3:1)	

Supplementary Table 3: Palladium Catalyst Optimization:

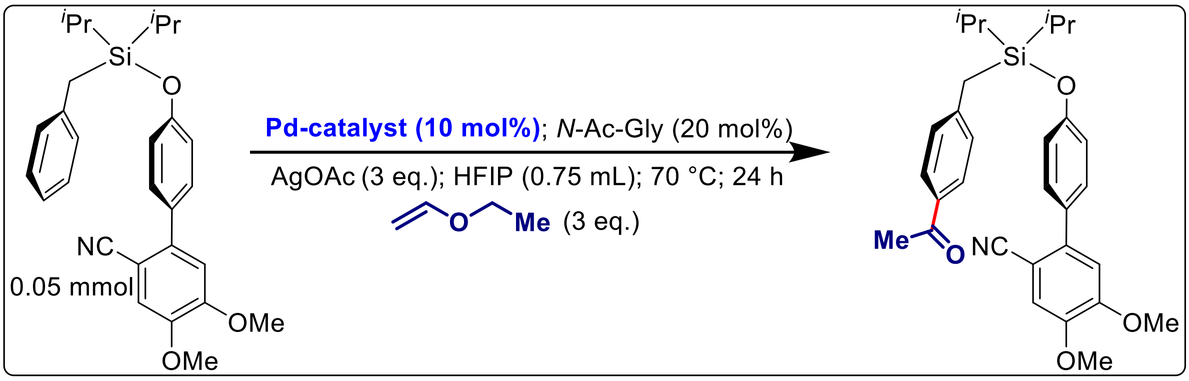

Entry	Pd-salt	Yield %	Selectivity (*para*: others)
**1**	**$Pd(OAc)_2$**	**52**	**11:1**
2	$Pd(Piv)_2$	42	11:1
3	$Pd(TFA)_2$	39	11:1

4	$PdCl_2$	n.d	n.d
5	PdO	n.d	n.d
6	$Pd(acac)_2$	n.d	n.d
7	$Pd_2(dba)_3$	n.d	n.d
8	$Pd(dppf)_2Cl_2$	n.d	n.d
9	$Pd(MeCN)_2Cl_2$	n.d	n.d
10	$Pd(PhCN)_2Cl_2$	n.d	n.d
11	$Pd(PPh_3)_2$	n.d	n.d
12	$Pd(OAc)_2$ + AcOH (2 eq.)	n.d	n.d
13	$Pd(OAc)_2$ + TfOH (2 eq.)	n.d	n.d
14	No catalyst	n.d	n.d

Supplementary Table 4: Ligand Optimization:

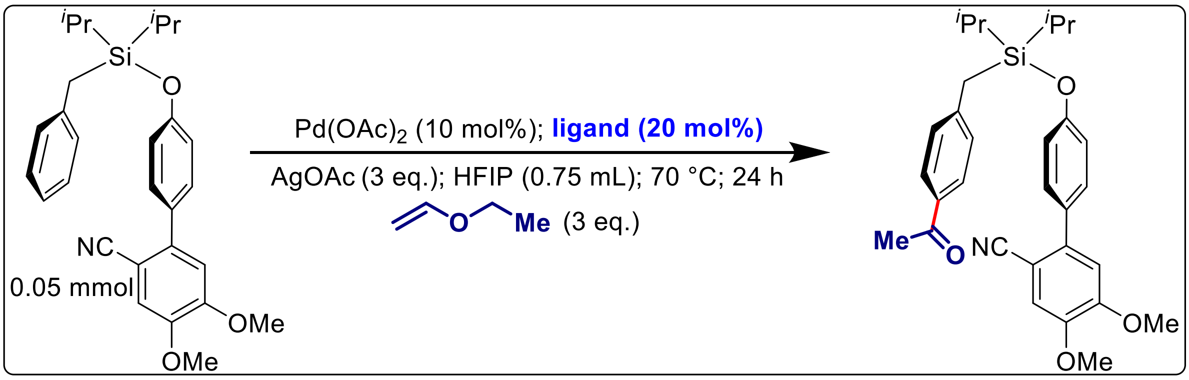

Entry	Ligand	Yield %	Selectivity (*para*: others)
**1**	***N*-Acetylglycine**	**52**	**11:1**
2	*N*-Benzyloxyglycine	n.d	n.d
3	*N*-Boc-glycine	n.d	n.d
4	*N*-Fmoc-glycine	n.d	n.d
**5**	***N*-Cbz-glycine**	**48**	**15:1**
6	*N*-Formylglycine	45	10:1
7	Glycyl glycine	n.d	n.d
8	*N*-Acetylglycine ethyl ester	n.d	n.d

9	*N*-Boc-phenylglycine	n.d	n.d
10	*N*-Acetylalanine	40	12:1
11	*N*-Boc-α-methylalanine	n.d	n.d
12	*N*-Boc-phenylalanine	n.d	n.d
13	*N*-Piv-valine	n.d	n.d
14	*N*-Benzoyl-valine	n.d	n.d
15	*N*-Cbz-valine	n.d	12:1
16	*N*-Acetylleucine	21	11:1
17	*N*-Boc-leucine	n.d	n.d
18	*N*-Cbz-leucine	19	13:1
19	*N*-Piv-leucine	17	10:1
20	*N*-Fmoc-leucine	n.d	n.d
21	*N*-Boc-tert-leucine	n.d	n.d
22	*N*-Boc-isoleucine	n.d	n.d
23	*N*-Benzoyl-isoleucine	n.d	n.d
24	*N*-Boc-aspartic acid	n.d	n.d
25	Proline	n.d	n.d
26	*N*-Boc-aminocyclopentane carboxylic acid	n.d	n.d
27	*N*-Boc-*β*-alanine	n.d	n.d
28	*N*-Benzoyl-*β*-alanine	n.d	n.d
29	Li-Yu’s tert-butyl quinoline	n.d	n.d
30	Quinoline	n.d	n.d
31	8-OMe Quinoline	n.d	n.d
32	5-OMe-2-CN-Quinoline	n.d	n.d
33	Boc-L-Valine	n.d	n.d
34	CBZ-alanine	15	11:1
35	CBZ-phenylalanine	n.d	
36	1,10-phen	n.d	

37	2,2'-bipyridine	n.d
38	4-Methoxyquinoline-2-carboxylic acid	n.d

Supplementary Table 5: Solvent Optimization:

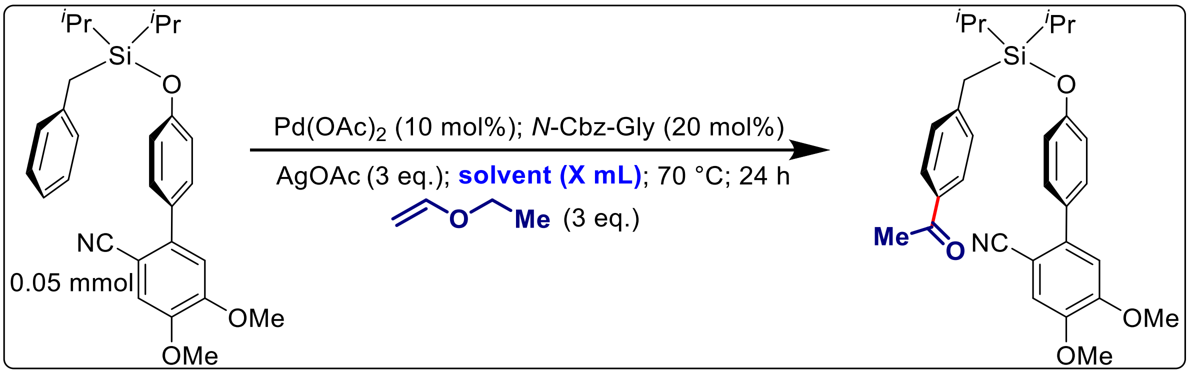

Entry	Solvent	Yield %	Selectivity (*para*: others)
1	HFIP (1 mL)	14	-
2	HFIP (0.75 mL)	48	15:1
**3**	**HFIP (0.6 mL)**	**60**	**15:1**
4	HFIP (0.3 mL)	trace	n.d
5	HFIP (1.5 mL)	trace	n.d
6	DCE	n.d	n.d
7	TFE	n.d	n.d
8	HFIP:DCE (1:1)	27	n.d
9	HFIP:TFE (1:1)	trace	n.d
10	MeCN	n.d	n.d
11	MeCN:H_2O (1:1)	n.d	n.d
12	*t*-BuOH	n.d	n.d
13	EtOH	n.d	n.d
14	HFIP:EtOH (1:1)	trace	n.d
15	EtOH:H_2O (1:1)	n.d	n.d
16	Acetone:H_2O (1:1)	n.d	n.d

17	AcOH	n.d	n.d

Supplementary Table 6: Oxidant Optimization:

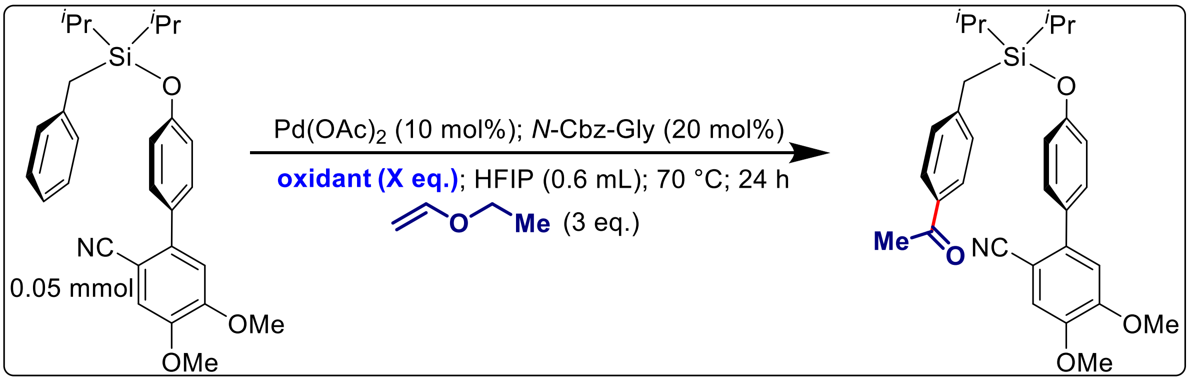

Entry	Oxidant	Yield %	Selectivity (*para*: others)
**1**	**Ag_2CO_3**	**68**	**16:1**
2	AgOAc	60	15:1
3	AgTFA	40	13:1
4	AgOTf	37	12:1
5	Ag_2SO_4	n.d	n.d
6	Ag_2O	n.d	n.d
7	AgF	n.d	n.d
8	AgI	n.d	n.d
9	$Cu(OAc)_2$	n.d	n.d
10	CuF_2	n.d	n.d
11	$Cu(acac)_2$	n.d	n.d
12	$Cu(NO_3)_2$, $3H_2O$	n.d	n.d
13	$Cu(BF_4)_2$, $4H_2O$	n.d	n.d
14	$AgNO_2$	n.d	n.d
15	BQ	n.d	n.d
16	$K_2S_2O_8$	n.d	n.d
17	Oxone	n.d	n.d

Supplementary Table 7: Temperature Optimization:

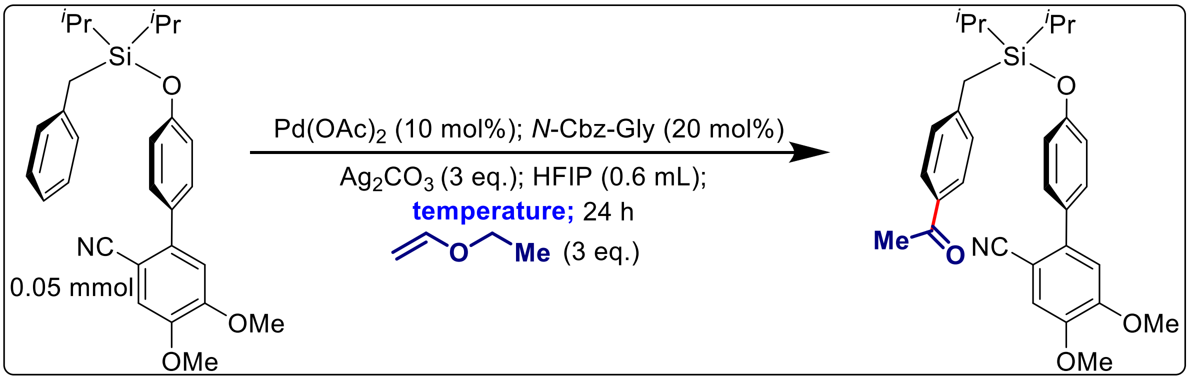

Entry	Temperature	Yield (%)	Selectivity (*para*: others)
1	40 °C	trace	-
2	50 °C	27	10:1
3	60 °C	48	16:1
4	70 °C	68	16:1
**5**	**80 °C**	**75**	**16:1**
6	90 °C	60	16:1
7	100 °C	37	-
8	110 °C	11	-

Supplementary Table 8: Time Optimization:

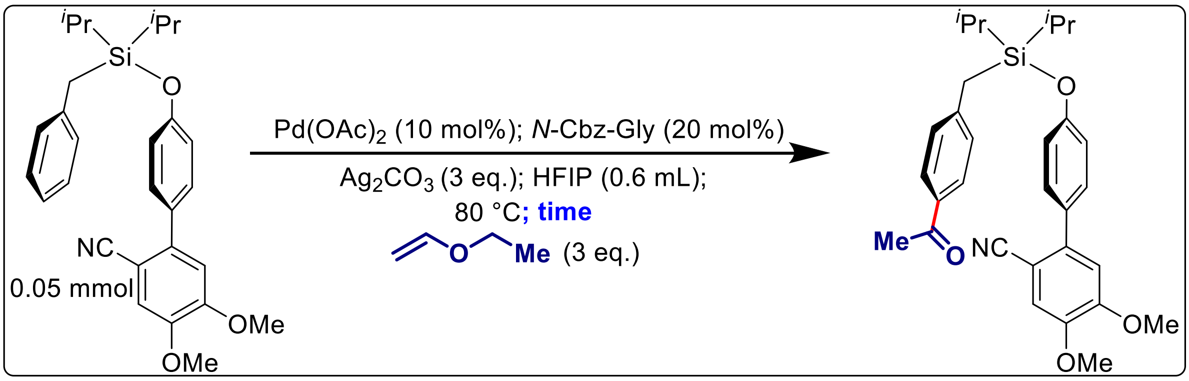

Entry	Time	Yield (%)	Selectivity (para: others)
1	12 h	68	16:1
**2**	**24 h**	**75**	**16:1**
**3**	**36 h**	**78**	**16:1**

Supplementary Table 9: Additive Optimization:

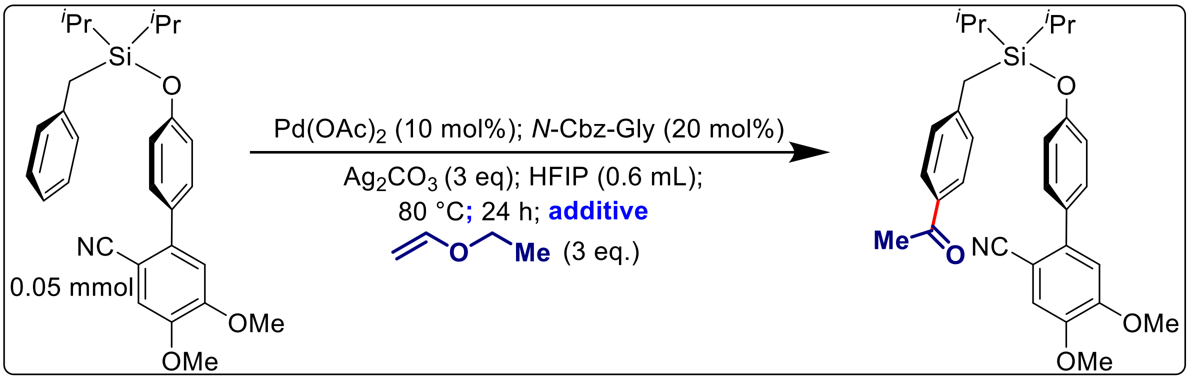

Entry	Additive (amount)	Yield (%)	Selectivity (*para*: others)
**1**	**-**	**75**	**16:1**
2	KOAc (2 eq.)	52	16:1
3	NH_4OAc (2 eq.)	60	16:1
4	NH_4Cl (2 eq.)	50	16:1
5	$ZnCl_2$ (50 mol%)	16	different from desired
6	$CaSO_4$ (2 eq.)	24	multiple products
10	MS (4 Å) (100 mg)	n.d	n.d
**11**	**NaOAc (2 eq.)**	**80**	**16:1**
12	NaOAc (1 eq.)	77	16:1
13	NaOAc (3 eq.)	70	16:1

Key Observations:

(a) *N*-Ac-Gly gives better yield and CBZ-Gly offers better selectivity.

(b) Changing reaction time from 24 h to 36 h give incremental increase in yield.

(c) Addition of NaOAc gives slight increase in yield.

<u>Supplementary Discussions</u>:

<u>General Procedure A</u> for *para*-Ketonisation:

In an oven-dried screw capped reaction tube was charged with magnetic stir-bar, benzylsilyl ether substrate (viscous benzylsilyl ether was weighed first), $Pd(OAc)_2$ (10 mol%), ligand (*N*-CBZ-Gly or *N*-Ac-Gly; 20 mol%), Ag_2CO_3 (3 eq.) and NaOAc (2 eq.). 1.2 mL (for 0.1 mmol scale) of 1,1,1,3,3,3-Hexafluoro-2-propanol (HFIP) was added followed by vinyl

ether (3 eq.). The reaction tube was capped and stirred (900 rpm) on a preheated oil-bath at 80 °C for 24/36 h. Upon completion the mixture was cooled and diluted with EtOAc and filtered through a celite pad. The filtrate was evaporation under reduced pressure and the crude mixture was purified by column chromatography using silica (100-200 mesh size) and petroleum ether/ ethyl acetate as the eluent. The selectivity was monitored using ^{1}H–NMR signal in presence of 1,3,5-trimethoxybenzene as internal standard. The regio-selectivity was determined from ^{1}H–NMR signals of aromatic region and benzylic position.

[*Note: The reaction is sensitive to the quality of HFIP. Freshly distilled solvent is recommended.]*

Synthesis of Substrates:

All the substrates were synthesized and characterized following literature reported methods.[1] New substrates synthesized are characterized as follows

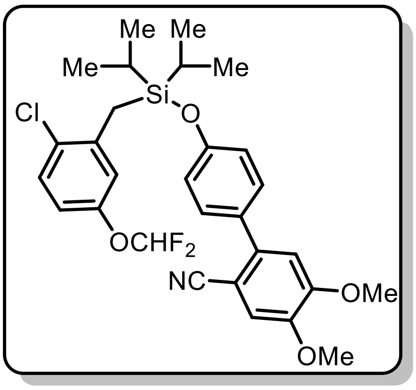

4'-(((2-chloro-5-(difluoromethoxy)benzyl)diisopropylsilyl)oxy)-4,5-dimethoxy-[1,1'-biphenyl]-2-carbonitrile:

^{1}H NMR (500 MHz, $CDCl_3$): δ 7.41 – 7.36 (m, 2H), 7.26 (s, 1H), 7.12 (s, 1H), 6.98 (t, *J* = 4.4 Hz, 1H), 6.89 (s, 1H), 6.86 – 6.82 (m, 2H), 6.80 (dd, *J* = 8.7, 2.8 Hz, 1H), 3.95 (s, 3H), 3.92 (s, 3H), 2.54 (s, 2H), 1.34 – 1.26 (m, 2H), 1.06 (dd, *J* = 9.4, 7.5 Hz, 12H).

^{13}C NMR (126 MHz, $CDCl_3$): δ 155.87, 152.69, 148.16, 147.01, 140.08, 139.31, 131.47, 130.59, 130.05, 129.94, 121.54, 119.81, 119.46, 118.03, 117.43, 115.96, 115.15, 113.89, 112.49, 102.24, 56.43, 56.32, 18.93, 17.56, 17.41, 13.57.

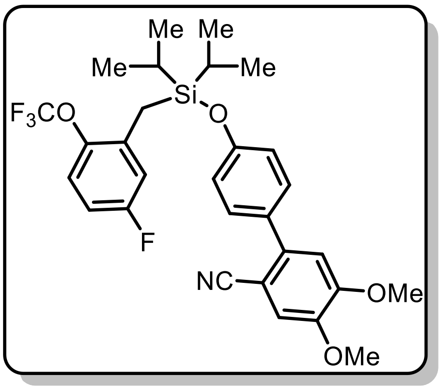

4'-(((5-fluoro-2-(trifluoromethoxy)benzyl)diisopropylsilyl)oxy)-4,5-dimethoxy-[1,1'-biphenyl]-2-carbonitrile:

^{1}H NMR (500 MHz, $CDCl_3$): δ 7.39 (t, *J* = 5.7 Hz, 2H), 7.16 – 7.13 (m, 1H), 7.13 (s, 1H), 6.93 (dd, *J* = 9.3, 3.1 Hz, 1H), 6.89 (s, 1H), 6.87 – 6.83 (m, 2H), 6.83 – 6.78 (m, 1H), 3.95 (s, 3H), 3.92 (s, 3H), 2.40 (s, 2H), 1.26 (dt, *J* = 14.8, 7.5 Hz, 2H), 1.05 (d, *J* = 7.4 Hz, 12H).

^{13}C NMR (126 MHz, $CDCl_3$): δ 161.50, 159.55, 155.80, 152.68, 148.16, 143.01, 140.07, 134.65, 134.59, 131.60, 130.10, 122.18, 122.10, 119.92, 119.44, 117.72, 117.51, 115.14, 112.80, 112.61, 112.49, 102.26, 56.42, 56.29, 17.40, 17.29, 15.11, 13.33.

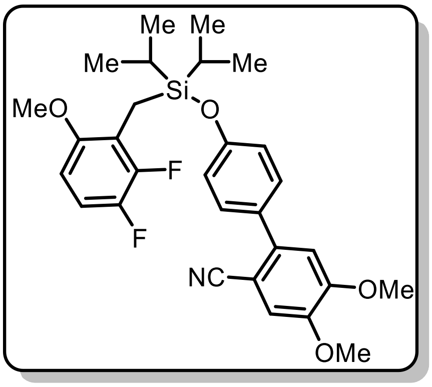

4'-(((2,3-difluoro-6-methoxybenzyl)diisopropylsilyl)oxy)-4,5-dimethoxy-[1,1'-biphenyl]-2-carbonitrile:

^{1}H NMR (500 MHz, $CDCl_3$): δ 7.34 (d, *J* = 8.1 Hz, 2H), 7.12 (s, 1H), 6.88 (s, 1H), 6.82 (dd, *J* = 18.2, 8.8 Hz, 3H), 6.42 (d, *J* = 7.7 Hz, 1H), 3.95 (s, 3H), 3.92 (s, 3H), 3.68 (s, 3H), 2.37 (s, 2H), 1.28 – 1.22 (m, 2H), 1.07 (d, *J* = 5.2 Hz, 12H).

^{13}C NMR (126 MHz, $CDCl_3$): δ 156.15, 152.70, 148.08, 140.33, 131.07, 129.81, 119.88, 119.50, 117.75, 117.63, 115.18, 112.59, 111.94, 111.80, 104.52, 104.50, 102.20, 56.46, 56.32, 55.67, 17.41, 17.38, 13.82, 8.13.

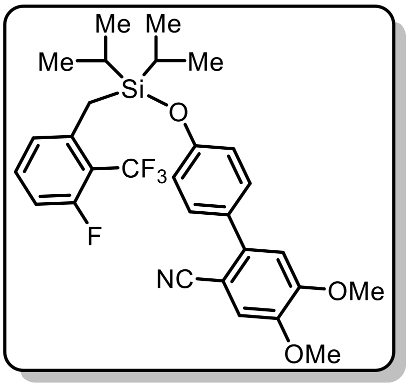

4'-(((3-fluoro-2-(trifluoromethyl)benzyl)diisopropylsilyl)oxy)-4,5-dimethoxy-[1,1'-biphenyl]-2-carbonitrile:

^{1}**H NMR** (500 MHz, $CDCl_3$): δ 7.38 (d, *J* = 8.6 Hz, 2H), 7.32 (dd, *J* = 13.6, 8.0 Hz, 1H), 7.13 (s, 1H), 7.07 (d, *J* = 7.8 Hz, 1H), 6.94 – 6.90 (m, 1H), 6.89 (s, 1H), 6.83 (d, *J* = 8.6 Hz, 2H), 3.96 (s, 3H), 3.93 (s, 3H), 2.64 (d, *J* = 1.9 Hz, 2H), 1.34 – 1.25 (m, 2H), 1.06 (d, *J* = 7.5 Hz, 6H), 1.00 (d, *J* = 7.4 Hz, 6H).

^{13}**C NMR** (126 MHz, $CDCl_3$): δ 155.88, 152.74, 148.22, 141.05, 140.14, 132.57, 132.49, 131.52, 130.09, 127.49, 127.46, 119.96, 119.48, 115.20, 113.39, 113.21, 112.53, 102.35, 56.48, 56.35, 19.25, 17.61, 17.34, 13.42.

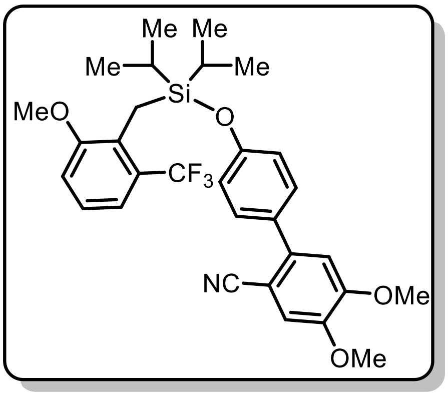

4'-((diisopropyl(2-methoxy-6-(trifluoromethyl)benzyl)silyl)oxy)-4,5-dimethoxy-[1,1'-biphenyl]-2-carbonitrile:

^{1}**H NMR** (500 MHz, $CDCl_3$): δ 7.33 (t, *J* = 5.7 Hz, 2H), 7.18 (d, *J* = 7.6 Hz, 1H), 7.15 – 7.10 (m, 2H), 6.88 (t, *J* = 4.0 Hz, 2H), 6.79 (t, *J* = 5.7 Hz, 2H), 3.95 (s, 3H), 3.93 (s, 3H), 3.70 (s, 3H), 2.58 (s, 2H), 1.25 (dd, *J* = 8.2, 4.6 Hz, 2H), 1.06 (d, *J* = 7.4 Hz, 6H), 1.02 (d, *J* = 7.4 Hz, 6H).

^{13}**C NMR** (126 MHz, $CDCl_3$): δ 157.57, 156.34, 152.69, 148.10, 140.40, 130.88, 129.75, 129.10, 128.87, 128.64, 127.91, 127.90, 125.89, 125.50, 123.71, 119.91, 119.55, 118.13, 118.08, 118.04, 117.99, 115.18, 112.82, 112.52, 102.26, 56.45, 56.31, 55.27, 17.59, 17.58, 14.05, 12.64.

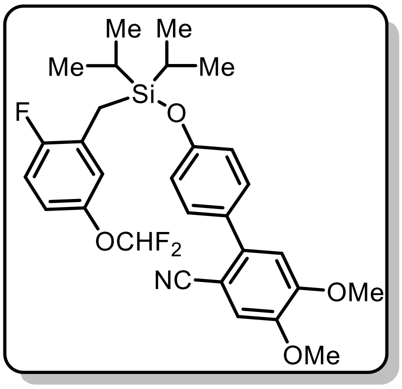

4'-(((5-(difluoromethoxy)-2-fluorobenzyl)diisopropylsilyl)oxy)-4,5-dimethoxy-[1,1'-biphenyl]-2-carbonitrile:

^{1}H NMR (500 MHz, $CDCl_3$): δ 7.42 – 7.36 (m, 2H), 7.13 (s, 1H), 6.95 (t, J = 9.0 Hz, 1H), 6.93 – 6.89 (m, 2H), 6.87 – 6.81 (m, 3H), 6.34 (dd, J = 78.6, 69.5 Hz, 1H), 3.96 (s, 3H), 3.93 (s, 3H), 2.36 (d, J = 1.3 Hz, 2H), 1.26 (ddd, J = 10.5, 9.6, 5.5 Hz, 2H), 1.09 – 1.05 (m, 12H).

^{13}C NMR (126 MHz, $CDCl_3$): δ 158.90, 156.98, 155.90, 152.73, 148.20, 146.83, 140.12, 131.56, 130.10, 129.97, 128.02, 127.87, 122.50, 122.46, 119.95, 119.48, 118.26, 117.73, 117.66, 116.27, 116.19, 116.07, 115.20, 114.12, 112.54, 102.32, 56.47, 56.33, 17.72, 17.52, 17.47, 17.40, 13.74, 13.72, 13.36.

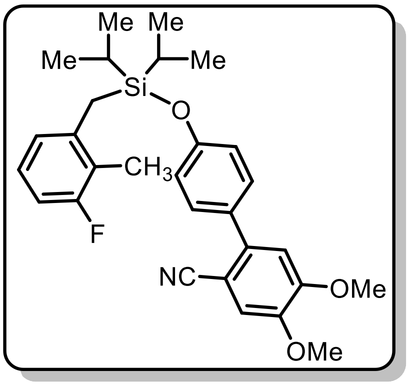

4'-(((3-fluoro-2-methylbenzyl)diisopropylsilyl)oxy)-4,5-dimethoxy-[1,1'-biphenyl]-2-carbonitrile:

^{1}H NMR (500 MHz, $CDCl_3$): δ 7.34 (t, J = 5.7 Hz, 2H), 7.12 (s, 1H), 7.02 (dd, J = 13.9, 7.8 Hz, 1H), 6.93 (d, J = 7.6 Hz, 1H), 6.88 (s, 1H), 6.78 (t, J = 8.8 Hz, 1H), 6.72 (t, J = 5.7 Hz, 2H), 3.95 (s, 3H), 3.92 (s, 3H), 2.39 (s, 2H), 2.19 (d, J = 2.0 Hz, 3H), 1.28 (dt, J = 14.9, 7.4 Hz, 2H), 1.08 (d, J = 7.5 Hz, 6H), 1.04 (d, J = 7.4 Hz, 6H).

^{13}C NMR (126 MHz, $CDCl_3$): δ 162.79, 160.86, 155.89, 152.69, 148.15, 140.25, 140.22, 140.18, 131.37, 129.96, 126.41, 126.34, 124.92, 124.90, 122.92, 122.80, 119.89, 119.46, 115.18, 112.52, 111.52, 111.33, 102.29, 56.45, 56.32, 18.25, 18.23, 17.71, 17.51, 13.39, 11.54, 11.49.

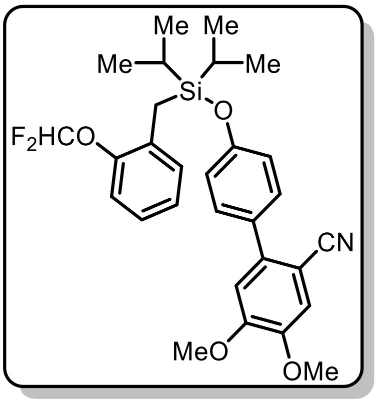

4'-(((2-(difluoromethoxy)benzyl)diisopropylsilyl)oxy)-4,5-dimethoxy-[1,1'-biphenyl]-2-carbonitrile:

^{1}H NMR (500 MHz, $CDCl_3$): δ 7.36 (d, *J* = 8.5 Hz, 2H), 7.21 (dd, *J* = 7.2, 1.8 Hz, 1H), 7.11 – 7.03 (m, 2H), 7.01 (d, *J* = 7.8 Hz, 1H), 6.88 (s, 1H), 6.79 (d, *J* = 8.5 Hz, 2H), 6.42 (t, *J* = 74.3 Hz, 1H), 3.95 (s, 3H), 3.92 (s, 3H), 2.43 (s, 2H), 1.27 (dt, *J* = 14.8, 7.5 Hz, 2H), 1.10 – 1.03 (m, 12H).

^{13}C NMR (126 MHz, $CDCl_3$): δ 156.06, 152.70, 149.33, 148.16, 140.20, 131.34, 131.23, 130.55, 129.99, 126.11, 125.30, 120.03, 119.50, 118.73, 118.19, 116.68, 115.18, 114.63, 112.51, 102.31, 56.46, 56.32, 17.53, 17.43, 14.65, 13.33.

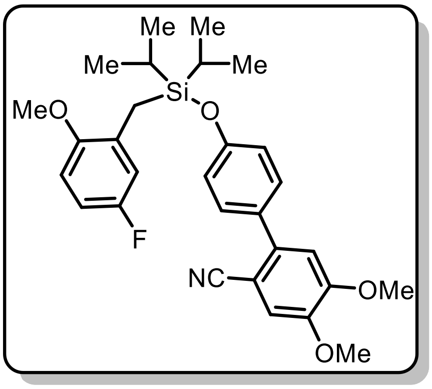

4'-(((5-fluoro-2-methoxybenzyl)diisopropylsilyl)oxy)-4,5-dimethoxy-[1,1'-biphenyl]-2-carbonitrile:

^{1}H NMR (500 MHz, $CDCl_3$): δ 7.37 (d, *J* = 8.5 Hz, 2H), 7.13 (s, 1H), 6.90 (s, 1H), 6.84 (d, *J* = 8.5 Hz, 2H), 6.79 (dd, *J* = 9.3, 3.0 Hz, 1H), 6.74 (td, *J* = 8.5, 3.1 Hz, 1H), 6.66 (dd, *J* = 8.9, 4.7 Hz, 1H), 3.95 (s, 3H), 3.92 (s, 3H), 3.70 (s, 3H), 2.37 (s, 2H), 1.22 (td, *J* = 14.7, 7.2 Hz, 2H), 1.05 (dd, *J* = 7.4, 1.4 Hz, 12H).

^{13}C NMR (126 MHz, $CDCl_3$): δ 157.93, 156.23, 156.04, 152.96, 152.69, 148.12, 140.28, 131.20, 129.91, 129.46, 129.40, 120.08, 119.51, 116.92, 116.74, 115.17, 112.54, 111.42, 111.24, 110.57, 110.50, 102.26, 56.44, 56.31, 55.51, 17.50, 17.46, 15.10, 13.47.

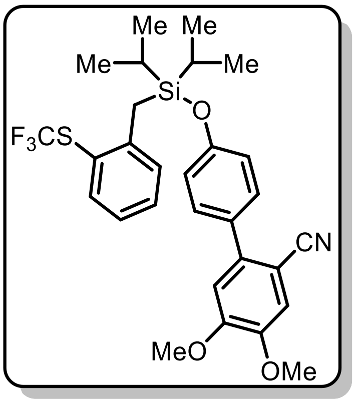

4'-((diisopropyl(2-((trifluoromethyl)thio)benzyl)silyl)oxy)-4,5-dimethoxy-[1,1'-biphenyl]-2-carbonitrile:

^{1}H NMR (500 MHz, $CDCl_3$): δ 7.63 (d, *J* = 7.8 Hz, 1H), 7.35 (dd, *J* = 4.9, 3.0 Hz, 4H), 7.16 (ddd, *J* = 8.0, 6.8, 4.1 Hz, 1H), 7.12 (s, 1H), 6.88 (s, 1H), 6.75 (d, *J* = 8.5 Hz, 2H), 3.95 (s, 3H), 3.92 (s, 3H), 2.78 (s, 2H), 1.27 (dt, *J* = 14.8, 7.4 Hz, 2H), 1.06 (dd, *J* = 15.8, 7.4 Hz, 12H).

^{13}C NMR (126 MHz, $CDCl_3$): δ 155.85, 152.71, 148.18, 145.34, 140.12, 138.51, 131.45, 131.31, 131.18, 130.65, 130.03, 128.72, 125.96, 123.30, 119.88, 119.45, 115.20, 112.51, 102.31, 56.46, 56.32, 19.90, 17.72, 17.52, 17.26, 13.71, 13.47, 13.22.

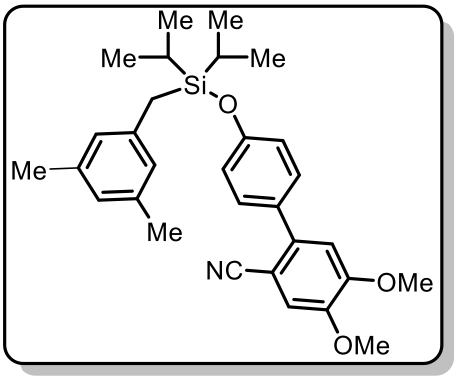

4'-(((3,5-dimethylbenzyl)diisopropylsilyl)oxy)-4,5-dimethoxy-[1,1'-biphenyl]-2-carbonitrile:

^{1}H NMR (500 MHz, $CDCl_3$): δ 7.44 – 7.38 (m, 2H), 7.14 (s, 1H), 6.91 (s, 1H), 6.90 – 6.86 (m, 2H), 6.73 (s, 3H), 3.96 (s, 3H), 3.93 (s, 3H), 2.32 (s, 2H), 2.23 (s, 6H), 1.22 (dt, *J* = 14.6, 7.3 Hz, 2H), 1.09 – 1.05 (m, 12H).

^{13}C NMR (126 MHz, $CDCl_3$): δ 156.33, 152.69, 148.13, 140.24, 138.30, 137.77, 131.27, 129.96, 126.98, 126.27, 120.28, 120.18, 119.50, 115.22, 112.52, 102.31, 56.46, 56.32, 23.94, 21.49, 20.82, 17.65, 17.63, 13.09.

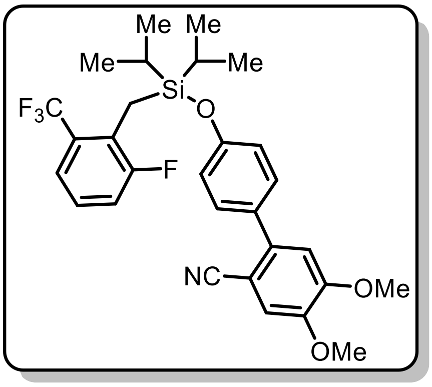

4'-(((2-fluoro-6-(trifluoromethyl)benzyl)diisopropylsilyl)oxy)-4,5-dimethoxy-[1,1'-biphenyl]-2-carbonitrile:

^{1}H NMR (500 MHz, $CDCl_3$): δ 7.34 (d, *J* = 8.3 Hz, 2H), 7.12 (s, 1H), 7.01 (dd, *J* = 15.1, 8.1 Hz, 1H), 6.88 (s, 1H), 6.82 (d, *J* = 8.5 Hz, 2H), 6.64 (t, *J* = 8.7 Hz, 1H), 6.55 (d, *J* = 8.2 Hz, 1H), 3.94 (s, 3H), 3.92 (s, 3H), 3.70 (d, *J* = 4.7 Hz, 3H), 2.36 (d, *J* = 1.7 Hz, 2H), 1.24 (dt, *J* = 13.3, 6.5 Hz, 3H), 1.09 – 1.05 (m, 12H).

^{13}C NMR (126 MHz, $CDCl_3$): δ 158.09, 156.33, 152.65, 148.06, 140.35, 130.90, 129.76, 125.57, 125.48, 119.97, 119.78, 119.52, 115.17, 112.50, 107.87, 107.69, 105.72, 102.22, 56.42, 56.29, 55.51, 17.41, 17.38, 13.75, 7.54, 7.51.

Synthesis of Vinyl Ethers:

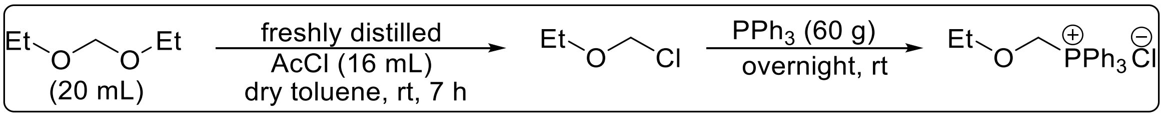

Procedure: In a three-neck round bottom flask (500 mL) dry toluene (60 mL) was taken under inert atmosphere and diethoxymethane or ethylal (20 mL) was added to it. Freshly distilled acetyl chloride (15.4 mL) was added to the mixture through dropping funnel over a period of 1 h. The mixture was stirred at room temperature for 6-7 h and triphenyl phosphine (60 g) was mixed and stirred for overnight. After initial clear solution, thick white solid precipitate formed. The reaction was filtered under suction and washed repeatedly with petroleum ether until a free-flowing solid forms. The solid was transferred to a round bottomed flask and suspended in toluene and warmed to 60 ºC for 3-4 h under reduced pressure (to remove moisture in the form of azeotrope). The wittig salt was then preserved in a air-tight container under nitrogen atmosphere.

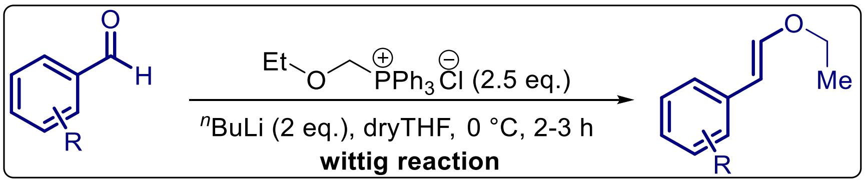

Procedure: In an over dried clean round bottomed flask 2.5 equivalent of wittig salt was taken (before every reaction above mentioned azeotrope treatment is recommended for better yield) and suspended in dry THF (6 mL) under inert atmosphere. The mixture was cooled to 0 ºC and butyllithium (2 eq.) was added to it. The solution color changed from white to brick red and

the solution was stirred for another 15 mins. The carbonyl substrate (2 mmol) was dissolved in dry THF added to the solution dropwise. After 5 mins the reaction was warmed to room temperature and stirred for 2-3 h. The progress was monitored by TLC. Upon completion the reaction was quenched with ice cooled water and extracted with ethyl acetate. The organic part was dried, concentrated and purified using column chromatography. [Note: vinyl ethers should be used ASAP for the reaction].

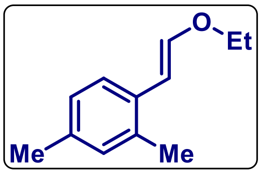

(E)-1-(2-ethoxyvinyl)-2,4-dimethylbenzene

^{1}H NMR (400 MHz, $CDCl_3$): δ 7.86 (d, *J* = 7.8 Hz, 1H), 7.18 (d, *J* = 7.8 Hz, 1H), 7.02 – 6.93 (m, 4H), 6.80 (d, *J* = 12.7 Hz, 1H), 6.26 – 6.21 (m, 1H), 5.97 (d, *J* = 12.7 Hz, 1H), 5.31 (d, *J* = 7.2 Hz, 1H), 3.94 (dq, *J* = 17.5, 7.1 Hz, 5H), 2.32 – 2.28 (m, 13H), 1.36 (td, *J* = 6.7, 4.2 Hz, 8H).

^{13}C NMR (101 MHz, $CDCl_3$): δ 147.96, 146.01, 135.68, 135.47, 134.82, 134.79, 132.49, 131.60, 131.15, 130.78, 129.04, 126.92, 126.50, 124.92, 104.19, 102.51, 69.01, 65.79, 34.83, 31.83, 29.32, 27.19, 25.50, 22.89, 15.06, 14.32.

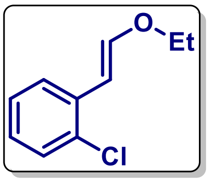

(E)-1-chloro-2-(2-ethoxyvinyl)benzene

^{1}H NMR (500 MHz, $CDCl_3$): δ 7.35 (t, *J* = 8.3 Hz, 2H), 7.19 – 7.14 (m, 1H), 7.11 – 7.05 (m, 1H), 6.99 (t, *J* = 11.8 Hz, 1H), 6.16 (d, *J* = 12.9 Hz, 1H), 3.96 (q, *J* = 7.0 Hz, 2H), 1.40 – 1.36 (m, 3H).

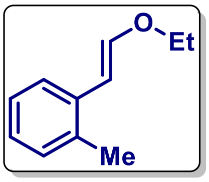

(E)-1-(2-ethoxyvinyl)-2-methylbenzene

^{1}H NMR (500 MHz, $CDCl_3$): δ 8.02 (d, *J* = 7.7 Hz, 1H), 7.33 (d, *J* = 7.1 Hz, 1H), 7.25 – 7.09 (m, 6H), 6.88 (d, *J* = 12.7 Hz, 1H), 6.30 (d, *J* = 7.2 Hz, 1H), 6.04 (d, *J* = 12.7 Hz, 1H), 5.38 (d, *J* = 7.2 Hz, 1H), 3.98 (dq, *J* = 21.2, 7.0 Hz, 4H), 2.37 (d, *J* = 4.8 Hz, 6H), 1.40 (dd, *J* = 12.8, 6.9 Hz, 6H).

^{13}C NMR (126 MHz, $CDCl_3$): δ 148.44, 146.50, 135.43, 134.93, 134.48, 130.27, 130.00, 129.06, 126.21, 126.07, 124.83, 104.29, 102.52, 69.10, 65.80, 20.37, 20.22, 15.56, 15.01.

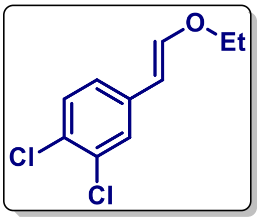

(E)-1,2-dichloro-4-(2-ethoxyvinyl)benzene

^{1}H NMR (400 MHz, $CDCl_3$): δ 7.72 (d, *J* = 2.0 Hz, 1H), 7.38 (dd, *J* = 8.4, 2.0 Hz, 1H), 7.33 – 7.26 (m, 3H), 7.04 – 7.00 (m, 1H), 6.97 (d, *J* = 12.9 Hz, 1H), 6.24 (t, *J* = 5.8 Hz, 1H), 5.76 – 5.67 (m, 1H), 5.12 (d, *J* = 7.0 Hz, 1H), 4.00 (q, *J* = 7.1 Hz, 2H), 3.89 (q, *J* = 7.0 Hz, 2H), 1.36 (dt, *J* = 10.4, 7.1 Hz, 6H).

^{13}C NMR (101 MHz, $CDCl_3$): δ 149.45, 148.18, 137.13, 136.44, 132.63, 132.09, 130.51, 130.06, 129.79, 127.57, 126.64, 124.35, 104.05, 103.37, 69.69, 66.01, 22.85, 15.58, 14.94, 14.31.

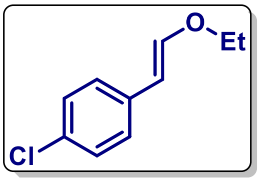

(E)-1-chloro-4-(2-ethoxyvinyl)benzene

^{1}H NMR (500 MHz, $CDCl_3$): δ 7.53 (d, *J* = 8.6 Hz, 2H), 7.26 – 7.23 (m, 2H), 7.23 – 7.20 (m, 1H), 7.18 – 7.13 (m, 1H), 6.97 (d, *J* = 13.0 Hz, 1H), 6.22 (d, *J* = 7.0 Hz, 1H), 5.18 (d, *J* = 7.0 Hz, 1H), 3.99 (q, *J* = 7.1 Hz, 2H), 3.90 (q, *J* = 7.0 Hz, 1H), 1.36 (dt, *J* = 11.0, 7.1 Hz, 5H).

^{13}C NMR (126 MHz, $CDCl_3$): δ 148.53, 147.18, 135.31, 134.80, 131.12, 131.05, 129.54, 128.80, 128.41, 126.37, 105.08, 104.54, 69.45, 65.85, 15.63, 15.00.

Characterization of Acylated Product:

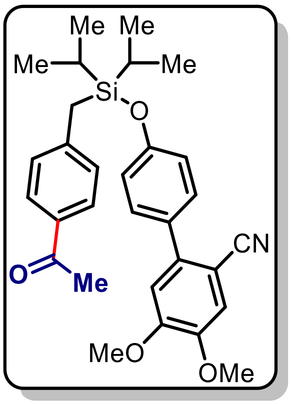

4'-((4-acetylbenzyl)diisopropylsilyloxy)-4,5-dimethoxybiphenyl-2-carbonitrile (1a & 2a): *Para*-ketonisation has been done following the general procedure A in 0.1 mmol scale.

R_f value: 0.30 (20% ethyl acetate in pet ether)

Column material: 100-200 mesh silica

Eluent: pet ether:ethyl acetate (90: 10)

Yield: 80% (40 mg); (brsm 95%)

Physical appearance: Colorless viscous liquid

^{1}H NMR (500 MHz, $CDCl_3$): δ 7.82 (d, *J* = 8.2 Hz, 2H), 7.38 (d, *J* = 8.5 Hz, 2H), 7.20 (d, *J* = 8.2 Hz, 2H), 7.13 (s, 1H), 6.89 (s, 1H), 6.86 (d, *J* = 8.5 Hz, 2H), 3.96 (s, 3H), 3.93 (s, 3H), 2.55 (s, 3H), 2.47 (s, 2H), 1.25 – 1.20 (m, 2H), 1.05 (d, *J* = 7.2 Hz, 12H).

^{13}C NMR (126 MHz, $CDCl_3$): δ 198.02, 155.96, 152.77, 148.25, 145.46, 140.13, 134.03, 131.63, 130.11, 129.11, 128.74, 120.11, 119.47, 115.23, 112.54, 102.36, 56.48, 56.36, 26.65, 22.01, 17.61, 17.56, 13.18.

HRMS: Calculated mass for $[M+H]^+$: 502.2408; observed mass: 502.2409

IR (cm^{-1}): 2932, 2868, 2218, 1679, 1601, 1500, 1463, 1353, 1263, 1241, 1172, 1136.

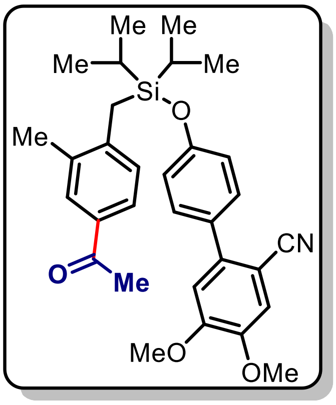

4'-((4-acetyl-2-methylbenzyl)diisopropylsilyloxy)-4,5-dimethoxybiphenyl-2-carbonitrile (2b): *Para*-ketonisation has been done following the general procedure A in 0.1 mmol scale.

R_f value: 0.30 (20% ethyl acetate in pet ether)

Column material: 100-200 mesh silica

Eluent: pet ether:ethyl acetate (90: 10)

Yield: 81% (41.7 mg); (brsm 96%)

Physical appearance: Colorless viscous liquid

^{1}H NMR (500 MHz, $CDCl_3$) δ 7.70 (d, *J* = 8.4 Hz, 1H), 7.67 (s, 1H), 7.31 (d, *J* = 8.6 Hz, 2H), 7.21 (d, *J* = 7.8 Hz, 1H), 7.12 (d, *J* = 5.6 Hz, 1H), 6.86 (s, 1H), 6.69 (d, *J* = 8.6 Hz, 2H), 3.95 (s, 3H), 3.92 (s, 3H), 2.55 (s, 3H), 2.44 (s, 2H), 2.35 (s, 3H), 1.32 – 1.27 (m, 2H), 1.09 (d, *J* = 7.5 Hz, 6H), 1.04 (d, *J* = 7.4 Hz, 6H).

^{13}C NMR (126 MHz, $CDCl_3$) δ 198.35, 155.77, 152.74, 148.21, 144.39, 140.14, 136.02, 134.18, 131.48, 130.48, 130.00, 129.51, 126.25, 119.86, 119.49, 115.18, 112.50, 102.32, 56.48, 56.37, 26.70, 20.68, 19.21, 17.73, 17.53, 13.52.

HRMS: Calculated mass for $[M+H]^+$: 516.2565; observed mass: 516.2563

IR (cm^{-1}): 2945, 2868, 2218, 1677, 1601, 1500, 1463, 1444, 1353, 1261, 1217, 1138, 1027, 915.

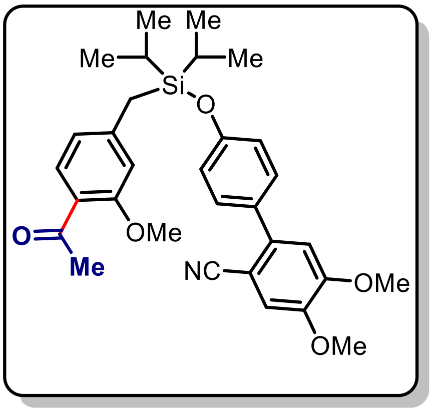

4'-((4-acetyl-3-methoxybenzyl)diisopropylsilyloxy)-4,5-dimethoxybiphenyl-2-carbonitrile (2c):

Para-ketonisation has been done following the general procedure A in 0.1 mmol scale.

R_f value: 0.29 (20% ethyl acetate in pet ether)

Column material: 100-200 mesh silica

Eluent: pet ether:ethyl acetate (89: 11)

Yield: 70% (37.2 mg); (brsm 93%)

Physical appearance: Colorless viscous liquid

^{1}H NMR (500 MHz, $CDCl_3$) δ 7.91 (s, 1H), 7.40 (d, *J* = 8.6 Hz, 2H), 7.13 (s, 1H), 6.93 – 6.88 (m, 2H), 6.87 (d, *J* = 5.5 Hz, 2H), 6.81 (s, 1H), 3.96 (s, 3H), 3.93 (s, 3H), 3.71 (s, 3H), 2.65 (s, 2H), 2.54 (s, 3H), 1.36 – 1.31 (m, 2H), 1.08 (dd, *J* = 15.3, 6.4 Hz, 12H).

^{13}C NMR (126 MHz, $CDCl_3$) δ 197.70, 158.21, 155.94, 152.83, 148.32, 145.83, 140.00, 134.61, 131.62, 130.22, 126.30, 119.88, 119.48, 115.32, 115.26, 113.76, 112.53, 102.33, 56.52, 56.40, 55.88, 31.97, 22.59, 17.71, 17.54, 13.78.

HRMS: Calculated mass for $[M+H]^+$: 532.2514; observed mass: 532.2513

IR (cm^{-1}): 2924, 2853, 2219, 1674, 1601, 1502, 1463, 1384, 1261, 1243, 1217, 915.

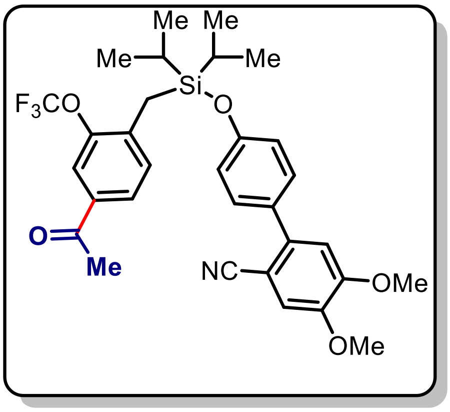

4'-((4-acetyl-2-(trifluoromethoxy)benzyl)diisopropylsilyloxy)-4,5-dimethoxybiphenyl-2-carbonitrile (2d):

Para-ketonisation has been done following the general procedure A in 0.1 mmol scale.

R$_f$ value: 0.29 (20% ethyl acetate in pet ether)

Column material: 100-200 mesh silica

Eluent: pet ether:ethyl acetate (89: 11)

Yield: 75% (37.2 mg); (brsm 91%)

Physical appearance: Colorless viscous liquid

^{1}H NMR (500 MHz, $CDCl_3$) δ 7.78 (s, 1H), 7.72 (dd, *J* = 8.1, 1.6 Hz, 1H), 7.39 – 7.36 (m, 2H), 7.30 (d, *J* = 8.1 Hz, 1H), 7.13 (s, 1H), 6.88 (s, 1H), 6.84 – 6.81 (m, 2H), 3.96 (s, 2H), 3.93 (s, 3H), 2.56 (s, 3H), 2.49 (s, 2H), 1.30 – 1.26 (m, 2H), 1.05 (dd, *J* = 7.4, 3.1 Hz, 12H).

^{13}C NMR (126 MHz, $CDCl_3$) δ 196.62, 155.79, 152.79, 148.28, 147.21, 140.11, 138.27, 135.55, 135.01, 131.92, 131.68, 131.51, 130.12, 130.04, 126.67, 126.51, 119.94, 119.82, 119.48, 115.22, 112.54, 102.37, 56.49, 56.37, 26.66, 17.46, 17.36, 15.84, 13.50.

HRMS: Calculated mass for $[M+H]^+$: 586.2231; observed mass: 586.2230

IR (cm^{-1}): 2947, 2870, 2220, 1687, 1605, 1504, 1467, 1355, 1262, 1219, 1174.

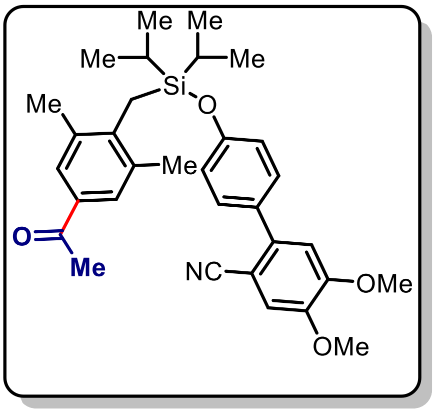

4'-((4-acetyl-2,6-dimethylbenzyl)diisopropylsilyloxy)-4,5-dimethoxybiphenyl-2-carbonitrile (2e):

Para-ketonisation has been done following the general procedure A in 0.1 mmol scale.

R_f value: 0.33 (20% ethyl acetate in pet ether)

Column material: 100-200 mesh silica

Eluent: pet ether:ethyl acetate (91: 9)

Yield: 82% (43.4 mg); (brsm 92%)

Physical appearance: Colorless viscous liquid

NMR:

^{1}H NMR (400 MHz, $CDCl_3$): δ 7.56 (s, 2H), 7.34 – 7.28 (m, 2H), 7.12 (s, 1H), 6.86 (s, 1H), 6.75 – 6.69 (m, 2H), 3.95 (s, 3H), 3.92 (s, 3H), 2.53 (s, 3H), 2.47 (s, 2H), 2.39 (s, 6H), 1.30 – 1.27 (m, 2H), 1.10 (d, *J* = 7.4 Hz, 6H), 0.97 (d, *J* = 7.3 Hz, 6H).

^{13}C NMR (101 MHz, $CDCl_3$): δ 198.58, 155.75, 152.77, 148.23, 143.65, 140.14, 135.96, 133.63, 131.43, 129.97, 128.24, 119.73, 119.48, 115.22, 112.52, 102.34, 56.49, 56.37, 29.90, 26.66, 21.65, 17.66, 17.42, 14.33.

HRMS: Calculated mass for $[M+H]^+$: 530.2721; observed mass: 530.2722

IR (cm^{-1}): 2943, 2866, 2220, 1679, 1602, 1501, 1463, 1444, 1350, 1261, 1218, 1137.

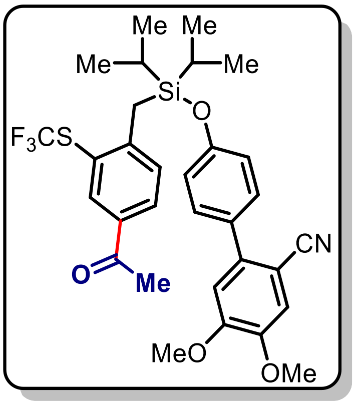

4'-(((4-acetyl-2-((trifluoromethyl)thio)benzyl)diisopropylsilyl)oxy)-4,5-dimethoxy-[1,1'-biphenyl]-2-carbonitrile (2f):

Para-ketonisation has been done following the general procedure A in 0.05 mmol scale.

R_f value: 0.32 (20% ethyl acetate in pet ether)

Column material: 100-200 mesh silica

Eluent: pet ether:ethyl acetate (91: 9)

Yield: 68% (20.4 mg); (brsm 90%)

Physical appearance: Colorless viscous liquid

^{1}H NMR (400 MHz, $CDCl_3$): δ 8.21 (s, 1H), 7.93 (dd, J = 8.1, 1.9 Hz, 1H), 7.42 (d, J = 8.2 Hz, 1H), 7.34 (d, J = 8.6 Hz, 2H), 7.12 (s, 1H), 6.87 (s, 1H), 6.74 (d, J = 8.6 Hz, 2H), 3.96 (s, 3H), 3.93 (s, 3H), 2.85 (s, 2H), 2.58 (s, 3H), 1.33 – 1.29 (m, 2H), 1.08 (d, J = 7.4 Hz, 6H), 1.05 (d, J = 7.4 Hz, 6H).

^{13}C NMR (126 MHz, $CDCl_3$): δ 196.71, 155.60, 152.78, 151.61, 148.28, 140.03, 138.60, 135.14, 131.73, 130.85, 130.77, 130.14, 130.05, 124.06, 119.77, 119.46, 115.21, 112.52, 102.37, 56.50, 56.39, 26.68, 21.08, 17.74, 17.53, 13.64.

HRMS: Calculated mass for $[M+H]^+$: 602.2003; observed mass: 602.2000

IR (cm^{-1}): 2924, 2870, 2220, 1687, 1601, 1502, 1463, 1248, 1113, 915.

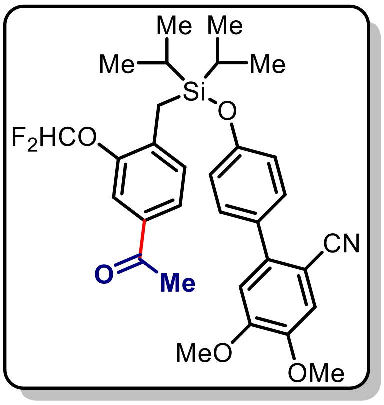

4'-(((4-acetyl-2-(difluoromethoxy)benzyl)diisopropylsilyl)oxy)-4,5-dimethoxy-[1,1'-biphenyl]-2-carbonitrile (2g):

Para-ketonisation has been done following the general procedure A in 0.1 mmol scale.

R_f value: 0.31 (20% ethyl acetate in pet ether)

Column material: 100-200 mesh silica

Eluent: pet ether:ethyl acetate (90: 10)

Yield: 58% (32.9 mg); (brsm 89%)

Physical appearance: Colorless viscous liquid

^{1}H NMR (500 MHz, $CDCl_3$): δ 7.66 (dd, J = 8.0, 1.6 Hz, 1H), 7.60 (s, 1H), 7.37 – 7.34 (m, 2H), 7.13 (s, 1H), 6.88 (s, 1H), 6.80 – 6.78 (t, J = 75 Hz, 1H), 6.67 – 6.36 (m, 2H), 3.96 (s, 3H), 3.93 (s, 3H), 2.56 (s, 3H), 2.50 (s, 2H), 1.30 – 1.27 (m, 2H), 1.07 (t, J = 7.3 Hz, 12H).

^{13}C NMR (126 MHz, $CDCl_3$): δ 196.98, 155.83, 152.76, 149.32, 148.24, 140.13, 137.22, 135.46, 131.60, 131.21, 130.08, 125.50, 119.95, 119.51, 117.05, 116.31(t, J = 260 Hz), 115.14, 112.49, 102.34, 56.49, 56.38, 26.71, 17.54, 17.42, 15.78, 13.50.

HRMS: Calculated mass for $[M+H]^+$ = 568.2325; observed mass: 563.2322

IR (cm^{-1}): 3021, 2219, 1701, 1654, 1559, 1507, 1217.

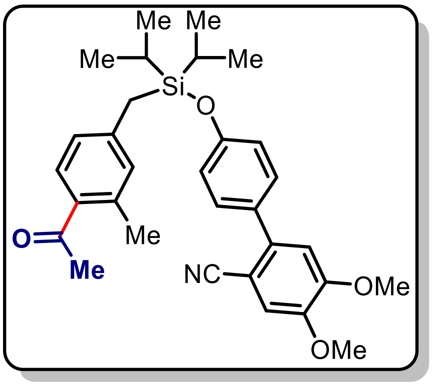

4'-((4-acetyl-3-methylbenzyl)diisopropylsilyloxy)-4,5-dimethoxybiphenyl-2-carbonitrile (2h): *Para*-ketonisation has been done following the general procedure A in 0.1 mmol scale.

R_f value: 0.30 (20% ethyl acetate in pet ether)

Column material: 100-200 mesh silica

Eluent: pet ether:ethyl acetate (90: 10)

Yield: 69% (35.5 mg); (brsm 95%)

Physical appearance: Colorless viscous liquid

^{1}H NMR (500 MHz, $CDCl_3$) δ 7.50 (d, *J* = 10.0 Hz, 2H), 7.38 (dd, *J* = 9.1, 2.5 Hz, 2H), 7.13 (s, 2H), 6.90 (s, 1H), 6.88 – 6.83 (m, 2H), 3.96 (s, 2H), 3.93 (s, 3H), 2.50 (s, 2H), 2.41 (s, 2H), 2.32 (s, 2H), 1.25 – 1.19 (m, 2H), 1.06 (d, *J* = 7.3 Hz, 11H).

^{13}C NMR (126 MHz, $CDCl_3$) δ 198.84, 156.10, 152.78, 148.23, 140.13, 139.22, 138.39, 137.38, 134.69, 132.92, 131.48, 130.09, 126.23, 125.45, 120.09, 115.26, 112.56, 102.31, 56.49, 56.37, 26.92, 21.51, 20.95, 17.67, 17.61, 13.18.

HRMS: Calculated mass for $[M+H]^+$: 516.2565; observed mass: 516.2565

IR (cm^{-1}): 2950, 2868, 2220, 1689, 1603, 1501, 1463, 1341, 1263, 1216, 1179,1038.

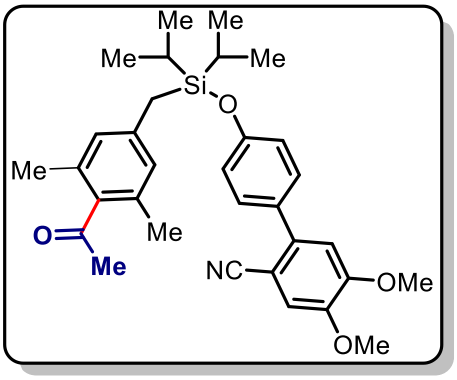

4'-((4-acetyl-3,5-dimethylbenzyl)diisopropylsilyloxy)-4,5-dimethoxybiphenyl-2-carbonitrile (2i): *Para*-ketonisation has been done following the general procedure A in 0.05 mmol scale.

R_f value: 0.33 (20% ethyl acetate in pet ether)

Column material: 100-200 mesh silica

Eluent: pet ether:ethyl acetate (91: 9)

Yield: 65% (17.2 mg); (brsm 96%)

Physical appearance: Colorless viscous liquid

^{1}H NMR (500 MHz, $CDCl_3$): δ 7.38 (dd, J = 9.0, 2.4 Hz, 2H), 7.13 (s, 1H), 6.89 (s, 1H), 6.84 (dd, J = 6.7, 4.8 Hz, 2H), 6.80 (s, 2H), 3.95 (s, 3H), 3.93 (s, 3H), 2.36 (d, J = 1.3 Hz, 3H), 2.31 (s, 2H), 2.13 (s, 6H), 1.24 – 1.20 (m, 2H), 1.06 (dd, J = 7.4, 5.5 Hz, 12H).

^{13}C NMR (126 MHz, $CDCl_3$): δ 191.38, 162.03, 156.26, 152.78, 148.24, 140.19, 138.84, 137.95, 133.11, 131.42, 130.95, 130.01, 128.42, 120.27, 119.51, 115.30, 112.54, 102.36, 56.51, 56.37, 20.59, 19.71, 19.15, 17.68, 17.63, 13.13.

HRMS: Calculated mass for $[M+H]^+$: 530.2721; observed mass: 530.2721

IR (cm^{-1}): 2943, 2866, 2220, 1679, 1602, 1501, 1463, 1444, 1350, 1261, 1218, 1137.

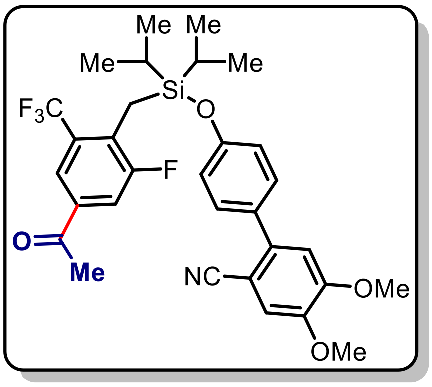

4'-((4-acetyl-2-fluoro-6-(trifluoromethyl)benzyl)diisopropylsilyloxy)-4,5-dimethoxybiphenyl -2-carbonitrile (3a): *Para*-ketonisation has been done following the general procedure A in 0.05 mmol scale.

R_f value: 0.32 (20% ethyl acetate in pet ether)

Column material: 100-200 mesh silica

Eluent: pet ether:ethyl acetate (91: 9)

Yield: 77% (22.6 mg); (brsm 96%)

Physical appearance: Colorless viscous liquid

^{1}H NMR (500 MHz, $CDCl_3$): δ 7.90 (d, *J* = 14.8 Hz, 1H), 7.56 (s, 1H), 7.39 (d, *J* = 8.5 Hz, 2H), 7.13 (s, 1H), 6.90 (s, 1H), 6.85 (d, *J* = 8.4 Hz, 2H), 3.97 (s, 2H), 3.93 (s, 3H), 2.55 (s, 3H), 2.52 (s, 2H), 1.26 (d, *J* = 6.4 Hz, 2H), 1.06 (dd, *J* = 12.9, 5.8 Hz, 12H).

^{13}C NMR (126 MHz, $CDCl_3$): δ 197.06, 155.75, 152.82, 148.31, 141.18, 140.02, 137.70, 131.95, 131.80, 131.42, 131.16, 130.24, 129.94, 124.01 (q, *J* = 273.42 Hz), 121.59 (q, *J* = 3.78 Hz), 119.87, 119.76, 119.44, 115.29, 112.60, 102.36, 56.51, 56.37, 26.86, 21.22, 17.64, 17.55, 13.28.

HRMS: Calculated mass for $[M+H]^+$ = 588.2188 ; observed mass: 580.2189

IR (cm^{-1}): 2958, 2870, 2220, 1690, 1603, 1502, 1243, 1123, 1023.

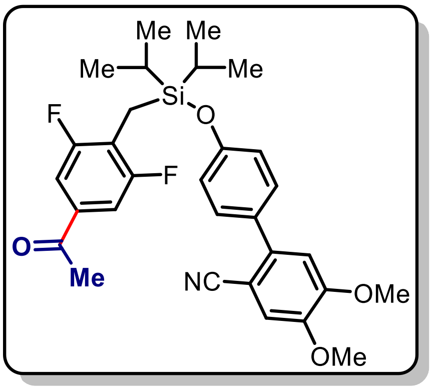

4'-((4-acetyl-2,6-difluorobenzyl)diisopropylsilyloxy)-4,5-dimethoxybiphenyl-2-carbonitrile (3b): *Para*-ketonisation has been done following the general procedure A in 0.1 mmol scale.

R$_f$ value: 0.32 (20% ethyl acetate in pet ether)

Column material: 100-200 mesh silica

Eluent: pet ether:ethyl acetate (91: 9)

Yield: 74% (39.7 mg); (brsm 82%)

Physical appearance: Colorless viscous liquid

^{1}H NMR (500 MHz, $CDCl_3$) δ 7.41 (d, *J* = 7.5 Hz, 2H), 7.35 (d, *J* = 8.5 Hz, 2H), 7.12 (s, 1H), 6.87 (s, 1H), 6.82 (d, *J* = 8.5 Hz, 2H), 3.96 (s, 3H), 3.93 (s, 3H), 2.54 (s, 3H), 2.41 (s, 2H), 1.33-1.28 (m, 2H), 1.09 (d, *J* = 7.4 Hz, 12H).

^{13}C NMR (126 MHz, $CDCl_3$) δ 195.68, 161.82 (d, *J* = 8.82 Hz), 159.83 (d, *J* = 6 Hz),, 159.81, 155.74, 152.78, 148.24, 140.17, 135.31, 131.55, 130.01, 121.83, 121.66, 119.87, 119.50, 115.25, 112.55, 111.02 (d, *J* = 13.86 Hz), 111.01 (d, *J* = 26.5 Hz), 102.35, 56.50, 56.38, 29.92, 26.64, 17.38, 17.32, 13.78, 8.28.

HRMS: Calculated mass for $[M+H]^+$ = 538.2220; observed mass: 538.2219.

IR (cm^{-1}): 2926, 2219, 1690, 1601, 1465, 1262, 1174, 1136, 1025.

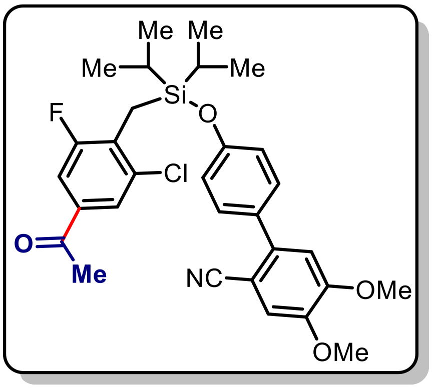

4'-((4-acetyl-2-chloro-6-fluorobenzyl)diisopropylsilyloxy)-4,5-dimethoxybiphenyl-2-carbonitrile (3c): *Para*-ketonisation has been done following the general procedure A in 0.1 mmol scale.

R$_f$ value: 0.32 (20% ethyl acetate in pet ether)

Column material: 100-200 mesh silica

Eluent: pet ether:ethyl acetate (91: 9)

Yield: 66% (36.5 mg); (brsm 94%)

Physical appearance: Colorless viscous liquid

^{1}H NMR (500 MHz, $CDCl_3$): δ 7.71 (s, 1H), 7.50 – 7.46 (m, 1H), 7.32 (d, *J* = 8.5 Hz, 2H), 7.12 (s, 1H), 6.86 (s, 1H), 6.79 (d, *J* = 8.6 Hz, 2H), 3.95 (s, 2H), 3.92 (s, 2H), 2.60 (d, 2H), 2.53 (s, 3H), 1.32-1.37 (m, 2H), 1.10 (d, *J* = 7.2 Hz, 4H), 1.09 – 1.07 (m, 6H).

^{13}C NMR (126 MHz, $CDCl_3$): δ 195.63, 161.72, 159.75, 155.72, 152.80, 148.26, 140.18, 135.42 (d, *J* = 7.56 Hz), 134.83 (d, *J* = 7.56 Hz), 132.54 (d, *J* = 18.9 Hz), 131.46, 129.95, 125.47, 125.44, 119.78, 119.48, 115.28, 113.20 (d, *J* = 23.94 Hz), 112.56, 102.36, 56.51, 56.38, 26.63, 17.50, 17.40, 14.15, 13.40.

HRMS: Calculated mass for $[M+Na]^+$ = 576.1744; observed mass: 576.1758

IR (cm^{-1}): 2924, 2220, 1688, 1603, 1463, 1262, 1174, 1136, 1026.

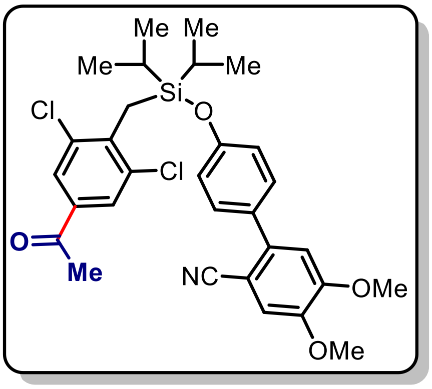

4'-((4-acetyl-2,6-dichlorobenzyl)diisopropylsilyloxy)-4,5-dimethoxybiphenyl-2-carbonitrile (3d): *Para*-ketonisation has been done following the general procedure A in 0.1 mmol scale.

R_f value: 0.32 (20% ethyl acetate in pet ether)

Column material: 100-200 mesh silica

Eluent: pet ether:ethyl acetate (91: 9)

Yield: 65% (40 mg); (brsm 87%)

Physical appearance: Colorless viscous liquid

^{1}H NMR (500 MHz, $CDCl_3$) δ 7.78 (s, 1H), 7.30 (d, *J* = 8.5 Hz, 1H), 7.11 (s, 1H), 6.84 (s, 1H), 6.79 (d, *J* = 8.6 Hz, 1H), 3.96 (s, 2H), 3.92 (s, 2H), 2.83 (s, 1H), 2.51 (s, 1H), 1.41 (dd, *J* = 15.0, 7.5 Hz, 1H), 1.13 (d, *J* = 7.5 Hz, 3H), 1.08 (d, *J* = 7.4 Hz, 3H).

^{13}C NMR (126 MHz, $CDCl_3$) δ 195.60, 155.67, 152.74, 148.18, 142.75, 140.14, 135.15, 134.91, 131.30, 129.85, 127.92, 119.67, 115.17, 112.47, 102.26, 56.47, 56.37, 26.62, 19.18, 17.60, 17.49, 14.59.

HRMS: Calculated mass for $[M+H]^+$ 570.1629; observed mass: 570.1626.

IR (cm^{-1}): 2950, 2870, 2220, 1689, 1605, 1504, 1467, 1389, 1243, 1217, 1135, 917.

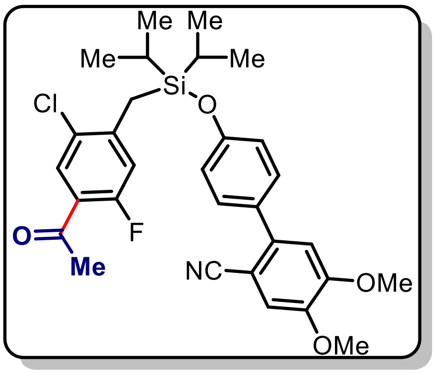

4'-((4-acetyl-2-chloro-5-fluorobenzyl)diisopropylsilyloxy)-4,5-dimethoxybiphenyl-2-carbonitrile (3e): *Para*-ketonisation has been done following the general procedure A in 0.05 mmol scale.

R$_f$ value: 0.32 (20% ethyl acetate in pet ether)

Column material: 100-200 mesh silica

Eluent: pet ether:ethyl acetate (91: 9)

Yield: 67% (18.5 mg); (brsm 87%)

Physical appearance: Colorless viscous liquid

^{1}H NMR (400 MHz, $CDCl_3$): δ 7.84 (d, *J* = 7.0 Hz, 1H), 7.40 (d, *J* = 8.6 Hz, 2H), 7.13 (s, 1H), 7.00 (d, *J* = 11.8 Hz, 1H), 6.92 – 6.84 (m, 3H), 3.96 (s, 3H), 3.93 (s, 3H), 1.34 – 1.30 (m, 2H), 1.07 (dd, *J* = 7.4, 4.3 Hz, 12H).

^{13}C NMR (126 MHz, $CDCl_3$): δ 194.33 (d, *J* = 3.78 Hz), 161.49, 159.46, 152.79, 148.29, 146.07(d, *J* = 10.08 Hz), 140.04, 132.53, 131.80, 131.12 (d, *J* = 2.52 Hz), 131.11, 130.18, 129.24 (d, *J* = 2.52 Hz), 129.23, 123.41 (d, *J* = 10.08 Hz), 119.92, 119.46, 118.45, 118.24, 115.24, 112.55, 102.38, 56.50, 56.38, 31.44 (d, *J* = 7.56 Hz), 19.89, 17.59, 17.44, 13.73.

HRMS: Calculated mass for $[M+Na]^+$ = 576.1744; observed mass: 576.1749

IR (cm^{-1}): 2925, 2219, 1689, 1603, 1444, 1241, 1174, 1136, 1026.

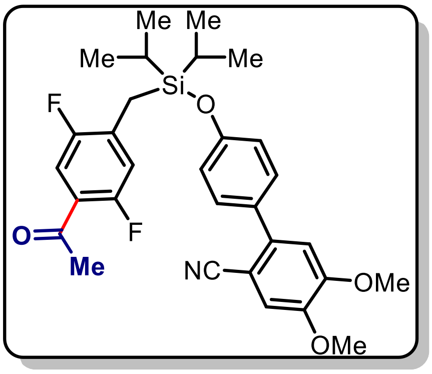

4'-((4-acetyl-2,5-difluorobenzyl)diisopropylsilyloxy)-4,5-dimethoxybiphenyl-2-carbonitrile (3f):

Para-ketonisation has been done following the general procedure A in 0.1 mmol scale.

R$_f$ value: 0.32 (20% ethyl acetate in pet ether)

Column material: 100-200 mesh silica

Eluent: pet ether:ethyl acetate (91: 9)

Yield: 67% (40 mg); (brsm 81%)

Physical appearance: Colorless viscous liquid

^{1}H NMR (500 MHz, $CDCl_3$): δ 7.55 – 7.47 (m, 1H), 7.40 (d, *J* = 7.9 Hz, 2H), 7.13 (s, 1H), 6.95 – 6.89 (m, 2H), 6.88 (d, *J* = 8.4 Hz, 2H), 3.96 (s, 3H), 3.93 (s, 3H), 2.59 (d, *J* = 4.7 Hz, 3H), 2.41 (s, 2H), 2.41 (s, 2H), 1.28 (d, *J* = 7.0 Hz, 2H), 1.10 – 1.04 (m, 12H).

^{13}C NMR (126 MHz, $CDCl_3$): δ 194.4 (d, *J* = 3.78 Hz), 159.21, 157.54, 157.20, 155.72, 152.82, 148.33, 140.06, 135.31 (d, *J* = 10.08 Hz), 135.15 (d, *J* = 10.08 Hz), 131.86, 130.20, 119.98, 119.44, 118.67 (d, *J* = 3.78 Hz), 118.45 (d, *J* = 3.78 Hz), 116.18 (d, *J* = 3.78 Hz), 115.96 (d, *J* = 3.78 Hz), 115.31, 112.60, 102.44, 56.52, 56.38, 31.40, 31.33, 17.48, 17.40, 13.50.

HRMS: Calculated mass for $[M+Na]^+$ = 560.2039; observed mass: 560.2039.

IR (cm^{-1}): 2930, 2218, 1689, 1600, 1463, 1264, 1176, 1137, 1027.

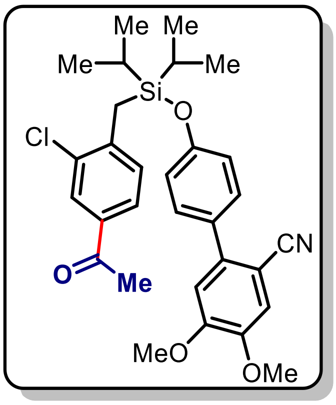

4'-((4-acetyl-2-chlorobenzyl)diisopropylsilyloxy)-4,5-dimethoxybiphenyl-2-carbonitrile (3g): *Para*-ketonisation has been done following the general procedure A in 0.1 mmol scale.

R_f value: 0.32 (20% ethyl acetate in pet ether)

Column material: 100-200 mesh silica

Eluent: pet ether:ethyl acetate (91: 9)

Yield: 73% (39 mg); (brsm 91%)

Physical appearance: Colorless viscous liquid

^{1}H NMR (500 MHz, $CDCl_3$): δ 7.91 (d, *J* = 1.8 Hz, 1H), 7.70 (dd, *J* = 8.1, 1.8 Hz, 1H), 7.37 (dd, *J* = 9.1, 2.5 Hz, 2H), 7.28 (d, *J* = 8.1 Hz, 1H), 7.13 (s, 1H), 6.88 (s, 1H), 6.84 (d, *J* = 8.6 Hz, 2H), 3.96 (s, 3H), 3.93 (s, 3H), 2.63 (s, 2H), 2.55 (s, 3H), 1.33 – 1.28 (m, 2H), 1.09 – 1.05 (m, 12H).

^{13}C NMR (126 MHz, $CDCl_3$): δ 196.84, 155.84, 152.78, 148.26, 143.71, 140.14, 135.41, 133.78, 131.61, 130.83, 130.10, 129.69, 126.65, 120.00, 119.50, 115.22, 112.53, 102.37, 56.50, 56.38, 26.69, 19.67, 17.62, 17.48, 13.71.

HRMS: Calculated mass for $[M+H]^+$ = 536.2018; observed mass: 536.2019.

IR (cm^{-1}): 2939, 2868, 2218, 1683, 1601, 1500, 1461, 1388, 1243, 1215, 1137, 1027.

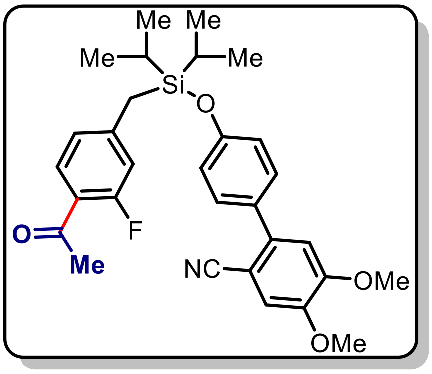

4'-((4-acetyl-3-fluorobenzyl)diisopropylsilyloxy)-4,5-dimethoxybiphenyl-2-carbonitrile (3h):

Para-ketonisation has been done following the general procedure A in 0.1 mmol scale.

R_f value: 0.32 (20% ethyl acetate in pet ether)

Column material: 100-200 mesh silica

Eluent: pet ether:ethyl acetate (91: 9)

Yield: 68% (35.3 mg); (brsm 94%)

Physical appearance: Colorless viscous liquid

^{1}H NMR (500 MHz, $CDCl_3$): δ 7.74 (t, *J* = 8.1 Hz, 1H), 7.40 (d, *J* = 8.6 Hz, 2H), 7.13 (s, 1H), 6.99 – 6.95 (m, 1H), 6.89 (d, *J* = 4.3 Hz, 1H), 6.89 – 6.84 (m, 3H), 3.96 (s, 3H), 3.93 (s, 3H), 2.60 (s, 1H), 2.44 (s, 2H), 1.22 (dd, *J* = 14.7, 7.3 Hz, 2H), 1.09 – 1.03 (m, 12H).

^{13}C NMR (126 MHz, $CDCl_3$): δ 195.64 (d, *J* = 3.78 Hz), 163.58, 161.55, 155.80, 152.78, 148.27, 148.20, 140.05, 133.72, 131.81, 130.68, 130.19, 125.23, 122.27 (d, *J* = 12.6 Hz), 122.21, 120.06, 119.45, 116.68 (d, *J* = 23.94 Hz), 115.24, 112.55, 102.38, 56.49, 56.36, 31.53, 31.47, 21.97, 17.60, 17.54, 13.22.

HRMS: Calculated mass for $[M+H]^+$ = 520.2314; observed mass: 520.2314.

IR (cm^{-1}): 2946, 2870, 2220, 1680, 1603, 1500, 1444, 1399, 1262, 1217, 1137, 1030, 881.

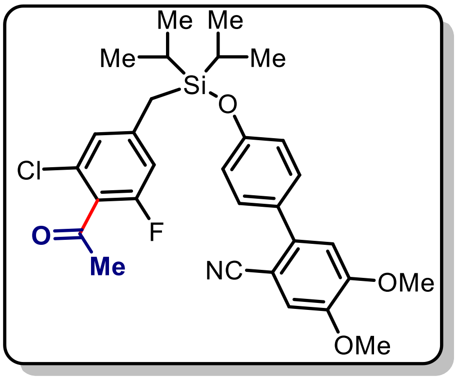

4'-((4-acetyl-3-chloro-5-fluorobenzyl)diisopropylsilyloxy)-4,5-dimethoxybiphenyl-2-carbo nitrile (3i):

Para-ketonisation has been done following the general procedure A in 0.05 mmol scale.

R_f value: 0.32 (20% ethyl acetate in pet ether)

Column material: 100-200 mesh silica

Eluent: pet ether:ethyl acetate (91: 9)

Yield: 67% (18.5 mg); (brsm 90%)

Physical appearance: Colorless viscous liquid

^{1}H NMR (500 MHz, $CDCl_3$): δ 7.43 – 7.40 (m, 2H), 7.14 (s, 1H), 6.98 (s, 1H), 6.90 (s, 1H), 6.89 – 6.87 (m, 2H), 6.82 – 6.78 (m, 1H), 3.96 (s, 3H), 3.93 (s, 3H), 2.43 (d, J = 1.1 Hz, 3H), 2.36 (s, 2H), 1.30 – 1.28 (m, 2H), 1.08 (dd, J = 7.4, 2.3 Hz, 12H).

^{13}C NMR (126 MHz, $CDCl_3$): δ 190.81, 159.90, 157.93, 155.78, 152.80, 151.99, 148.31, 142.47 (d, J = 10.08 Hz), 142.43, 140.03, 132.82, 131.89, 130.23, 125.97 (d, J = 2.52 Hz), 120.04, 115.28, 114.83 (d, J = 22.68 Hz), 112.57, 102.41, 56.51, 56.37, 32.14, 21.12, 17.64, 17.56, 13.22.

HRMS: Calculated mass for $[M+Na]^+$ = 576.1744; observed mass: 576.1747.

IR (cm^{-1}): 2925, 2218, 1690, 1605, 1462, 1262, 1174, 1136, 1028.

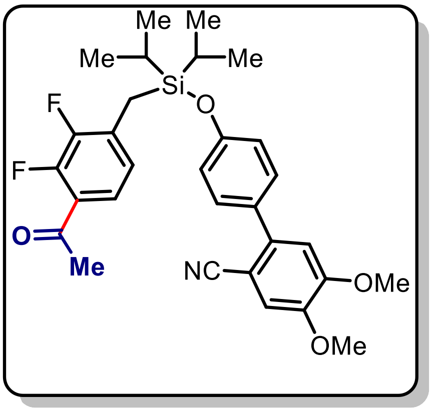

4'-((4-acetyl-2,3-difluorobenzyl)diisopropylsilyloxy)-4,5-dimethoxybiphenyl-2-carbonitrile (3j):

Para-ketonisation has been done following the general procedure A in 0.05 mmol scale.

R_f value: 0.32 (20% ethyl acetate in pet ether)

Column material: 100-200 mesh silica

Eluent: pet ether:ethyl acetate (91: 9)

Yield: 66% (17.7 mg); (brsm 88%)

Physical appearance: Colorless viscous liquid

^{1}H NMR (400 MHz, $CDCl_3$): δ 7.84 (d, J = 7.0 Hz, 1H), 7.40 (d, J = 8.6 Hz, 2H), 7.13 (s, 1H), 7.00 (d, J = 11.8 Hz, 1H), 6.88 (dd, J = 8.8, 6.3 Hz, 3H), 3.96 (s, 3H), 3.93 (s, 3H), 2.62 – 2.58 (m, 3H), 2.58 (s, 2H), 1.36 – 1.31 (m, 2H), 1.07 (dd, J = 7.4, 4.3 Hz, 12H).

^{13}C NMR (126 MHz, $CDCl_3$): δ 194.62, (d, J = 3.78 Hz), 155.72, 152.83, 148.34, 140.08, 134.97, 134.91 (d, J = 11.34 Hz),, 133.77, 131.83, 130.17, 125.49 (t, J = 3.78 Hz), 124.29, 124.01 , (d, J = 10.08 Hz), 119.99, 119.44, 115.32, 114.28, 112.59, 102.45, 56.52, 56.39, 31.40 (d, J = 7.56 Hz), 22.90, 17.49, 17.43, 13.51.

HRMS: Calculated mass for $[M+Na]^+$ = 560.2039; observed mass: 560.2039

IR (cm^{-1}): 2926, 2219, 1690, 1601, 1465, 1262, 1174, 1136, 1025.

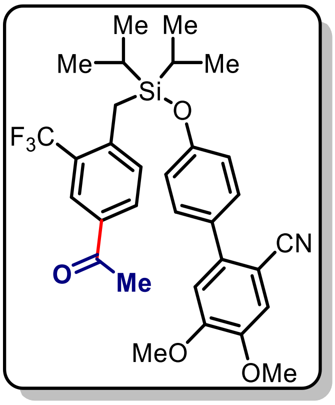

4'-((4-acetyl-2-(trifluoromethyl)benzyl)diisopropylsilyloxy)-4,5-dimethoxybiphenyl-2-carbonitrile (3k): *Para*-ketonisation has been done following the general procedure A in 0.1 mmol scale.

R_f value: 0.32 (20% ethyl acetate in pet ether);

Column material: 100-200 mesh silica;

Eluent: pet ether:ethyl acetate (91: 9)

Yield: 78% (44.4 mg); (brsm 89%).

Physical appearance: Colorless viscous liquid.

NMR:

^{1}H NMR (500 MHz, $CDCl_3$) δ 8.18 (s, 1H), 7.96 (d, *J* = 8.1 Hz, 1H), 7.46 (d, *J* = 8.2 Hz, 1H), 7.38 (d, *J* = 8.6 Hz, 2H), 7.13 (s, 1H), 7.13 (s, 1H), 6.88 (s, 1H), 6.86 (d, *J* = 8.6 Hz, 2H), 3.96 (s, 3H), 3.93 (s, 3H), 2.66 (s, 2H), 2.58 (d, *J* = 7.6 Hz, 3H), 1.31 (dd, *J* = 14.9, 7.5 Hz, 2H), 1.06 (d, *J* = 7.5 Hz, 6H), 1.00 – 0.96 (m, 6H).

^{13}C NMR (126 MHz, $CDCl_3$) δ 196.74, 155.79, 152.84, 148.35, 144.73, 140.09, 134.00, 132.08, 131.78, 131.15, 130.19, 128.52, 126.76, 126.71, 126.67, 126.62, 125.58, 123.40, 120.00, 119.45, 115.31, 112.59, 102.46, 56.52, 56.39, 26.64, 17.59, 17.36, 13.55.

HRMS: Calculated mass for $[M+Na]^+$ = 592.2101; observed mass: 592.2121

IR (cm^{-1}): 2928, 2870, 2218, 1689, 1601, 1500, 1341, 1262, 1138, 1016.

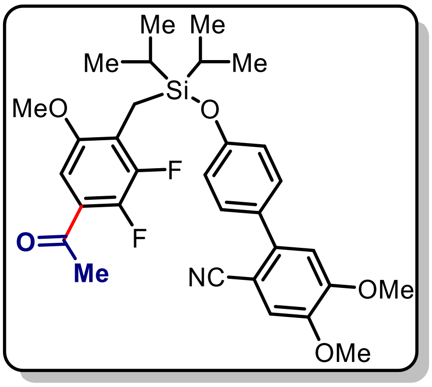

4'-(((4-acetyl-2,3-difluoro-6-methoxybenzyl)diisopropylsilyl)oxy)-4,5-dimethoxy-[1,1'-biphenyl]-2-carbonitrile (4a): *Para*-ketonisation has been done following the general procedure A in 0.05 mmol scale.

R_f value: 0.30 (20% ethyl acetate in pet ether)

Column material: 100-200 mesh silica

Eluent: pet ether:ethyl acetate (90: 10)

Yield: 61% (17.3 mg); (brsm 90%)

Physical appearance: Colorless viscous liquid

^{1}H NMR (500 MHz, $CDCl_3$): δ 7.36 – 7.34 (m, 2H), 7.12 (s, 1H), 7.01 (dd, *J* = 5.1, 1.7 Hz, 1H), 6.87 (s, 1H), 6.83 – 6.81 (m, 2H), 3.96 (s, 3H), 3.93 (s, 3H), 3.76 (s, 3H), 2.61 (d, *J* = 5.3 Hz, 3H), 2.43 (d, *J* = 2.5 Hz, 2H), 1.28 (d, *J* = 3.6 Hz, 2H), 1.08 (dd, *J* = 7.4, 0.7 Hz, 12H).

^{13}C NMR (126 MHz, $CDCl_3$): δ 194.57, 171.05, 167.07, 163.87, 159.81, 155.93, 152.73, 148.18, 140.13, 131.34, 129.92, 124.72, 124.59, 122.18, 122.08, 119.86, 119.54, 115.19, 112.49, 103.89, 102.31, 56.46, 56.38, 55.86, 17.41, 17.37, 13.96.

HRMS: Calculated mass for $[M+H]^+$ = 538.2220 ; observed mass: 538.2221.

IR (cm^{-1}): 2923, 2854, 2220, 1670, 1604, 1500, 1465, 1394, 1263, 1206, 914, 880.

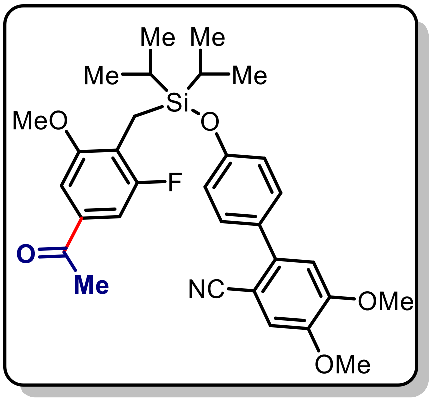

4'-(((4-acetyl-2-fluoro-6-methoxybenzyl)diisopropylsilyl)oxy)-4,5-dimethoxy-[1,1'-biphenyl] -2-carbonitrile (4b): *Para*-ketonisation has been done following the general procedure A in 0.05 mmol scale.

R_f value: 0.30 (20% ethyl acetate in pet ether)

Column material: 100-200 mesh silica

Eluent: pet ether:ethyl acetate (90: 10)

Yield: 71% (19.5 mg); (brsm 88%)

Physical appearance: Colorless viscous liquid

^{1}H NMR (500 MHz, $CDCl_3$): δ 7.34 – 7.32 (m, 2H), 7.23 (dd, *J* = 9.6, 1.5 Hz, 1H), 7.18 (s, 1H), 7.12 (s, 1H), 6.87 (s, 1H), 6.81 – 6.78 (m, 2H), 3.96 (s, 3H), 3.93 (s, 3H), 3.79 (s, 3H), 2.54 (s, 3H), 2.43 (d, *J* = 2.1 Hz, 2H), 1.29 – 1.26 (m, 2H), 1.07 (dd, *J* = 7.4, 3.4 Hz, 12H).

^{13}**C NMR** (126 MHz, $CDCl_3$): δ 192.26, 157.06 (d, *J* = 252 Hz), 152.75, 148.18, 140.30, 134.97, 131.18, 129.86, 119.90, 119.59, 115.18, 112.52, 108.93, 108.74, 104.82, 102.29, 56.50, 56.38, 55.85, 26.68, 17.45, 17.41, 13.95, 8.79.

HRMS: Calculated mass for $[M+H]^+$ = 550.2420; observed mass: 550.2420

IR (cm^{-1}): 2921, 2853, 2219, 1669, 1603, 1502, 1463, 1397, 1267, 1208, 917, 882.

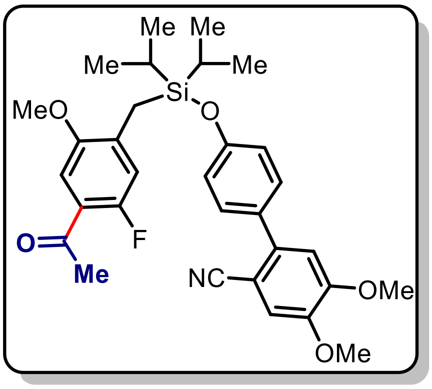

4'-(((4-acetyl-5-fluoro-2-methoxybenzyl)diisopropylsilyl)oxy)-4,5-dimethoxy-[1,1'-biphenyl]-2-carbonitrile (4c): *Para*-ketonisation has been done following the general procedure A in 0.05 mmol scale.

R_f value: 0.30 (20% ethyl acetate in pet ether)

Column material: 100-200 mesh silica

Eluent: pet ether:ethyl acetate (90: 10)

Yield: 61% (16.7 mg); (brsm 96%)

Physical appearance: Colorless viscous liquid

^{1}**H NMR** (400 MHz, $CDCl_3$): δ 7.41 – 7.36 (m, 2H), 7.24 (d, *J* = 6.1 Hz, 1H), 7.13 (s, 1H), 6.90 (s, 1H), 6.88 – 6.83 (m, 3H), 3.97 (s, 3H), 3.93 (s, 3H), 3.78 (s, 3H), 2.60 (s, 3H), 2.44 (s, 2H), 1.24 – 1.20 (m, 2H), 1.05 (d, *J* = 7.2 Hz, 12H).

^{13}**C NMR** (101 MHz, $CDCl_3$): δ 195.43, 157.17 (d, *J* = 248 Hz), 156.04, 153.04, 152.77, 148.23, 140.18, 137.29 (d, *J* = 9 Hz), 131.50, 130.05, 121.89 (d, *J* = 14 Hz), 120.05, 119.52, 118.04, 117.78, 115.20, 112.55, 109.72 (d, *J* = 2 Hz), 102.37, 56.51, 56.38, 55.73, 17.51, 17.46, 16.02, 13.60.

HRMS: Calculated mass for $[M+H]^+$ = 550.2420; observed mass: 550.2421

IR (cm^{-1}): 2922, 2853, 2220, 1679, 1605, 1502, 1463, 1397, 1267, 1207, 915.

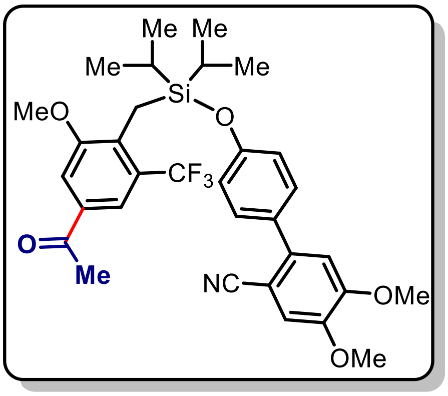

4'-(((4-acetyl-2-methoxy-6-(trifluoromethyl)benzyl)diisopropylsilyl)oxy)-4,5-dimethoxy-[1,1'-biphenyl]-2-carbonitrile (4d): *Para*-ketonisation has been done following the general procedure A in 0.05 mmol scale.

R_f value: 0.30 (20% ethyl acetate in pet ether)

Column material: 100-200 mesh silica

Eluent: pet ether:ethyl acetate (90: 10)

Yield: 73% (21.9 mg); (brsm 91%)

Physical appearance: Colorless viscous liquid

^{1}H NMR (400 MHz, $CDCl_3$): δ 7.76 (s, 1H), 7.47 (s, 1H), 7.33 – 7.29 (m, 2H), 7.12 (s, 1H), 6.86 (s, 1H), 6.79 – 6.75 (m, 2H), 3.96 (s, 3H), 3.93 (s, 3H), 3.79 (s, 3H), 2.66 (s, 2H), 2.57 (s, 3H), 1.29 (d, J = 7.6 Hz, 2H), 1.06 (d, J = 7.4 Hz, 6H), 1.02 (d, J = 7.4 Hz, 6H).

^{13}C NMR (101 MHz, $CDCl_3$): δ 196.88, 157.98, 156.11, 152.75, 148.22, 140.28, 134.94, 134.65, 131.18, 129.86, 126.97 (q, J = 266 Hz), 119.92, 119.58, 119.19, 119.13, 115.21, 112.53, 111.11, 102.34, 56.52, 56.41, 55.71, 26.63, 17.62, 14.19.

HRMS: Calculated mass for $[M+H]^+$ = 600.2388; observed mass: 600.2387.

IR (cm^{-1}): 2946, 2868, 2221, 1683, 1601, 1503, 1468, 1344, 1214, 1137, 915, 830.

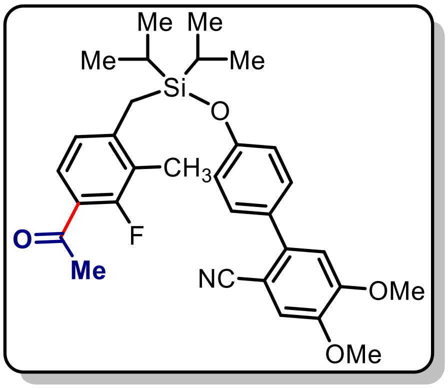

4'-(((4-acetyl-3-fluoro-2-methylbenzyl)diisopropylsilyl)oxy)-4,5-dimethoxy-[1,1'-biphenyl]-2-carbonitrile (4e):

Para-ketonisation has been done following the general procedure A in 0.1 mmol scale.

R$_f$ value: 0.32 (20% ethyl acetate in pet ether)

Column material: 100-200 mesh silica

Eluent: pet ether:ethyl acetate (91: 9)

Yield: 69% (36.8 mg); (brsm 97%)

Physical appearance: Colorless viscous liquid

^{1}H NMR (500 MHz, $CDCl_3$): δ 7.59 (t, *J* = 7.9 Hz, 1H), 7.34 (d, *J* = 8.6 Hz, 2H), 7.12 (s, 1H), 7.00 (d, *J* = 8.2 Hz, 1H), 6.87 (s, 1H), 6.73 (d, *J* = 8.6 Hz, 2H), 3.96 (s, 3H), 3.92 (s, 3H), 2.59 (d, *J* = 5.2 Hz, 3H), 2.45 (s, 2H), 2.24 (d, *J* = 2.5 Hz, 3H), 1.33 – 1.29 (m, 2H), 1.09 (d, *J* = 7.5 Hz, 6H), 1.04 (d, *J* = 7.4 Hz, 6H).

^{13}C NMR (126 MHz, $CDCl_3$): δ 196.44 (d, *J* = 3.8 Hz), 161.27 (d, *J* = 252 Hz), 155.67, 152.78, 148.23, 146.88 (d, *J* = 5 Hz), 140.06, 131.58, 130.06, 127.23 (d, *J* = 3.8 Hz), 125.10 (d, *J* = 3.8 Hz), 123.74 (d, *J* = 17.6 Hz), 119.80, 119.47, 115.21, 112.50, 102.35, 56.45, 56.38, 27.17, 17.71, 17.52, 13.55, 11.64 (d, *J* = 7.6 Hz).

HRMS: Calculated mass for $[M+H]^+$ = 534.2470; observed mass: 534.2470.

IR (cm^{-1}): 2943, 2867, 2229, 1680, 1601, 1502, 1463, 1353, 1217, 1137, 911, 838.

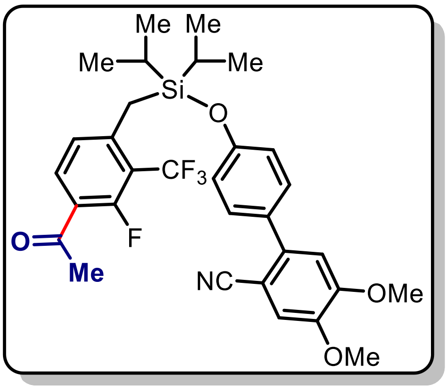

4'-(((4-acetyl-3-fluoro-2-(trifluoromethyl)benzyl)diisopropylsilyl)oxy)-4,5-dimethoxy-[1,1'-biphenyl]-2-carbonitrile (4f): *Para*-ketonisation has been done following the general procedure A in 0.05 mmol scale.

R$_f$ value: 0.32 (20% ethyl acetate in pet ether)

Column material: 100-200 mesh silica

Eluent: pet ether:ethyl acetate (91: 9)

Yield: 62% (18.2 mg); (brsm 90%)

Physical appearance: Colorless viscous liquid

^{1}H NMR (500 MHz, $CDCl_3$): δ 7.38 (d, *J* = 8.5 Hz, 2H), 7.30 (t, *J* = 7.9 Hz, 1H), 7.13 (s, 1H), 7.07 (d, *J* = 8.4 Hz, 1H), 6.88 (s, 1H), 6.83 (d, *J* = 8.5 Hz, 2H), 3.96 (s, 3H), 3.93 (s, 3H), 2.66 (d, *J* = 1.7 Hz, 2H), 2.52 (s, 3H), 1.34 – 1.31 (m, 2H), 1.08 (d, *J* = 7.5 Hz, 6H), 1.01 (d, *J* = 7.4 Hz, 6H).

^{13}C NMR (126 MHz, $CDCl_3$): δ 194.72, 155.65, 152.80, 152.74, 148.32, 147.45, 140.07, 139.56, 132.92, 132.89, 131.82, 130.18, 130.11, 127.74, 119.84, 119.48, 119.41, 115.28, 115.15, 114.29, 112.56, 112.52, 102.44, 56.51, 56.39, 17.62, 17.40, 14.32, 13.63.

HRMS: Calculated mass for $[M+H]^+$ = 588.2188 ; observed mass: 580.2188.

IR (cm^{-1}): 2958, 2870, 2222, 1689, 1603, 1502, 1244, 1260, 1123, 1023.

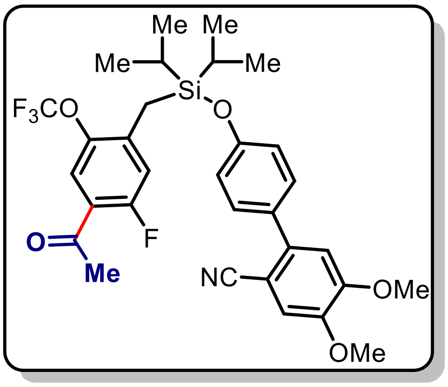

4'-(((4-acetyl-5-fluoro-2-(trifluoromethoxy)benzyl)diisopropylsilyl)oxy)-4,5-dimethoxy-[1,1'-biphenyl]-2-carbonitrile (4g): *Para*-ketonisation has been done following the general procedure A in 0.05 mmol scale.

R_f value: 0.31 (20% ethyl acetate in pet ether)

Column material: 100-200 mesh silica

Eluent: pet ether:ethyl acetate (91: 9)

Yield: 74% (22.3 mg); (brsm 95%)

Physical appearance: Colorless viscous liquid

^{1}H NMR (400 MHz, $CDCl_3$): δ 7.73 (dd, *J* = 6.3, 1.5 Hz, 1H), 7.42 – 7.37 (m, 2H), 7.13 (s, 1H), 7.01 (d, *J* = 11.5 Hz, 1H), 6.89 (d, *J* = 4.2 Hz, 1H), 6.87 – 6.83 (m, 2H), 3.96 (s, 3H), 3.93 (s, 3H), 2.61 (d, *J* = 5.1 Hz, 3H), 2.46 (s, 2H), 1.33 – 1.28 (m, 2H), 1.08 – 1.05 (m, 11H).

^{13}C NMR (101 MHz, $CDCl_3$): δ 194.12 (d, *J* = 4 Hz), 159.86 (d, *J* = 256 Hz), 155.62, 152.78, 148.29, 143.29, 141.16 (d, *J* = 10 Hz), 140.00, 131.86, 130.21, 123.07 (d, *J* = 15.15 Hz), 122.11, 119.86, 119.45, 118.91 (d, *J* = 26.26 Hz), 115.23, 112.53, 102.38, 56.50, 56.37, 17.44, 17.33, 16.01, 13.52.

HRMS: Calculated mass for $[M+H]^+$ = 586.2231; observed mass: 586.2230.

IR (cm^{-1}): 2930, 2867, 2220, 1686, 1603, 1501, 1468,1444,1343, 1263, 1215, 918.

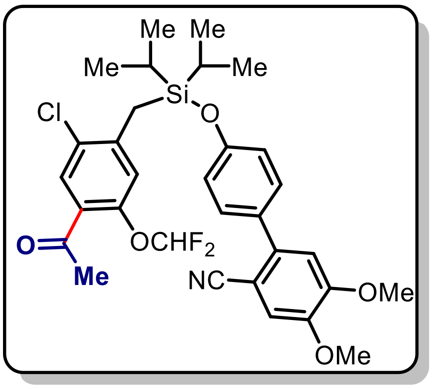

4'-(((4-acetyl-2-chloro-5-(difluoromethoxy)benzyl)diisopropylsilyl)oxy)-4,5-dimethoxy-[1,1'-biphenyl]-2-carbonitrile (4h): *Para*-ketonisation has been done following the general procedure A in 0.05 mmol scale.

R_f value: 0.32 (20% ethyl acetate in pet ether)

Column material: 100-200 mesh silica

Eluent: pet ether:ethyl acetate (91: 9)

Yield: 71% (21.3 mg); (brsm 93%)

Physical appearance: Colorless viscous liquid

^{1}H NMR (500 MHz, $CDCl_3$): δ 7.77 (s, 1H), 7.39 (d, *J* = 8.6 Hz, 2H), 7.13 (s, 1H), 7.02 (s, 1H), 6.89 (d, *J* = 4.9 Hz, 1H), 6.85 (d, *J* = 8.6 Hz, 2H), 6.53 (s, 1H), 6.38 (s, 1H), 6.24 (s, 1H), 3.96 (s, 3H), 3.93 (s, 3H), 2.60 (s, 2H), 2.57 (s, 2H), 1.34 – 1.31 (m, 2H), 1.08 (dd, *J* = 10.2, 7.5 Hz, 11H).

^{13}C NMR (126 MHz, $CDCl_3$): δ 196.47, 155.65, 152.82, 148.34, 148.14, 144.58, 139.96, 131.79, 131.54, 130.46, 130.21, 128.86, 121.75, 119.72, 119.46, 118.25, 116.17, 115.24, 112.55, 102.47, 102.38, 56.46, 56.38, 19.84, 17.60, 17.45, 13.76.

HRMS: Calculated mass for $[M+H]^+$ = 602.1936; observed mass: 602.1938

IR (cm^{-1}): 2920, 2853, 2222, 1689, 1603, 1504, 1463, 1384, 1267, 1217, 1176, 919.

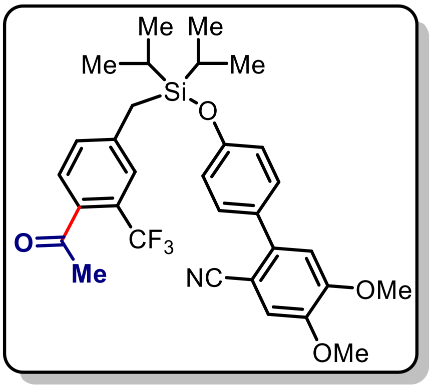

4'-(((4-acetyl-3-(trifluoromethyl)benzyl)diisopropylsilyl)oxy)-4,5-dimethoxy-[1,1'-biphenyl]-2-carbonitrile (4i):

Para-ketonisation has been done following the general procedure A in 0.05 mmol scale.

R_f value: 0.32 (20% ethyl acetate in pet ether)

Column material: 100-200 mesh silica

Eluent: pet ether:ethyl acetate (91: 9)

Yield: 68% (19.3 mg); (brsm 81%)

Physical appearance: Colorless viscous liquid

^{1}H NMR (500 MHz, $CDCl_3$): δ 7.90 (d, *J* = 14.9 Hz, 2H), 7.56 (s, 1H), 7.42 – 7.37 (m, 2H), 7.13 (d, *J* = 5.5 Hz, 1H), 6.90 (s, 1H), 6.88 – 6.83 (m, 2H), 3.96 (d, *J* = 3.3 Hz, 3H), 3.93 (s, 3H), 2.55 (s, 3H), 2.52 (d, *J* = 4.4 Hz, 2H), 1.29 – 1.25 (m, 2H), 1.07 (dd, *J* = 7.4, 5.7 Hz, 13H).

^{13}**C NMR** (126 MHz, $CDCl_3$): δ 197.06, 155.75, 152.81, 148.30, 141.18, 140.02, 137.72, 131.96, 131.80, 130.25, 129.94, 128.77, 127.05, 125.09, 123.28, 121.64, 121.61, 121.58, 121.55, 119.87, 119.76, 119.45, 115.27, 112.59, 102.36, 77.48, 56.51, 56.37, 26.87, 21.21, 17.64, 17.55, 13.28.

HRMS: Calculated mass for $[M+Na]^+$ = 592.2101; observed mass: 592.2120.

IR (cm^{-1}): 2949, 2873, 2220, 1687, 1605, 1501, 1467, 1341, 1268, 1176, 1036.

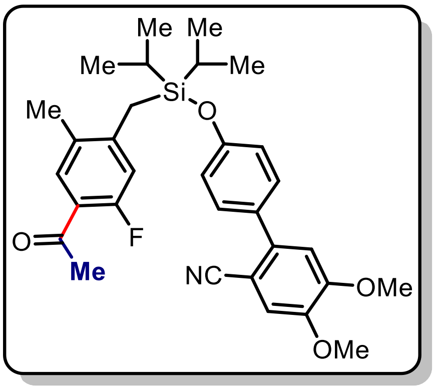

4'-(((4-acetyl-5-fluoro-2-methylbenzyl)diisopropylsilyl)oxy)-4,5-dimethoxy-[1,1'-biphenyl]-2-carbonitrile (4j): *Para*-ketonisation has been done following the general procedure A in 0.05 mmol scale.

R_f value: 0.32 (20% ethyl acetate in pet ether)

Column material: 100-200 mesh silica

Eluent: pet ether:ethyl acetate (91: 9)

Yield: 70% (18.7 mg); (brsm 87%)

Physical appearance: Colorless viscous liquid

^{1}**H NMR** (400 MHz, $CDCl_3$): δ 7.61 (d, *J* = 7.9 Hz, 1H), 7.39 – 7.31 (m, 2H), 7.12 (s, 1H), 6.90 (d, *J* = 12.4 Hz, 1H), 6.87 (s, 1H), 6.78 – 6.73 (m, 2H), 3.95 (s, 3H), 3.92 (s, 3H), 2.59 (d, *J* = 4.8 Hz, 3H), 2.39 (s, 2H), 2.28 (s, 3H), 1.34 – 1.27 (m, 2H), 1.12 – 1.06 (m, 6H), 1.06 – 1.01 (m, 6H).

^{13}**C NMR** (101 MHz, $CDCl_3$): δ 195.82 (d, *J* = 4.04 Hz), 160.80 (d, *J* = 253.5 Hz), 155.61, 152.75, 148.24, 146.88, 146.79, 140.04, 131.95, 131.93, 131.81, 131.78, 131.68, 130.08, 122.01, 121.88, 119.81, 119.42, 116.92, 116.68, 115.21, 112.53, 102.34, 56.47, 56.35, 31.56, 31.48, 19.71, 19.37, 17.68, 17.48, 13.56.

HRMS: Calculated mass for $[M+H]^+$ = 534.2470; observed mass: 534.2470.

IR (cm^{-1}): 2945, 2868, 2220, 1679, 1603, 1500, 1444, 1399, 1262, 1217, 1137, 1030, 881.

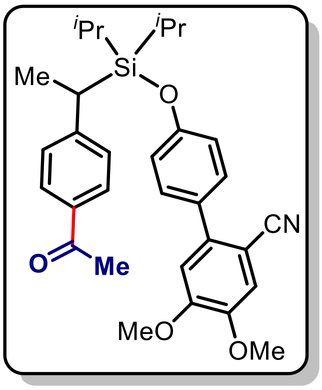

4'-(((1-(4-acetylphenyl)ethyl)diisopropylsilyl)oxy)-4,5-dimethoxy-[1,1'-biphenyl]-2-carbonitrile (4k): *Para*-ketonisation has been done following the general procedure A in 0.1 mmol scale.

R_f value: 0.32 (20% ethyl acetate in pet ether)

Column material: 100-200 mesh silica

Eluent: pet ether:ethyl acetate (91: 9)

Yield: 70% (36 mg); (brsm 89%)

Physical appearance: Colorless viscous liquid

^{1}H NMR (500 MHz, $CDCl_3$): δ 7.86 (d, *J* = 8.2 Hz, 2H), 7.38 (d, *J* = 8.4 Hz, 2H), 7.31 (d, *J* = 8.2 Hz, 2H), 7.13 (s, 1H), 6.89 (d, *J* = 6.5 Hz, 1H), 6.86 (d, *J* = 8.5 Hz, 2H), 3.96 (s, 3H), 3.93 (s, 3H), 2.72 (q, *J* = 7.4 Hz, 1H), 2.57 (s, 3H), 1.56 (d, *J* = 7.5 Hz, 3H), 1.34 (ddd, *J* = 23.5, 15.6, 8.1 Hz, 2H), 1.08 (d, *J* = 7.5 Hz, 6H), 1.01 (d, *J* = 7.4 Hz, 3H), 0.96 (d, *J* = 7.4 Hz, 3H).

^{13}C NMR (126 MHz, $CDCl_3$): δ 198.11, 156.16, 152.76, 151.47, 148.22, 140.17, 134.37, 131.48, 130.09, 128.63, 128.33, 120.02, 119.51, 115.21, 112.53, 102.35, 56.49, 56.37, 28.38, 26.72, 18.16, 18.06, 17.86, 17.78, 16.02, 13.23, 13.14.

HRMS: Calculated mass for $[M+H]^+$: 516.2565; observed mass: 516.2565.

IR (cm^{-1}): 2943, 2863, 2217, 1679, 1600, 1500, 1465, 1443, 1355, 1262, 1218, 1138, 1027.

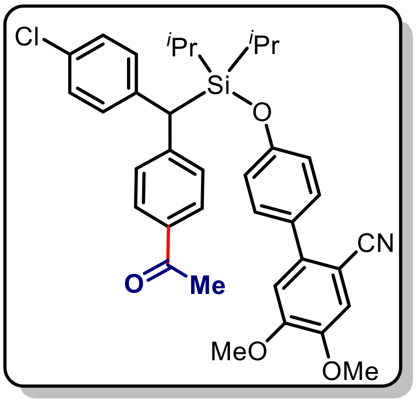

4'-((((4-acetylphenyl)(4-chlorophenyl)methyl)diisopropylsilyl)oxy)-4,5-dimethoxy-[1,1'-biphenyl]-2-carbonitrile (4l): *Para*-ketonisation has been done following the general procedure A in 0.05 mmol scale.

R_f value: 0.34 (20% ethyl acetate in pet ether)

Column material: 100-200 mesh silica

Eluent: pet ether:ethyl acetate (92: 8)

Yield: 75% (22.9 mg); (brsm 83%)

Physical appearance: Colorless viscous liquid

^{1}H NMR (500 MHz, $CDCl_3$): δ 7.88 (d, *J* = 8.3 Hz, 2H), 7.54 (d, *J* = 8.3 Hz, 2H), 7.43 (d, *J* = 8.4 Hz, 2H), 7.39 (d, *J* = 8.5 Hz, 2H), 7.27 (d, *J* = 6.5 Hz, 2H), 7.14 (s, 1H), 6.89 (s, 1H), 6.86 (d, *J* = 8.6 Hz, 2H), 3.97 (s, 3H), 3.93 (s, 3H), 3.84 (s, 1H), 2.57 (s, 2H), 1.31 – 1.27 (m, 2H), 0.96 (d, *J* = 7.5 Hz, 3H), 0.94 – 0.90 (m, 9H).

^{13}C NMR (126 MHz, $CDCl_3$): δ 197.98, 155.68, 152.79, 148.30, 147.79, 140.02, 139.80, 135.06, 132.16, 131.90, 131.03, 130.23, 129.51, 128.99, 128.96, 119.91, 119.48, 115.21, 112.52, 102.40, 56.51, 56.39, 42.54, 26.76, 18.13, 18.08, 17.84, 17.75, 13.89, 13.80.

HRMS: Calculated mass for $[C_{36}H_{38}ClNO_4Si]H^+$: 612.2331; observed mass: 612.2330.

IR (cm^{-1}): 2932, 2870, 2218, 1679, 1600, 1500, 1262 1217, 1137, 917.

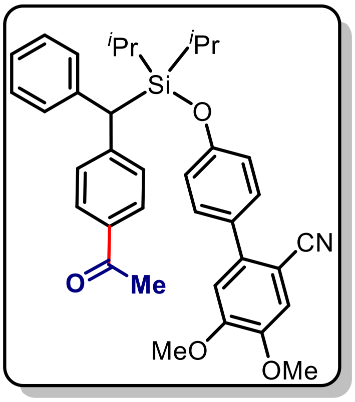

4'-((((4-acetylphenyl)(phenyl)methyl)diisopropylsilyl)oxy)-4,5-dimethoxy-[1,1'-biphenyl]-2-carbonitrile (4m): *Para*-ketonisation has been done following the general procedure A in 0.05 mmol scale.

R_f value: 0.34 (20% ethyl acetate in pet ether)

Column material: 100-200 mesh silica

Eluent: pet ether:ethyl acetate (92: 8)

Yield: 81% (23.4 mg); (brsm 97%)

Physical appearance: Colorless viscous liquid

^{1}H NMR (500 MHz, $CDCl_3$): δ 7.87 (d, *J* = 8.4 Hz, 2H), 7.58 (d, *J* = 8.4 Hz, 2H), 7.49 (d, *J* = 7.3 Hz, 2H), 7.42 – 7.37 (m, 2H), 7.32 – 7.28 (m, 2H), 7.19 (t, *J* = 7.3 Hz, 1H), 7.14 (s, 1H), 7.14 (s, 1H), 6.90 (s, 1H), 6.88 – 6.84 (m, 2H), 3.96 (s, 3H), 3.93 (s, 3H), 3.88 (s, 1H), 2.56 (s, 2H), 1.32 – 1.27 (m, 2H), 0.92 (ddd, *J* = 13.9, 9.7, 5.3 Hz, 12H).

^{13}C NMR (126 MHz, $CDCl_3$): δ 198.07, 155.86, 152.78, 148.35, 148.27, 141.07, 140.11, 134.86, 131.71, 130.14, 129.78, 129.58, 128.88, 128.83, 126.27, 119.98, 119.48, 115.23, 112.54, 102.38, 56.49, 56.37, 43.32, 26.73, 18.09, 18.07, 17.81, 17.78, 13.86.

HRMS: Calculated mass for $[M+H]^+$ = 578.2721; observed mass: 578.2720

IR (cm^{-1}): 2950, 2870, 2220, 1679, 1601, 1500, 1442, 1354, 1263, 1241, 1215, 1136, 1027, 915.

Characterization of C_{aryl}-C(O) Product:

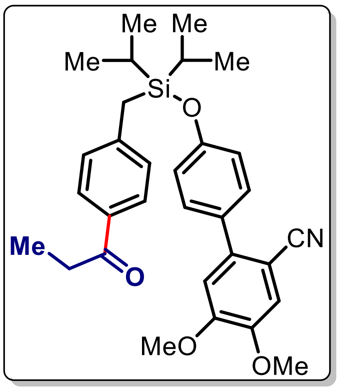

4'-((diisopropyl(4-propionylbenzyl)silyl)oxy)-4,5-dimethoxy-[1,1'-biphenyl]-2-carbonitrile (1q): *Para*-ketonisation has been done following the general procedure A in 0.1 mmol scale.

R_f value: 0.30 (20% ethyl acetate in pet ether)

Column material: 100-200 mesh silica

Eluent: pet ether:ethyl acetate (90: 10)

Yield: 82% (42.2 mg); (brsm 93%)

Physical appearance: Colorless viscous liquid

^{1}H NMR (500 MHz, $CDCl_3$): δ 7.83 (d, *J* = 8.0 Hz, 2H), 7.38 (d, *J* = 8.4 Hz, 2H), 7.20 (d, *J* = 8.0 Hz, 2H), 7.13 (s, 1H), 6.89 (s, 1H), 6.86 (d, *J* = 8.3 Hz, 2H), 3.96 (s, 3H), 3.93 (s, 3H), 2.96 (q, *J* = 7.2 Hz, 2H), 2.47 (s, 2H), 1.20 (t, *J* = 7.3 Hz, 4H), 1.05 (d, *J* = 7.1 Hz, 12H).

^{13}C NMR (126 MHz, $CDCl_3$): δ 200.73, 156.00, 152.81, 148.25, 145.14, 140.17, 133.78, 131.63, 130.13, 129.03, 128.42, 120.14, 119.57, 115.23, 112.55, 102.38, 56.50, 56.39, 31.77, 22.00, 17.63, 17.58, 13.24, 8.56.

HRMS: **HRMS**: Calculated mass for $[M+H]^+$ =516.2565; observed mass: 516.2560

IR (cm^{-1}): 2926, 2853, 2219, 1681, 1601, 1500, 1461, 1351, 1241, 1217, 1137, 1027.

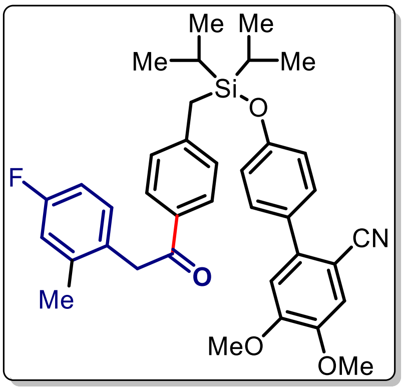

4'-(((4-(2-(4-fluoro-2-methylphenyl)acetyl)benzyl)diisopropylsilyl)oxy)-4,5-dimethoxy-[1,1'-biphenyl]-2-carbonitrile (1r):

Para- ketonisation has been done following the general procedure A in 0.05 mmol scale.

R_f value: 0.33 (20% ethyl acetate in pet ether)

Column material: 100-200 mesh silica

Eluent: pet ether:ethyl acetate (91: 9)

Yield: 58% (17.7 mg); (brsm 91%)

Physical appearance: Colorless viscous liquid

^{1}H NMR (400 MHz, $CDCl_3$): δ 7.88 (d, J = 8.4 Hz, 2H), 7.38 (d, J = 8.7 Hz, 2H), 7.22 (s, 1H), 7.13 (s, 2H), 6.89 (s, 1H), 6.85 (d, J = 9 Hz, 1H), 4.23 (s, 2H), 3.96 (s, 3H), 3.93 (s, 3H), 2.48 (s, 2H), 2.22 (s, 3H), 1.22 (d, J = 7.0 Hz, 2H), 1.07 (dd, J = 7.4, 1.2 Hz, 12H).

^{13}C NMR (101 MHz, $CDCl_3$): δ 196.99, 162.0 (d, J = 245.4 Hz), 155.97, 152.79, 148.27, 145.75, 140.15, 133.55, 131.86 (d, J = 8.1 Hz), 131.67, 130.26, 129.26, 128.71, 120.12, 117.16 (d, J = 21.2 Hz), 115.20, 112.83 (d, J = 21.2 Hz), 112.53, 102.38, 56.50, 56.39, 42.63, 22.08, 17.65, 17.59, 13.21.

HRMS: Calculated mass for $[M+H]^+$ = 610.2783; observed mass: 610.2785

IR (cm^{-1}): 3021, 2928, 2223, 1718, 1685, 1604, 1505, 1217.

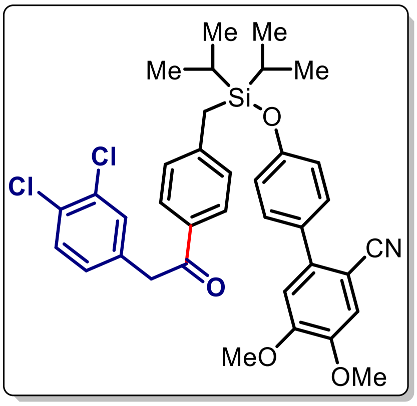

4'-(((4-(2-(3,4-dichlorophenyl)acetyl)benzyl)diisopropylsilyl)oxy)-4,5-dimethoxy-[1,1'-biphenyl]-2-carbonitrile (1s): *Para*-ketonisation has been done following the general procedure A in 0.05 mmol scale.

R_f value: 0.34 (20% ethyl acetate in pet ether)

Column material: 100-200 mesh silica

Eluent: pet ether:ethyl acetate (91: 9)

Yield: 58% (18.7 mg); (brsm 93%)

Physical appearance: Colorless viscous liquid.

^{1}H NMR (500 MHz, $CDCl_3$): δ 7.85 (d, *J* = 8.3 Hz, 2H), 7.39 – 7.35 (m, 3H), 7.34 (d, *J* = 1.9 Hz, 1H), 7.20 (d, *J* = 8.3 Hz, 2H), 7.14 (s, 1H), 7.08 (dd, *J* = 8.2, 2.1 Hz, 1H), 6.89 (s, 1H),

6.86 – 6.82 (m, 2H), 4.20 (s, 2H), 3.95 (d, *J* = 6.9 Hz, 3H), 3.93 (s, 3H), 2.48 (s, 2H), 1.25 – 1.20 (m, 2H), 1.06 (dd, *J* = 7.4, 1.7 Hz, 12H).

^{13}C NMR (126 MHz, $CDCl_3$): δ 196.25, 155.93, 152.78, 148.25, 146.10, 140.13, 135.15, 133.03, 131.78, 130.56, 130.14, 129.34, 129.32, 128.89, 120.10, 115.17, 112.52, 102.35, 56.49, 56.38, 44.21, 22.14, 17.63, 17.57, 13.20.

HRMS: Calculated mass for $[M+H]^+$ = 646.1942; observed mass: 646.1942

IR (cm^{-1}): 3023, 2845, 2868, 2220, 1681, 1603, 1502, 1265, 1215, 1136, 915.

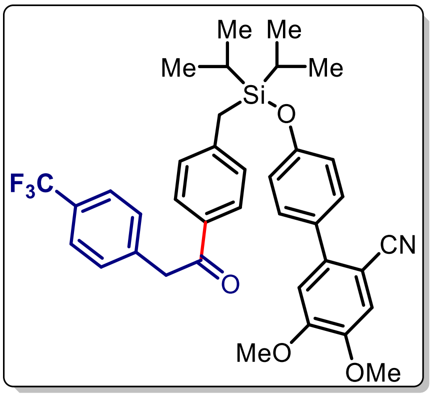

4'-((diisopropyl(4-(2-(4-(trifluoromethyl)phenyl)acetyl)benzyl)silyl)oxy)-4,5-dimethoxy-[1,1'-biphenyl]-2-carbonitrile (1t): *Para*-ketonisation has been done following the general procedure A in 0.05 mmol scale.

R_f value: 0.34 (20% ethyl acetate in pet ether)

Column material: 100-200 mesh silica

Eluent: pet ether:ethyl acetate (91: 9)

Yield: 77% (24.8 mg); (brsm 94%)

Physical appearance: Colorless viscous liquid

^{1}H NMR (500 MHz, $CDCl_3$): δ 7.87 (d, *J* = 8.3 Hz, 2H), 7.56 (d, *J* = 8.1 Hz, 2H), 7.40 – 7.34 (m, 4H), 7.20 (d, *J* = 8.3 Hz, 2H), 7.14 (s, 1H), 6.89 (s, 1H), 6.86 – 6.83 (m, 2H), 4.31 (s, 2H), 3.96 (s, 3H), 3.93 (s, 3H), 2.47 (d, *J* = 6.7 Hz, 2H), 1.24 – 1.20 (m, 2H), 1.08 – 1.04 (m, 12H).

^{13}C NMR (126 MHz, $CDCl_3$): δ 196.52, 155.94, 152.78, 148.26, 146.08, 140.14, 139.10, 134.20, 133.14, 131.68, 130.71, 130.21, 130.14, 129.32, 128.93, 125.68, 125.65, 125.62, 125.59, 120.10, 115.17, 112.51, 102.34, 56.49, 56.37, 45.03, 22.12, 17.63, 17.57, 13.20.

HRMS: Calculated mass for $[M+H]^+$ = 646.2595 ; observed mass: 646.2597

IR (cm^{-1}): 2928, 2864, 2219, 1683, 1601, 1502, 1323, 1264, 1164, 1123, 913.

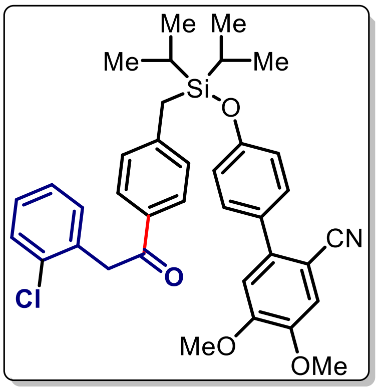

4'-(((4-(2-(2-chlorophenyl)acetyl)benzyl)diisopropylsilyl)oxy)-4,5-dimethoxy-[1,1'-biphenyl]-2-carbonitrile (1u): *Para*-ketonisation has been done following the general procedure A in 0.05 mmol scale.

R_f value: 0.33 (20% ethyl acetate in pet ether)

Column material: 100-200 mesh silica

Eluent: pet ether:ethyl acetate (91: 9)

Yield: 68% (20.8 mg); (brsm 95%)

Physical appearance: Colorless viscous liquid

^{1}H NMR (500 MHz, $CDCl_3$): δ 7.90 (d, J = 8.1 Hz, 2H), 7.38 (d, J = 8.4 Hz, 3H), 7.24 – 7.18 (m, 5H), 7.13 (s, 1H), 6.89 (s, 1H), 6.84 (d, J = 8.4 Hz, 2H), 4.40 (s, 2H), 3.95 (d, J = 6.5 Hz, 3H), 3.93 (s, 3H), 2.48 (s, 2H), 1.21 (dd, J = 12.2, 5.1 Hz, 2H), 1.09 – 1.04 (m, 12H).

^{13}C NMR (126 MHz, $CDCl_3$): δ 196.13, 155.96, 152.75, 148.22, 145.78, 140.15, 134.70, 133.62, 133.40, 131.91, 131.63, 130.12, 129.64, 129.23, 128.82, 128.63, 127.04, 120.10, 115.17, 112.49, 102.34, 56.49, 56.38, 43.23, 22.09, 17.65, 17.59, 13.18.

HRMS: Calculated mass for $[M+H]^+$ = 612.2331; observed mass: 612.2331.

IR (cm^{-1}): 2933, 2868, 2218, 1687, 1601, 1500, 1463, 1444, 1241, 1217, 1027, 913.

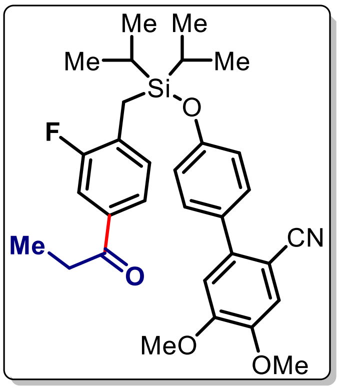

4'-(((2-fluoro-4-propionylbenzyl)diisopropylsilyl)oxy)-4,5-dimethoxy-[1,1'-biphenyl]-2-carbonitrile (1v): *Para*- ketonisation has been done following the general procedure A in 0.05 mmol scale.

R_f value: 0.33 (20% ethyl acetate in pet ether)

Column material: 100-200 mesh silica

Eluent: pet ether:ethyl acetate (91: 9)

Yield: 67% (17.9 mg); (brsm 92%)

Physical appearance: Colorless viscous liquid

^{1}H NMR (500 MHz, $CDCl_3$): δ 7.61 (dd, *J* = 7.9, 1.7 Hz, 1H), 7.59 (d, *J* = 10.6 Hz, 1H), 7.38 (d, *J* = 8.6 Hz, 3H), 7.21 (t, *J* = 7.8 Hz, 1H), 7.13 (s, 1H), 6.89 (s, 1H), 6.85 (d, *J* = 8.5 Hz, 2H), 3.96 (s, 3H), 3.93 (s, 4H), 2.97 – 2.92 (m, 2H), 2.44 (s, 2H), 1.19 (dd, *J* = 14.8, 7.6 Hz, 5H), 1.07 – 1.05 (m, 12H).

^{13}C NMR (126 MHz, $CDCl_3$): δ 199.59, 155.90, 152.79, 148.28, 140.16, 135.65, 131.65, 131.17, 130.18, 123.91, 123.62, 120.06, 119.98, 119.53, 115.25, 114.64, 112.57, 102.40, 56.51, 56.39, 29.92, 17.51, 17.45, 13.42, 8.42.

HRMS: Calculated mass for $[M+H]^+$ = 534.2470; observed mass: 534.2470

IR (cm^{-1}): 2928, 2855, 2219, 1689, 1605, 1504, 1463, 1269, 1139, 919.

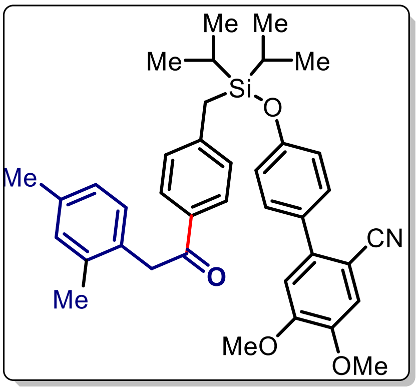

4'-(((4-(2-(2,4-dimethylphenyl)acetyl)benzyl)diisopropylsilyl)oxy)-4,5-dimethoxy-[1,1'-biphenyl]-2-carbonitrile (1w): *Para*-ketonisation has been done following the general procedure A in 0.05 mmol scale.

R_f value: 0.34 (20% ethyl acetate in pet ether)

Column material: 100-200 mesh silica

Eluent: pet ether:ethyl acetate (91: 9)

Yield: 63% (19.1 mg); (brsm 94%)

Physical appearance: Colorless viscous liquid

^{1}H NMR (500 MHz, $CDCl_3$): δ 7.88 (d, *J* = 8.3 Hz, 1H), 7.67 (dd, *J* = 6.2, 4.7 Hz, 1H), 7.64 (s, 1H), 7.58 – 7.53 (m, 1H), 7.49 – 7.47 (m, 1H), 7.38 (dd, *J* = 8.5, 1.6 Hz, 2H), 7.20 (dd, *J* = 8.1, 2.6 Hz, 2H), 6.89 (s, 1H), 6.87 – 6.84 (m, 2H), 4.22 (s, 1H), 3.95 (s, 3H), 3.93 (s, 3H), 2.36 (s, 2H), 2.29 (d, 6H), 1.09 – 1.04 (m, 12H).

^{13}C NMR (126 MHz, $CDCl_3$): δ 197.48, 156.00, 152.81, 132.38, 132.30, 132.18, 131.95, 131.67, 131.36, 130.57, 130.41, 130.14, 129.19, 129.09, 128.99, 128.77, 128.68, 126.92, 125.97, 120.13, 119.50, 115.27, 112.58, 102.51, 56.51, 56.39, 43.08, 21.20, 17.65, 17.59, 14.33, 13.22.

HRMS: Calculated mass for $[M+H]^+$ = 606.3034; observed mass: 606.3037.

IR (cm^{-1}): 2993, 2825, 2798, 2220, 1688, 1601, 1502, 1262, 1217, 1136.

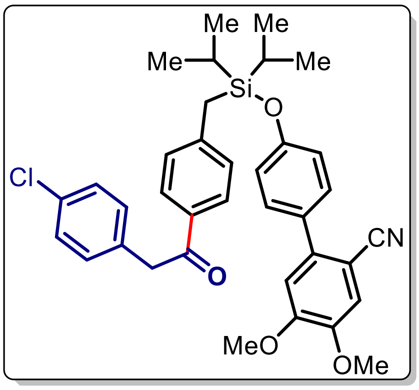

4'-(((4-(2-(4-chlorophenyl)acetyl)benzyl)diisopropylsilyl)oxy)-4,5-dimethoxy-[1,1'-biphenyl]-2-carbonitrile (1x): *Para*- ketonisation has been done following the general procedure in 0.05 mmol scale.

R_f value: 0.33 (20% ethyl acetate in pet ether)

Column material: 100-200 mesh silica

Eluent: pet ether:ethyl acetate (91: 9)

Yield: 65% (19.9 mg); (brsm 93%)

Physical appearance: Colorless viscous liquid

^{1}H NMR (500 MHz, $CDCl_3$): δ 7.88 (d, *J* = 8.3 Hz, 2H), 7.39 – 7.37 (m, 2H), 7.21 (s, 1H), 7.17 (dd, *J* = 6.4, 1.6 Hz, 2H), 7.14 (s, 1H), 7.11 (d, *J* = 7.7 Hz, 2H), 6.89 (s, 1H), 6.86 – 6.84 (m, 2H), 4.26 (s, 2H), 3.96 (s, 3H), 3.93 (s, 3H), 2.65 (s, 2H), 1.23 – 1.21 (m, 3H), 1.08 – 1.06 (m, 12H).

^{13}C NMR (126 MHz, $CDCl_3$): δ 197.28, 155.98, 152.77, 148.24, 145.57, 140.16, 133.00, 132.08, 131.68, 130.48, 130.13, 129.26, 128.76, 127.28, 126.05, 120.12, 115.26, 112.57, 102.41, 56.50, 56.38, 43.45, 22.06, 17.65, 17.58, 13.21.

HRMS: Calculated mass for $[M+H]^+$ = 612.2331; observed mass: 612.2331

IR (cm^{-1}): 2935, 2869, 2219, 1689, 1600, 1500, 1462, 1443, 1240, 1216, 1028.

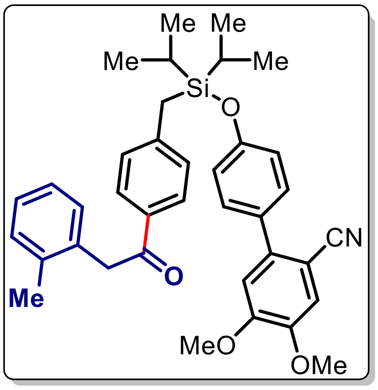

4'-((diisopropyl(4-(2-(o-tolyl)acetyl)benzyl)silyl)oxy)-4,5-dimethoxy-[1,1'-biphenyl]-2-carbonitrile (1y): *Para*- ketonisation has been done following the general procedure A in 0.05 mmol scale.

R_f value: 0.33 (20% ethyl acetate in pet ether)

Column material: 100-200 mesh silica

Eluent: pet ether:ethyl acetate (91: 9)

Yield: 61% (18.1 mg); (brsm 95%)

Physical appearance: Colorless viscous liquid

^{1}H NMR (500 MHz, $CDCl_3$): δ 8.03 (d, *J* = 8.3 Hz, 1H), 7.88 (d, *J* = 7.8 Hz, 1H), 7.45 (t, *J* = 7.6 Hz, 1H), 7.38 (d, *J* = 8.3 Hz, 2H), 7.21 (d, *J* = 7.8 Hz, 2H), 7.17 (d, *J* = 5.6 Hz, 2H), 7.13 (s, 1H), 7.11 (d, *J* = 8.3 Hz, 2H), 6.89 (s, 1H), 6.85 (d, *J* = 7.9 Hz, 2H), 4.26 (s, 1H), 3.95 (s, 2H), 3.93 (s, 3H), 2.64 (s, 3H), 2.24 (s, 2H), 1.28 (s, 1H), 1.06 (t, *J* = 7.5 Hz, 12H).

HRMS: Calculated mass for $[M+H]^+$ = 592.2878; observed mass: 592.2876

IR (cm^{-1}): 2924, 2870, 2220, 1687, 1603, 1502, 1463, 1386, 1265, 1194, 1103, 1031.

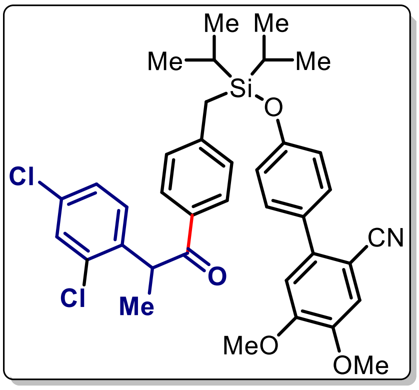

4'-(((4-(2-(2,4-dichlorophenyl)propanoyl)benzyl)diisopropylsilyl)oxy)-4,5-dimethoxy-[1,1'-biphenyl]-2-carbonitrile (1z): *Para*- ketonisation has been done following the general procedure A in 0.05 mmol scale.

R_f value: 0.33 (20% ethyl acetate in pet ether)

Column material: 100-200 mesh silica

Eluent: pet ether:ethyl acetate (91: 9)

Yield: 77% (25.4 mg); (brsm 94%)

Physical appearance: Colorless viscous liquid

^{1}H NMR (500 MHz, $CDCl_3$): δ 7.77 (d, *J* = 8.1 Hz, 2H), 7.37 (dd, *J* = 8.9, 5.0 Hz, 3H), 7.15 (d, *J* = 7.0 Hz, 3H), 7.10 (dd, *J* = 10.0, 4.9 Hz, 2H), 6.89 (s, 1H), 6.82 (d, *J* = 8.4 Hz, 2H), 5.05 (dd, *J* = 13.8, 6.9 Hz, 1H), 3.97 (s, 3H), 3.94 (s, 3H), 2.42 (s, 2H), 1.45 (d, *J* = 6.9 Hz, 3H), 1.20 (dd, *J* = 14.7, 7.3 Hz, 2H), 1.02 (d, *J* = 7.4 Hz, 12H).

^{13}C NMR (126 MHz, $CDCl_3$): δ 199.58, 155.92, 152.75, 148.24, 145.69, 140.14, 138.35, 133.85, 133.39, 132.69, 131.66, 130.11, 129.76, 129.71, 129.23, 128.95, 127.91, 120.10, 119.48, 115.21, 112.53, 102.41, 56.50, 56.38, 43.61, 22.04, 17.99, 17.59, 17.54, 13.17, 13.14.

HRMS: Calculated mass for $[M+Na]^+$ = 682.1905; observed mass: 682.1918.

IR (cm^{-1}): 3026, 2841, 2866, 2219, 1685, 1600, 1500, 1264, 1212, 1133, 917.

Mechanistic Studies:

Control Experiments:

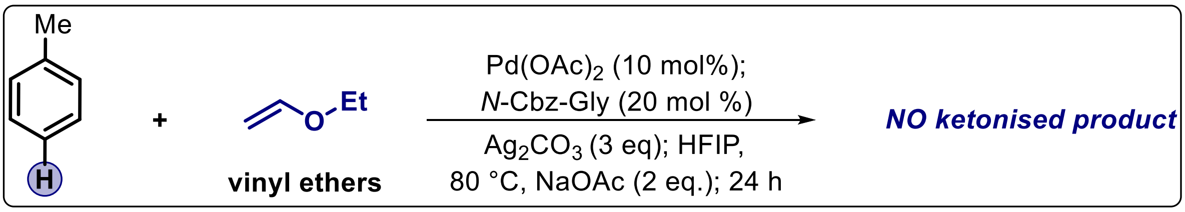

In order to understand the role of directing group, simple toluene as the substrate was treated under the standard condition and the product composition of the reaction mixture was monitored by GC and GCMS. GCMS data showed the presence of toluene homo-coupled product.

GCMS Data:

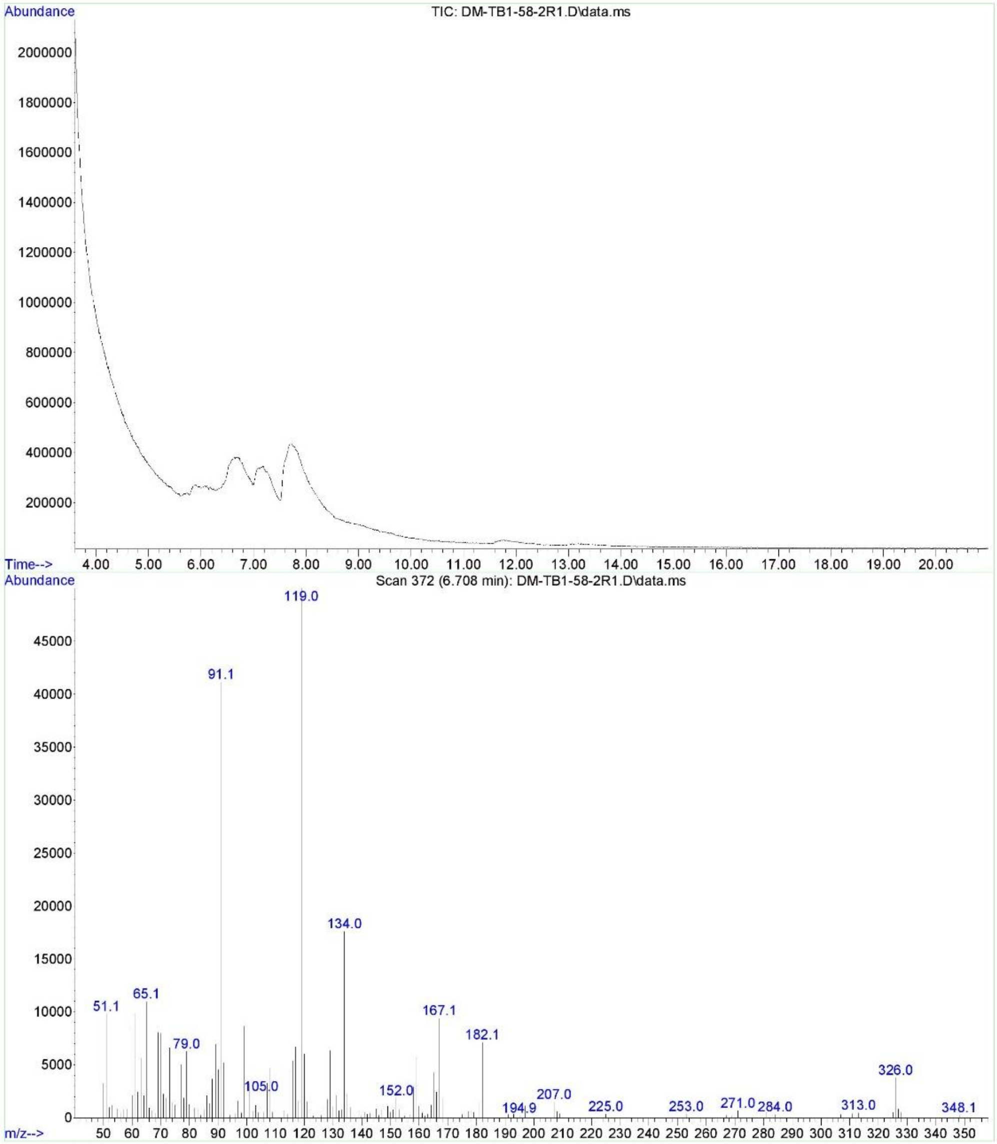

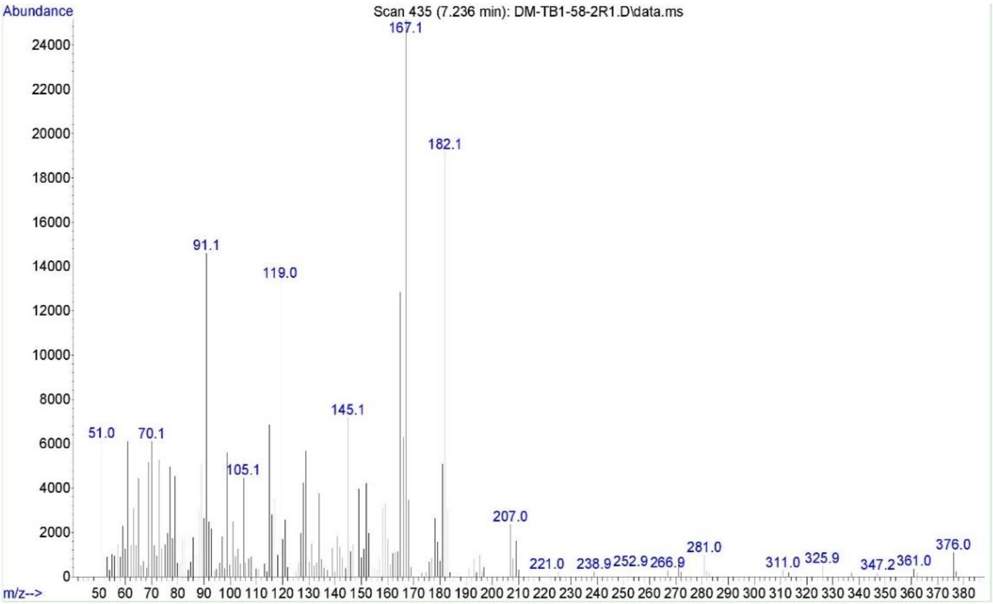

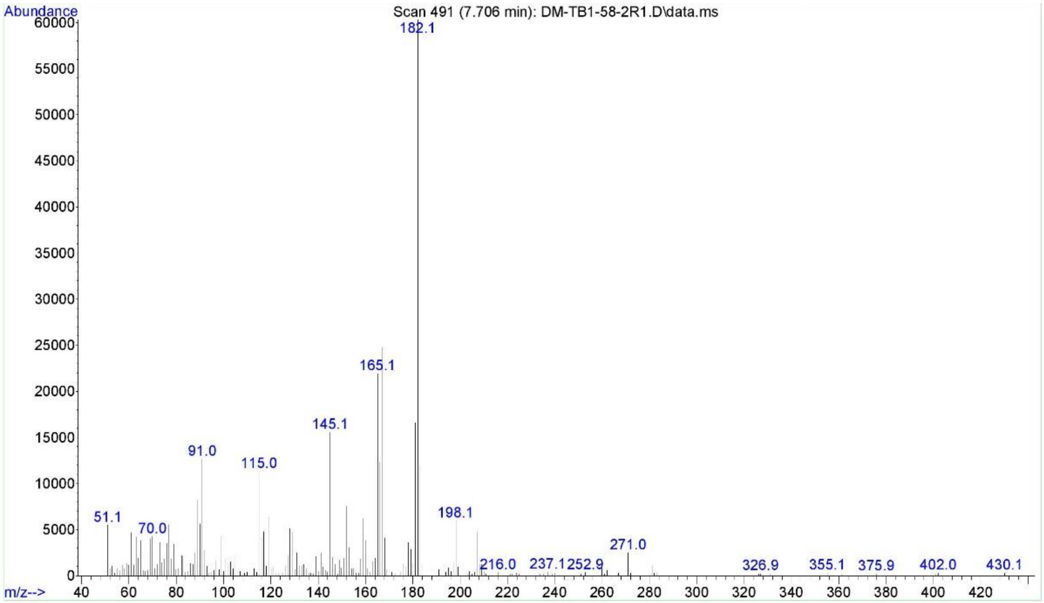

Supplementary Figure 2: GCMS signals of the control reaction mixture

Unknown: Scan 422 (7.127 min): DM-TB1-58-2R1.D\data.ms
Compound in Library Factor = -1169

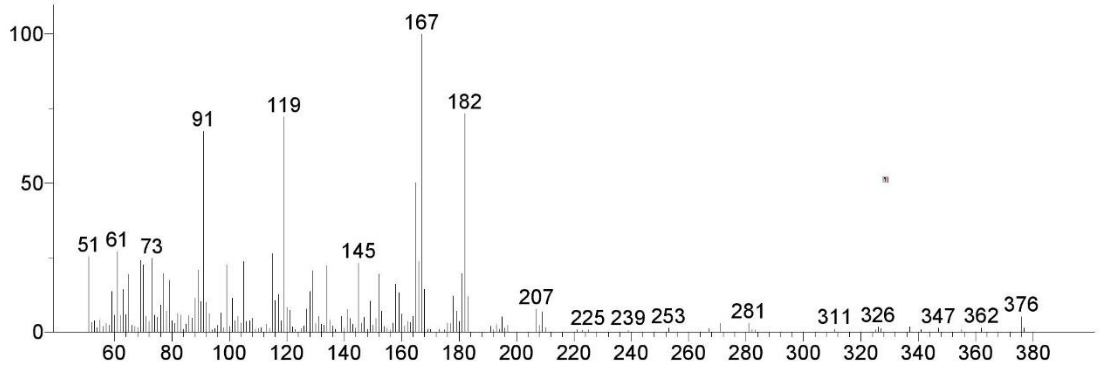

Hit 1 : Benzene, 1-methyl-3-(phenylmethyl)-
C14H14; MF: 664; RMF: 825; Prob 10.8%; CAS: 620-47-3; Lib: mainlib; ID: 124559.

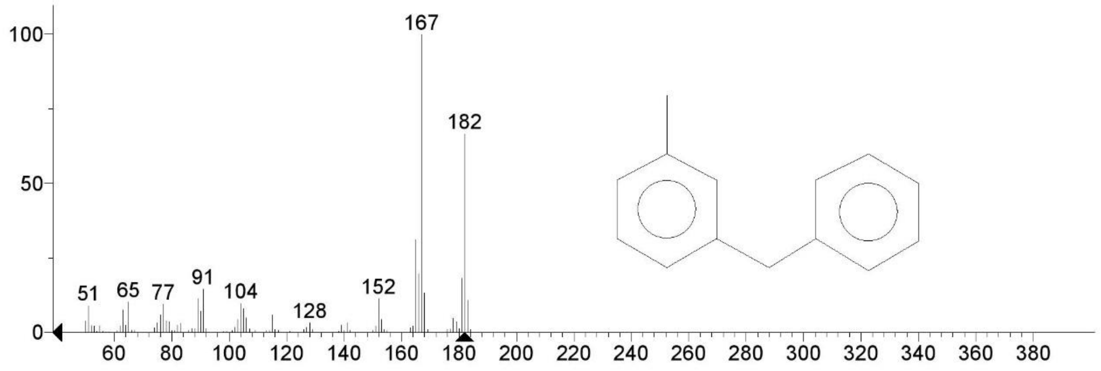

Hit 2 : Benzene, 1-methyl-4-(phenylmethyl)-
C14H14; MF: 660; RMF: 753; Prob 9.14%; CAS: 620-83-7; Lib: replib; ID: 21898.

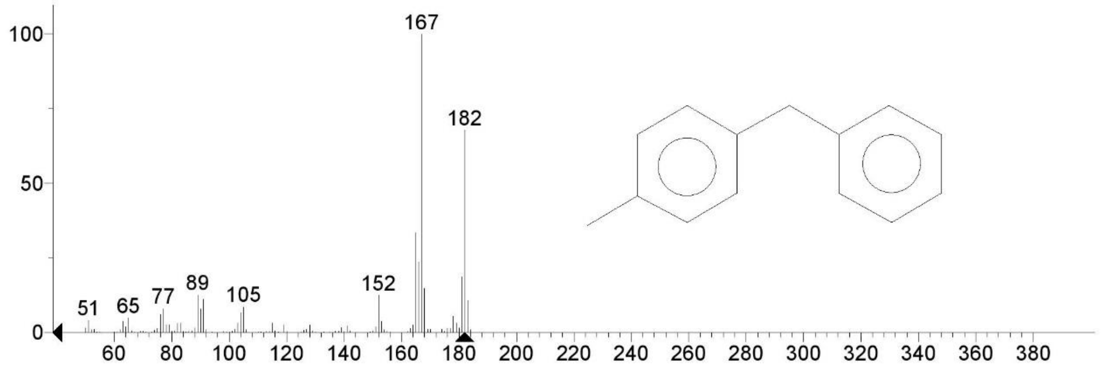

Supplementary Figure 3: GCMS assessment report for peak at 7.2 mins

Unknown: Scan 493 (7.723 min): DM-TB1-58-2R1.D\data.ms
Compound in Library Factor = -816

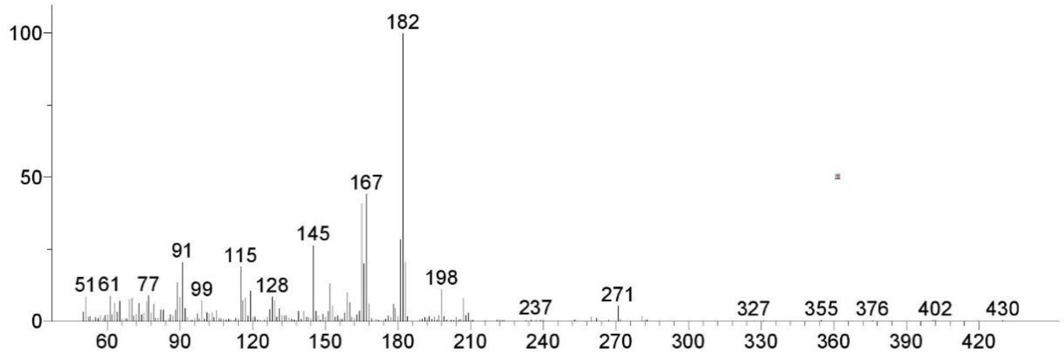

Hit 1 : 4,4'-Dimethylbiphenyl
C14H14; MF: 732; RMF: 906; Prob 13.6%; CAS: 613-33-2; Lib: replib; ID: 23113.

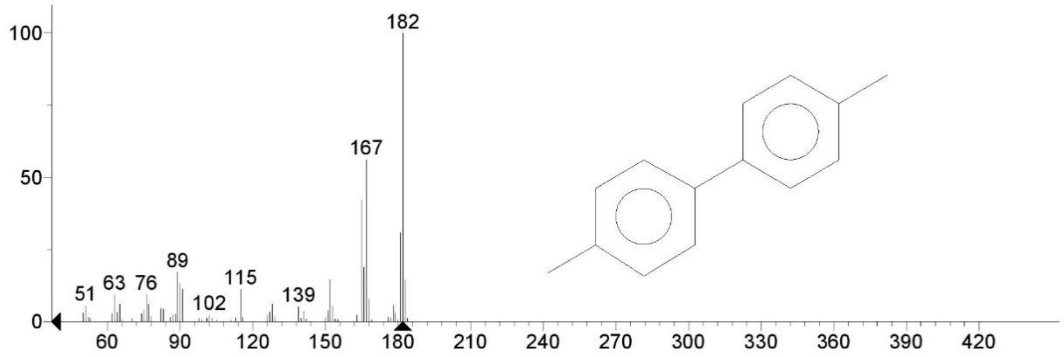

Hit 2 : 3,3'-Dimethylbiphenyl
C14H14; MF: 732; RMF: 868; Prob 13.6%; CAS: 612-75-9; Lib: mainlib; ID: 134734.

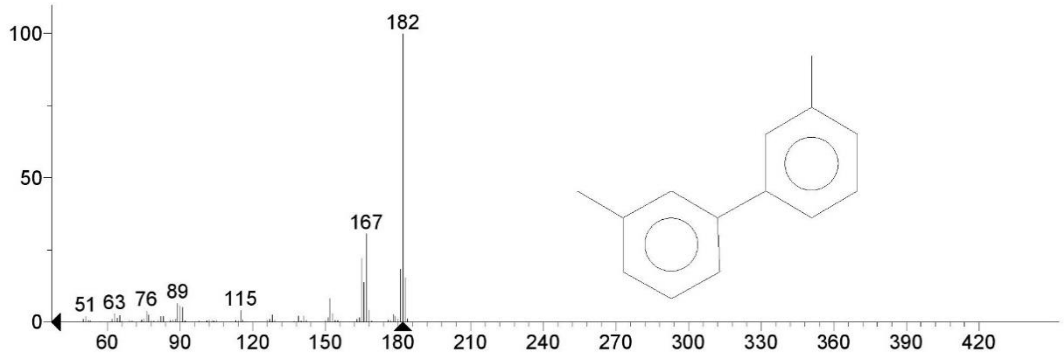

Supplementary Figure 4: GCMS assessment report for peak at 7.7 mins

The absence of the ketonised product in the reaction mixture was confirmed by comparing the signal with pure 2- and 4-methyl acetophenone.

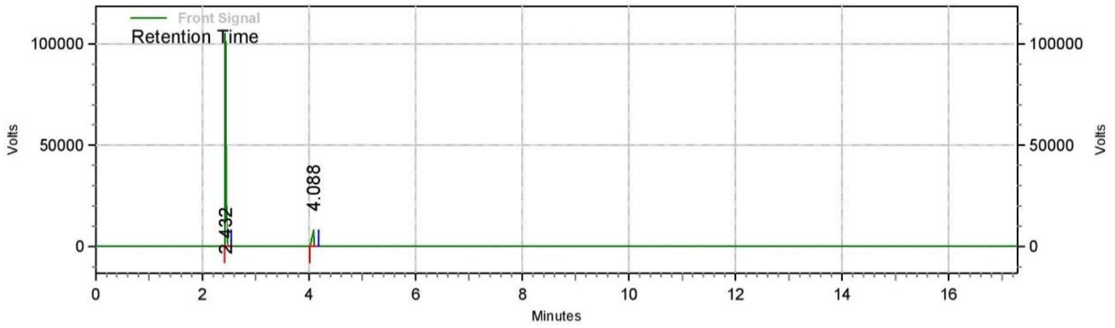

Front Signal
Results

Retention Time	Area	Area %	Height	Height %
2.432	914420225	85.82	808614033	92.97
4.088	151126306	14.18	61136298	7.03
Totals	1065546531	100.00	869750331	100.00

Supplementary Figure 5: GC reference for 2-methyl acetophenone

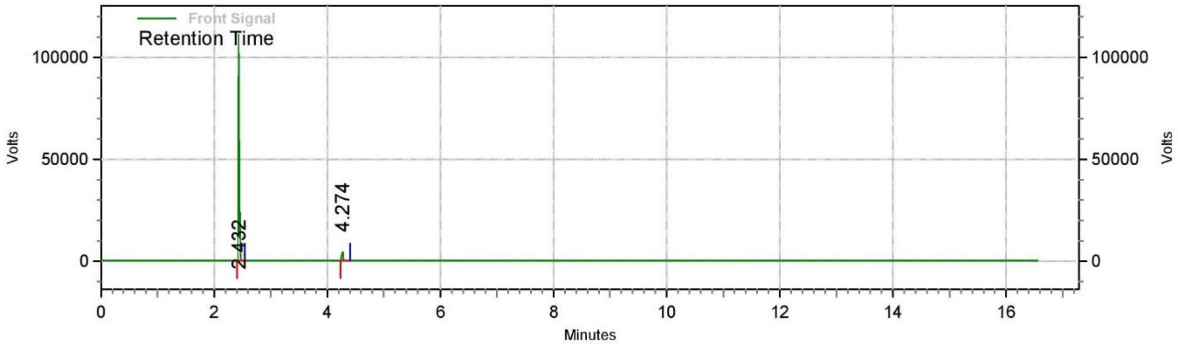

Front Signal
Results

Retention Time	Area	Area %	Height	Height %
2.432	951696917	94.16	854245445	96.21
4.274	58976944	5.84	33633221	3.79
Totals	1010673861	100.00	887878666	100.00

Supplementary Figure 6: GC reference for 4-methyl acetophenone

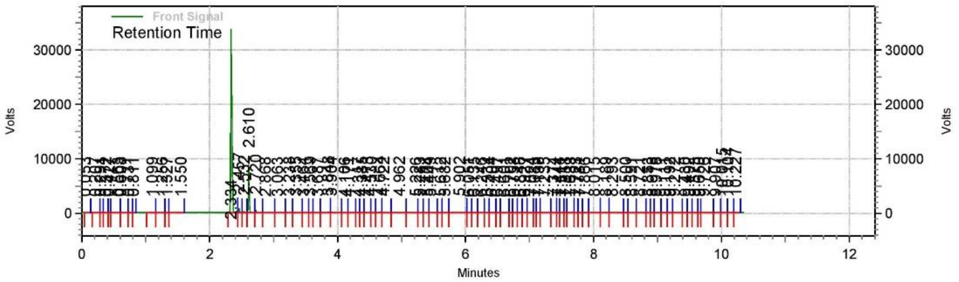

Front Signal
Results

Retention Time	Area	Area %	Height	Height %
0.053	1572	0.00	489	0.00
0.207	1977	0.00	678	0.00
0.291	862	0.00	425	0.00
0.372	1385	0.00	618	0.00
0.427	709	0.00	575	0.00
0.563	3109	0.00	727	0.00
0.608	3058	0.00	656	0.00
0.731	1641	0.00	645	0.00
0.811	1022	0.00	627	0.00
1.099	6241	0.00	1165	0.00
1.256	5824	0.00	906	0.00
1.327	2041	0.00	901	0.00
1.550	5691	0.00	688	0.00
2.334	344388651	77.22	259023907	74.71
2.457	6687160	1.50	5961264	1.72
2.532	1932058	0.43	1739967	0.50
2.610	67570347	15.15	68769695	19.84
2.720	3673900	0.82	3197471	0.92
2.868	299027	0.07	41679	0.01
3.063	216176	0.05	57152	0.02
3.238	521150	0.12	426473	0.12
3.355	861025	0.19	533713	0.15
3.464	158355	0.04	77712	0.02
3.569	245914	0.06	181460	0.05
3.687	162815	0.04	31756	0.01
3.848	1602334	0.36	1112035	0.32
3.904	1333199	0.30	676136	0.20
4.106	339757	0.08	124831	0.04
4.171	239127	0.05	57637	0.02
4.317	128589	0.03	87880	0.03
4.385	267392	0.06	219534	0.06
4.473	627470	0.14	343990	0.10
4.530	285060	0.06	158976	0.05

Supplementary Figure 7: GC data of reaction mixture (direct)

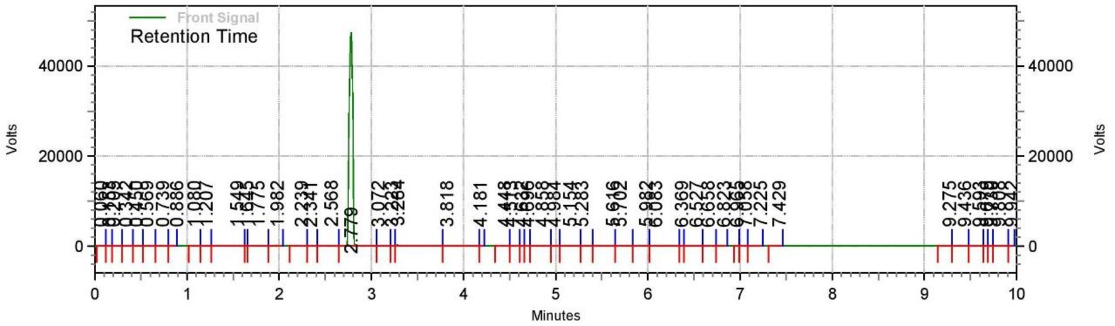

Front Signal
Results

Retention Time	Area	Area %	Height	Height %
0.060	1577	0.00	615	0.00
0.153	1267	0.00	523	0.00
0.205	2593	0.00	643	0.00
0.342	2828	0.00	825	0.00
0.450	2864	0.00	714	0.00
0.569	3813	0.00	699	0.00
0.739	3279	0.00	828	0.00
0.886	1638	0.00	668	0.00
1.080	6076	0.00	1138	0.00
1.207	5218	0.00	1284	0.00
1.549	16572	0.00	1104	0.00
1.645	1446	0.00	992	0.00
1.775	9141	0.00	1380	0.00
1.982	3783	0.00	795	0.00
2.239	4471	0.00	665	0.00
2.341	2222	0.00	781	0.00
2.568	873645	0.09	462766	0.13
2.779	988908814	99.66	363365652	99.45
3.072	224695	0.02	58618	0.02
3.223	35501	0.00	14116	0.00
3.284	1879017	0.19	1401416	0.38
3.818	55394	0.01	4786	0.00
4.181	1529	0.00	826	0.00
4.448	12829	0.00	2348	0.00
4.513	8875	0.00	1603	0.00
4.632	2999	0.00	1285	0.00
4.696	3307	0.00	1156	0.00
4.858	14493	0.00	1409	0.00
4.984	5375	0.00	1161	0.00
5.154	12393	0.00	1394	0.00
5.283	4897	0.00	1015	0.00
5.616	8289	0.00	963	0.00
5.702	9504	0.00	1322	0.00

Supplementary Figure 8: GC data of reaction mixture (diluted with ethyl acetate)

Effect of D2-HFIP solvent:

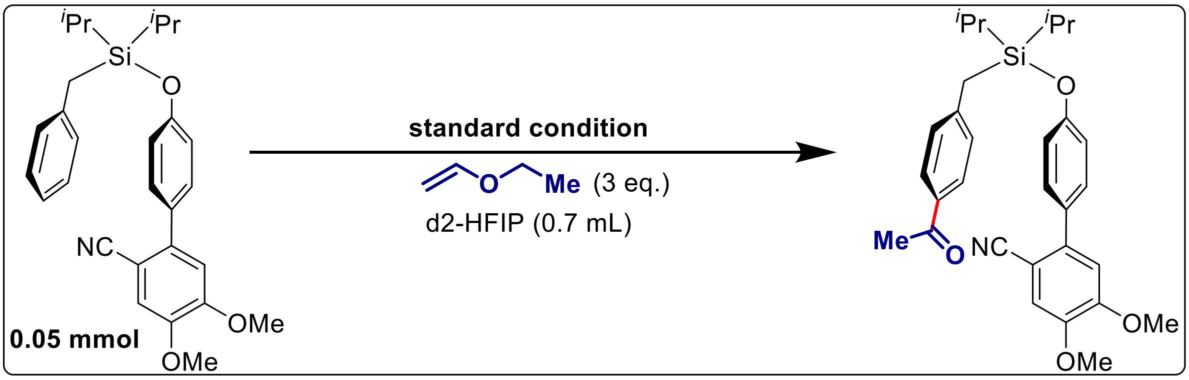

Procedure: In a clean oven dried reaction tube all the reaction components were added and capped. The tube was purged with dried air using Schlenk line set up and dried d2-HFIP (by passing through activated neutral alumina) was added. The mixture was then stirred on a preheated oil-bath for 24 h. Upon completion the reaction composition was monitored by TLC and NMR.

Vinyl Ether -HFIP interaction:

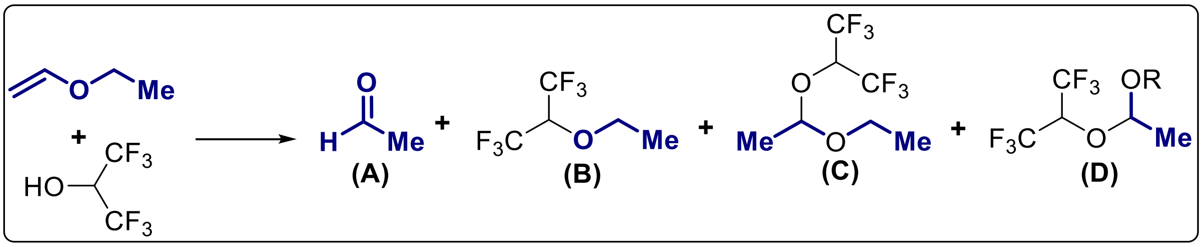

Procedure: In a clean NMR tube ethyl vinyl ether (0.1 mmol) was taken along with 0.6 mL of $CDCl_3$. The signal was recorded for the reference. In the following steps HFIP was added to the tube. The amount of HFIP added was increased from 0.5 eq. to 10 eq. and the signals were recorded following each addition. After the addition of 4 eq. of HFIP the changes in the NMR signal were prominent which further intensified upon heating. Finally, vinyl ether got consumed completely with the formation of multiple products. The products were also detected in GCMS.

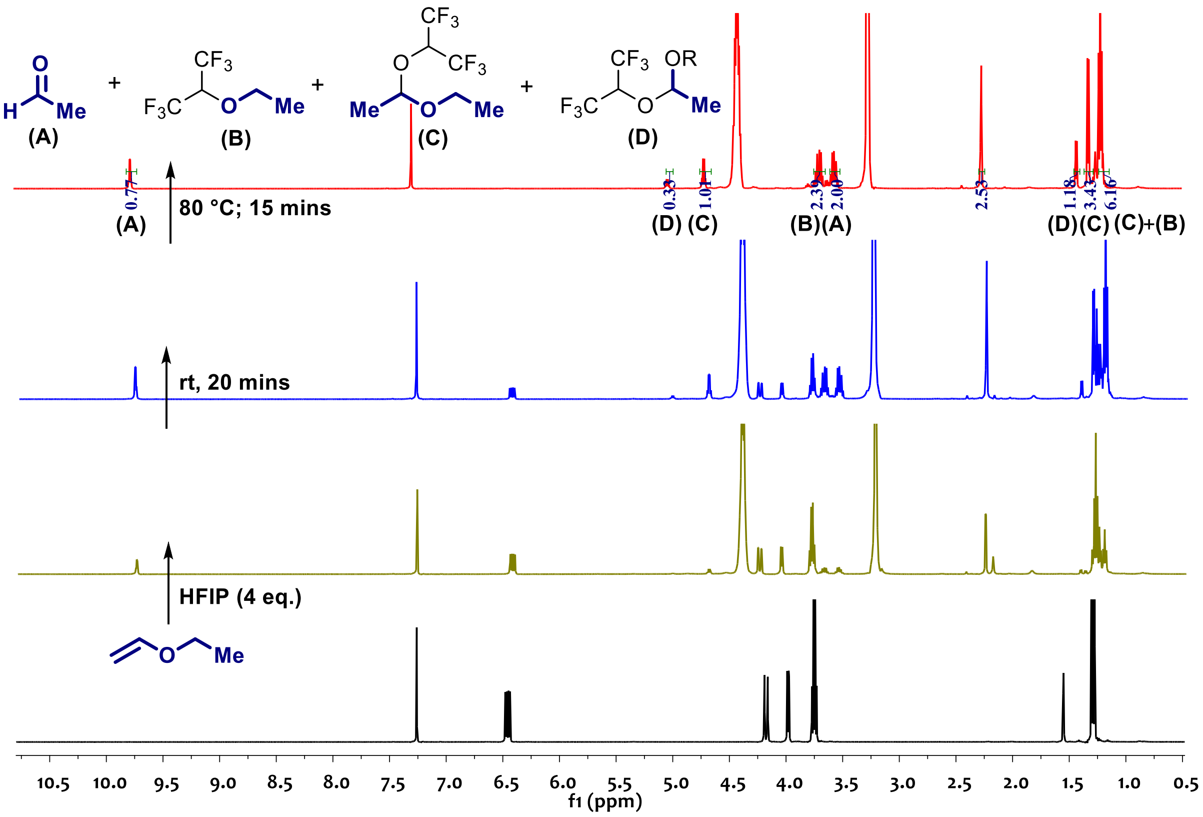

Supplementary Figure 9: NMR titration of HFIP and ethyl vinyl ether

Based on the spectroscopic observations a plausible pathway of the decomposition of vinyl ether was proposed. ***Notably none of the decomposition product is effective reagent for ketonisation.***

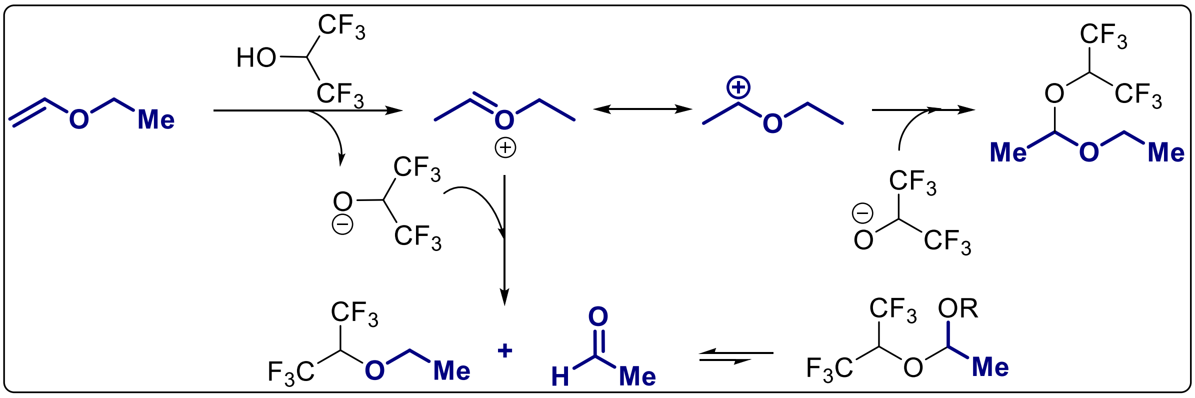

Kinetic Isotope Effect:

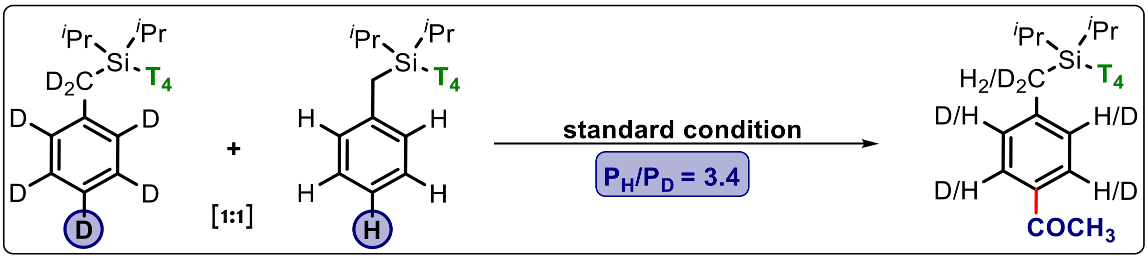

Procedure: The kinetic isotope effect was measured both from product distribution and rate constant ratio.

Product Distribution Study (*P_H/P_D*):

In a clean oven dried reaction tube 1:1 ratio mixture of the model substrate and deuterated variant (0.05 mmol each) was taken. All the remaining reagents were added according to the standard condition of a 0.1 mmol scale reaction. Upon completion of the reaction the product was isolated the product distribution was monitored by NMR. In this regard the signals corresponding to the –Me of acetyl group and the benzylic $–CH_2$ were compared.

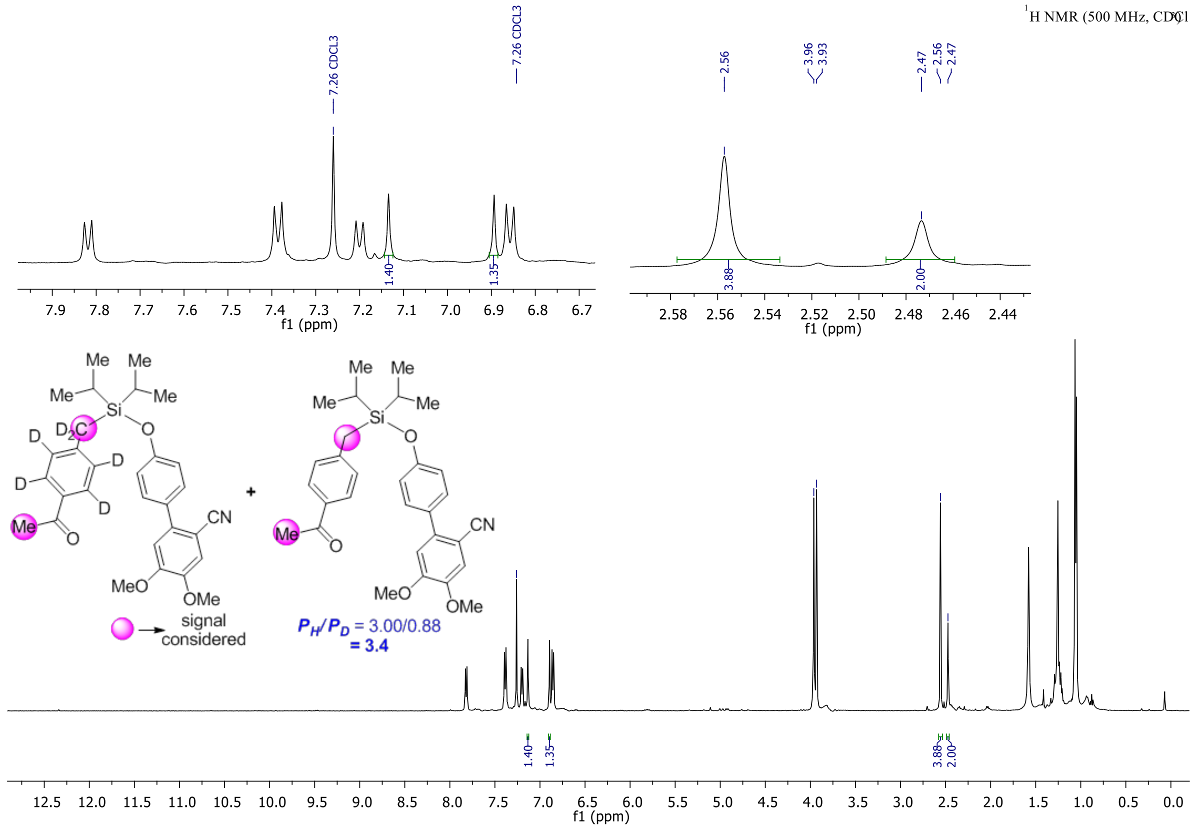

Supplementary Figure 10: NMR signals for product distribution

Determination of k_H/k_D:

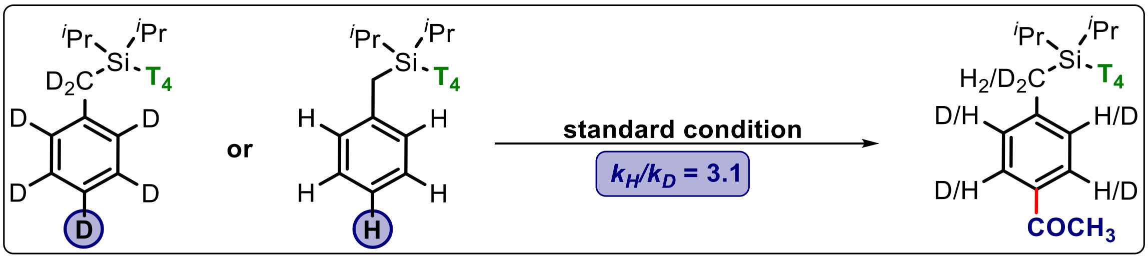

Procedure: Multiple reactions of 0.05 mmol scale reaction were set to with the model substrate and the progress of the reaction was monitored with time. Concentration of the product was measured for quantification.

Same set of experiments were repeated with the deuterated substrates and the formation of product was monitored.

Time (h)	Product conc. with model substrate (in 10^{-3} mM)	Product conc. with d_7-model substrate (in 10^{-3} mM)
0	0	0
0.5	2.4	0.8
1	4.8	2.3
2	10.9	3.3
3	14.3	4.6
4	18.2	5.4
5	22	6.2
6	28	7.5
8	35	9.2
10	42	11
12	48	12.8
16	57.1	13.3
20	60.2	14
24	61.6	14.3

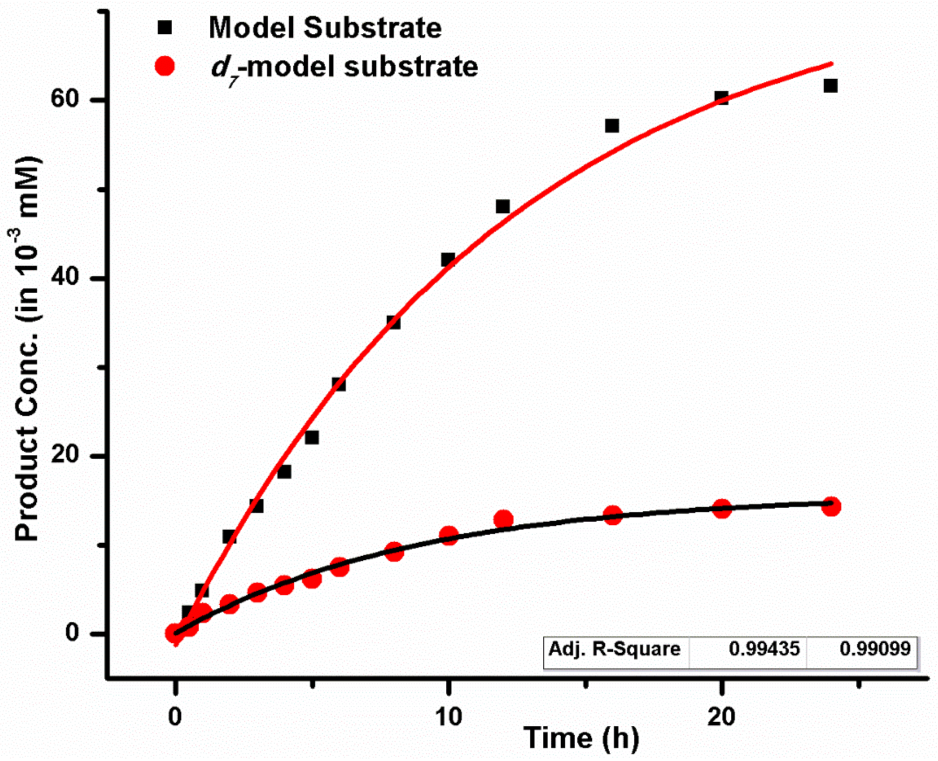

Supplementary Figure 11: Kinetic analysis with model substrate and deuterated model

Initial rate with model substrate (R_H) = 6.173 x 10^{-3} mM/h

Initial rate with d_7-model substrate (R_D) = 2.015 x 10^{-3} mM/h

k_H/k_D = R_H/R_D = 6.173/2.015 = 3.06 ≈ **3.1**

Applications:

Removal of Directing Group:

Part 1:

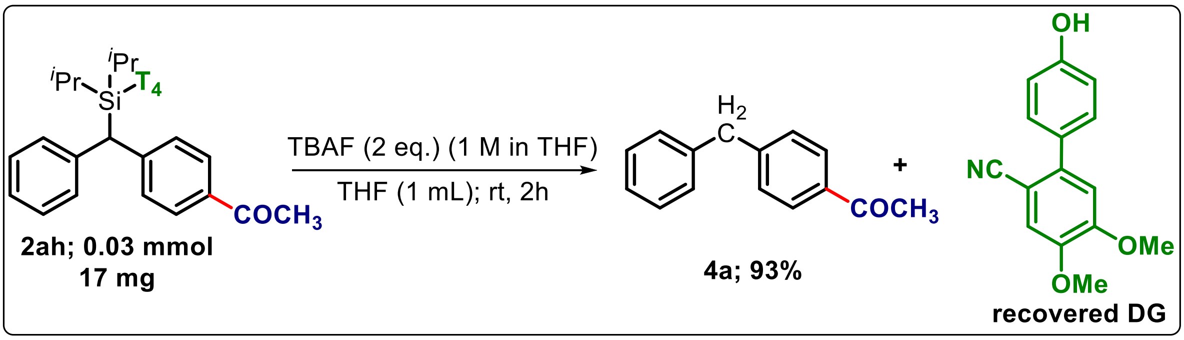

Procedure: In a clean reaction tube 0.03 mmol (17 mg) of 4'-((((4-acetylphenyl)(phenyl)methyl) diisopropylsilyl)oxy)-4,5-dimethoxy-[1,1'-biphenyl]-2-carbonitrile (**2ah**) was taken and dissolved in 1mL of THF. A solution of TBAF in THF [60 μL of 1.0 (M)] was added to the and stirred at room temperature for 2 h. The progress of the reaction was monitored by TLC. Upon completion the solvent was evaporated and the compound was isolated through column chromatography.

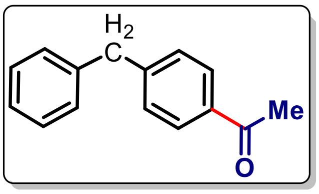

1-(4-benzylphenyl)ethan-1-one (5a):

R_f value: 0.6 (10% ethyl acetate in pet ether)

Column material: 100-200 mesh silica

Eluent: pet ether: ethyl acetate (97: 3)

Yield: 93% (5.9 mg)

Physical appearance: Colorless viscous liquid.

^{1}H NMR (500 MHz, $CDCl_3$): δ 7.91 – 7.87 (m, 2H), 7.33 – 7.27 (m, 4H), 7.22 (ddd, J = 7.4, 4.4, 1.3 Hz, 1H), 7.19 – 7.16 (m, 2H), 4.04 (s, 2H), 2.58 (s, 3H).

^{13}C NMR (126 MHz, $CDCl_3$): δ 198.08, 147.04, 140.26, 135.49, 133.92, 129.33, 129.16, 128.87, 126.64, 42.08, 26.79.

HRMS: Calculated mass for $[M+H]^+$ = 211.1117; observed mass: 211.0730

IR (cm^{-1}): 2922, 2855, 1683, 1604, 1504, 1359, 1270.

Part 2:

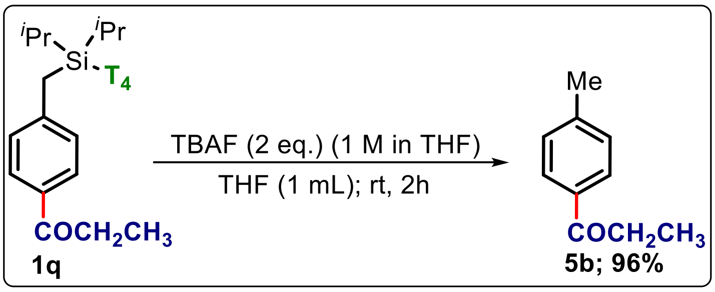

Procedure: Afore mentioned procedure has been followed for the generation of **5b**.

Characterization: The product formation was confirmed by comparing it with the literature reports.

α-Dibromination:

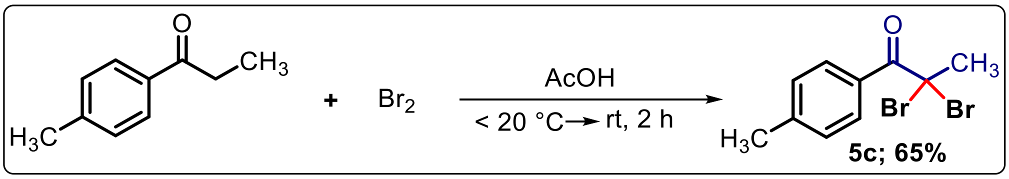

Procedure: A solution of bromine in acetic acid (1 eq.; 1 (M)) was added dropwise to a stirred solution of the substrate in acetic acid (2.45 mmol/mL) keeping the temperature below 20 ºC. After the addition the mixture was stirred at room temperature for 2 h. The reaction mixture was poured into 10 volumes on water. Solid precipitate obtained was filtered, washed with cold water and dried under vacuum.

^{1}H NMR (500 MHz, $CDCl_3$): δ 8.35 (d, *J* = 8.3 Hz, 2H), 7.30 (d, *J* = 8.2 Hz, 2H), 2.78 (s, 3H), 2.47 (s, 3H).

^{13}C NMR (126 MHz, $CDCl_3$): δ 188.12, 144.77, 131.75, 129.22, 128.88, 58.27, 37.97, 21.92.

Hydroxylation:

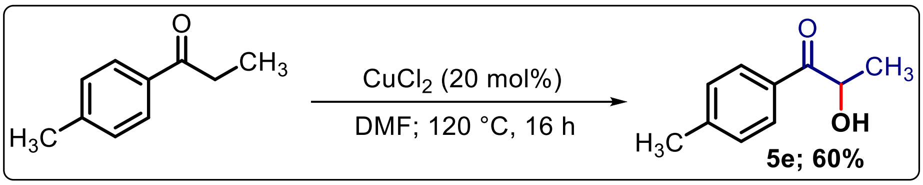

Procedure: In the clean oven dried reaction tube, ketone (0.1 mmol) was mixed with 20 mol% $CuCl_2$ in presence of 0.6 mL DMF and stirred on a preheated oil bath at 120 ºC for 16 h. The reaction was cooled, diluted with water and extracted with ethyl acetate. The organic portion was dried on anhydrous Na_2SO_4 and purified by column chromatography.

^{1}H NMR (400 MHz, $CDCl_3$): δ 8.13 (s, 1H), 7.85 (d, *J* = 8.2 Hz, 2H), 7.29 (d, *J* = 8.1 Hz, 2H), 6.10 (q, *J* = 7.0 Hz, 1H), 2.42 (s, 3H), 1.57 (d, *J* = 7.0 Hz, 3H).

^{13}C NMR (126 MHz, $CDCl_3$): δ 195.61, 160.29, 145.05, 131.71, 129.77, 128.84, 71.16, 21.94, 17.55.

Mono bromination:

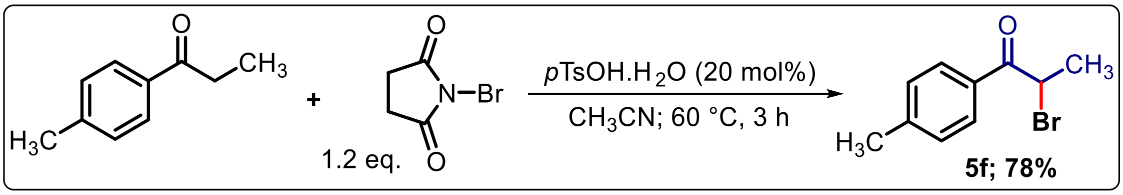

Procedure: In an over dried clean reaction tube ketone (0.2 mmol), N-bromosuccinimide (1.2 eq) and *p*-toluenesulphonic acid (20 mol%) were mixed followed by the addition of 1 mL of acetonitrile. The reaction was stirred on a preheated oil bath at 60 ºC for 4 h. The mixture was dried, suspended in water followed by extraction with ethyl acetate. Pure product (**4f**) was obtained upon purification through column chromatography.

^{1}H NMR (400 MHz, $CDCl_3$): δ 7.95 – 7.91 (m, 2H), 7.29 (d, *J* = 8.0 Hz, 2H), 5.28 (q, *J* = 6.6 Hz, 1H), 2.43 (s, 3H), 1.89 (d, *J* = 6.6 Hz, 3H).

^{13}C NMR (101 MHz, $CDCl_3$): δ 193.26, 144.89, 131.70, 129.68, 129.26, 41.74, 21.94, 20.39.

Pyridine Synthesis:

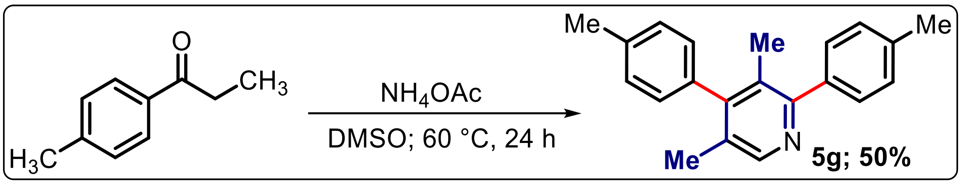

Procedure: In a clean reaction tube ketone substrate was added to the solution of NH_4OAc in DMSO. The mixture was stirred vigorously on a preheated oil bath at 120 ºC for 24 h. Upon completion the reaction was diluted with water and extracted with ethyl acetate. The compound was concentrated and purified through column chromatography.

^{1}H NMR (500 MHz, $CDCl_3$): δ 8.41 (s, 1H), 7.41 (d, *J* = 8.0 Hz, 2H), 7.27 (d, *J* = 7.8 Hz, 2H), 7.24 (d, *J* = 7.9 Hz, 2H), 7.04 (d, *J* = 7.9 Hz, 2H), 2.41 (d, *J* = 4.8 Hz, 3H), 2.39 (d, *J* = 6.4 Hz, 3H), 2.03 (d, *J* = 8.8 Hz, 3H), 2.00 (s, 3H).

^{13}C NMR (126 MHz, $CDCl_3$): δ 157.11, 150.92, 147.57, 138.53, 137.49, 137.24, 136.11, 130.02, 129.64, 129.23, 128.95, 128.79, 128.17, 21.46, 18.26, 17.73.

Computational Details and Discussions:

All calculations were performed with Gaussian 09.[2] The B3LYP [3,4] functional and a mixed basis set of SDD for Pd and 6-31G(d) for other atoms were used in geometry optimizations. Single-point energies were calculated using M06[5] and a mixed basis set of SDD for Pd and 6-311+G(d,p) for other atoms. Solvation energy corrections were calculated using the SMD[6] implicit solvation model. HFIP was used as the solvent. Since the solvent parameters for HFIP are not available in Gaussian 09, the solvent parameters of isopropanol were used and the dielectric constant of the solvent was modified to the dielectric constant of HFIP ($\varepsilon = 16.7$) by using the "scrf=(smd, solvent=2-propanol, read)" keywords in Gaussian 09. The 3D structures were generated using CYLView.[7]

In the main manuscript (Figure 5a), only part of the catalytic cycle of the reaction of **1** with vinyl methyl ether was shown. The complete energy profile of the Pd-catalyzed *para*-functionalization of **1** is shown below. As revealed in previous computational studies of Pd(II)-catalyzed C−H activation with mono-*N*-protected amino acid (MPAA) ligands,[8] the *para*-C–H activation (**s-TS1**) occurred via the CMD mechanism with the *N*-acyl group on the MPAA ligand serving as a base. The energetics of the C−H activation pathway of **1** and the origin of *para*-selectivity was found to follow the same trend as observed during *para*-selective silylation reaction.[1] Following the C-H activation, intermediate **s4** tautomerizes and rearranges to the more stable palladacycle **5** with the carboxylic group bound to the Pd center in an κ^2 fashion. The coordination of vinyl methyl ether to the Pd center in **5** leads to a slightly more stable intermediate **6**, which undergoes migratory insertion via **TS1** followed by β-H elimination (**TS2**). These two steps are both facile and require lower barriers than the C–H activation step. The resulting Pd(II) hydride **8** then undergoes reductive elimination to form the alkenyl ether product. Finally, oxidation of Pd(0) regenerates the Pd(II) active catalyst. The final reductive elimination and oxidation steps are expected to be facile and thus are not studied computationally. The C–H activation is irreversible and represents the rate- and selectivity-determining step in the overall catalytic cycle.

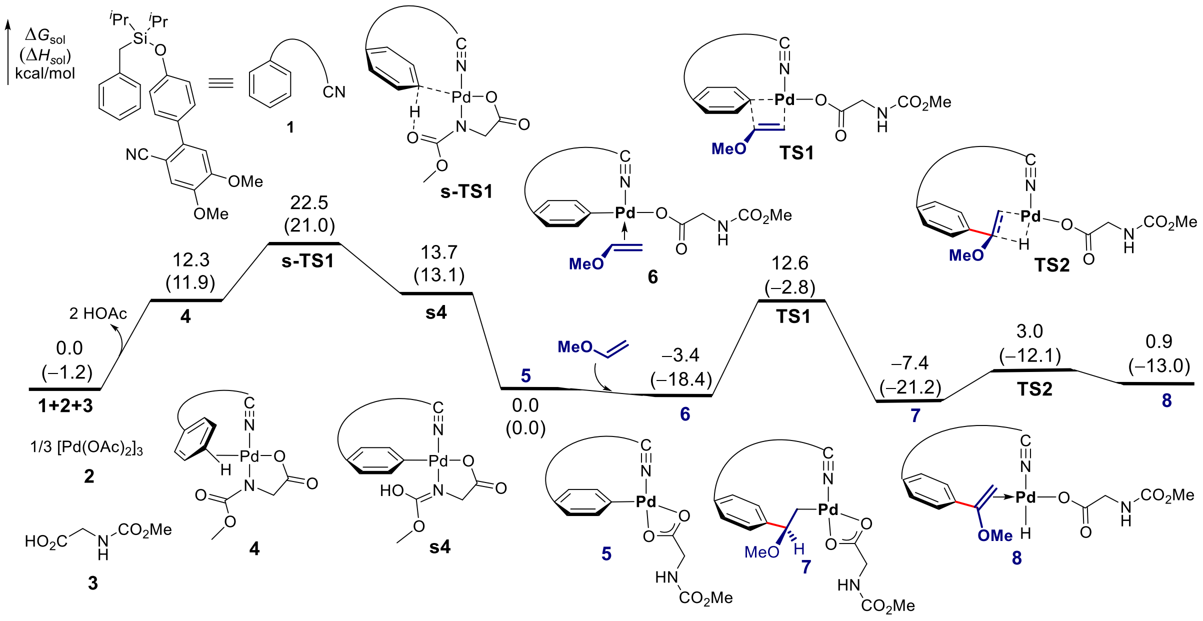

Supplementary Figure 12: Energy profile of the catalytic cycle

NMR Characterization:

Substrates:

Supplementary Figure 13: ^{1}H and ^{13}C NMR (500 MHz, $CDCl_3$) of 4'-(((2,3-difluoro-6-methoxybenzyl)diisopropylsilyl)oxy)-4,5-dimethoxy-[1,1'-biphenyl]-2-carbonitrile

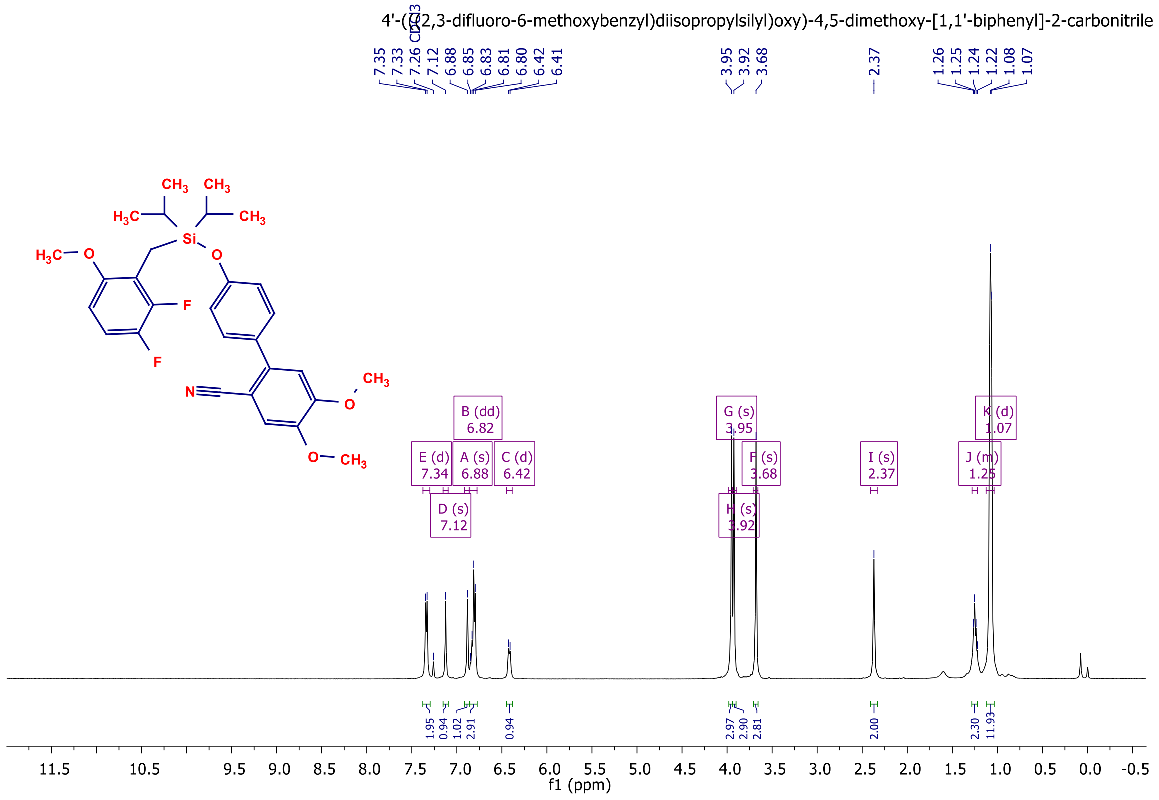

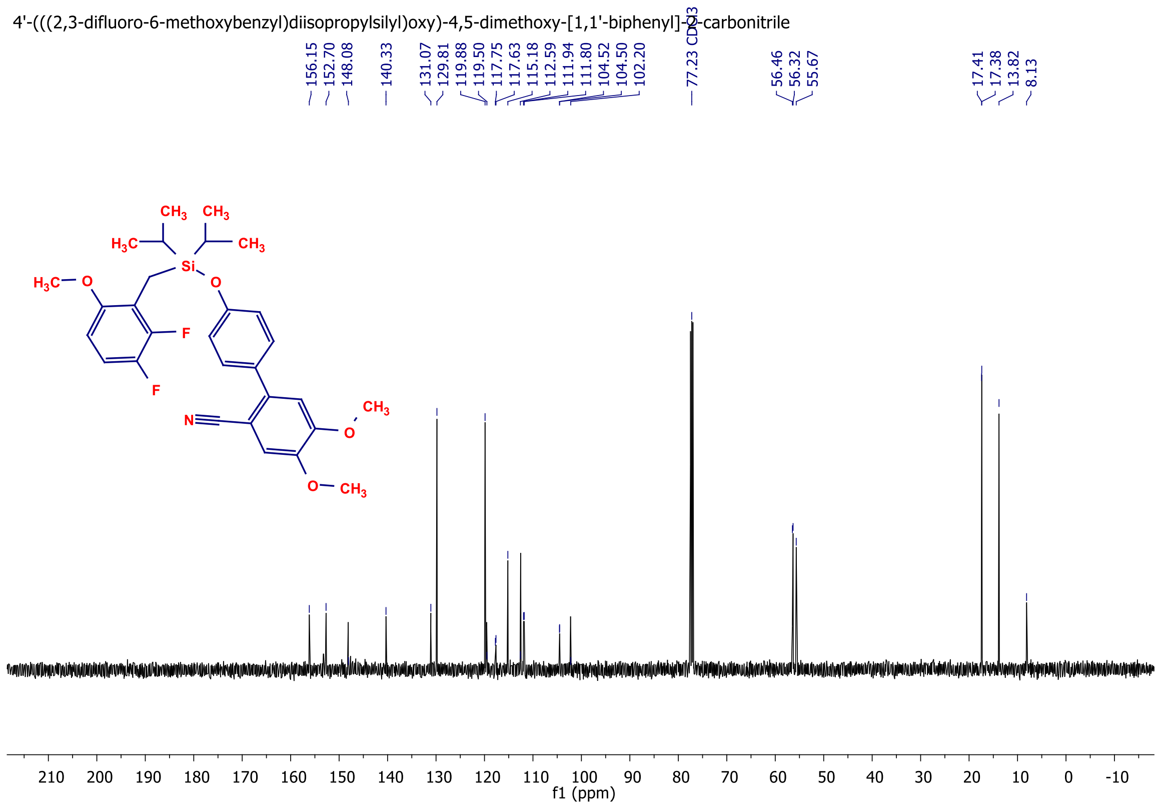

Supplementary Figure 14: ^{1}H and ^{13}C NMR (500 MHz, $CDCl_3$) of 4'-(((3-fluoro-2-(trifluoromethyl)benzyl)diisopropylsilyl)oxy)-4,5-dimethoxy-[1,1'-biphenyl]-2-carbonitrile:

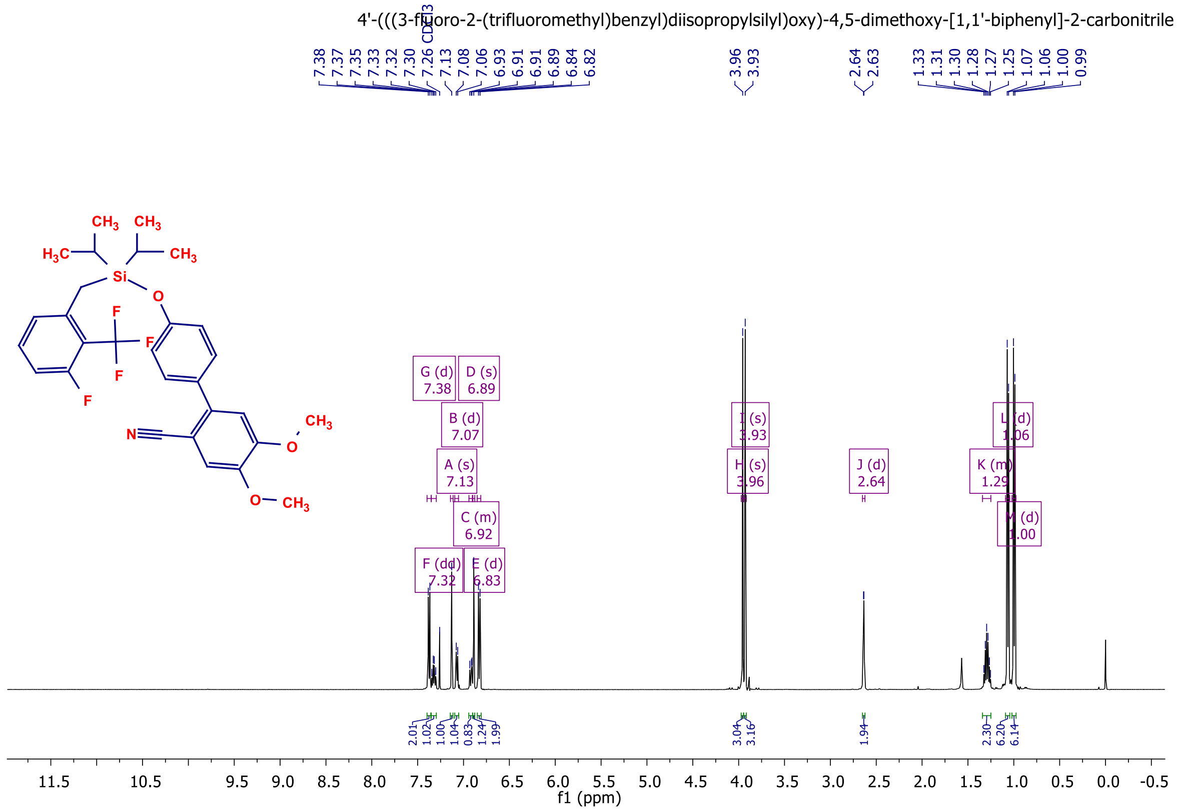

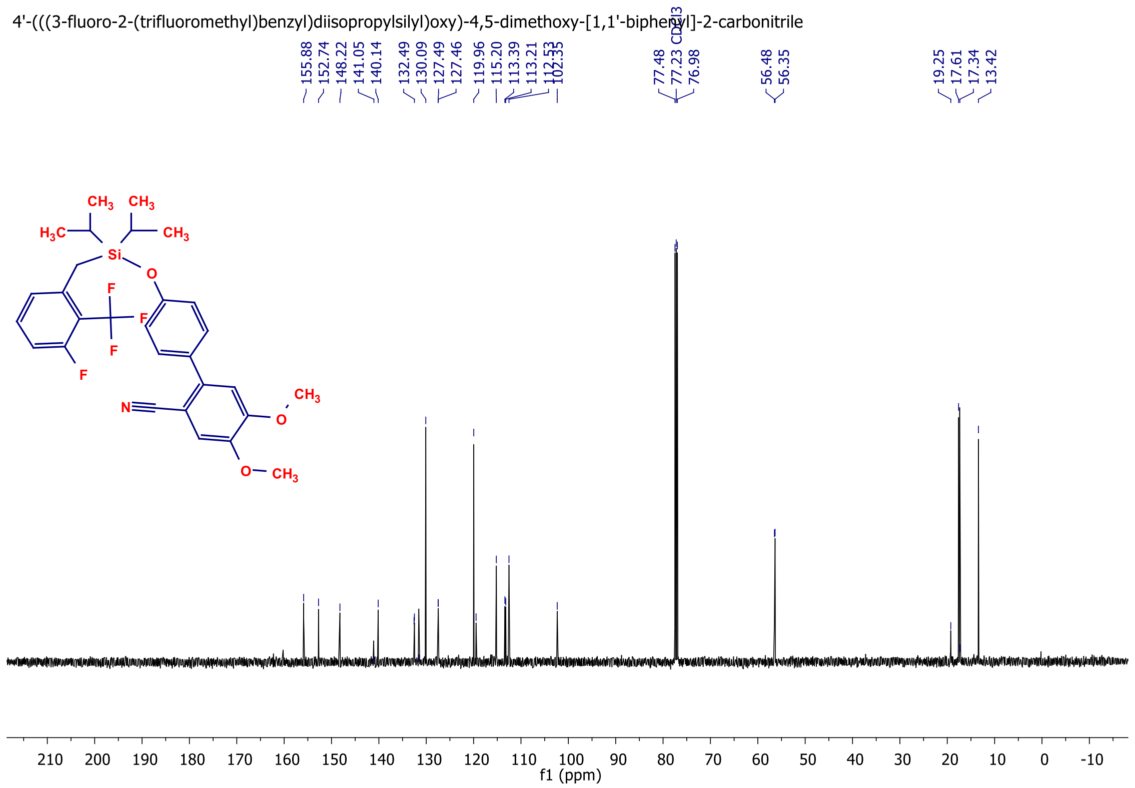

Supplementary Figure 15: ^{1}H and ^{13}C NMR (500 MHz, $CDCl_3$) of 4'-((diisopropyl(2-methoxy-6-(trifluoromethyl)benzyl)silyl)oxy)-4,5-dimethoxy-[1,1'-biphenyl]-2-carbonitrile:

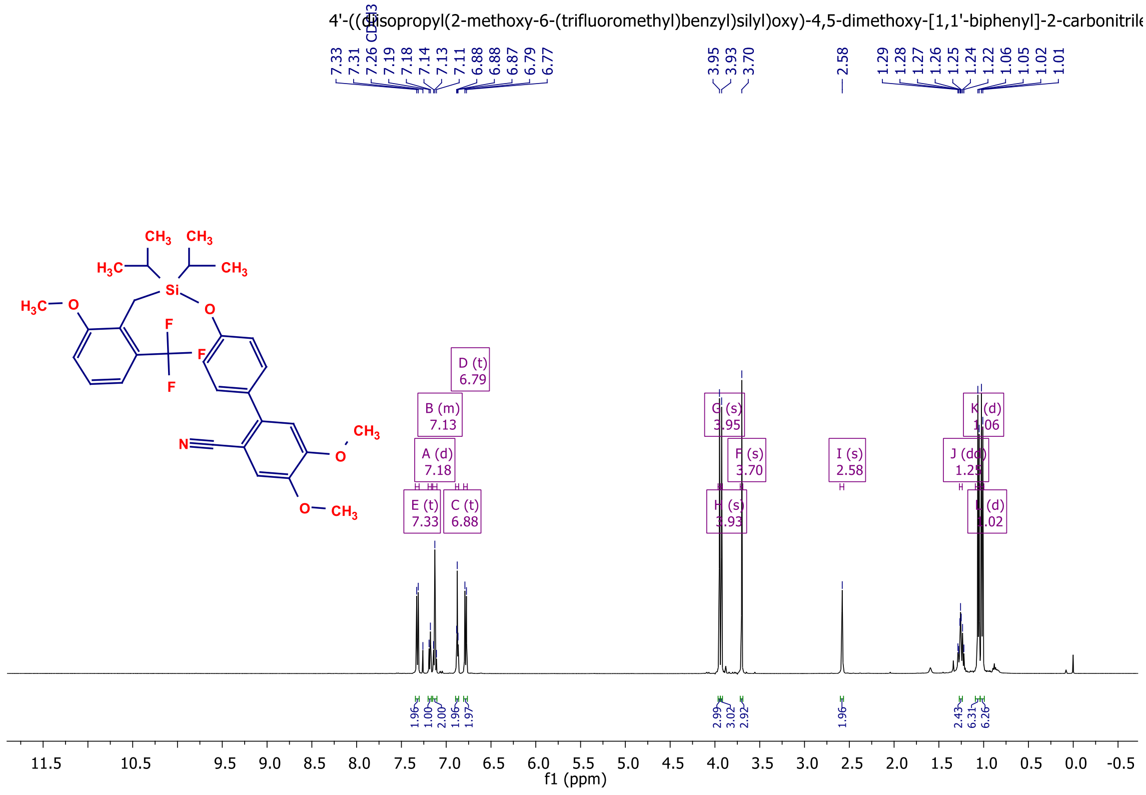

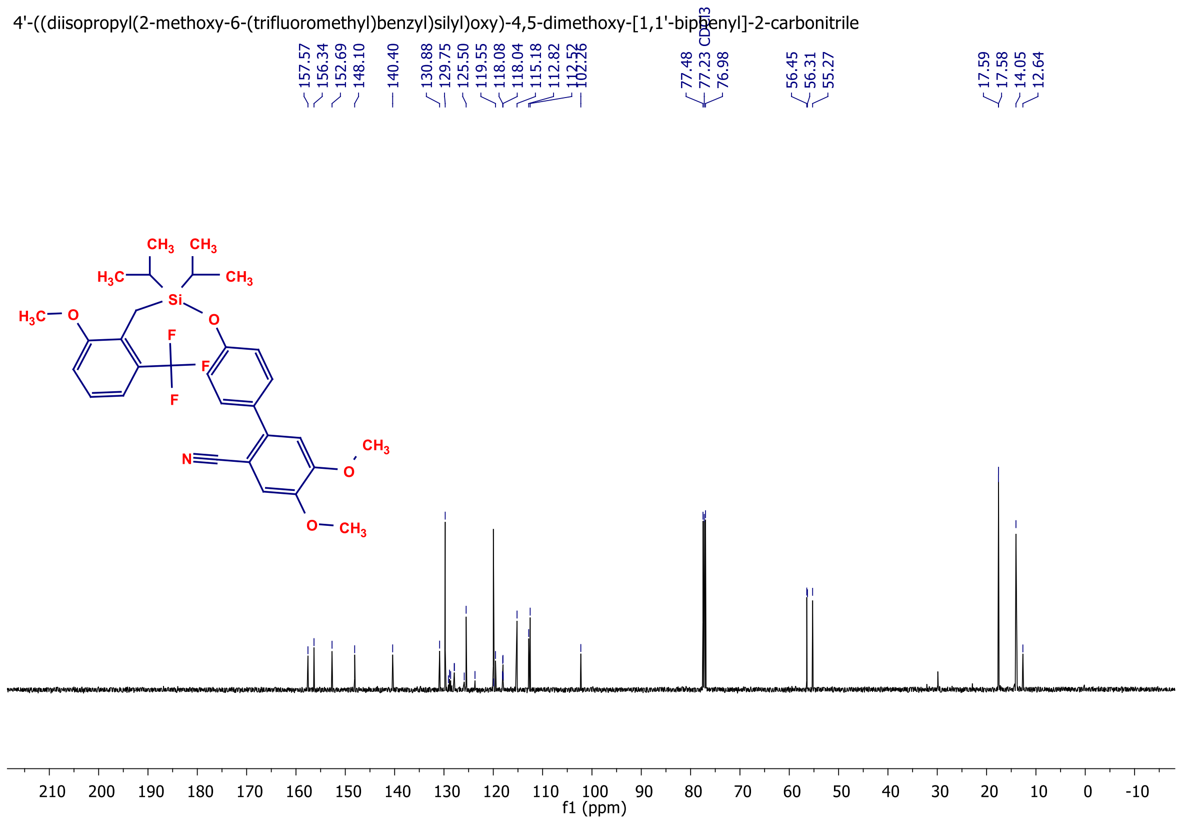

Supplementary Figure 16: ^{1}H and ^{13}C NMR (500 MHz, $CDCl_3$) of 4'-(((5-(difluoromethoxy)-2-fluorobenzyl)diisopropylsilyl)oxy)-4,5-dimethoxy-[1,1'-biphenyl]-2-carbonitrile:

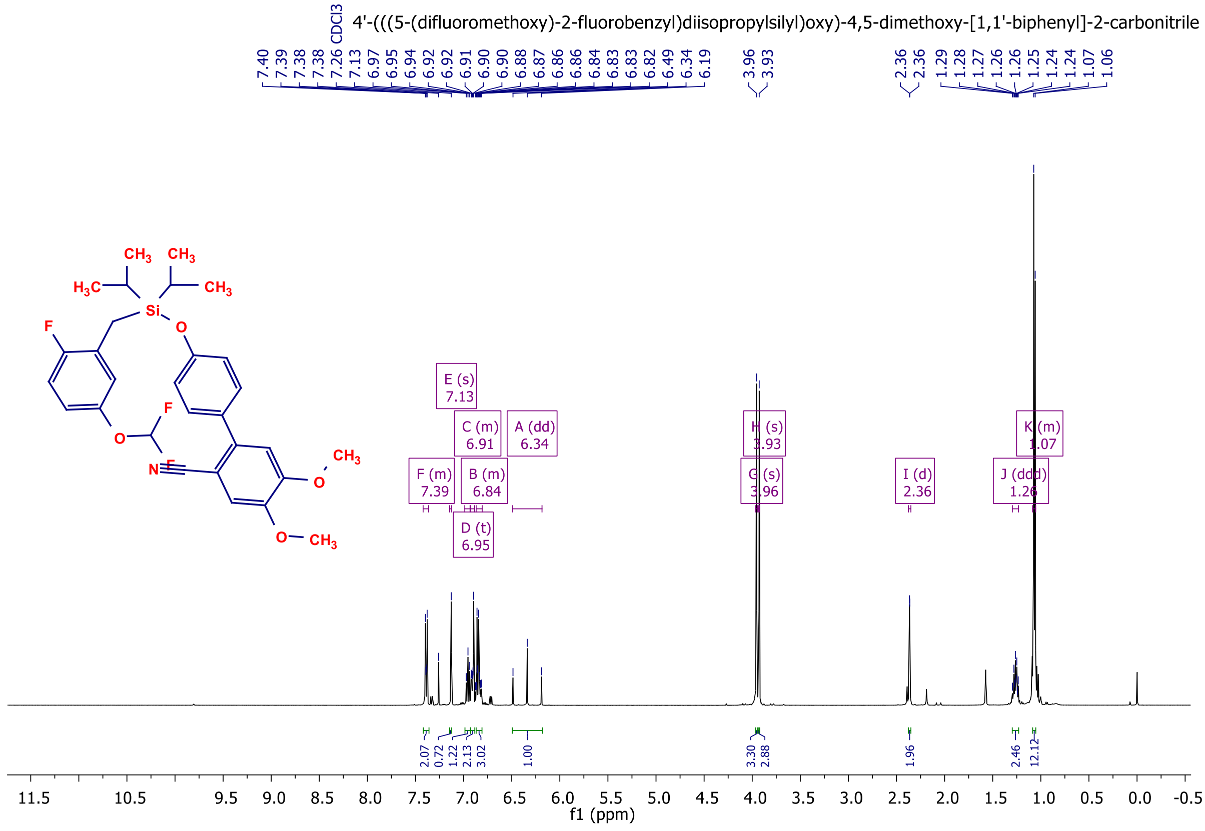

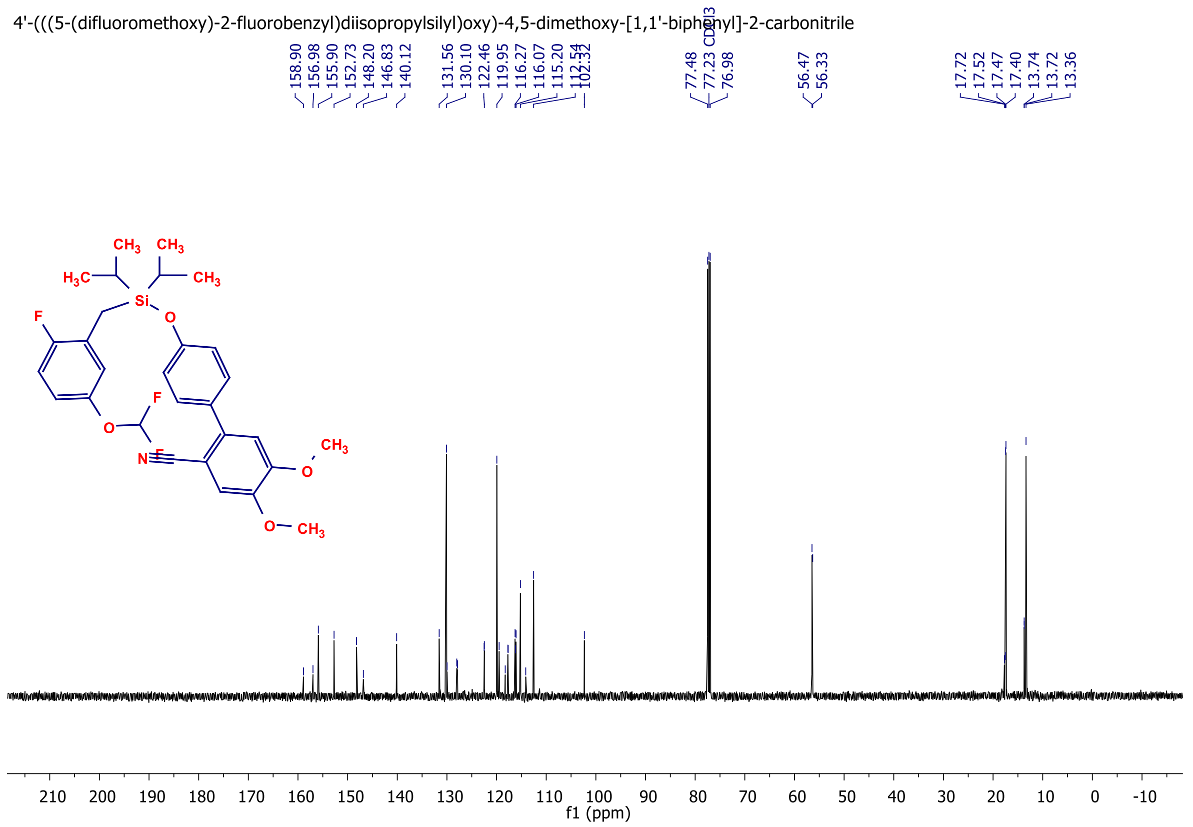

Supplementary Figure 17: ^{1}H and ^{13}C NMR (500 MHz, $CDCl_3$) of 4'-(((3-fluoro-2-methylbenzyl)diisopropylsilyl)oxy)-4,5-dimethoxy-[1,1'-biphenyl]-2-carbonitrile:

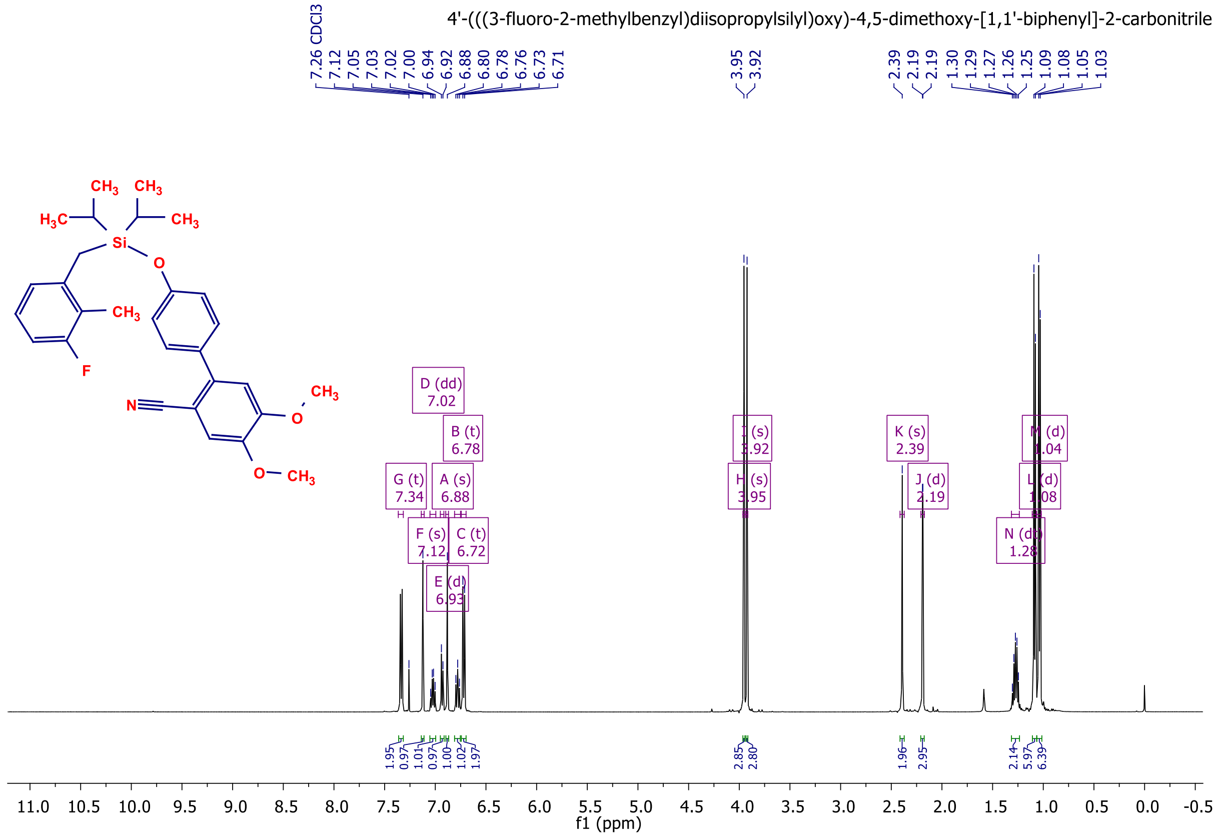

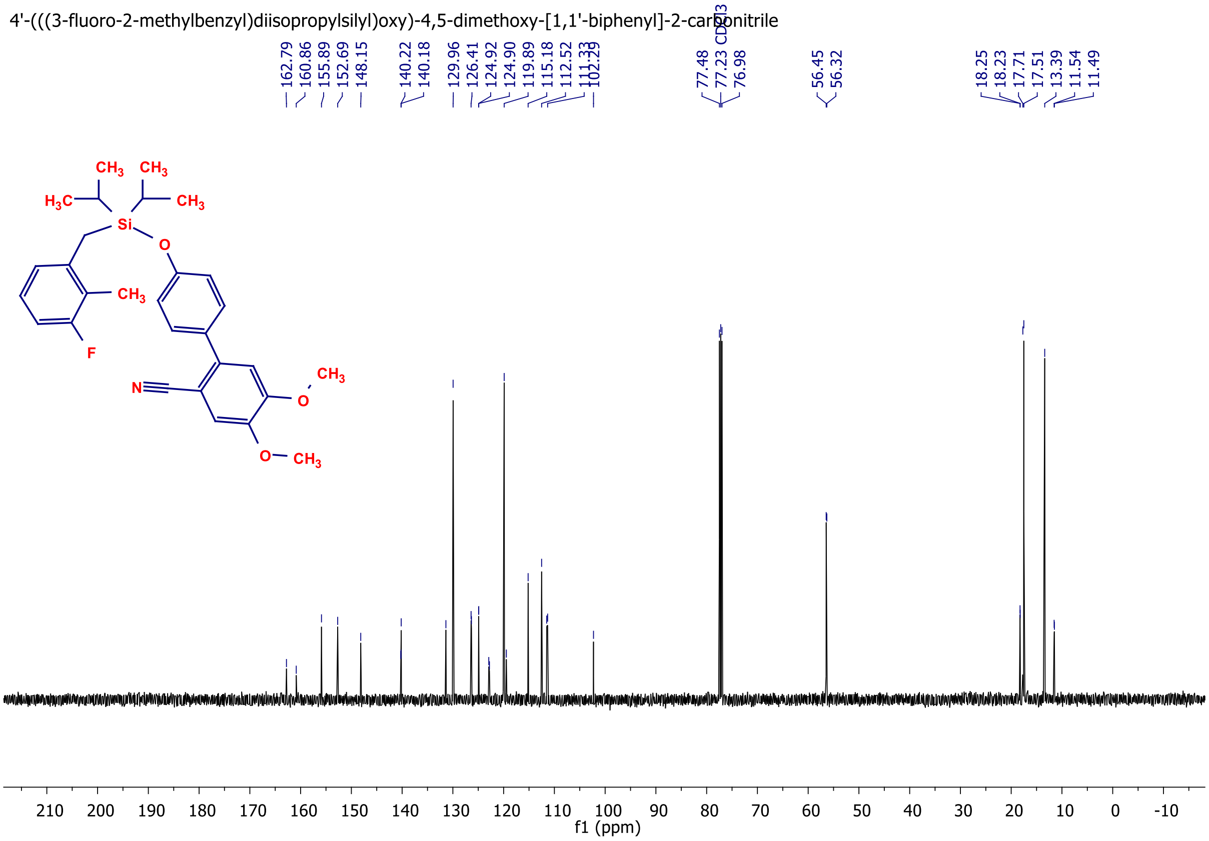

Supplementary Figure 18: ^{1}H and ^{13}C NMR (500 MHz, $CDCl_3$) of 4'-(((2-(difluoromethoxy)benzyl)diisopropylsilyl)oxy)-4,5-dimethoxy-[1,1'-biphenyl]-2-carbonitrile:

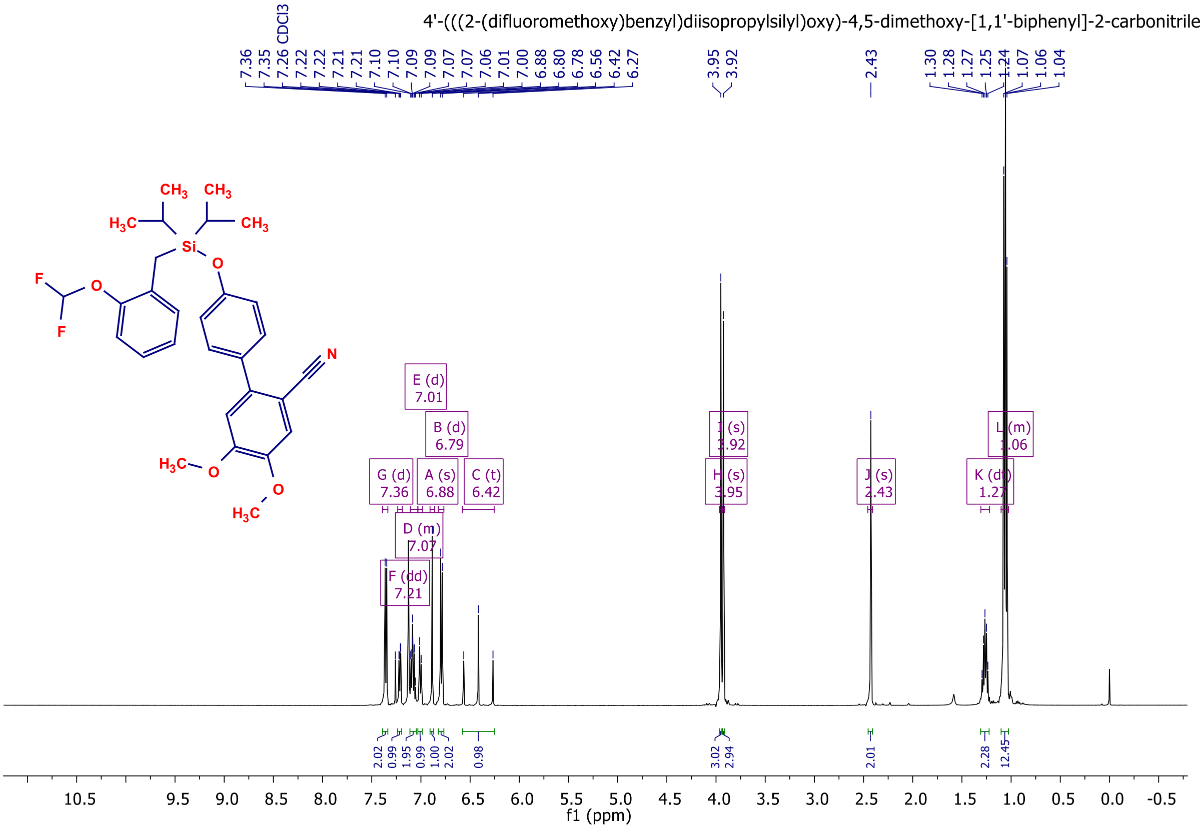

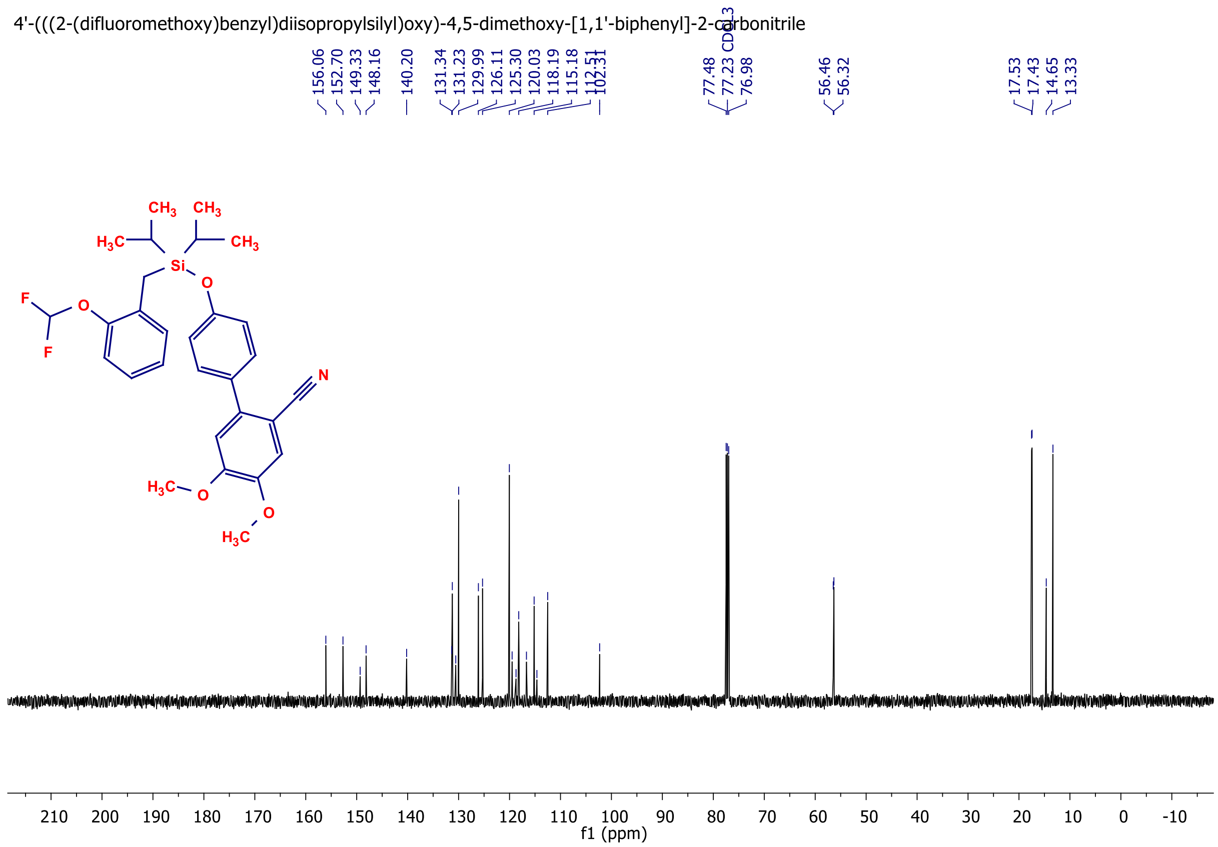

Supplementary Figure 19: ^{1}H and ^{13}C NMR (500 MHz, $CDCl_3$) of 4'-(((5-fluoro-2-methoxybenzyl)diisopropylsilyl)oxy)-4,5-dimethoxy-[1,1'-biphenyl]-2-carbonitrile:

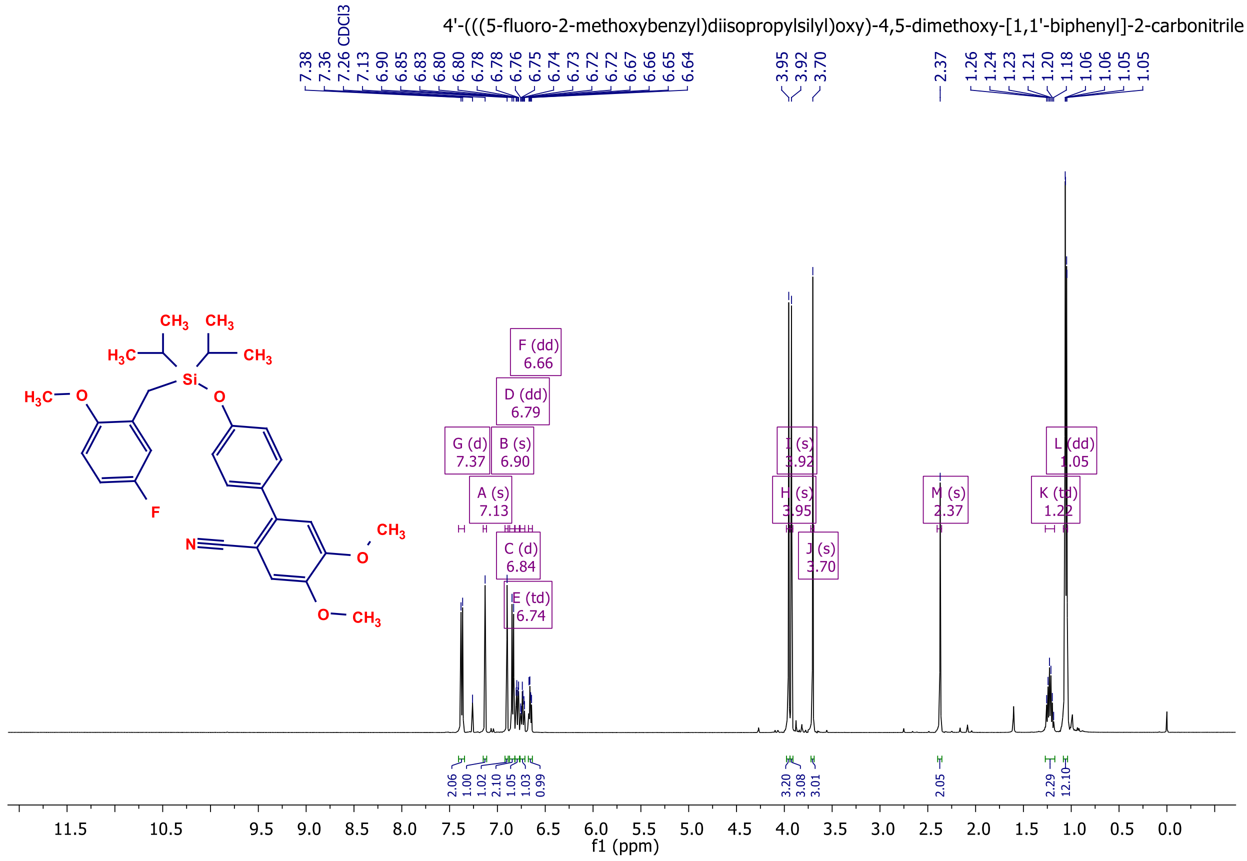

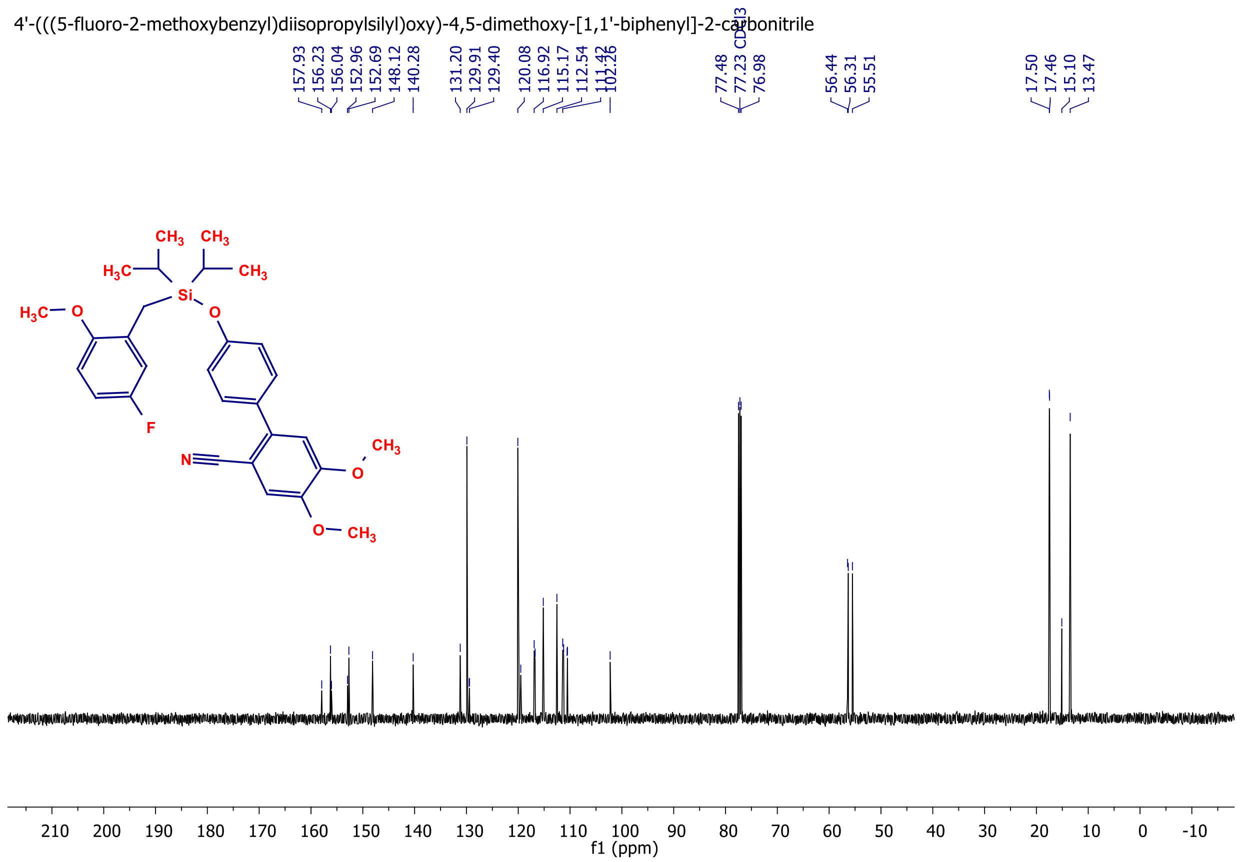

Supplementary Figure 20: ^{1}H and ^{13}C NMR (500 MHz, $CDCl_3$) of 4'-((diisopropyl(2-((trifluoromethyl)thio)benzyl)silyl)oxy)-4,5-dimethoxy-[1,1'-biphenyl]-2-carbonitrile:

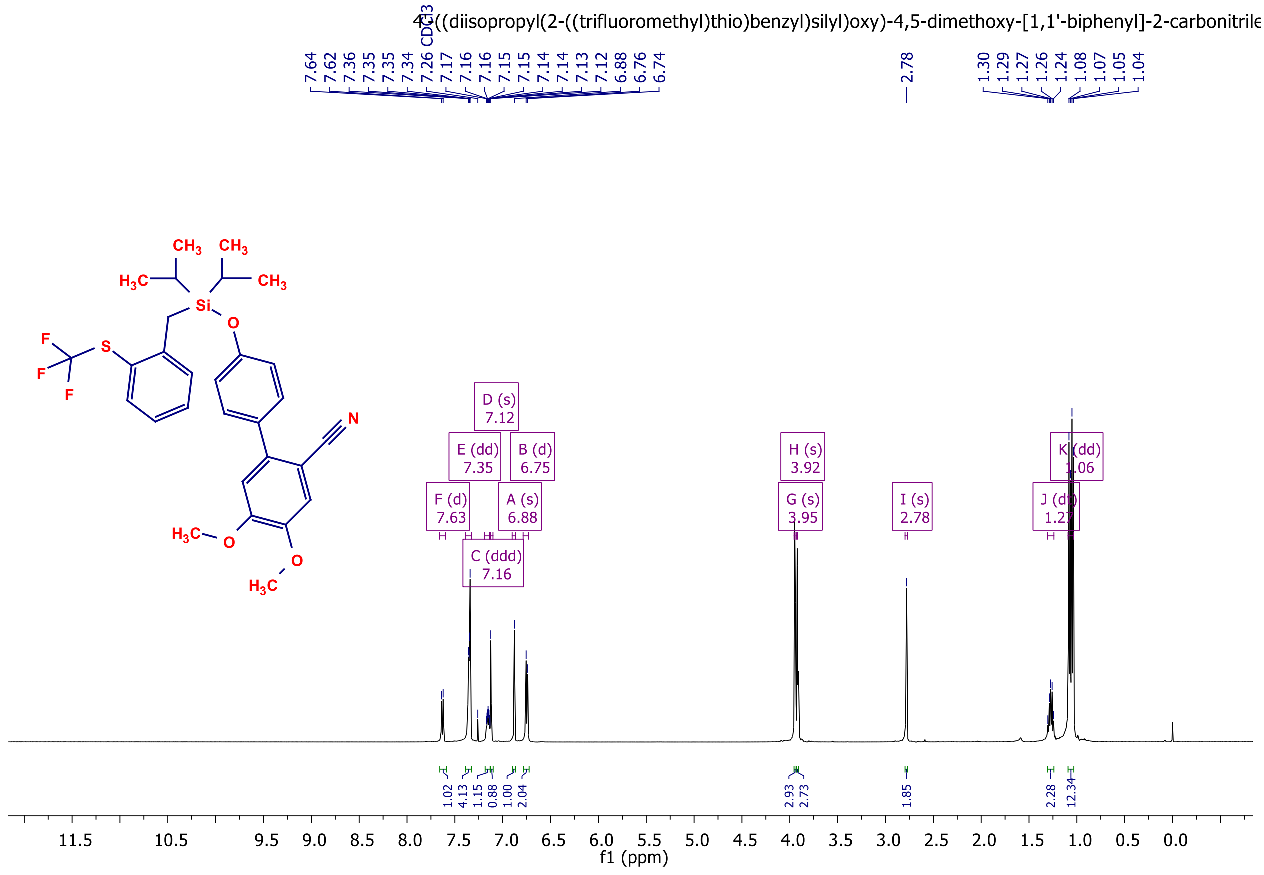

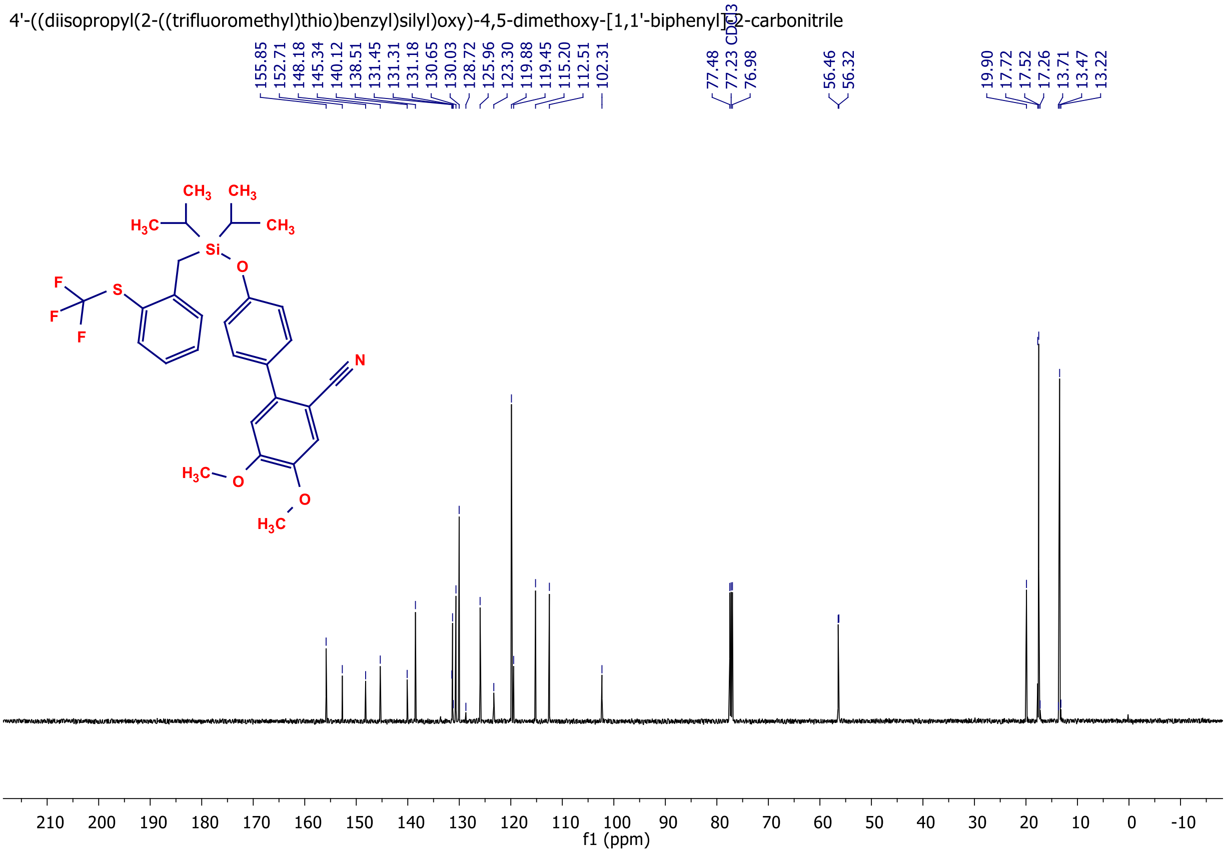

Supplementary Figure 21: ^{1}H and ^{13}C NMR (500 MHz, $CDCl_3$) of 4'-(((3,5-dimethylbenzyl)diisopropylsilyl)oxy)-4,5-dimethoxy-[1,1'-biphenyl]-2-carbonitrile:

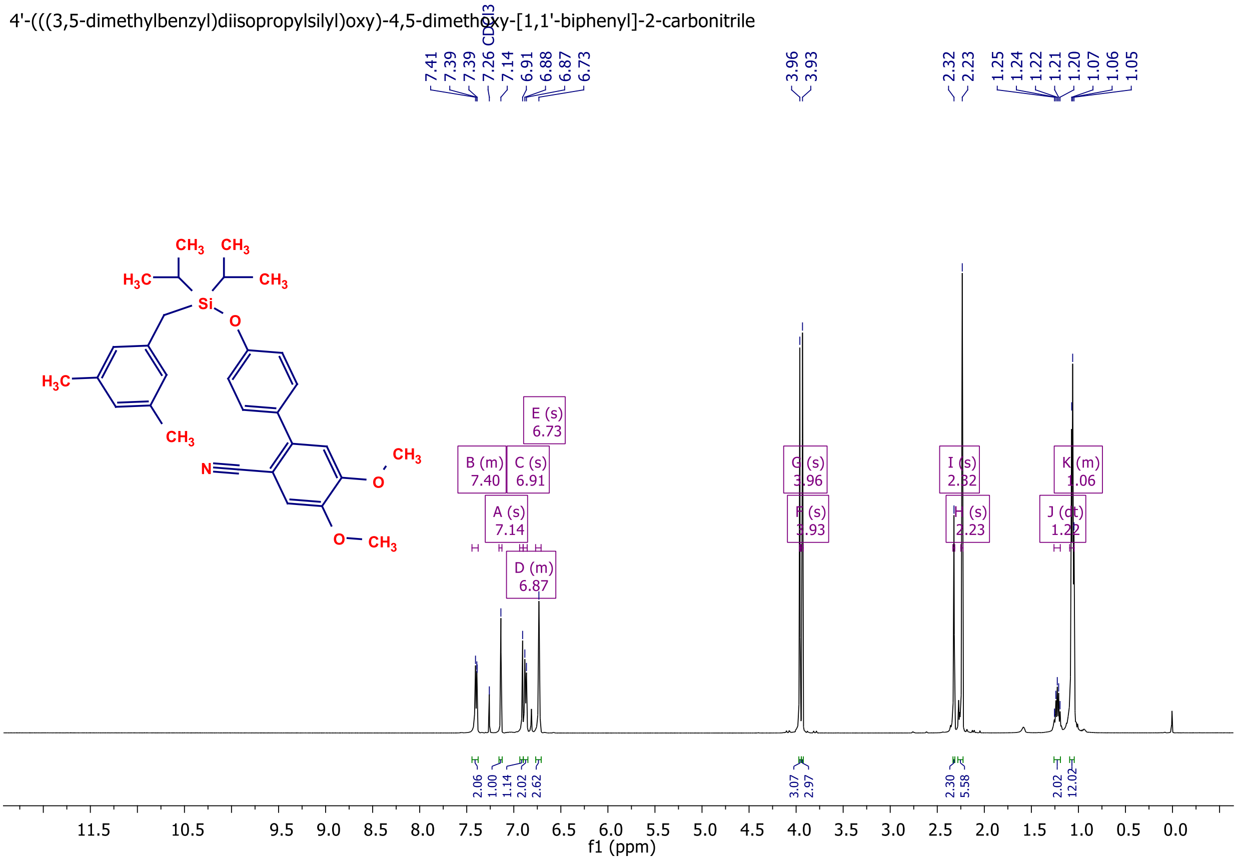

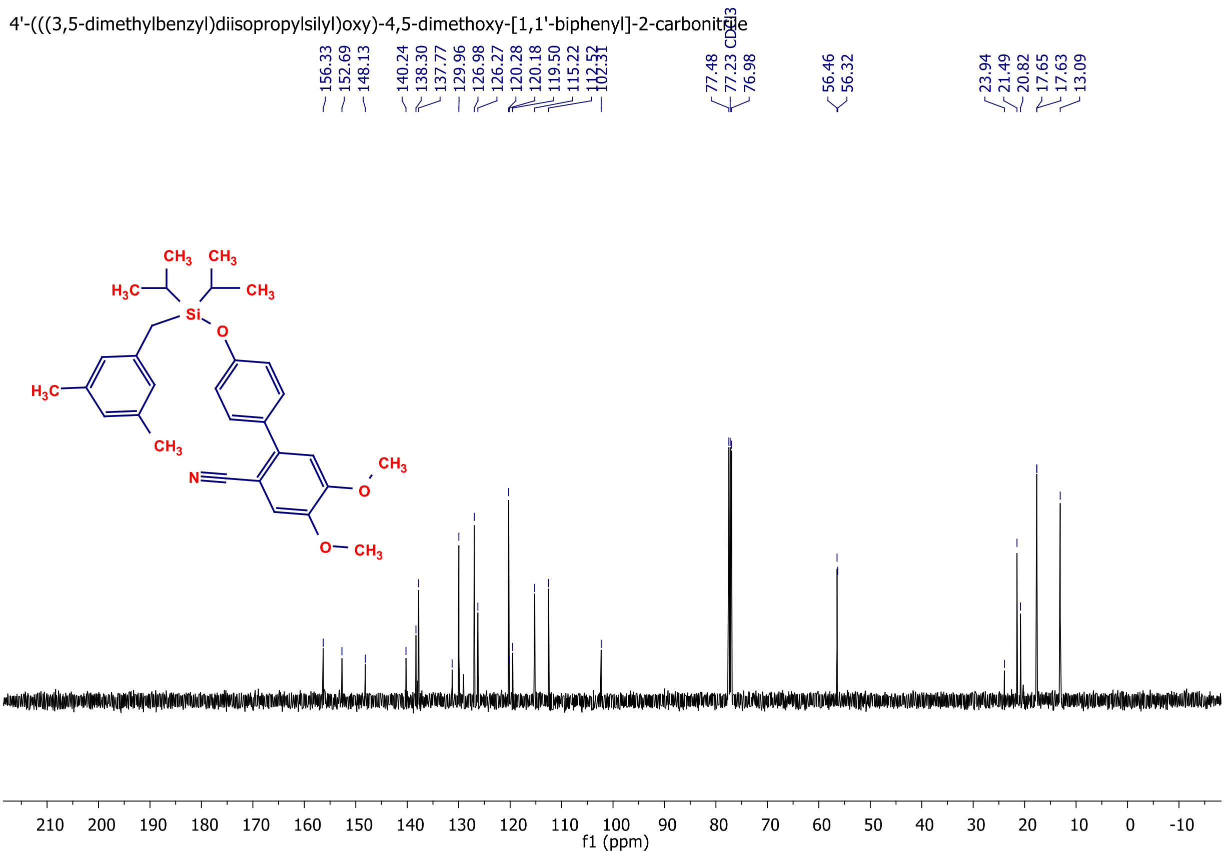

Supplementary Figure 22: ^{1}H and ^{13}C NMR (500 MHz, $CDCl_3$) of 4'-(((2-fluoro-6-(trifluoromethyl)benzyl)diisopropylsilyl)oxy)-4,5-dimethoxy-[1,1'-biphenyl]-2-carbonitrile:

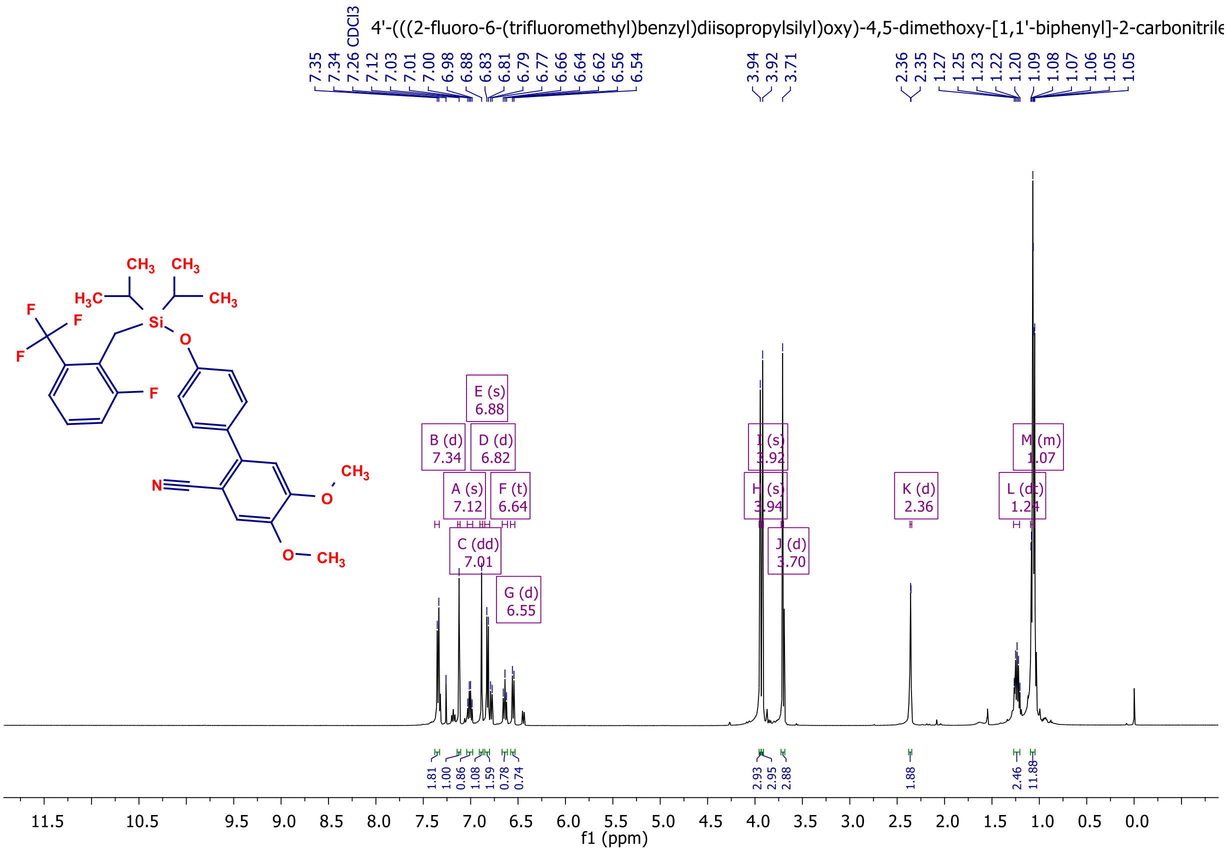

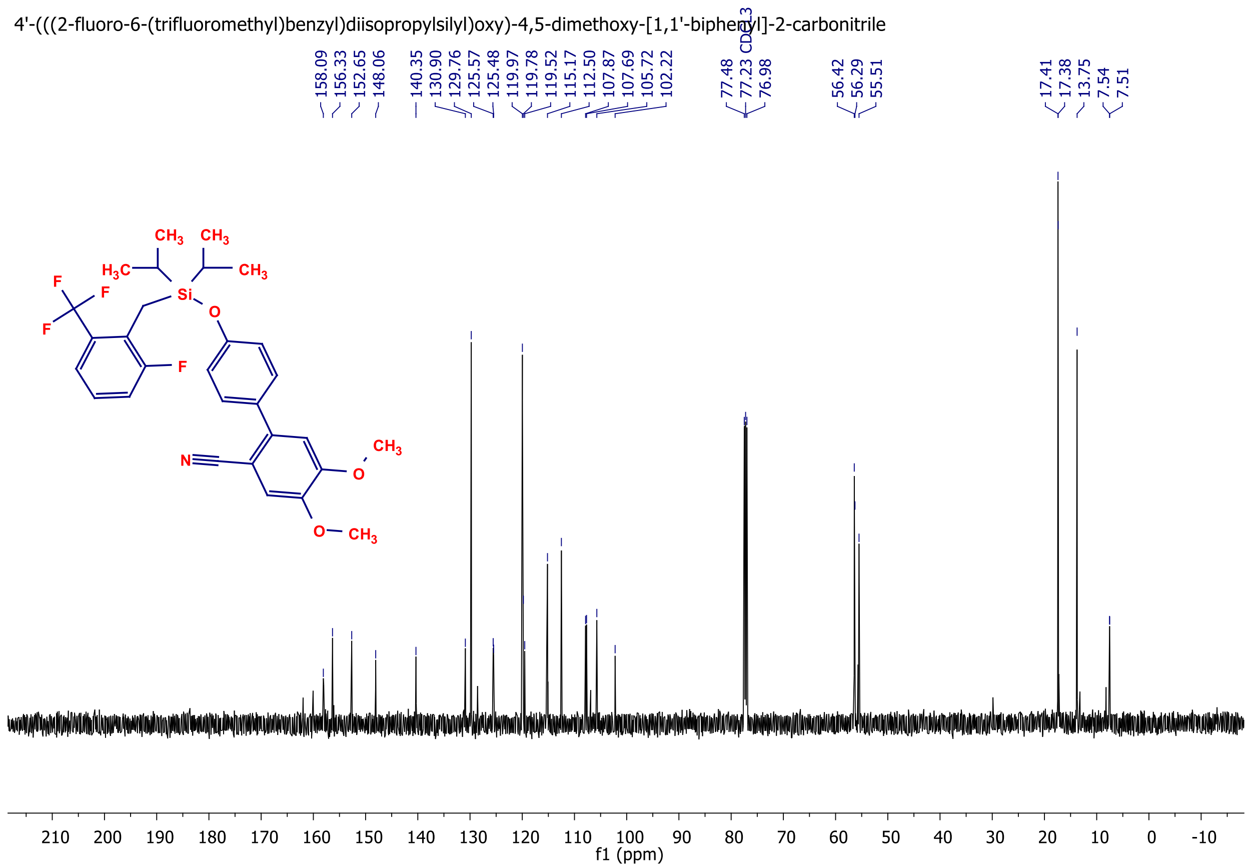

Supplementary Figure 23: ^{1}H and ^{13}C NMR (500 MHz, $CDCl_3$) of 4'-(((2-chloro-5-(difluoromethoxy)benzyl)diisopropylsilyl)oxy)-4,5-dimethoxy-[1,1'-biphenyl]-2-carbonitrile:

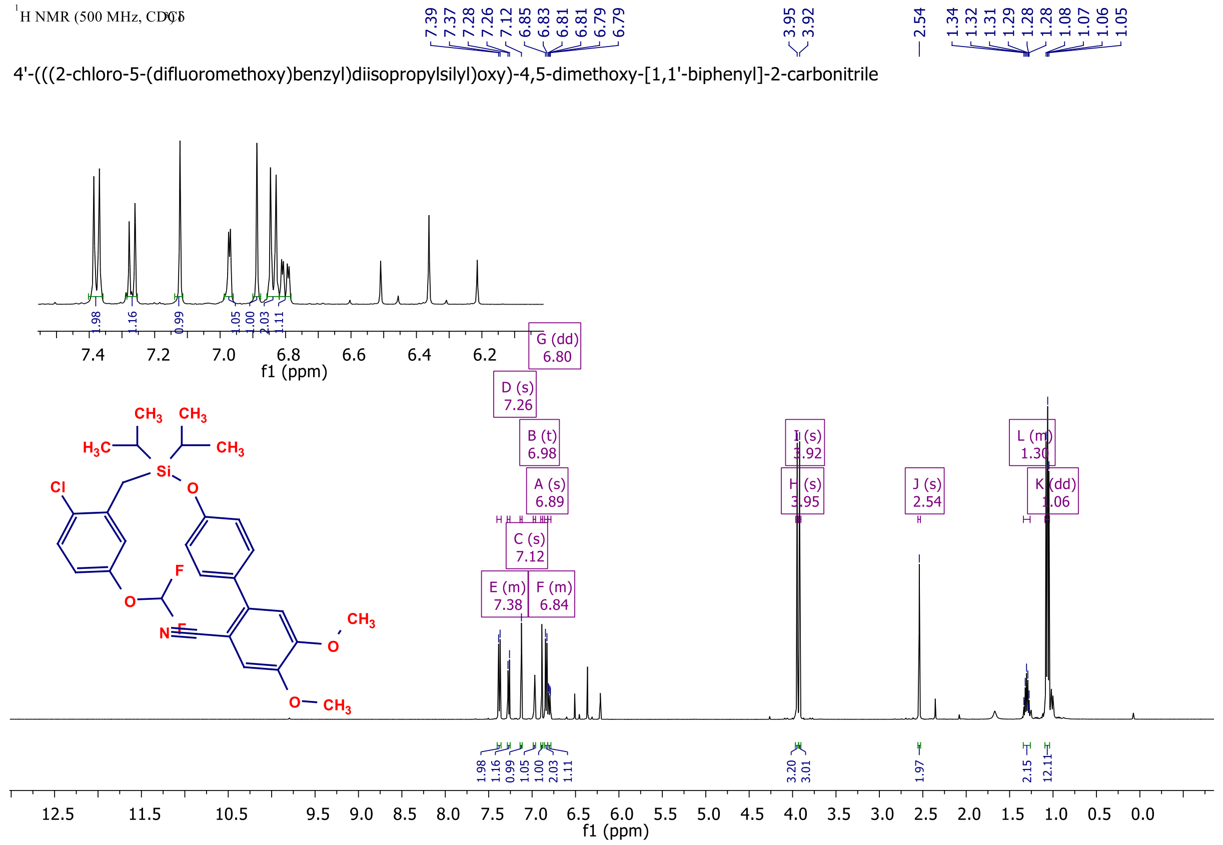

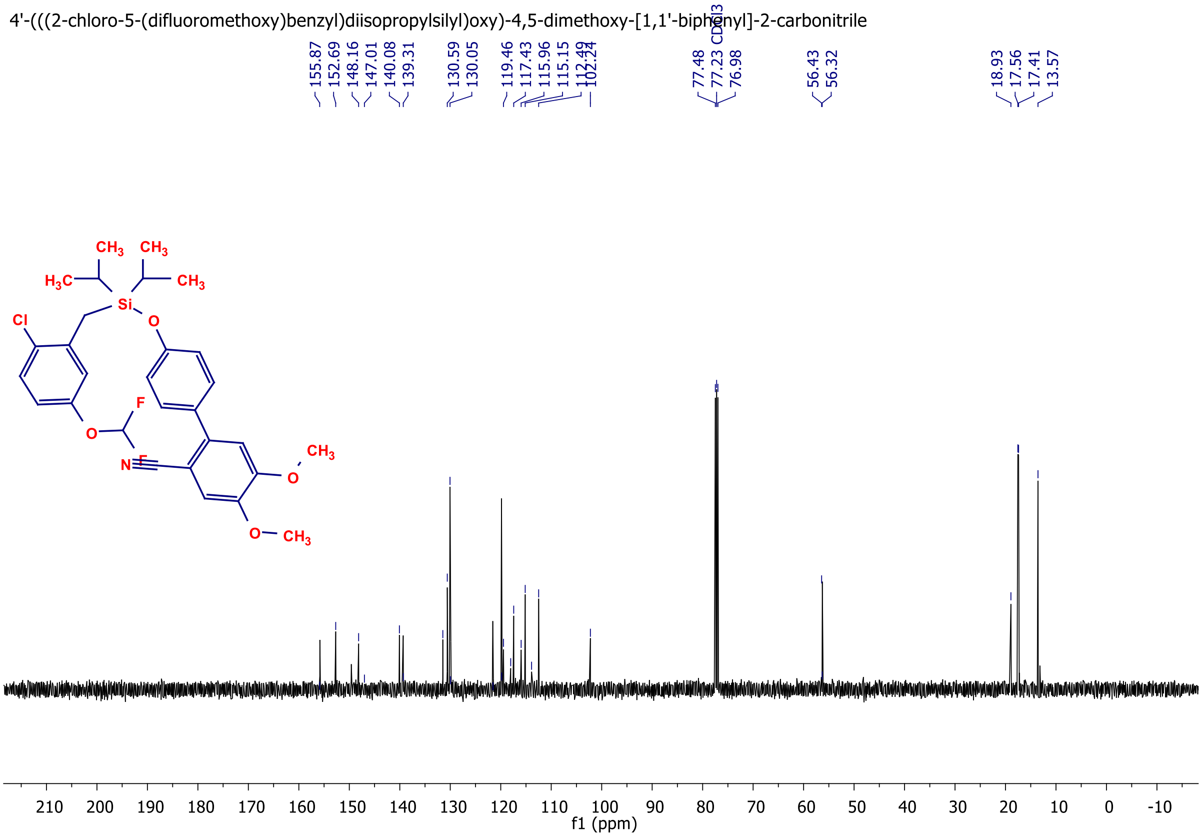

Supplementary Figure 24: ^{1}H and ^{13}C NMR (500 MHz, $CDCl_3$) of 4'-(((5-fluoro-2-(trifluoromethoxy)benzyl)diisopropylsilyl)oxy)-4,5-dimethoxy-[1,1'-biphenyl]-2-carbonitrile:

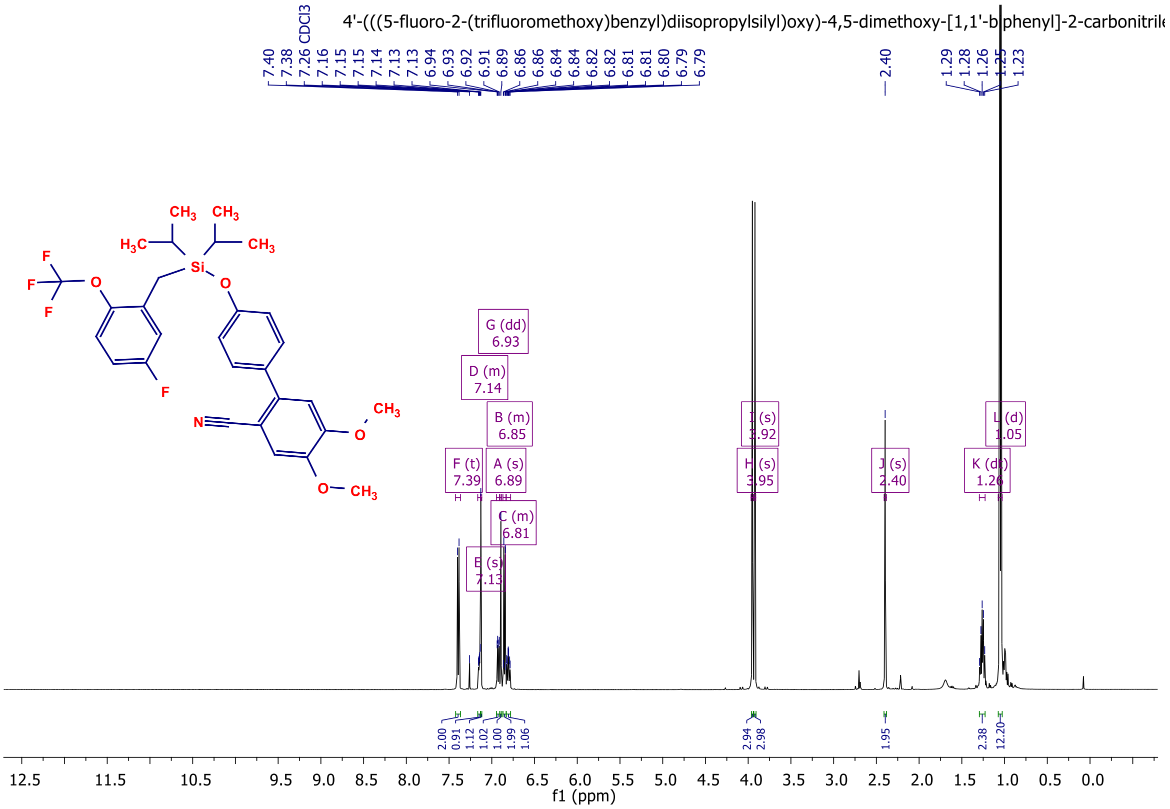

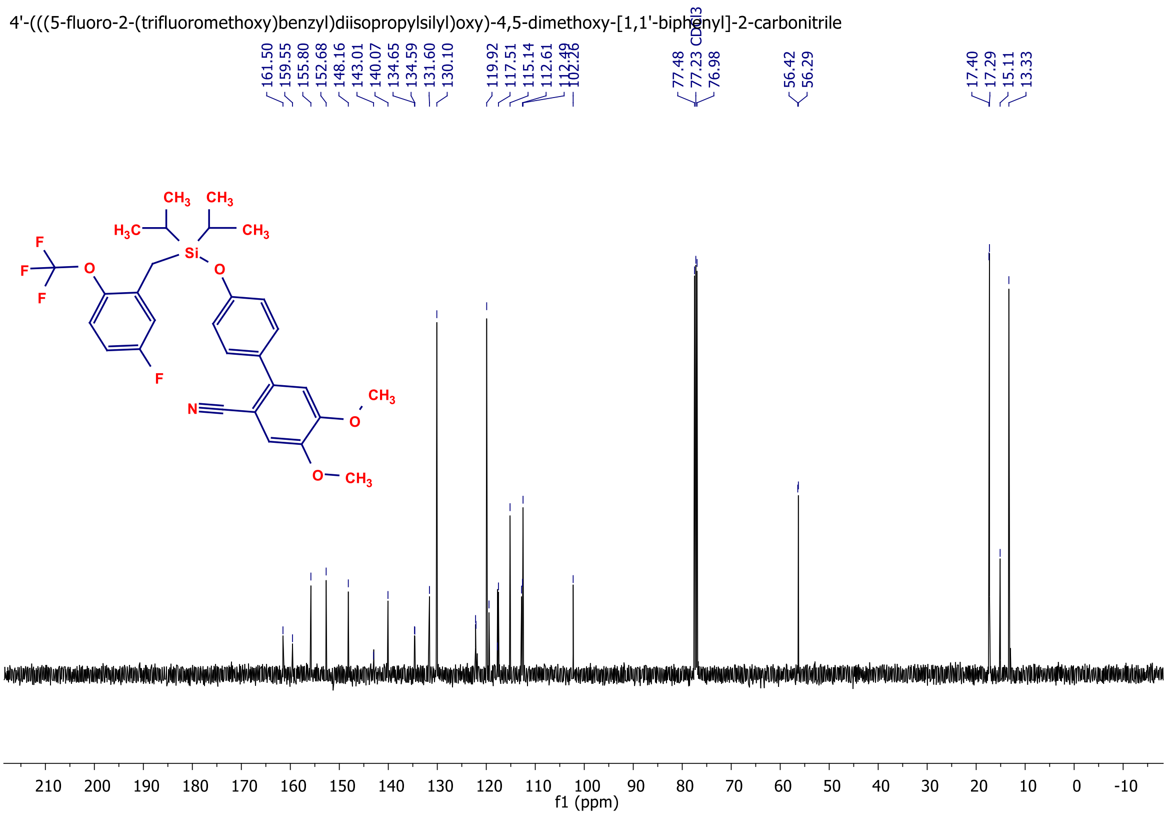

Vinyl Ether:

Supplementary Figure 25: ^{1}H and ^{13}C NMR (500 MHz, $CDCl_3$) of (E)-1-(2-ethoxyvinyl)-2,4-dimethylbenzene

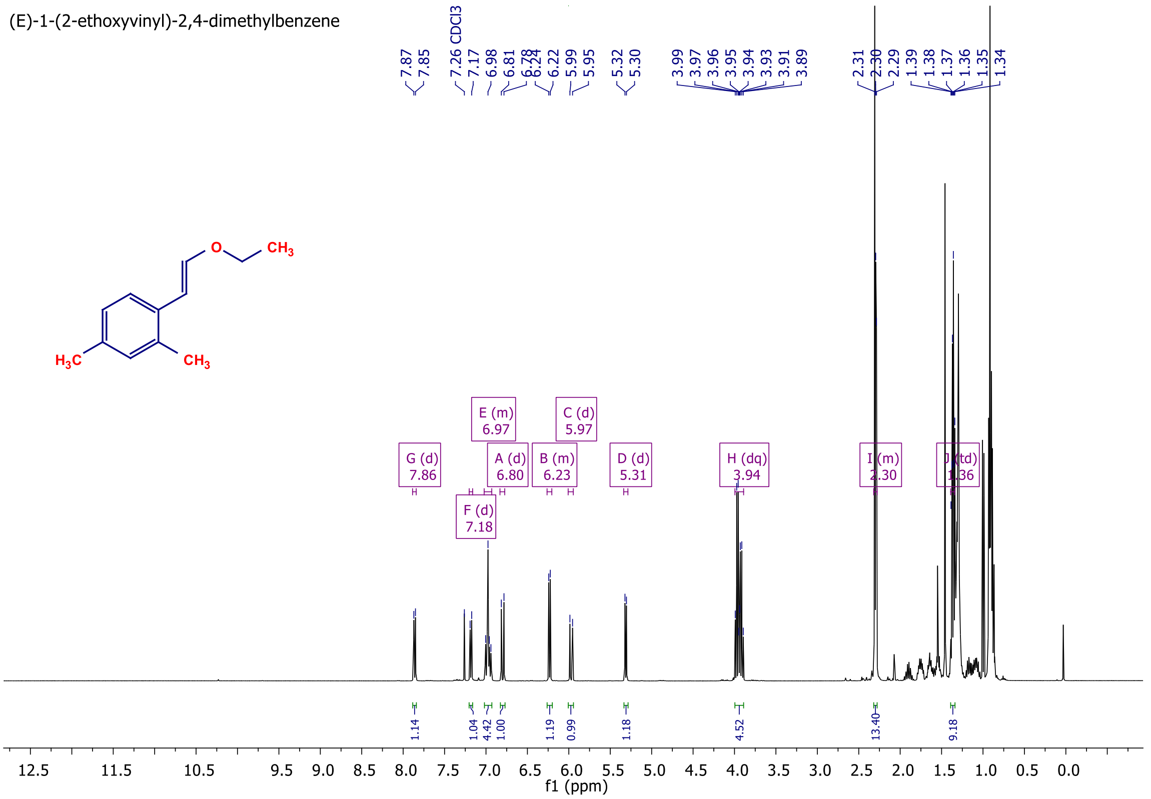

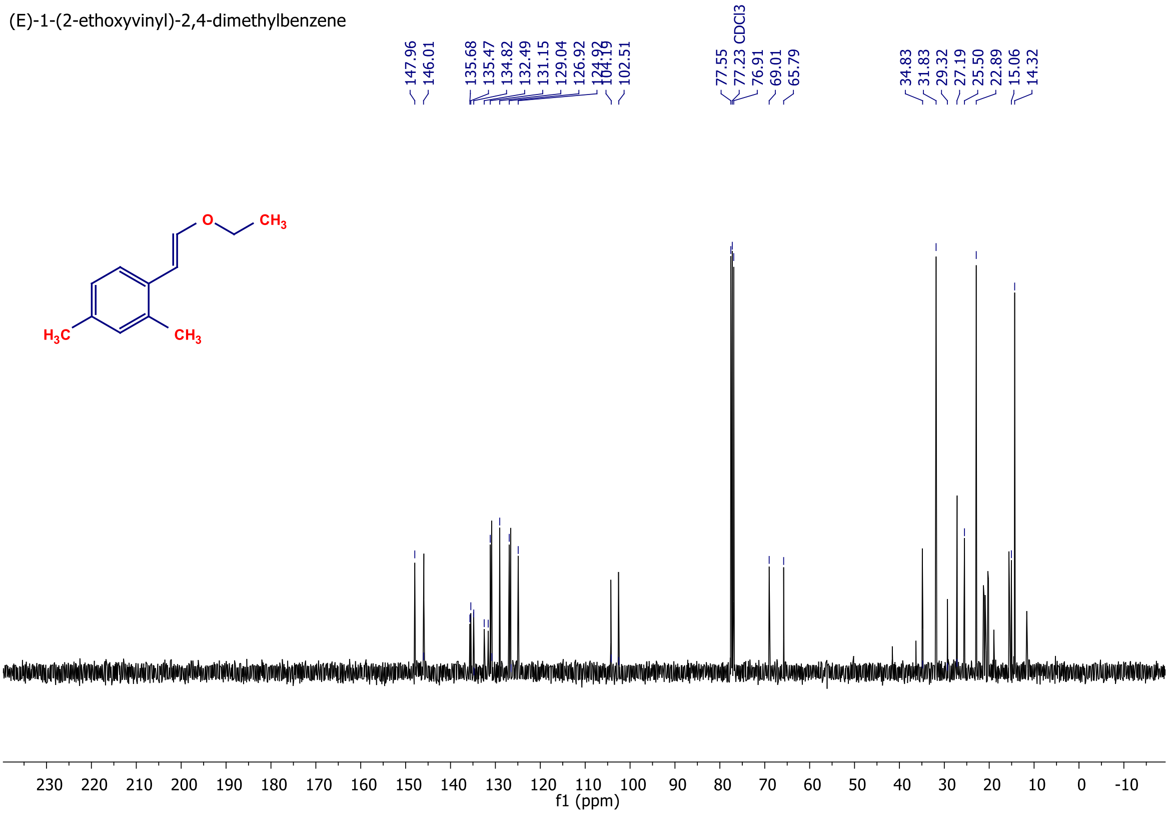

Supplementary Figure 26: ^{1}H NMR (500 MHz, $CDCl_3$) of (E)-1-chloro-2-(2-ethoxyvinyl)benzene:

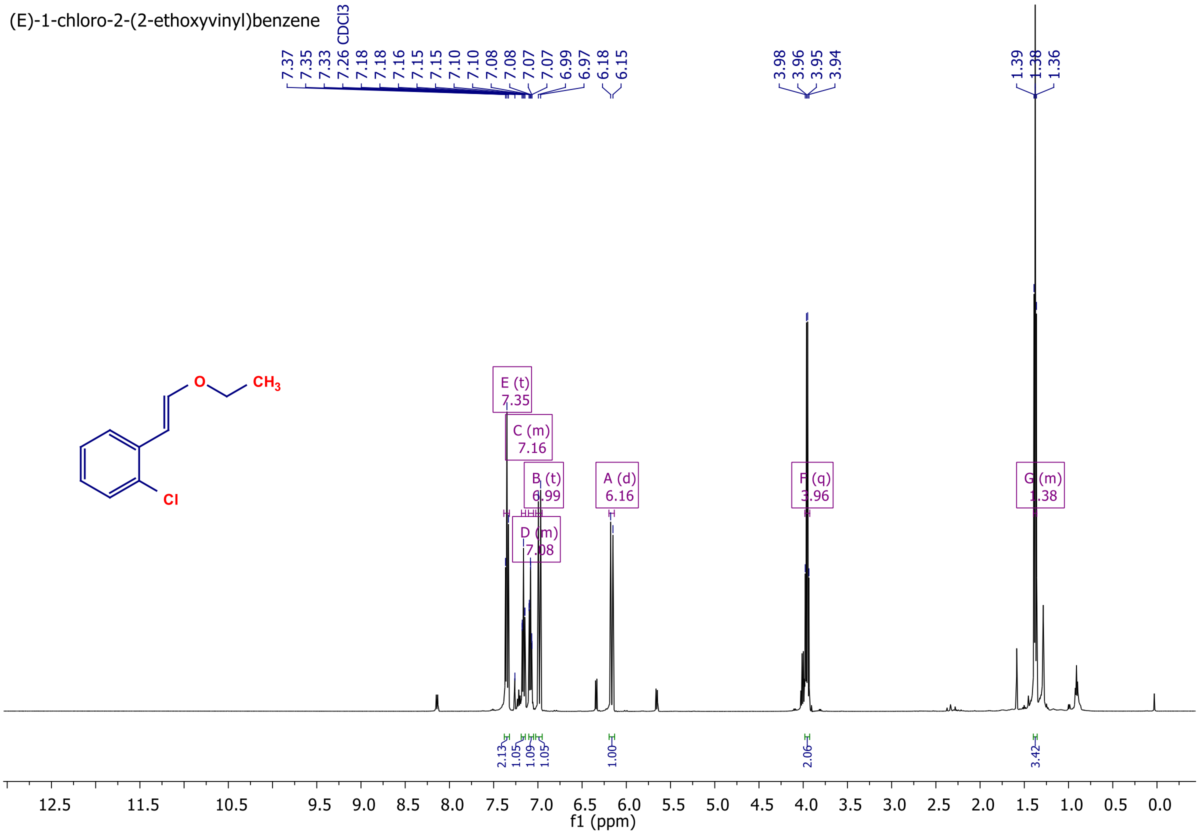

Supplementary Figure 27: ^{1}H and ^{13}C NMR (500 MHz, $CDCl_3$) of (E)-1-(2-ethoxyvinyl)-2-methylbenzene

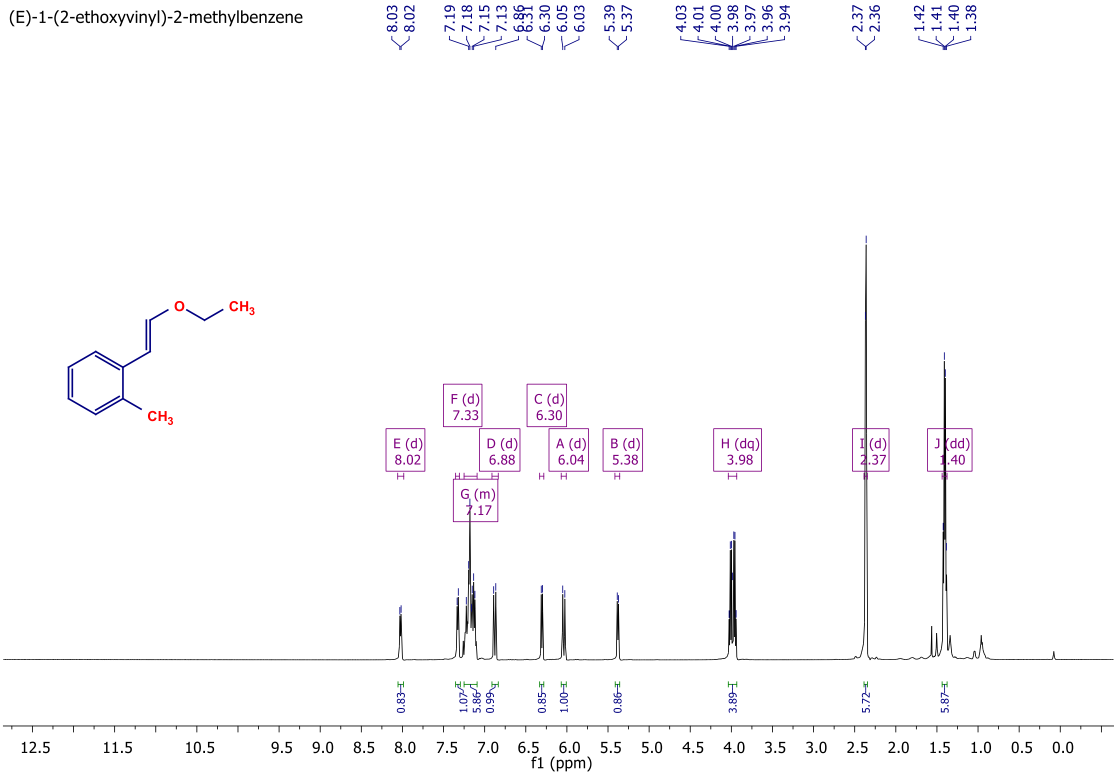

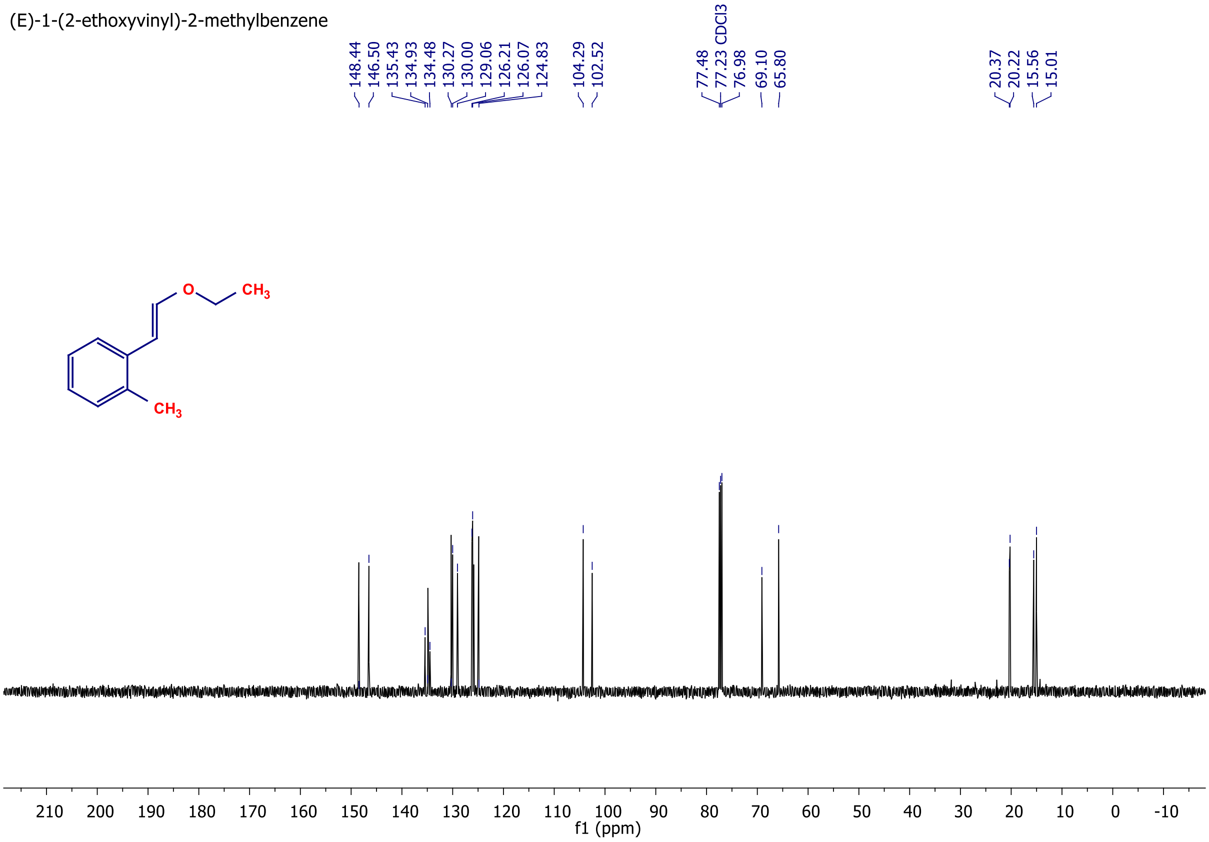

Supplementary Figure 28: ^{1}H and ^{13}C NMR (400 MHz, $CDCl_3$) of (E)-1,2-dichloro-4-(2-ethoxyvinyl)benzene:

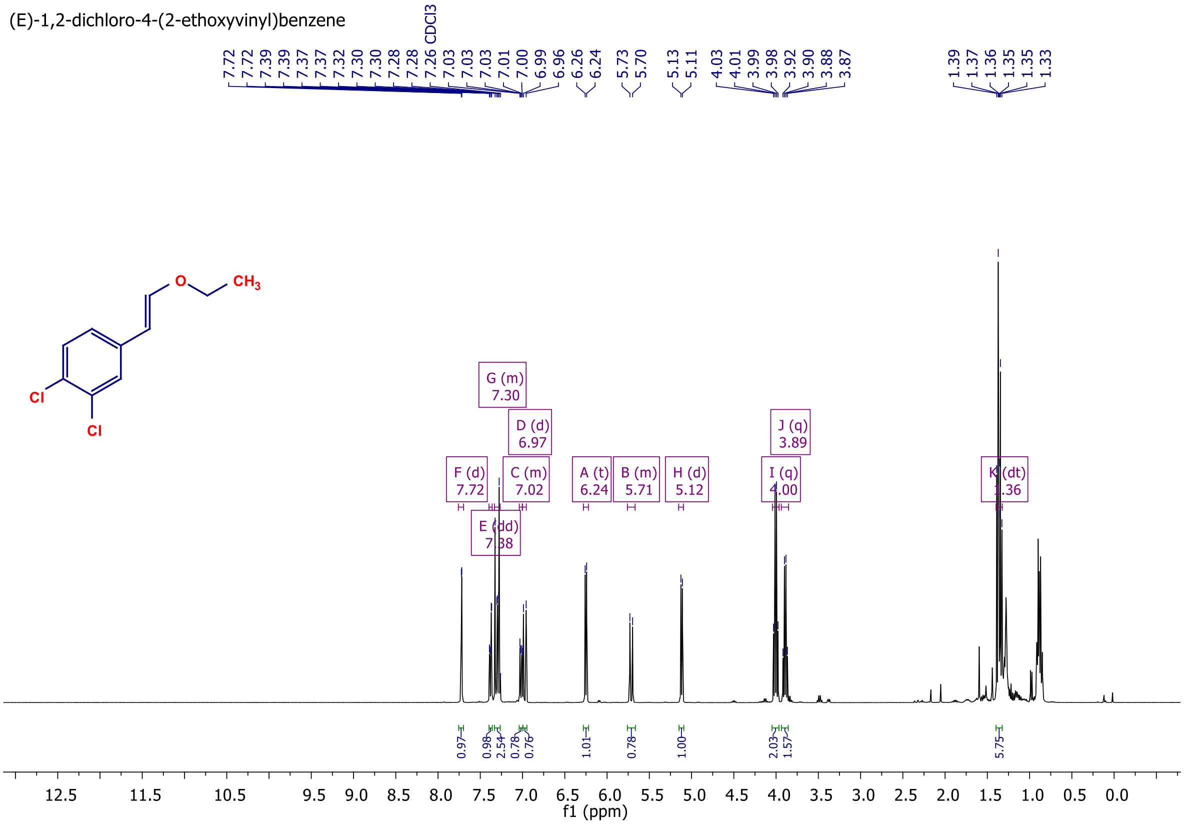

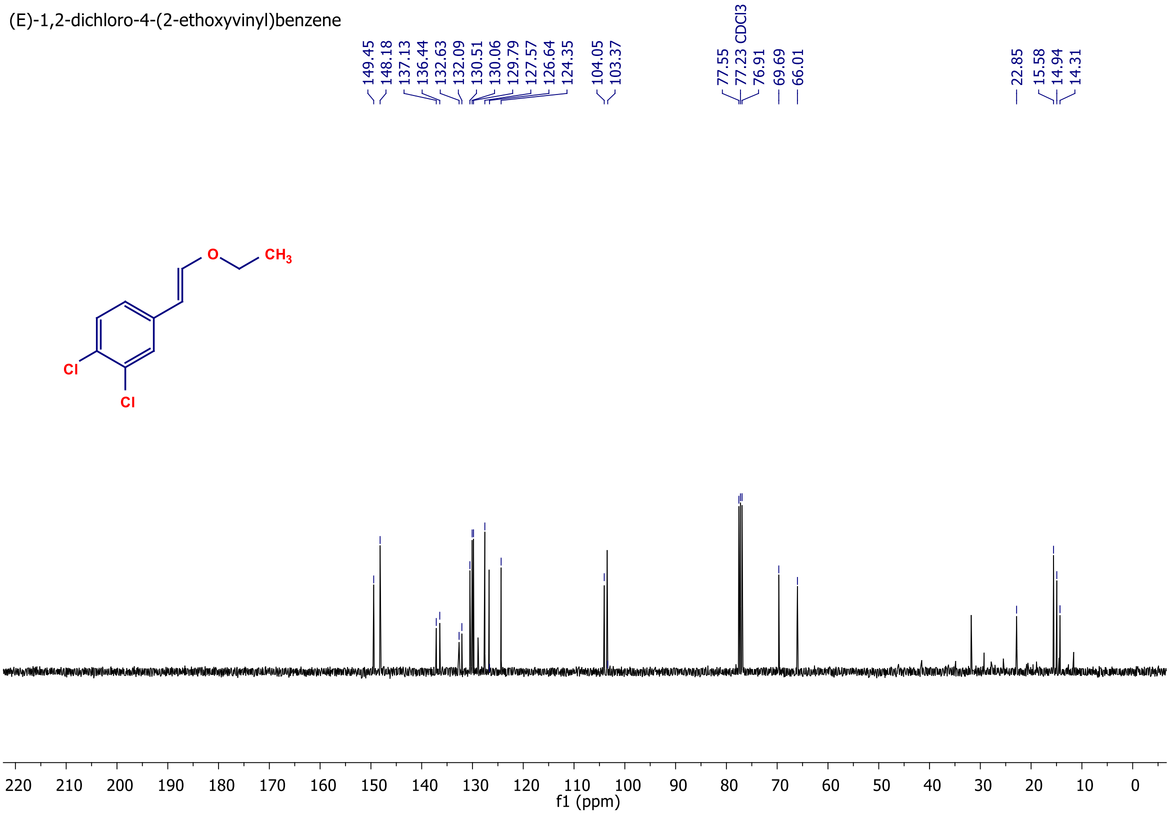

Supplementary Figure 29: ^{1}H and ^{13}C NMR (500 MHz, $CDCl_3$) of (E)-1-chloro-4-(2-ethoxyvinyl)benzene:

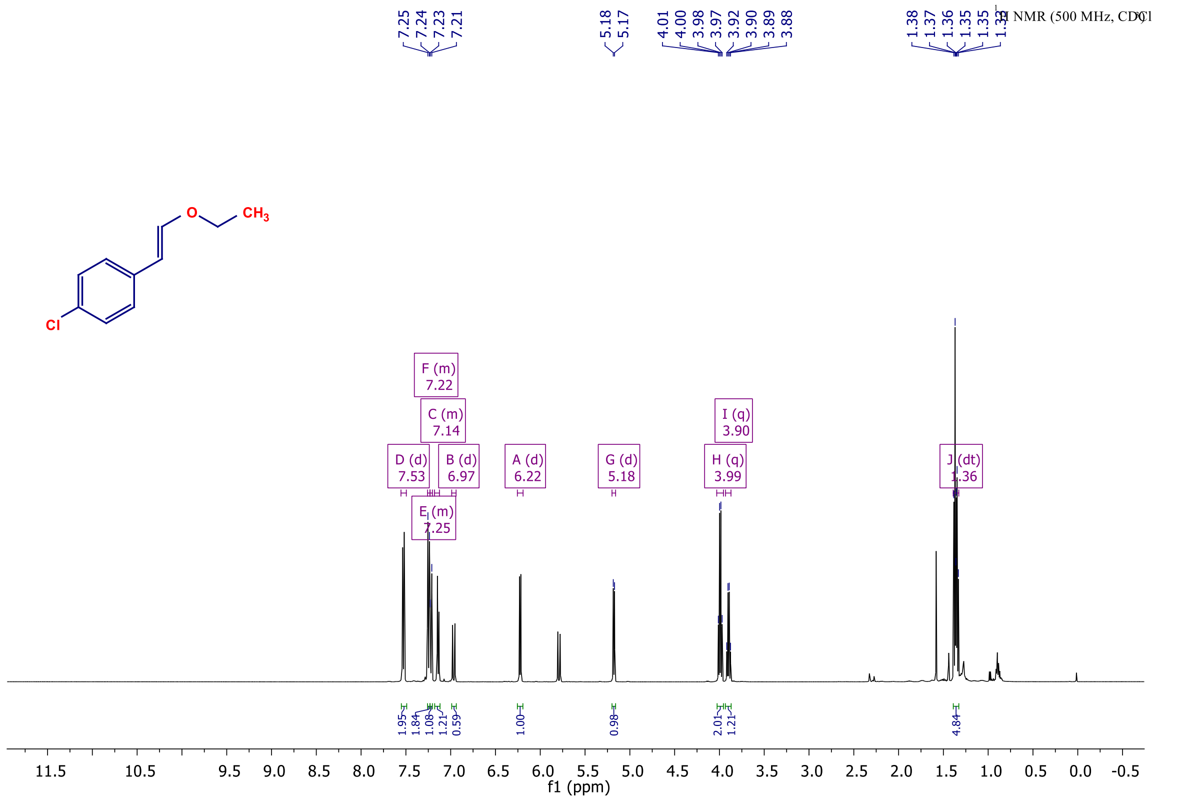

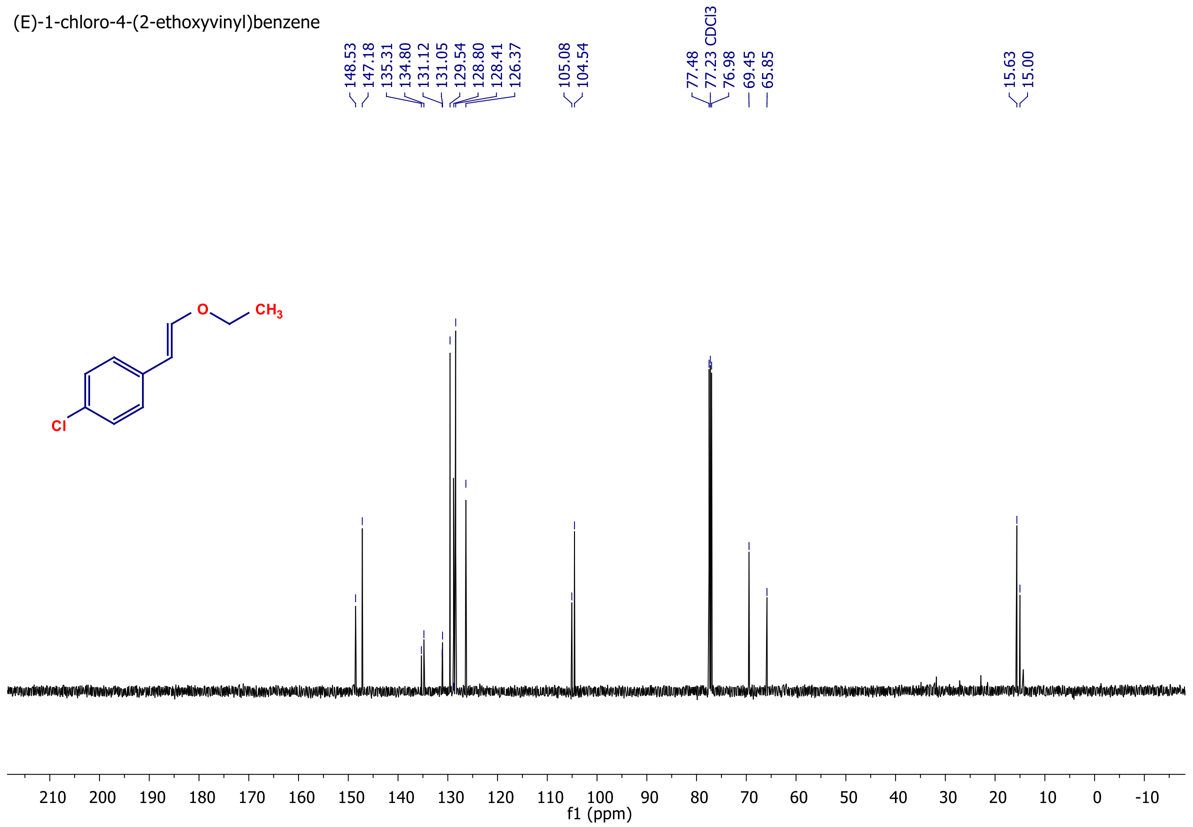

Product Acylation:

Supplementary Figure 30: ^{1}H and ^{13}C NMR (500 MHz, $CDCl_3$) of 4'-((4-acetylbenzyl)diisopropylsilyloxy)-4,5-dimethoxybiphenyl-2-carbonitrile (1a and 2a):

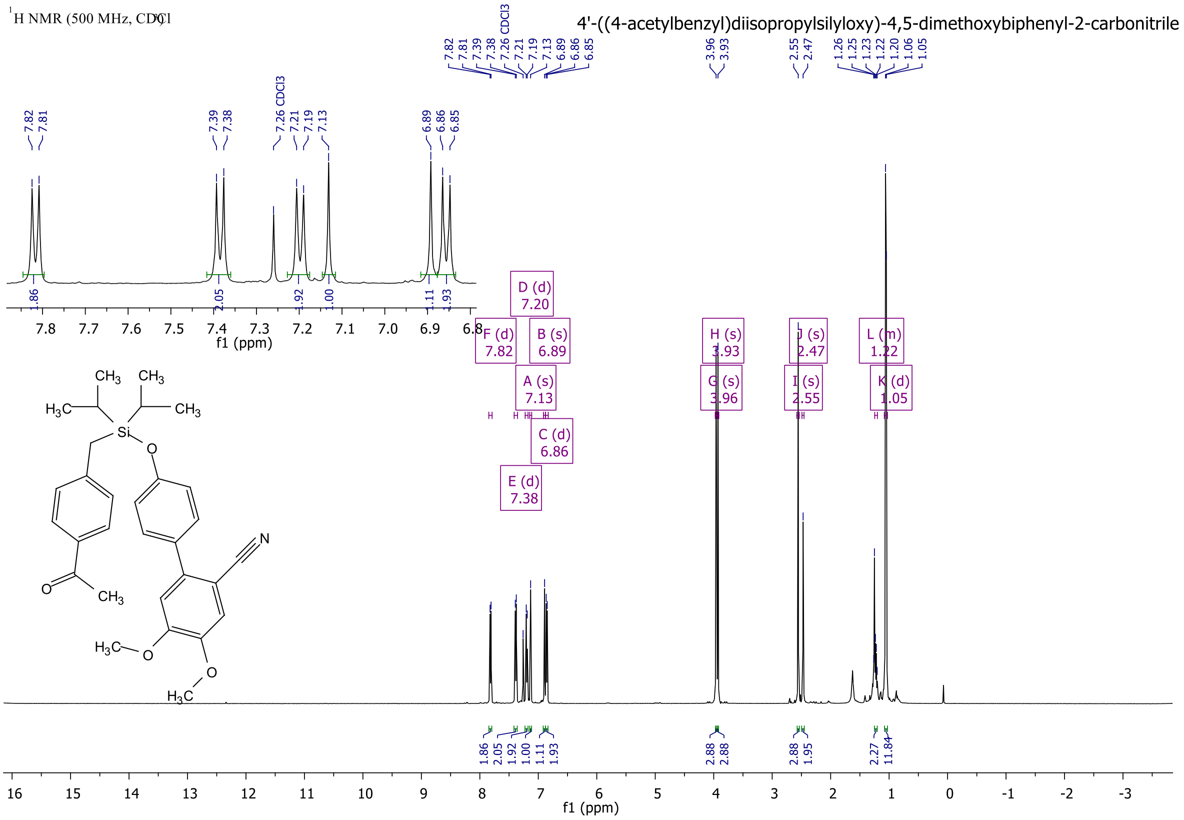

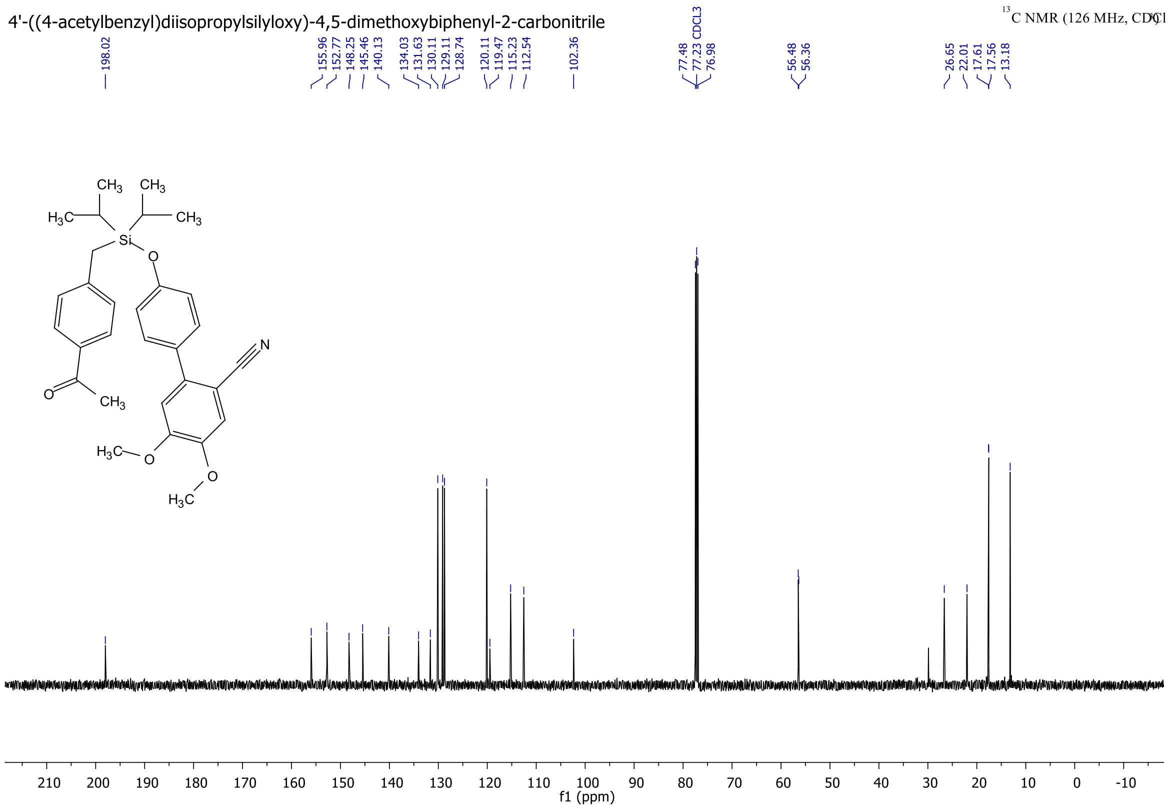

Supplementary Figure 31: ^{1}H and ^{13}C NMR (500 MHz, $CDCl_3$) of 4'-((4-acetyl-2-methylbenzyl)diisopropylsilyloxy)-4,5-dimethoxybiphenyl-2-carbonitrile (2b):

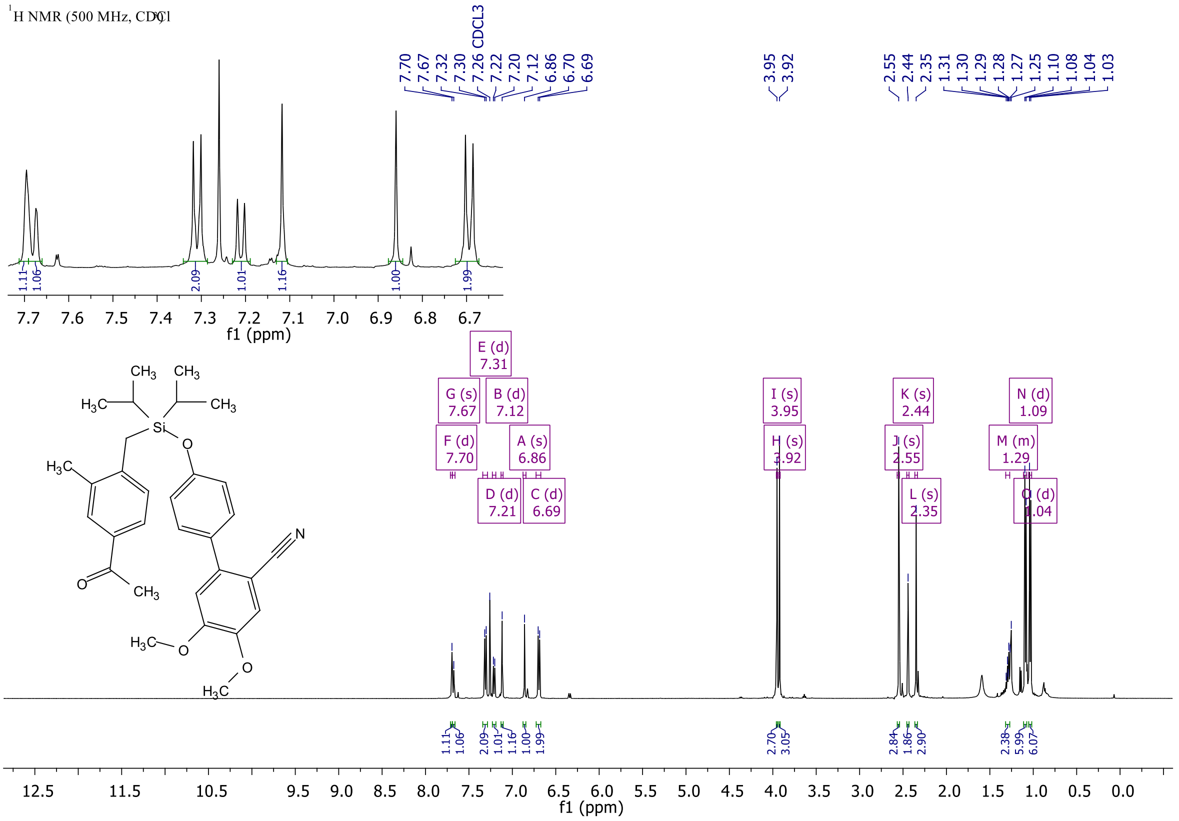

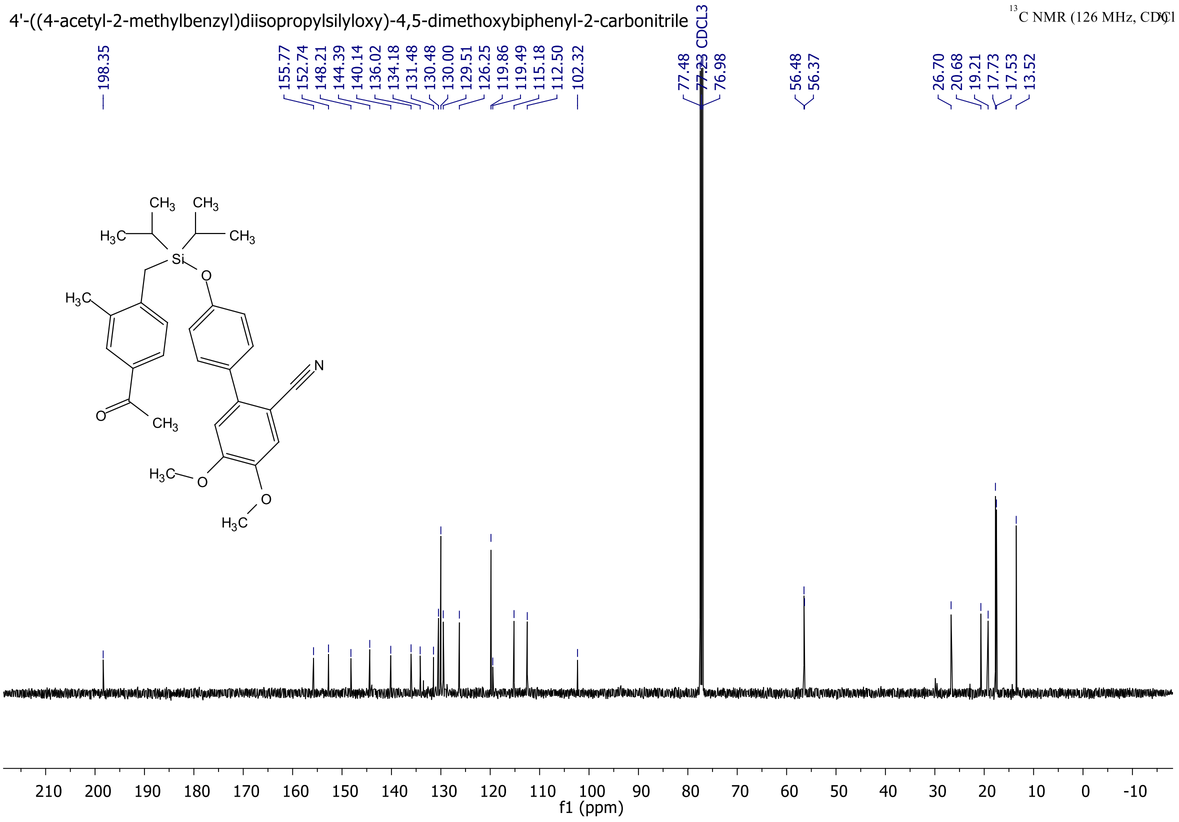

Supplementary Figure 32: ^{1}H and ^{13}C NMR (500 MHz, $CDCl_3$) of 4'-((4-acetyl-3-methoxybenzyl)diisopropylsilyloxy)-4,5-dimethoxybiphenyl-2-carbonitrile (2c):

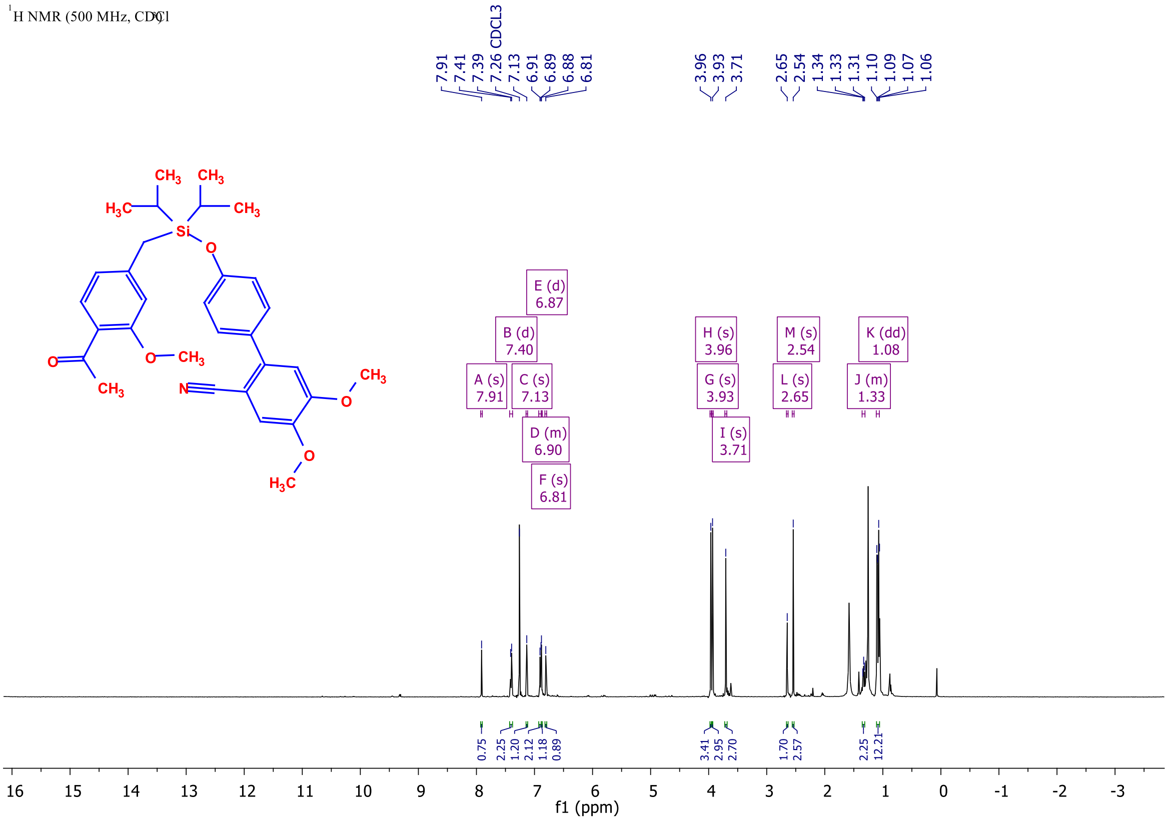

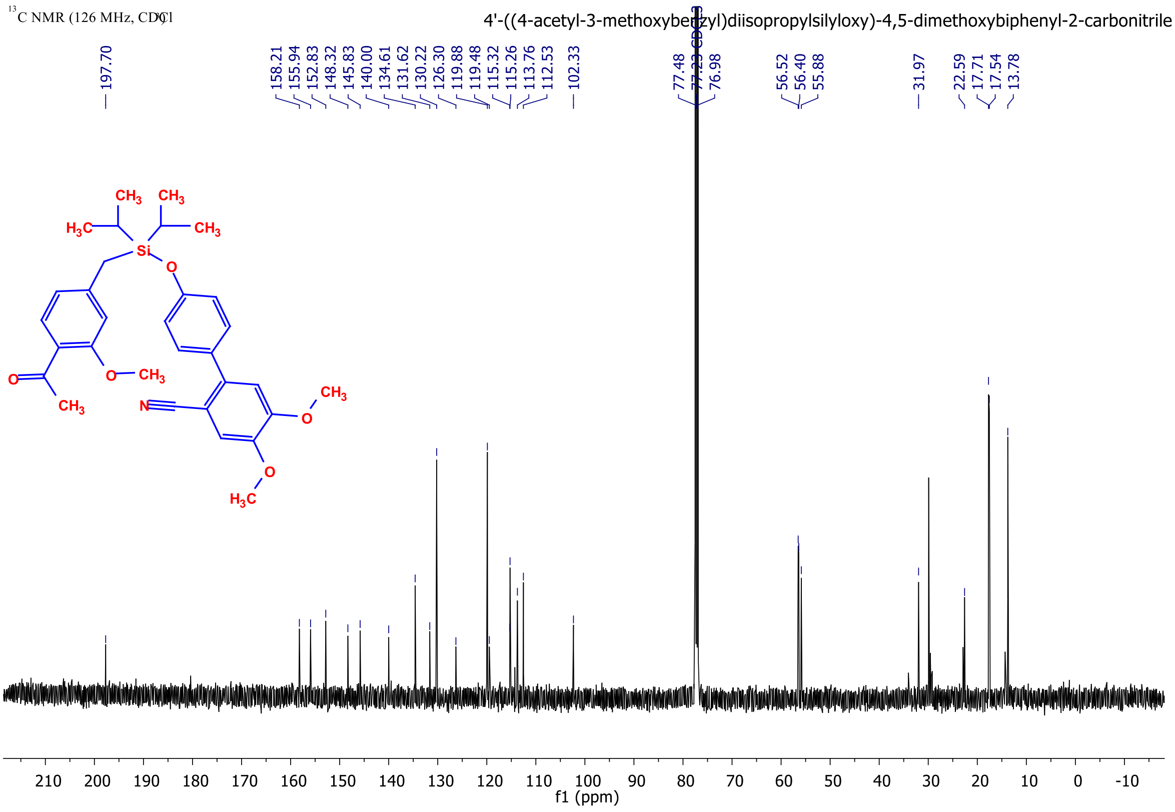

Supplementary Figure 33: ^{1}H and ^{13}C NMR (500 MHz, $CDCl_3$) of 4'-((4-acetyl-2-(trifluoromethoxy)benzyl)diisopropylsilyloxy)-4,5-dimethoxybiphenyl-2-carbonitrile (2d):

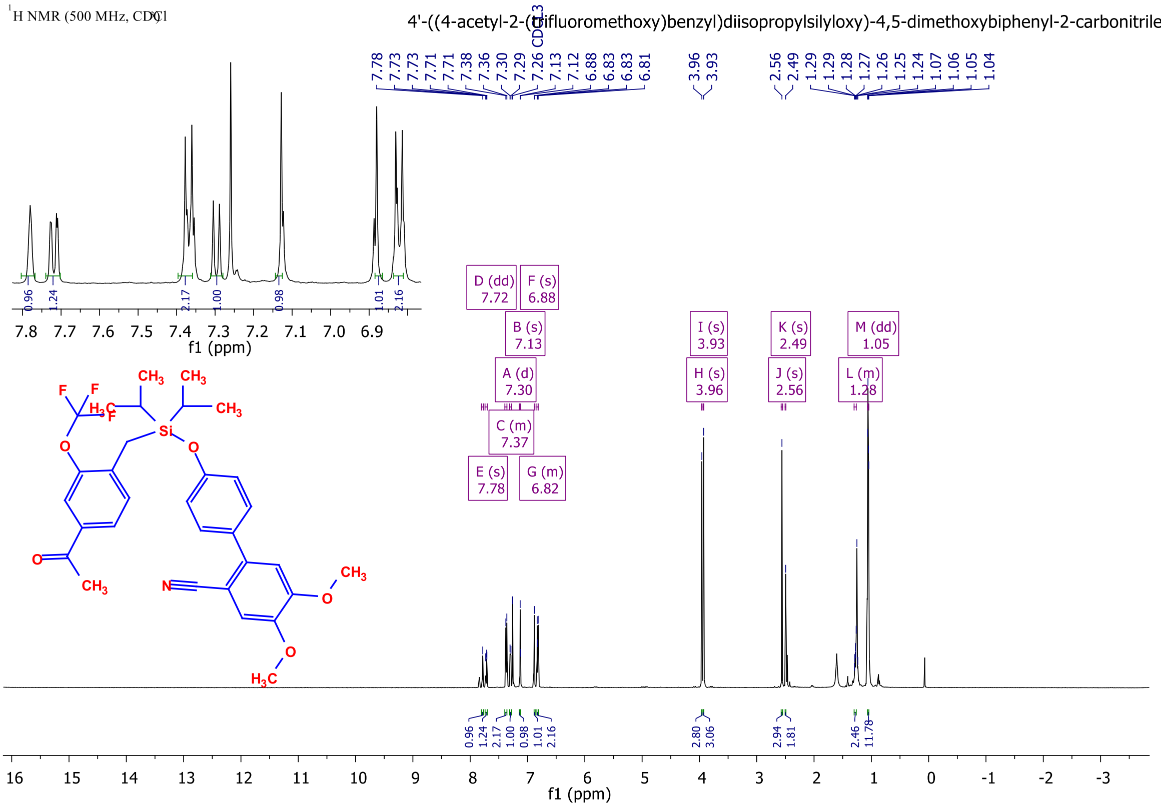

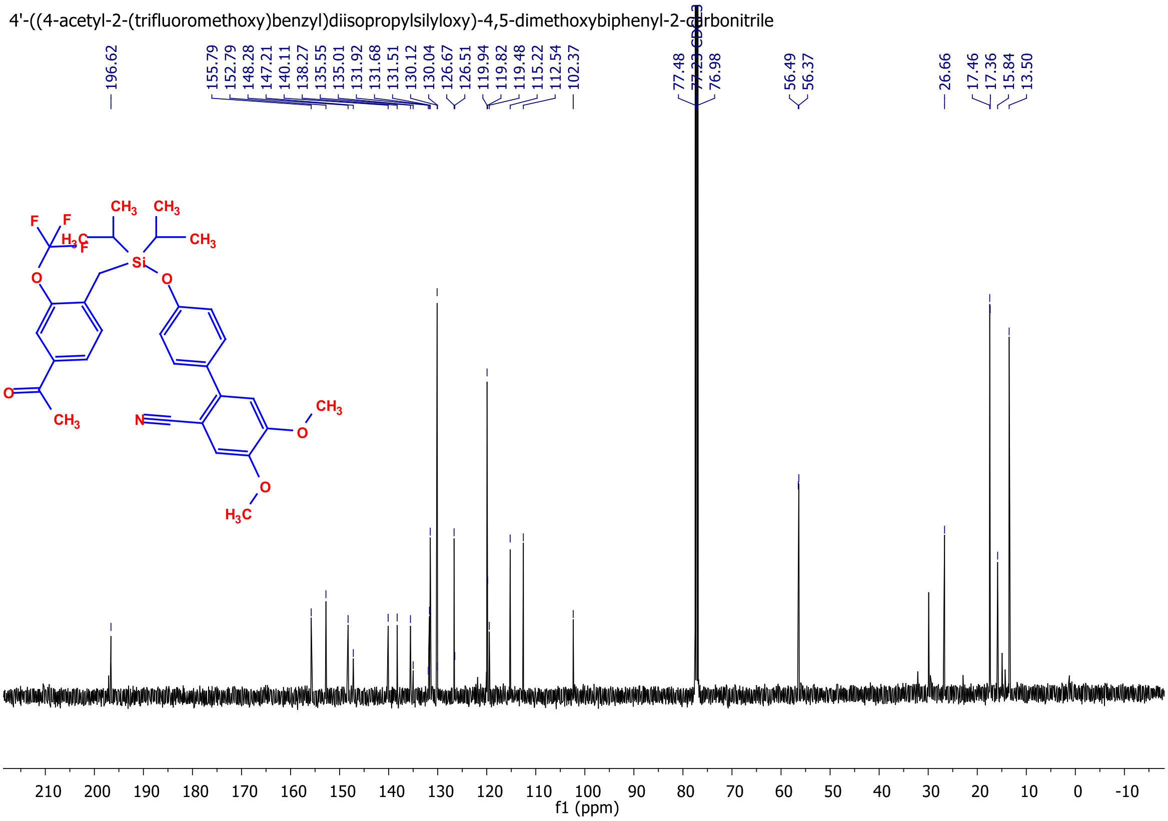

Supplementary Figure 34: ^1H and ^{13}C NMR (400 MHz, $CDCl_3$) of 4'-((4-acetyl-2,6-dimethylbenzyl)diisopropylsilyloxy)-4,5-dimethoxybiphenyl-2-carbonitrile (2e):

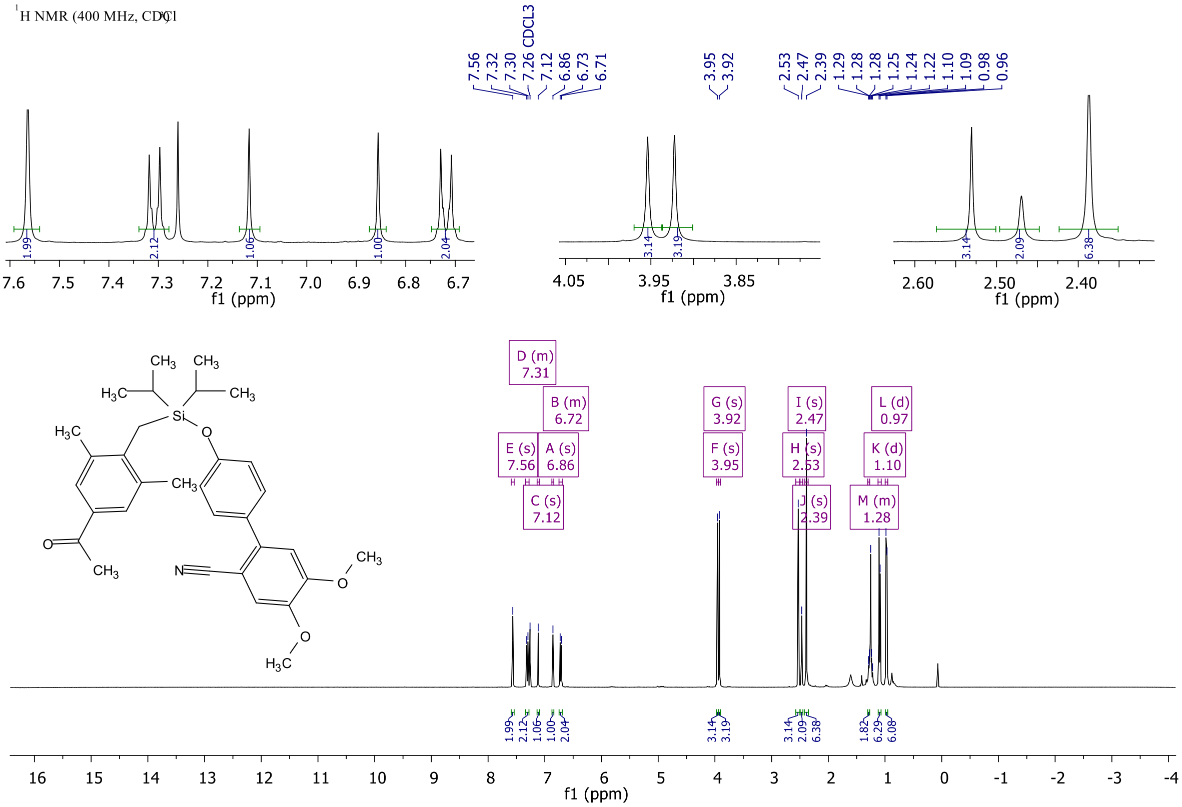

4'-((4-acetyl-2,6-dimethylbenzyl)diisopropylsilyloxy)-4,5-dimethoxybiphenyl-2-carbonitrile

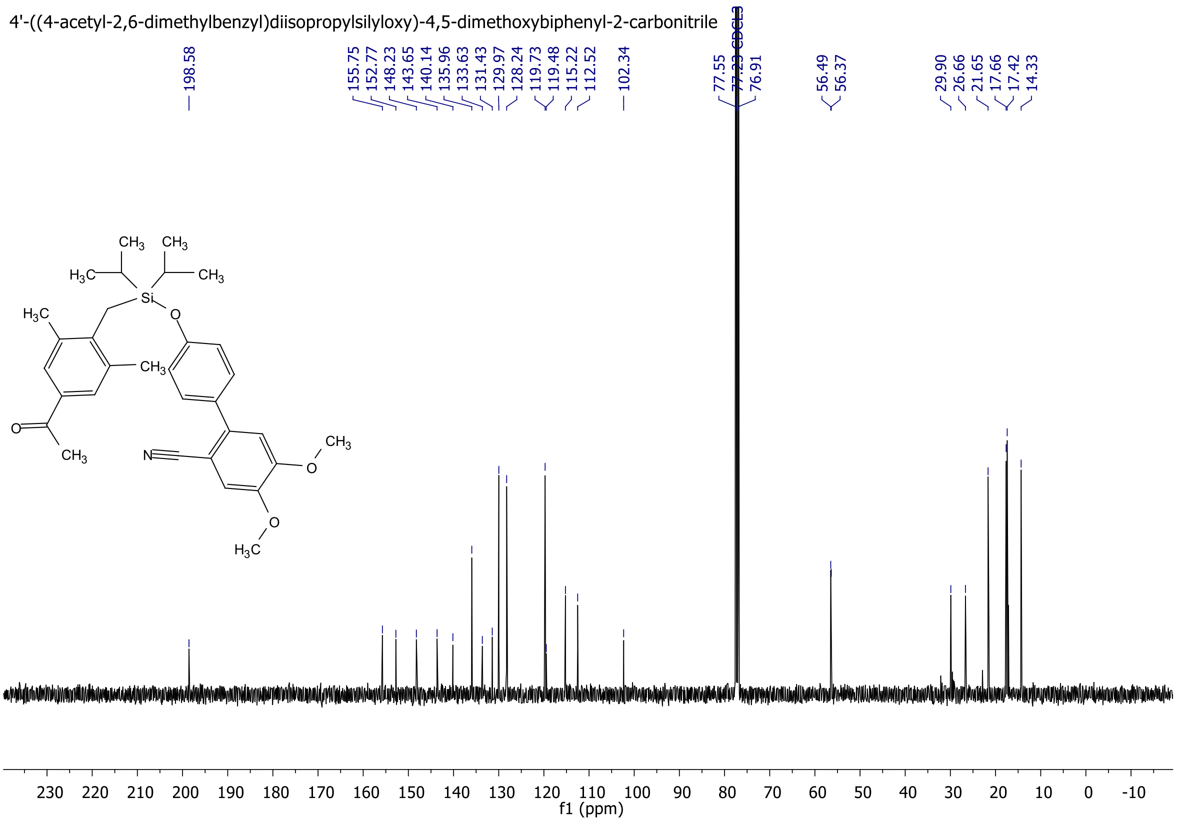

Supplementary Figure 35: ^{1}H and ^{13}C NMR (400 MHz, $CDCl_3$) of 4'-(((4-acetyl-2-((trifluoromethyl)thio)benzyl)diisopropylsilyl)oxy)-4,5-dimethoxy-[1,1'-biphenyl]-2-carbonitrile (2f):

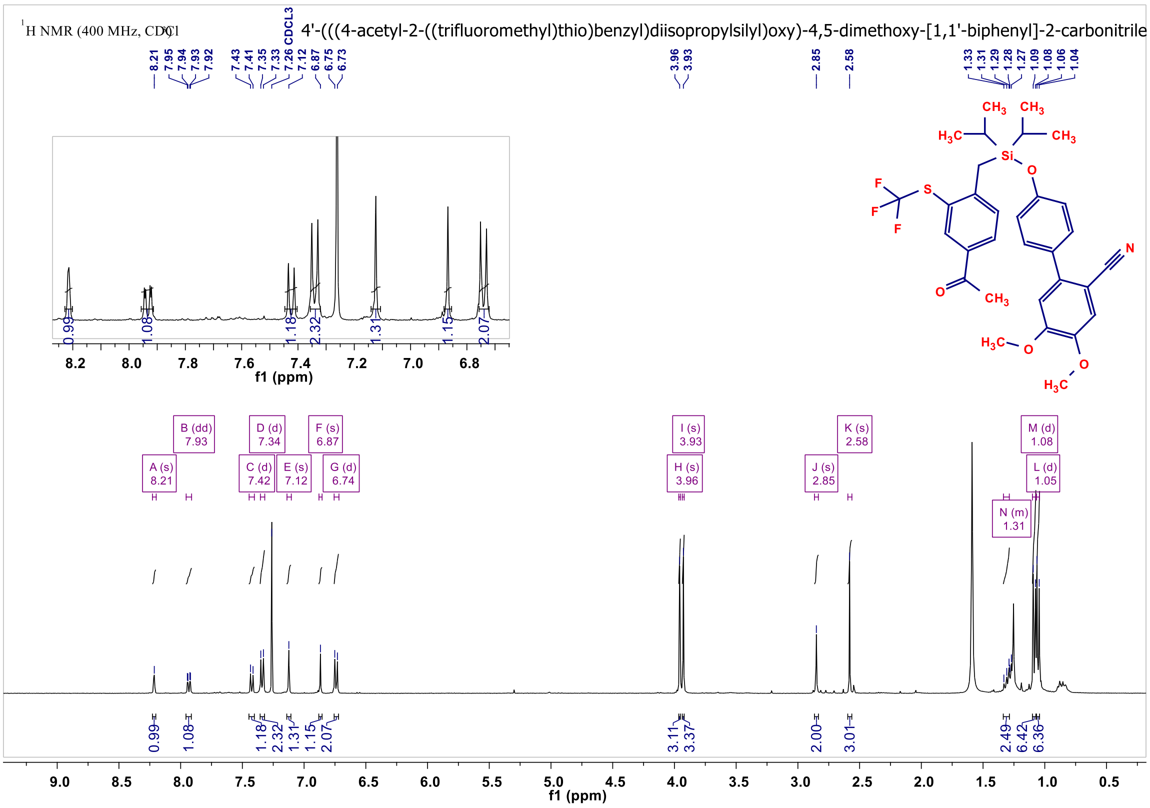

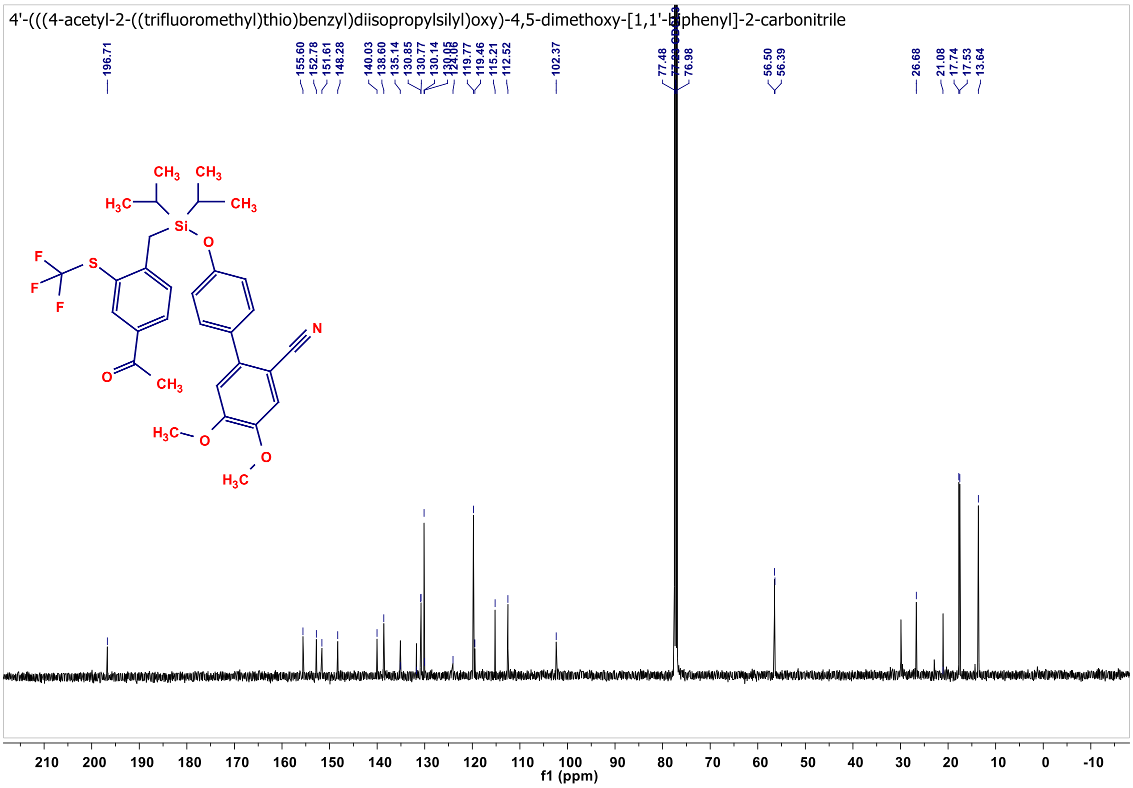

Supplementary Figure 36: ^{1}H and ^{13}C NMR (500 MHz, $CDCl_3$) of 4'-(((4-acetyl-2-(difluoromethoxy)benzyl)diisopropylsilyl)oxy)-4,5-dimethoxy-[1,1'-biphenyl]-2-carbonitrile (2g):

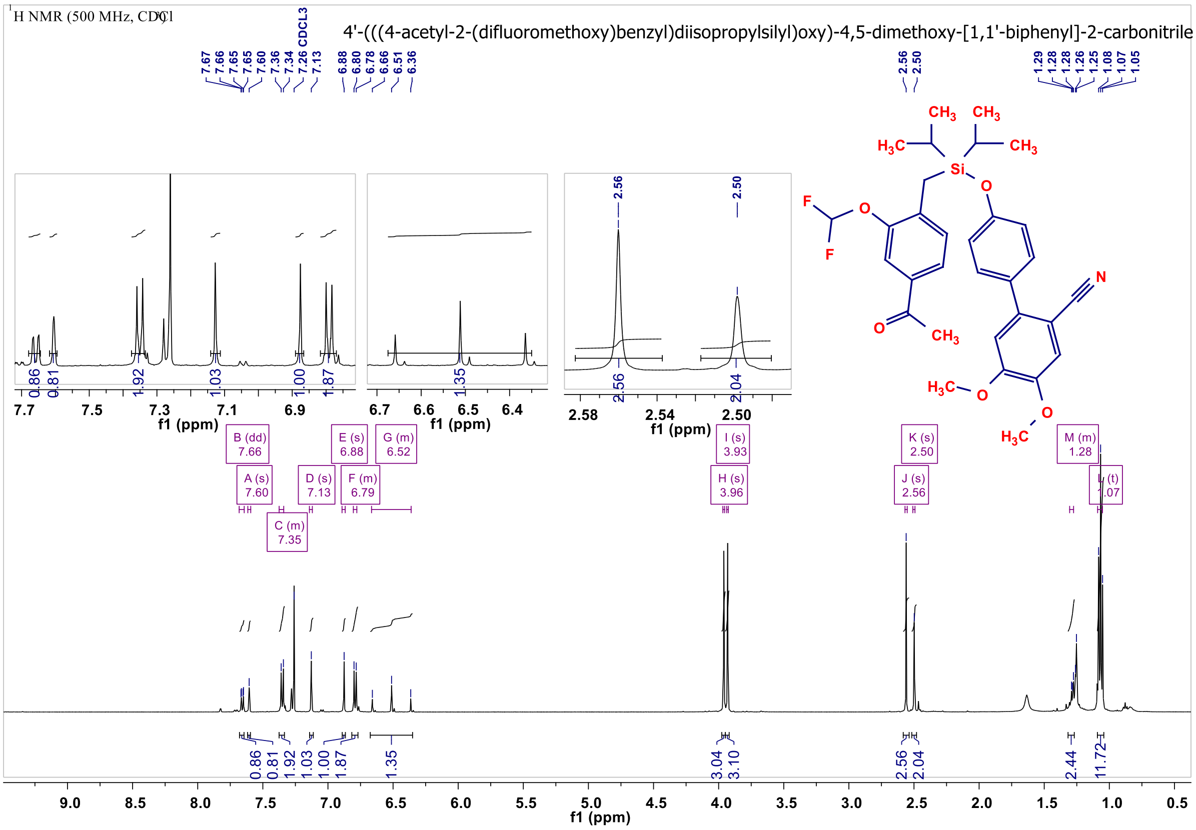

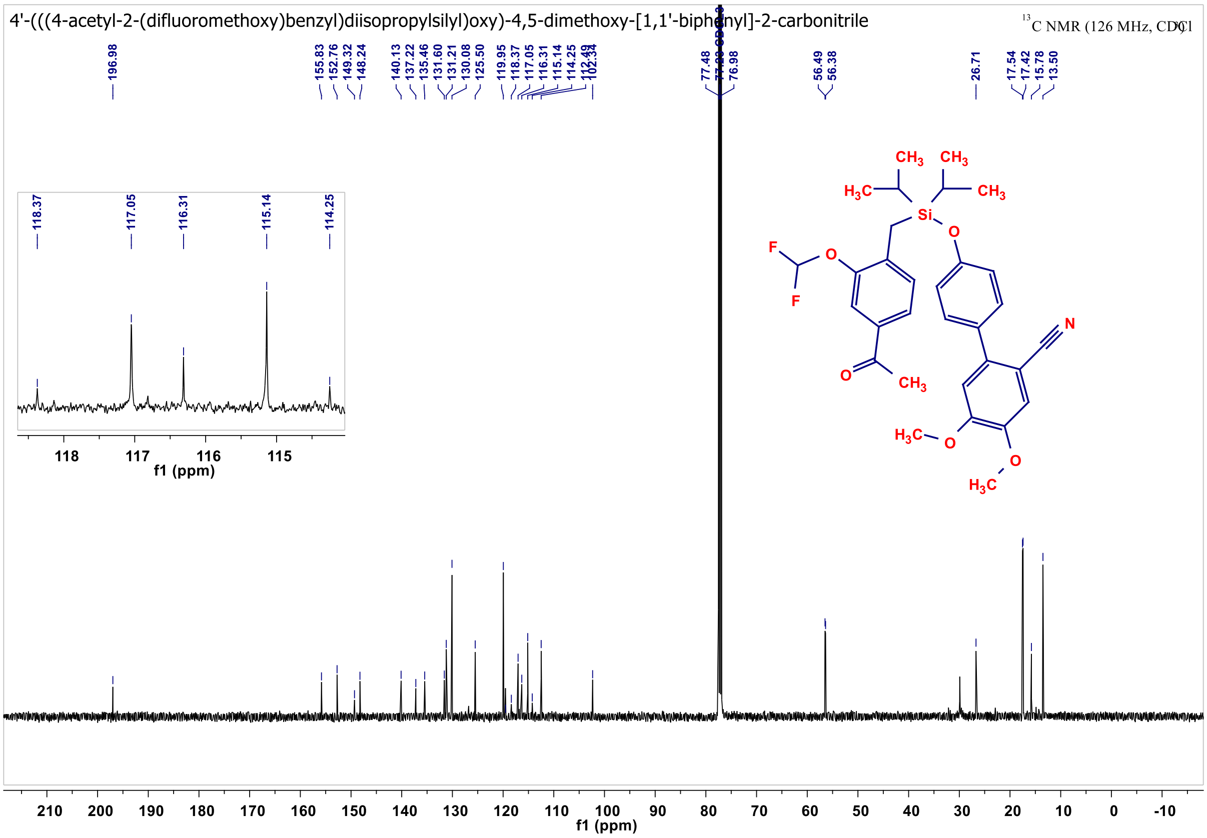

Supplementary Figure 37: ^{1}H and ^{13}C NMR (500 MHz, $CDCl_3$) of 4'-((4-acetyl-3-methylbenzyl)diisopropylsilyloxy)-4,5-dimethoxybiphenyl-2-carbonitrile (2h):

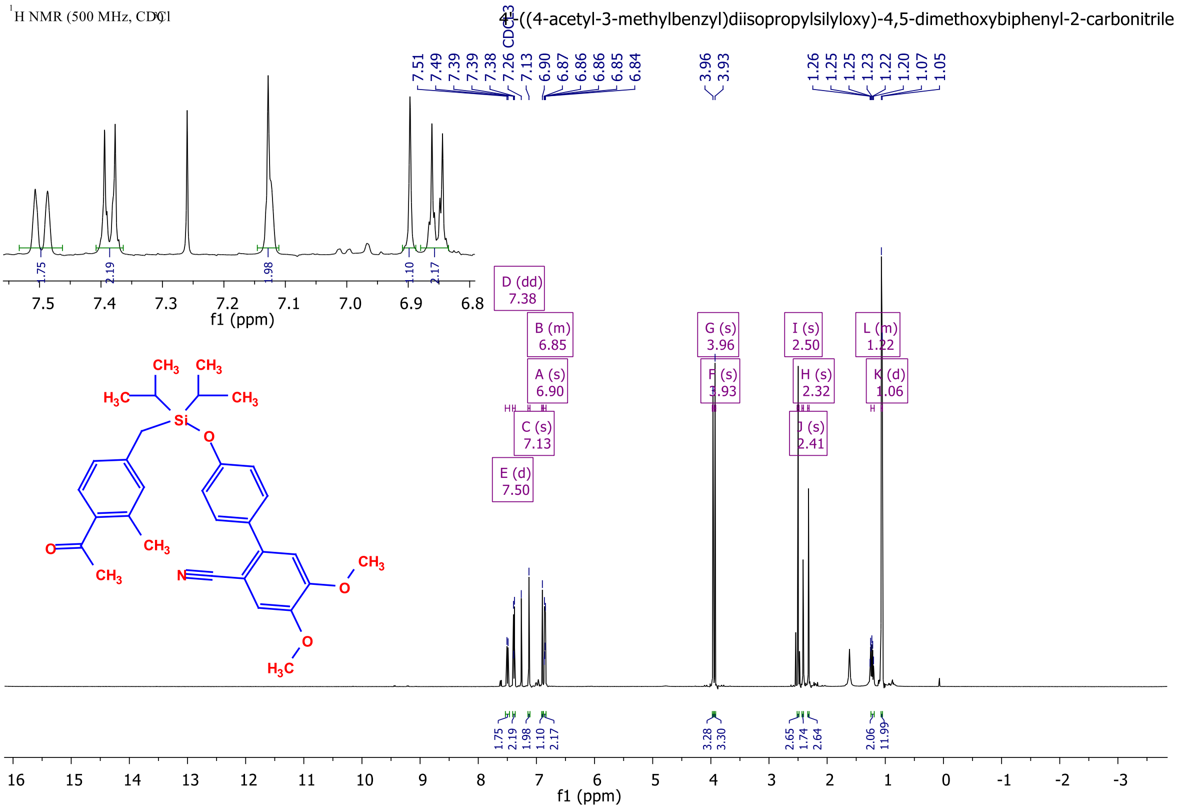

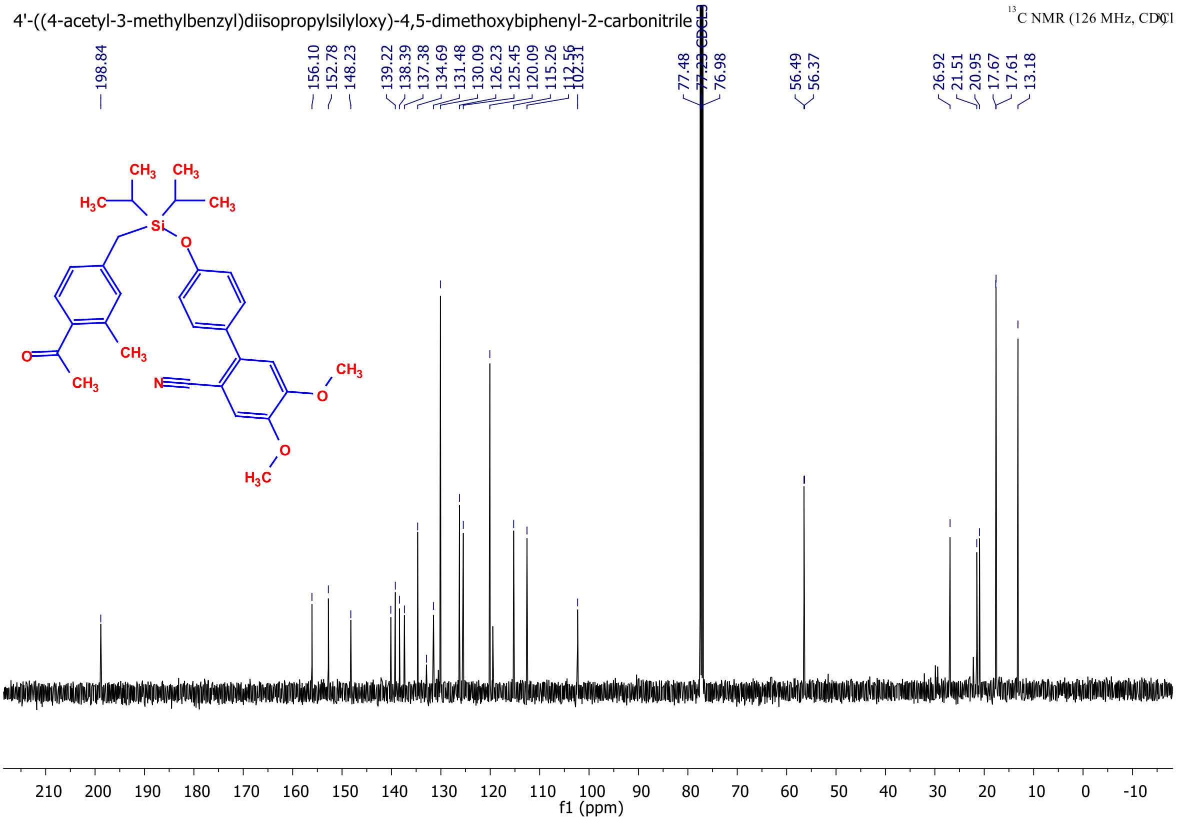

Supplementary Figure 38: ^{1}H and ^{13}C NMR (500 MHz, $CDCl_3$) of 4'-((4-acetyl-3,5-dimethylbenzyl)diisopropylsilyloxy)-4,5-dimethoxybiphenyl-2-carbonitrile (2i):

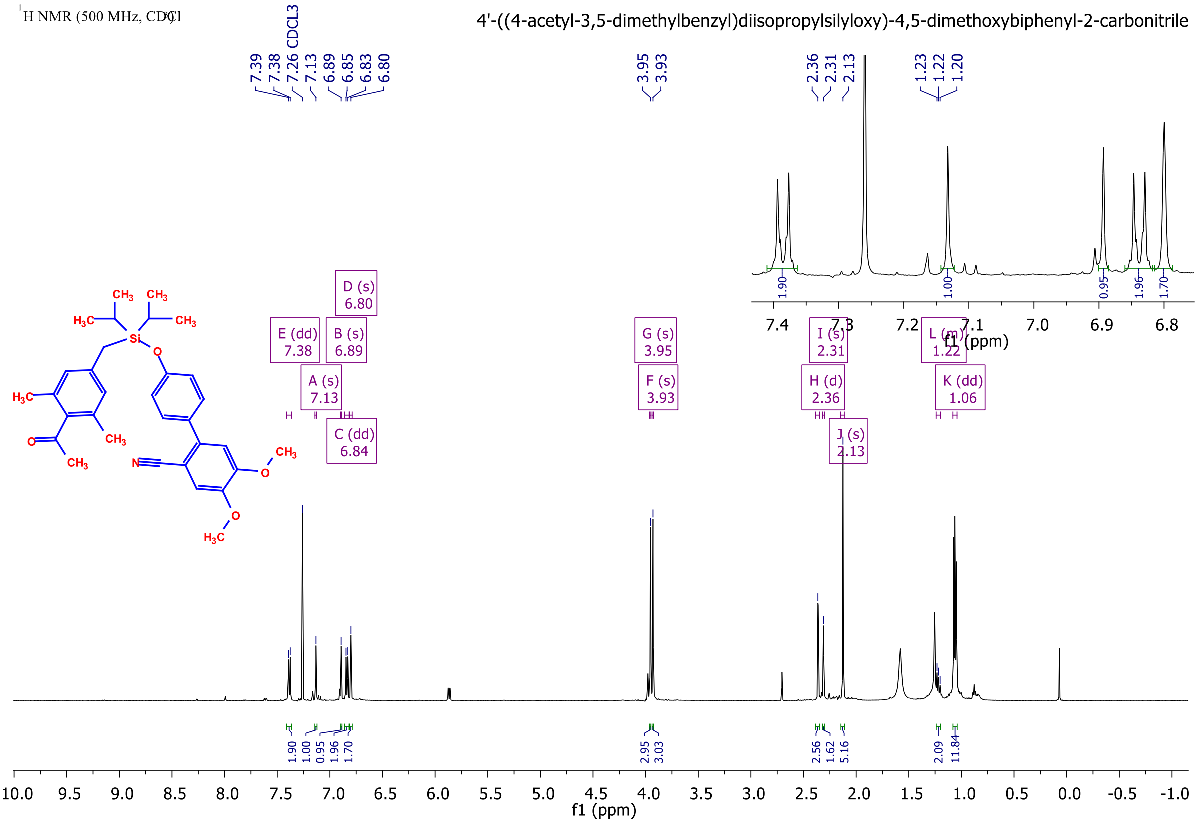

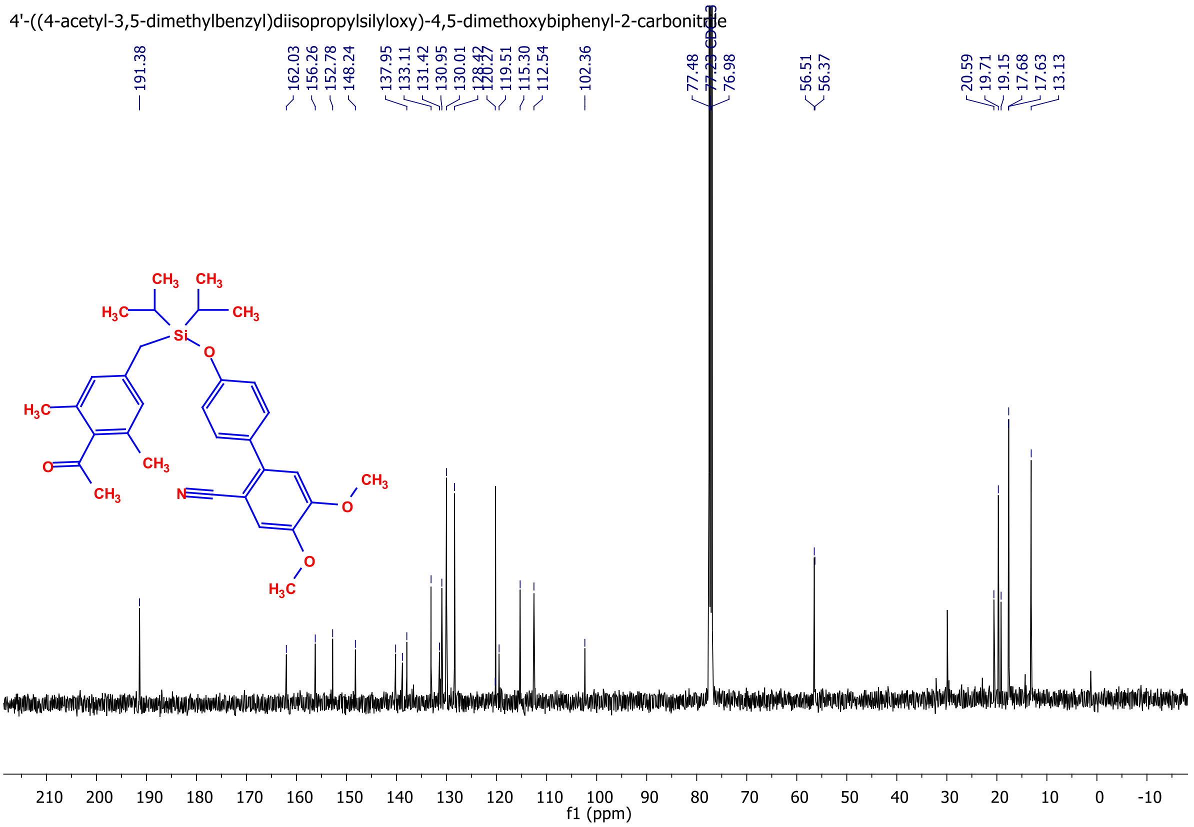

Characterizations: Electron Deficient Arene Scope

Supplementary Figure 38: ^{1}H and ^{13}C NMR (500 MHz, $CDCl_3$) of 4'-((4-acetyl-2-fluoro-6-(trifluoromethyl)benzyl)diisopropylsilyloxy)-4,5-dimethoxybiphenyl-2-carbonitrile (3a):

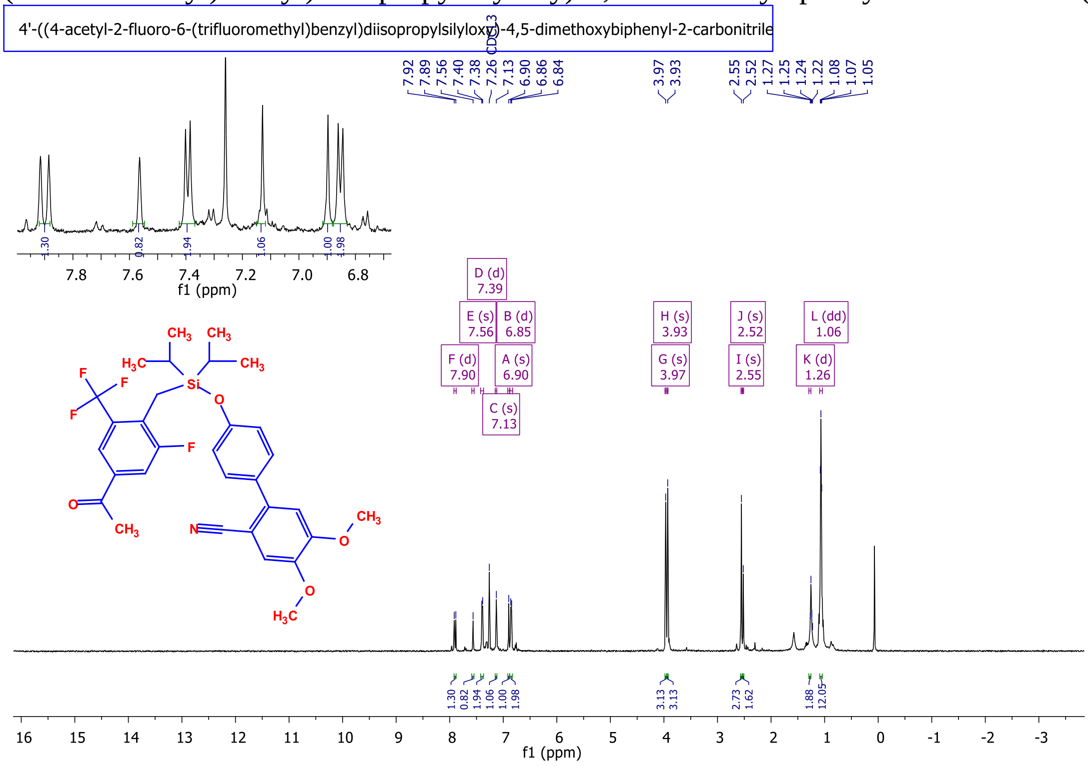

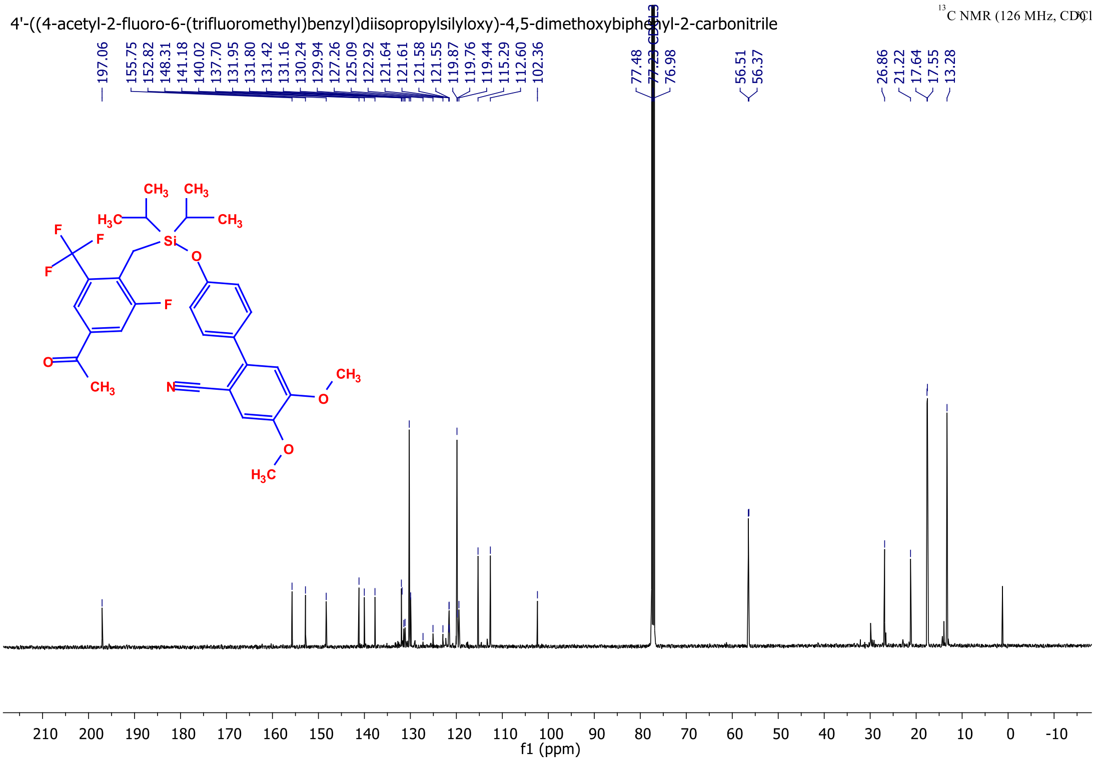

Supplementary Figure 39: ^{1}H and ^{13}C NMR (500 MHz, $CDCl_3$) of 4'-((4-acetyl-2,6-difluorobenzyl)diisopropylsilyloxy)-4,5-dimethoxybiphenyl-2-carbonitrile (3b):

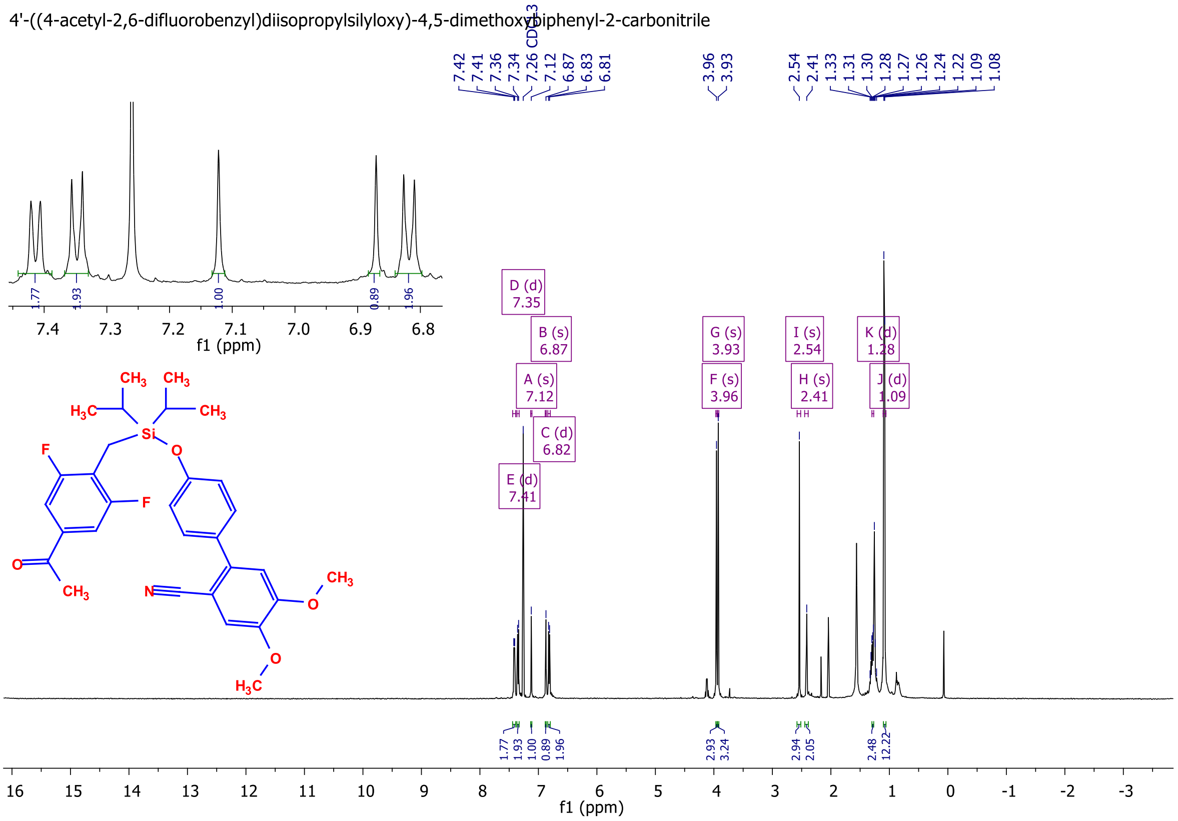

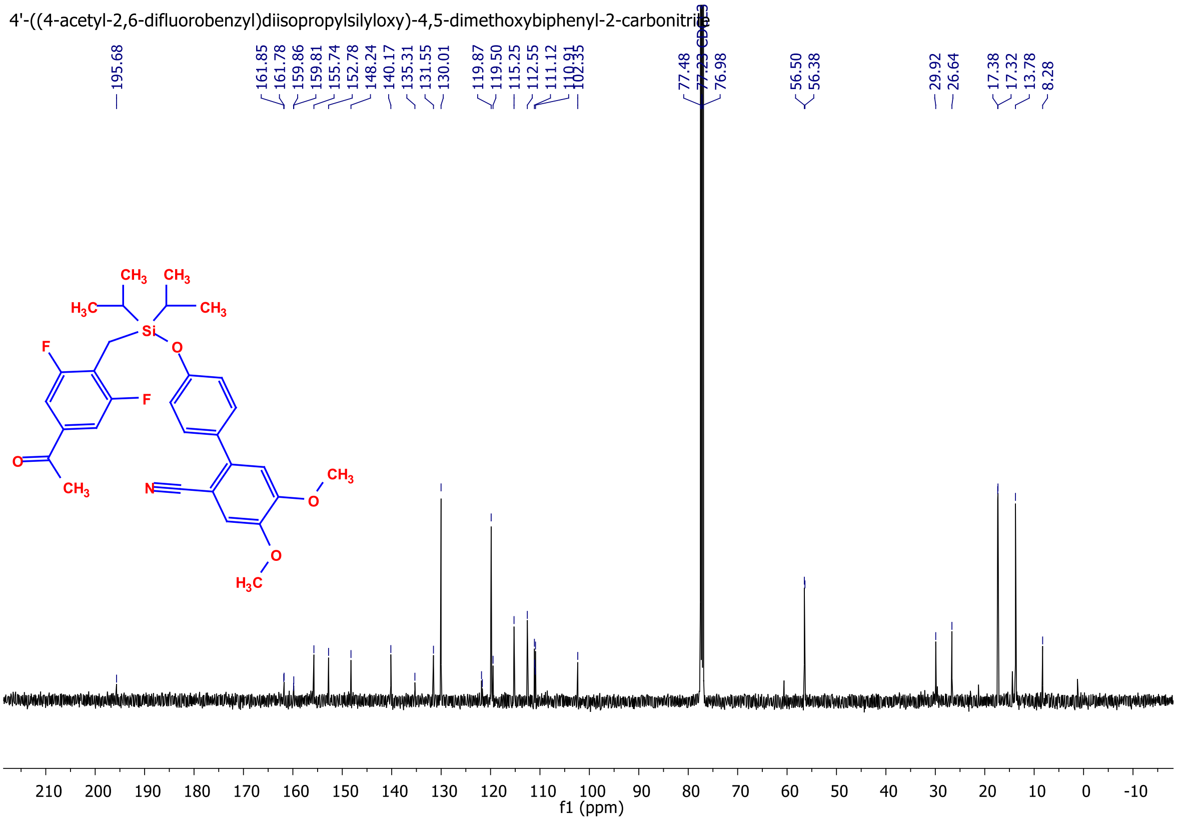

Supplementary Figure 40: ^{1}H and ^{13}C NMR (500 MHz, $CDCl_3$) of 4'-((4-acetyl-2-chloro-6-fluorobenzyl)diisopropylsilyloxy)-4,5-dimethoxybiphenyl-2-carbonitrile (3c):

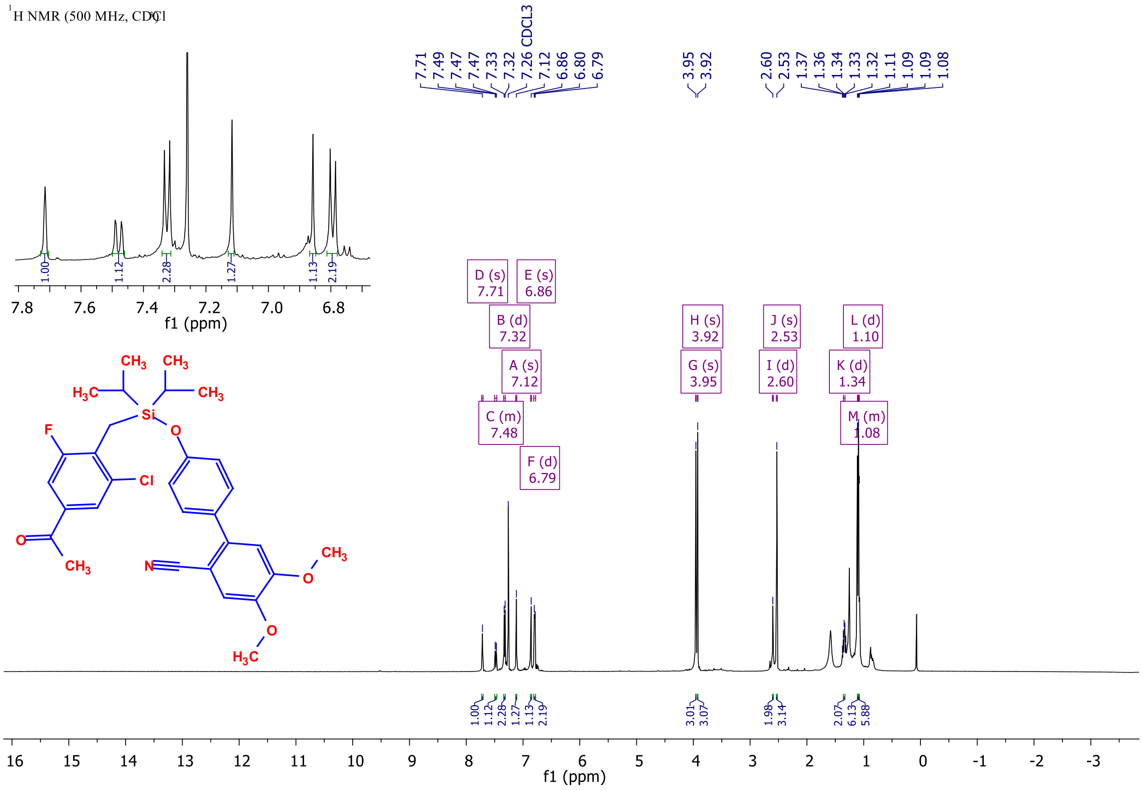

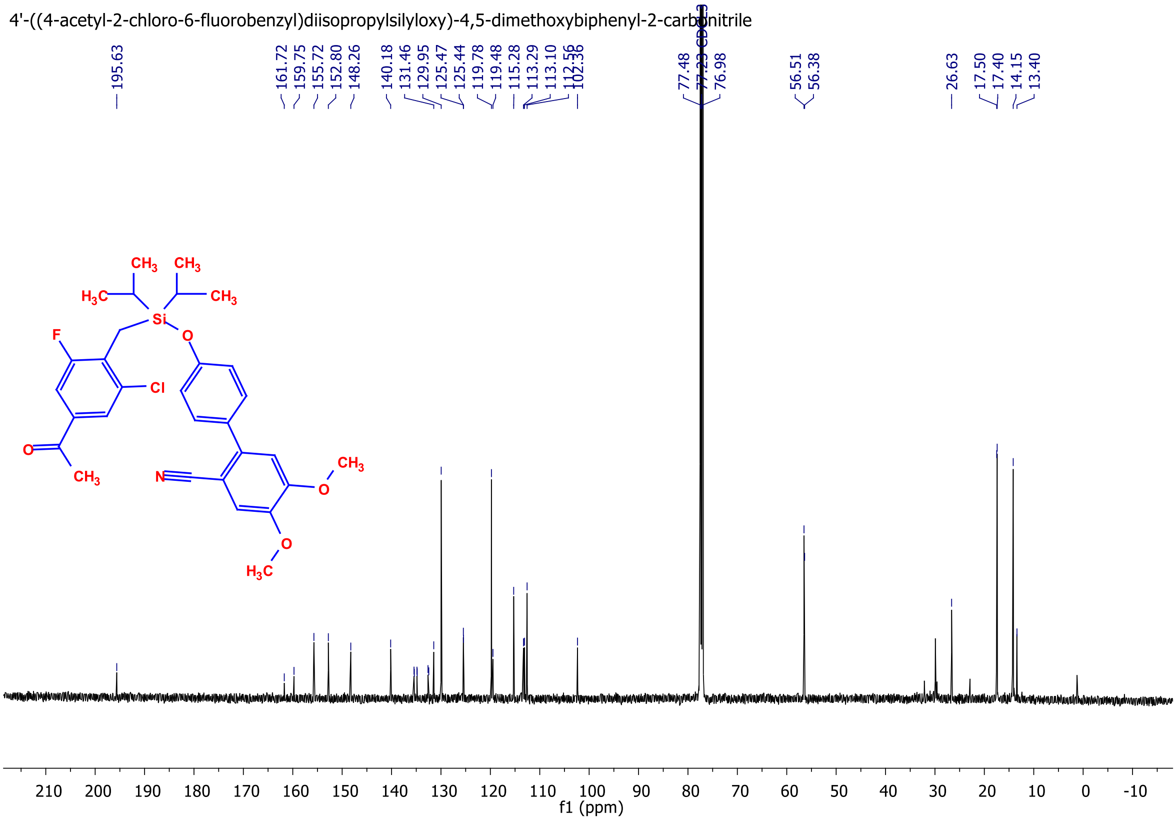

Supplementary Figure 41: ^{1}H and ^{13}C NMR (500 MHz, $CDCl_3$) of 4'-((4-acetyl-2,6-dichlorobenzyl)diisopropylsilyloxy)-4,5-dimethoxybiphenyl-2-carbonitrile (3d):

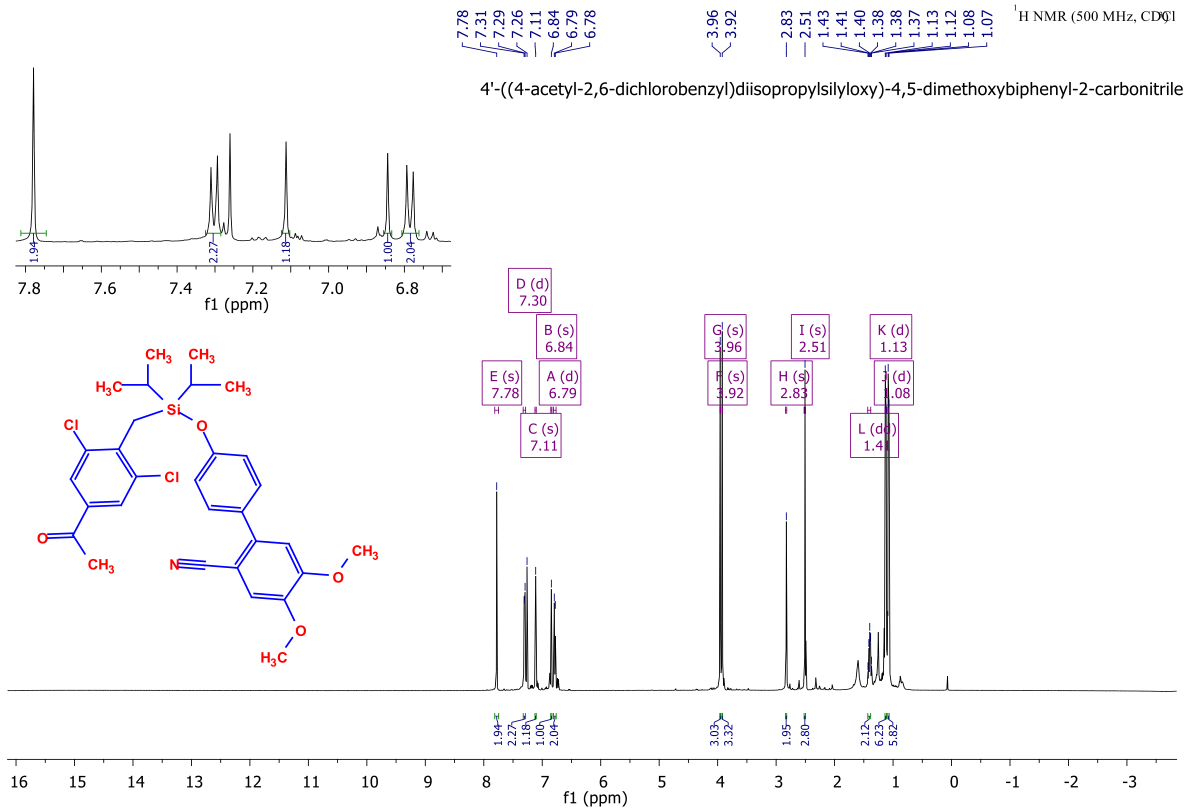

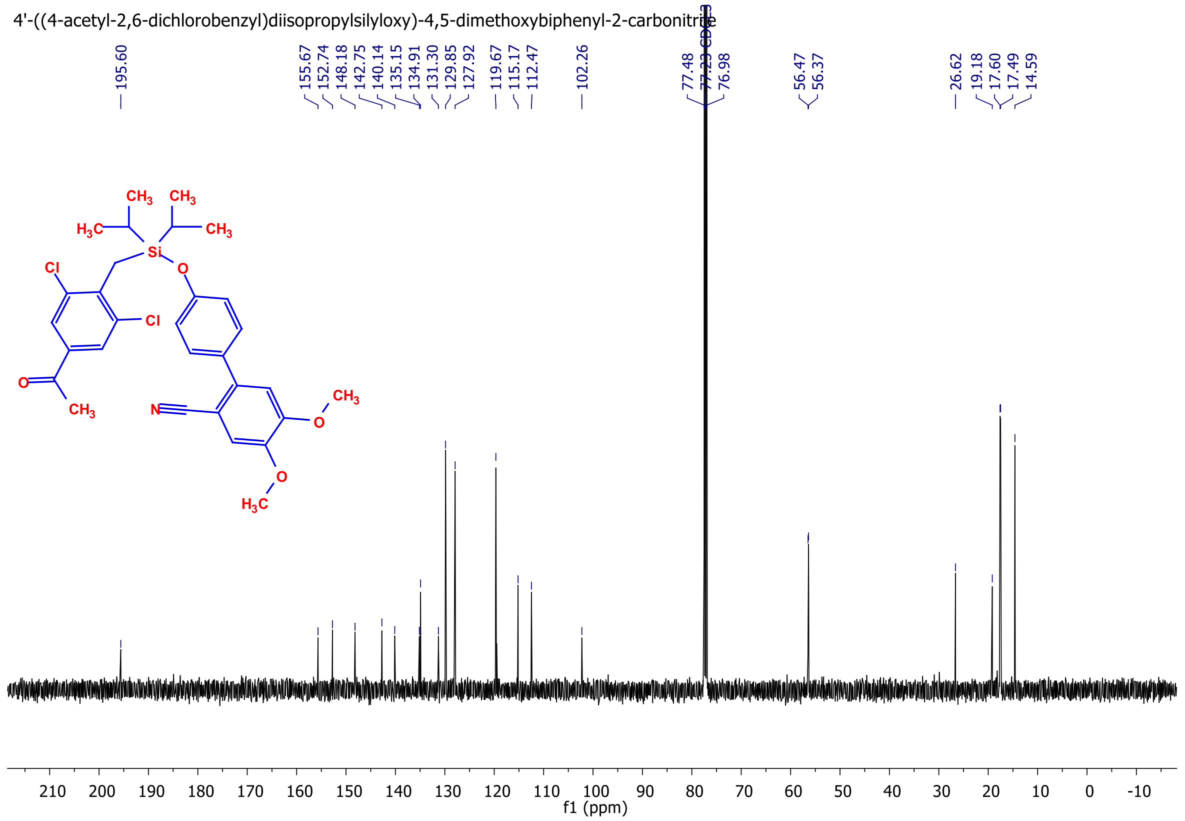

Supplementary Figure 42: ^{1}H (400 MHz, $CDCl_3$) and ^{13}C NMR (500 MHz, $CDCl_3$) of 4'-((4-acetyl-2-chloro-5-fluorobenzyl)diisopropylsilyloxy)-4,5-dimethoxybiphenyl-2-carbonitrile (3e):

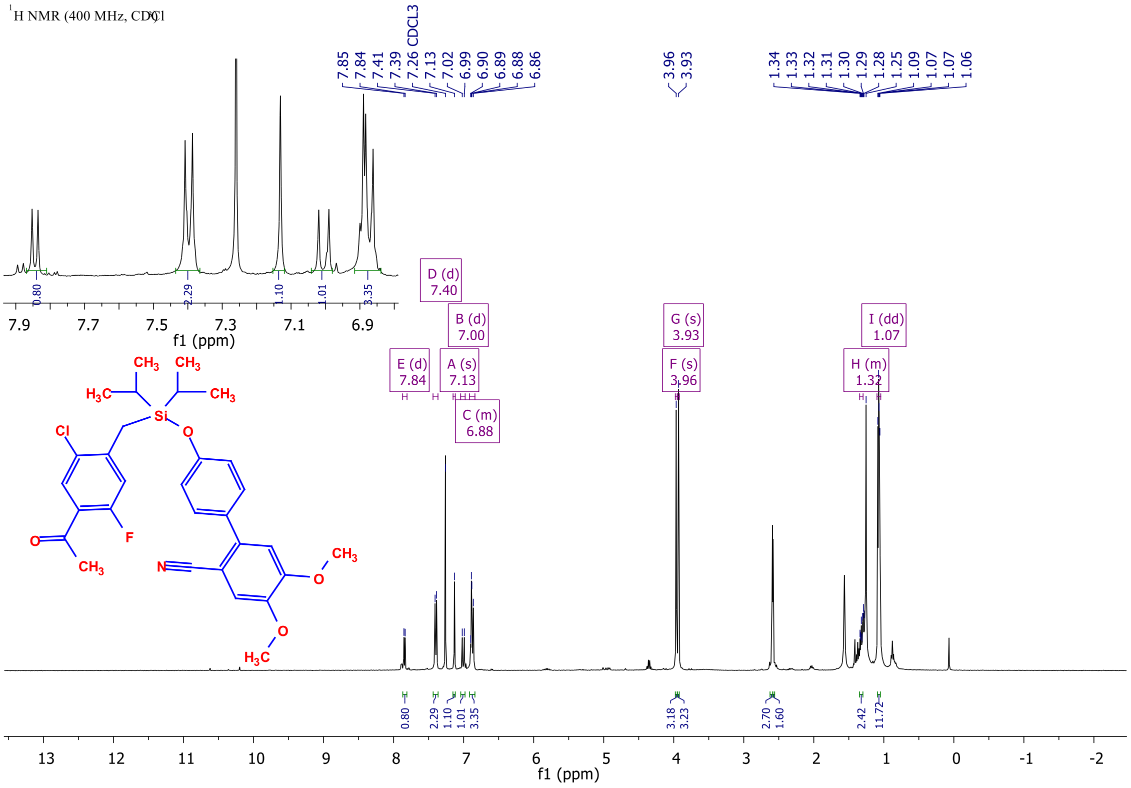

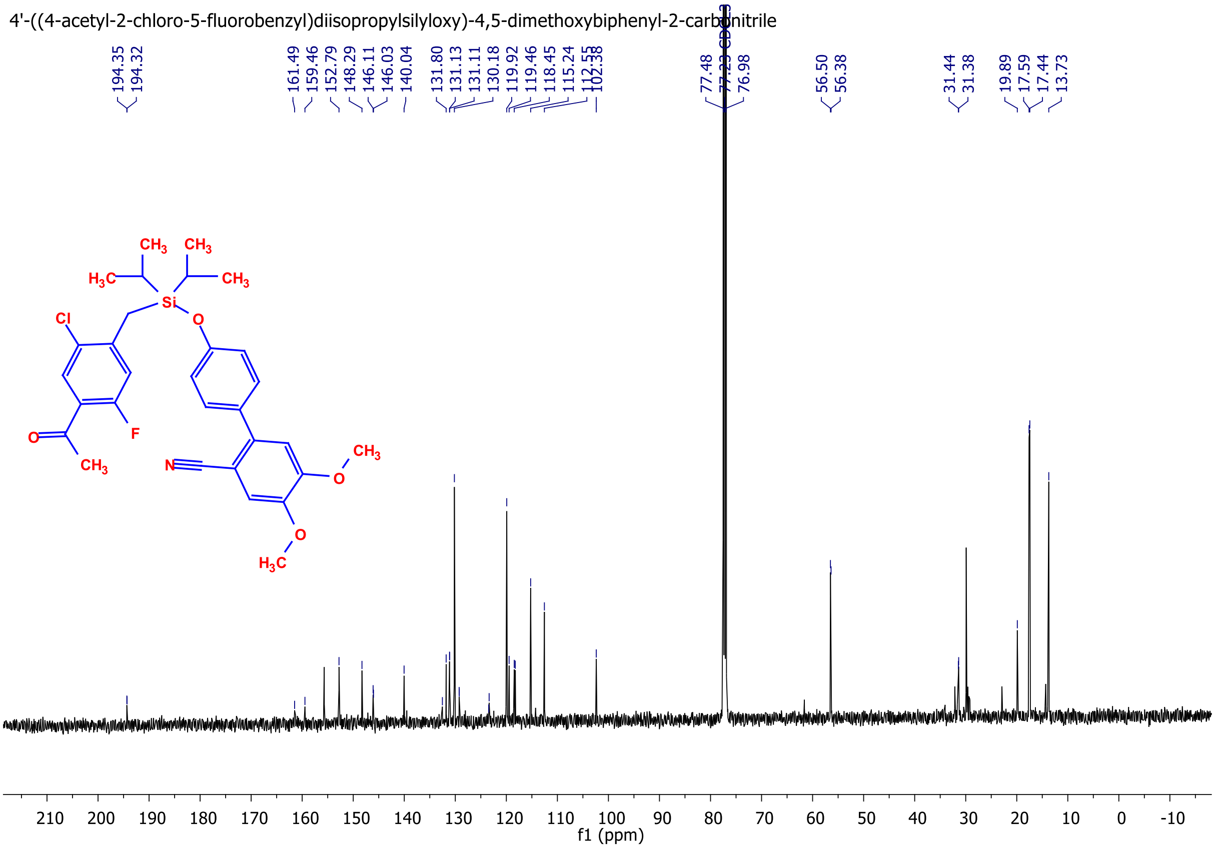

Supplementary Figure 43: ^{1}H and ^{13}C NMR (500 MHz, $CDCl_3$) of 4'-((4-acetyl-2,5-difluorobenzyl)diisopropylsilyloxy)-4,5-dimethoxybiphenyl-2-carbonitrile (3f):

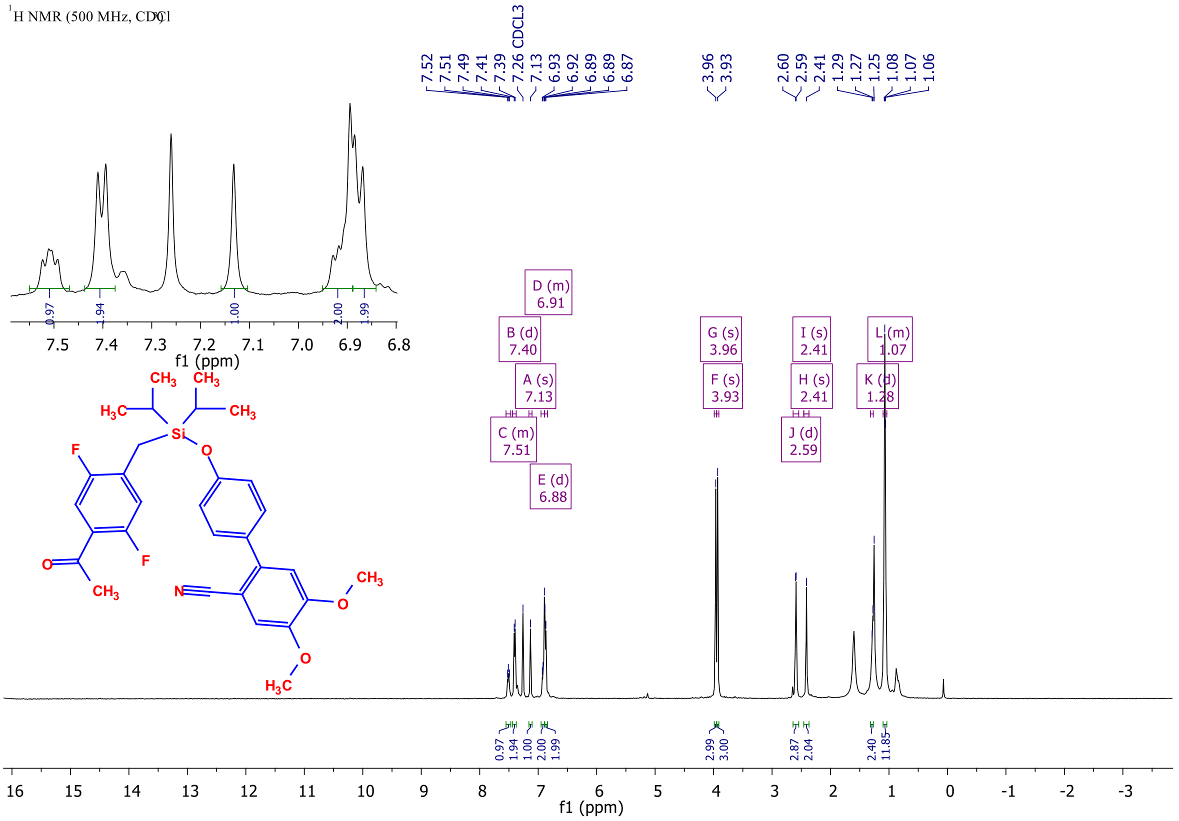

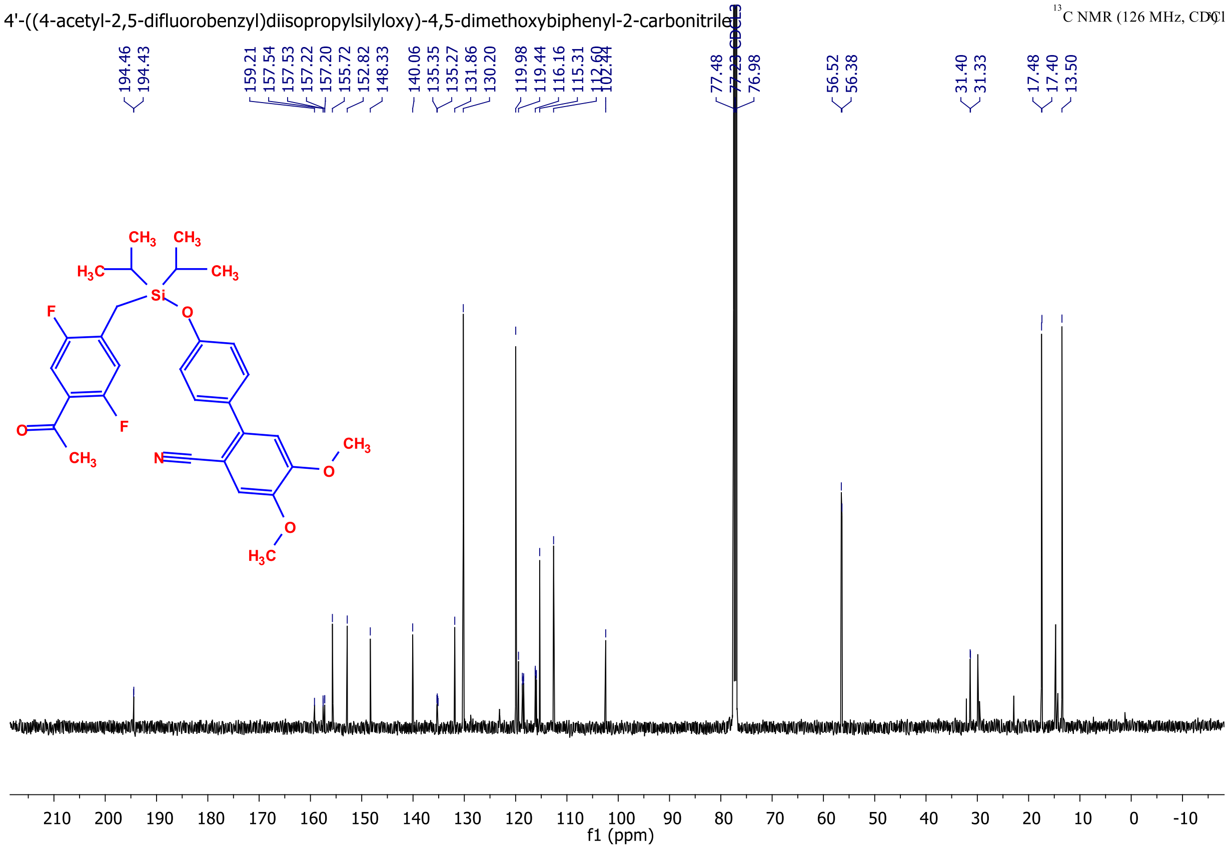

Supplementary Figure 44: ^{1}H and ^{13}C NMR (500 MHz, $CDCl_3$) of 4'-((4-acetyl-2-chlorobenzyl)diisopropylsilyloxy)-4,5-dimethoxybiphenyl-2-carbonitrile (3g):

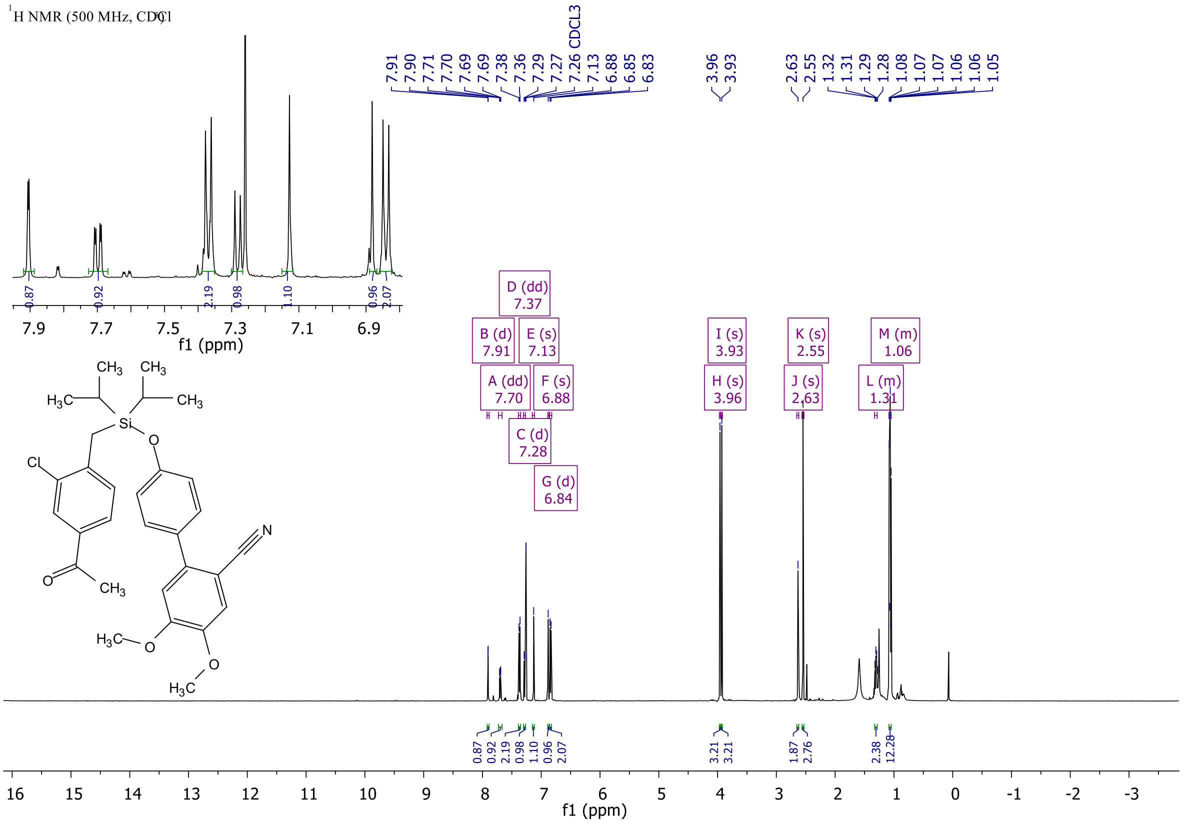

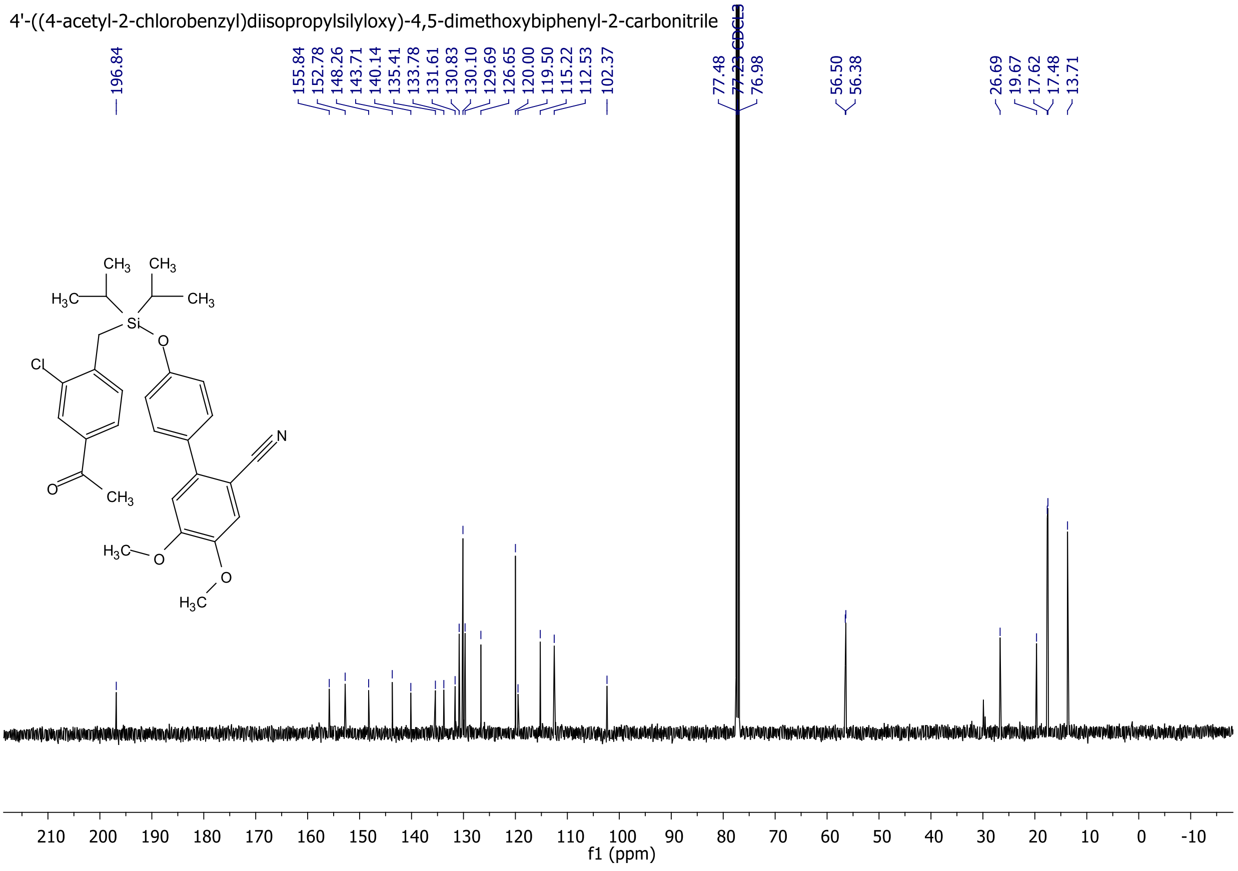

Supplementary Figure 45: ^{1}H and ^{13}C NMR (500 MHz, $CDCl_3$) of 4'-((4-acetyl-3-fluorobenzyl)diisopropylsilyloxy)-4,5-dimethoxybiphenyl-2-carbonitrile (3h):

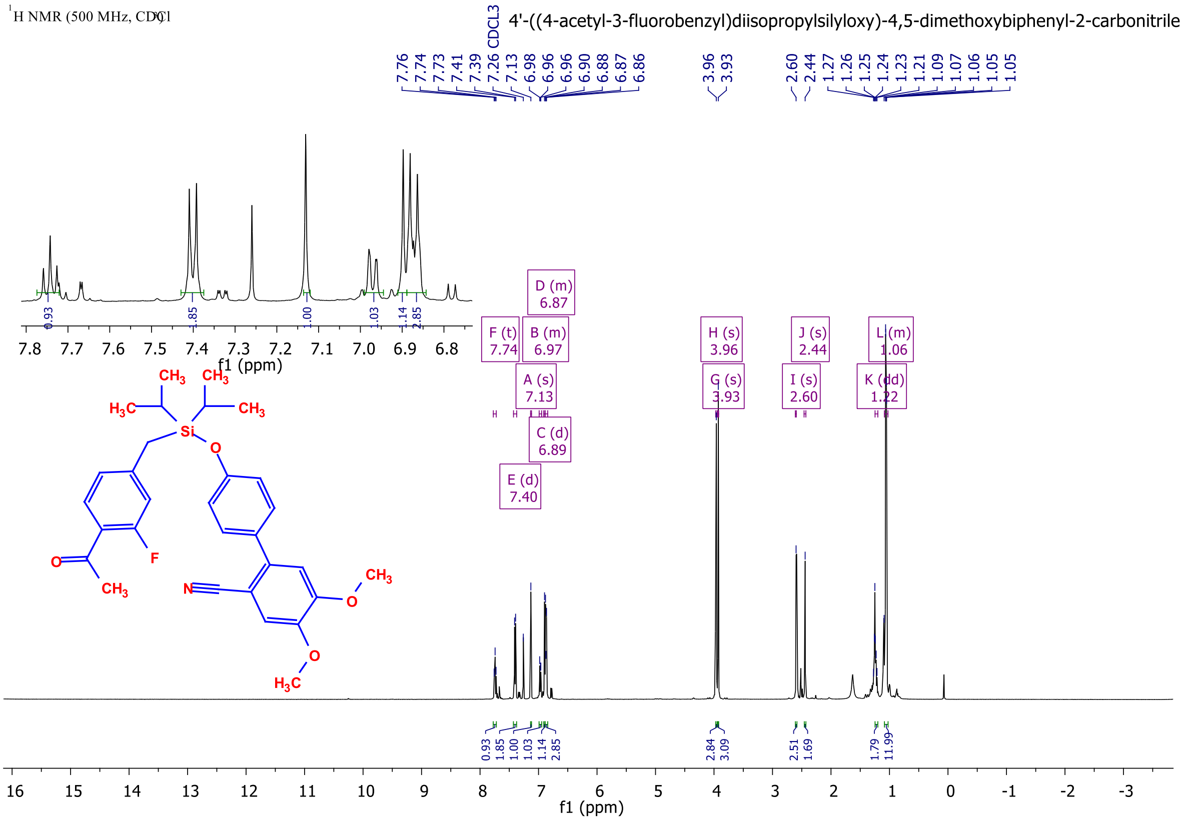

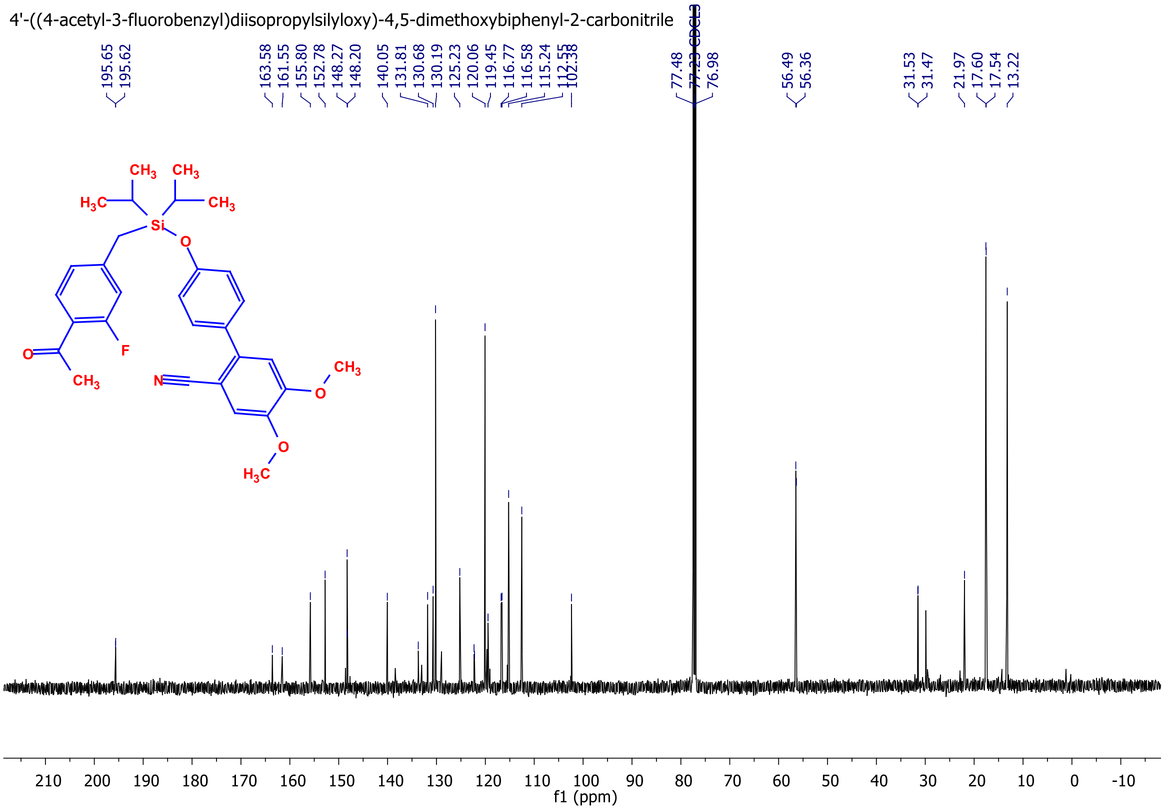

Supplementary Figure 46: ^{1}H and ^{13}C NMR (500 MHz, $CDCl_3$) of 4'-((4-acetyl-3-chloro-5-fluorobenzyl)diisopropylsilyloxy)-4,5-dimethoxybiphenyl-2-carbonitrile (3i):

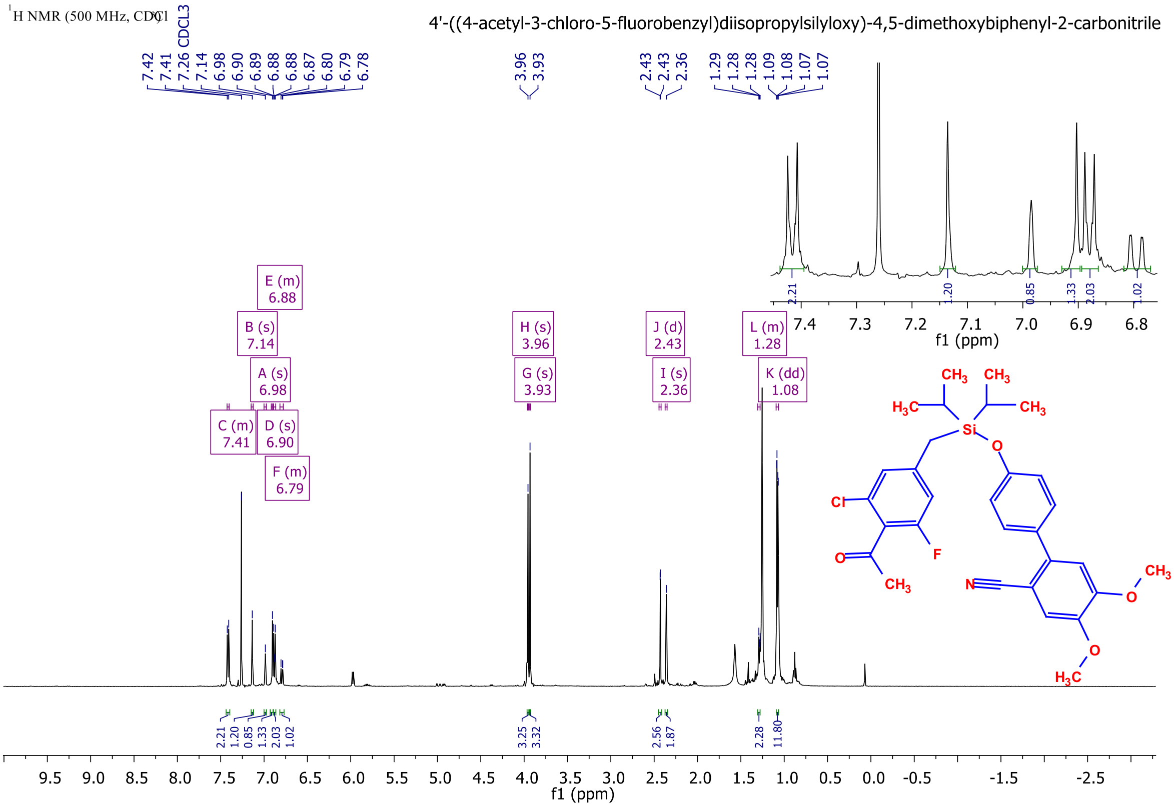

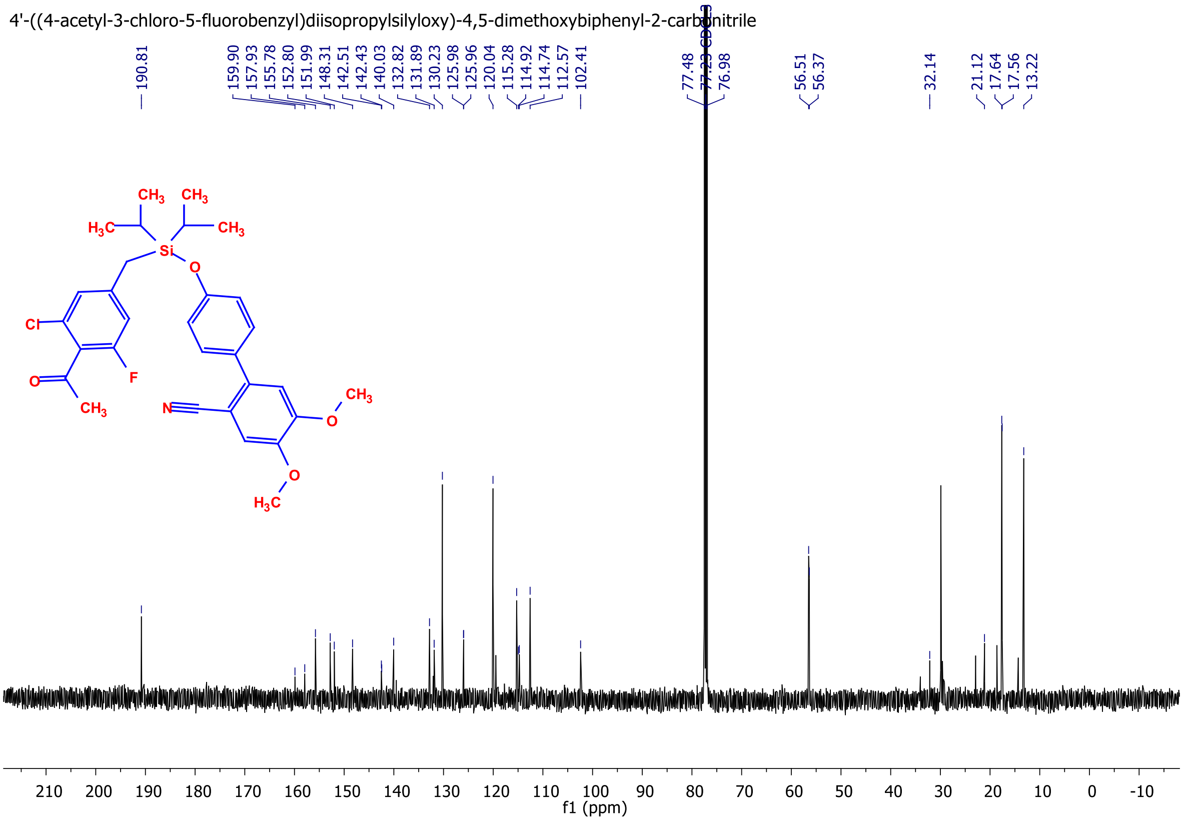

Supplementary Figure 47: ^{1}H (400 MHz, $CDCl_3$) and ^{13}C NMR (500 MHz, $CDCl_3$) of 4'-((4-acetyl-2,3-difluorobenzyl)diisopropylsilyloxy)-4,5-dimethoxybiphenyl-2-carbonitrile (3j):

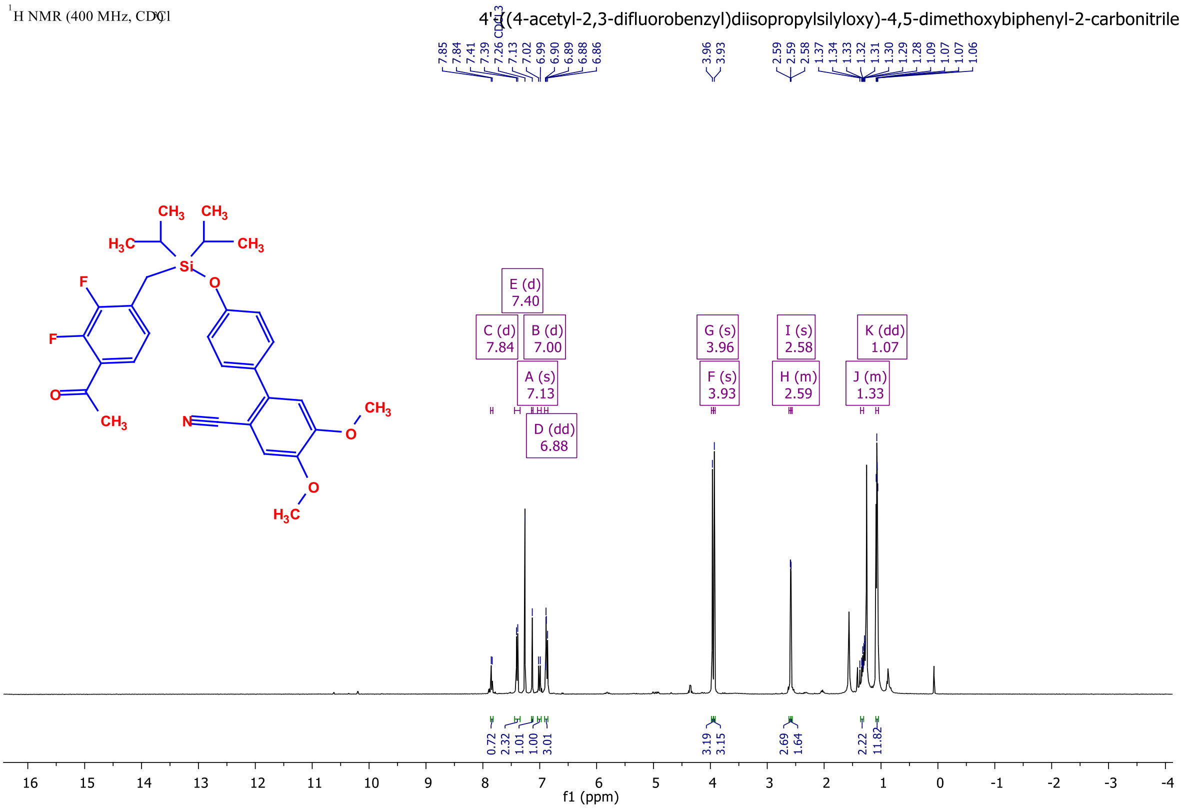

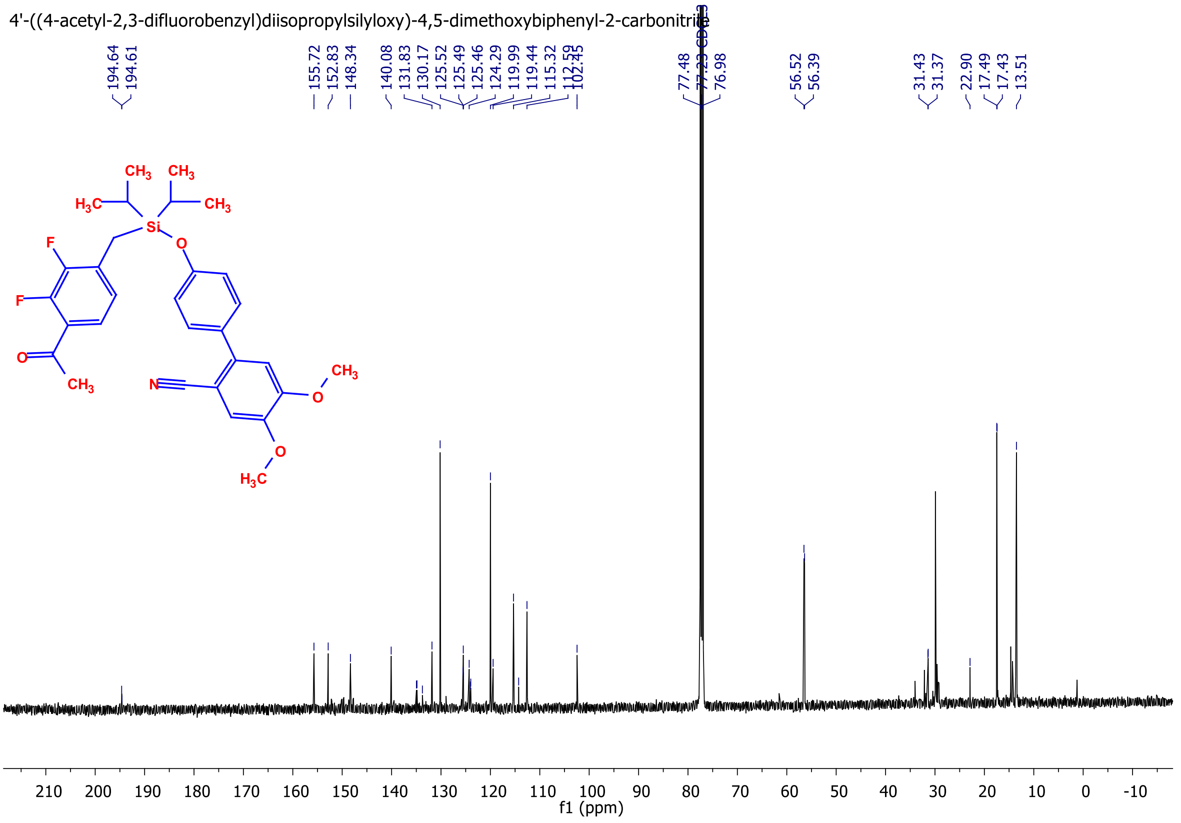

Supplementary Figure 48: ^{1}H and ^{13}C NMR (500 MHz, $CDCl_3$) of 4'-((4-acetyl-2-(trifluoromethyl)benzyl)diisopropylsilyloxy)-4,5-dimethoxybiphenyl-2-carbonitrile (3k):

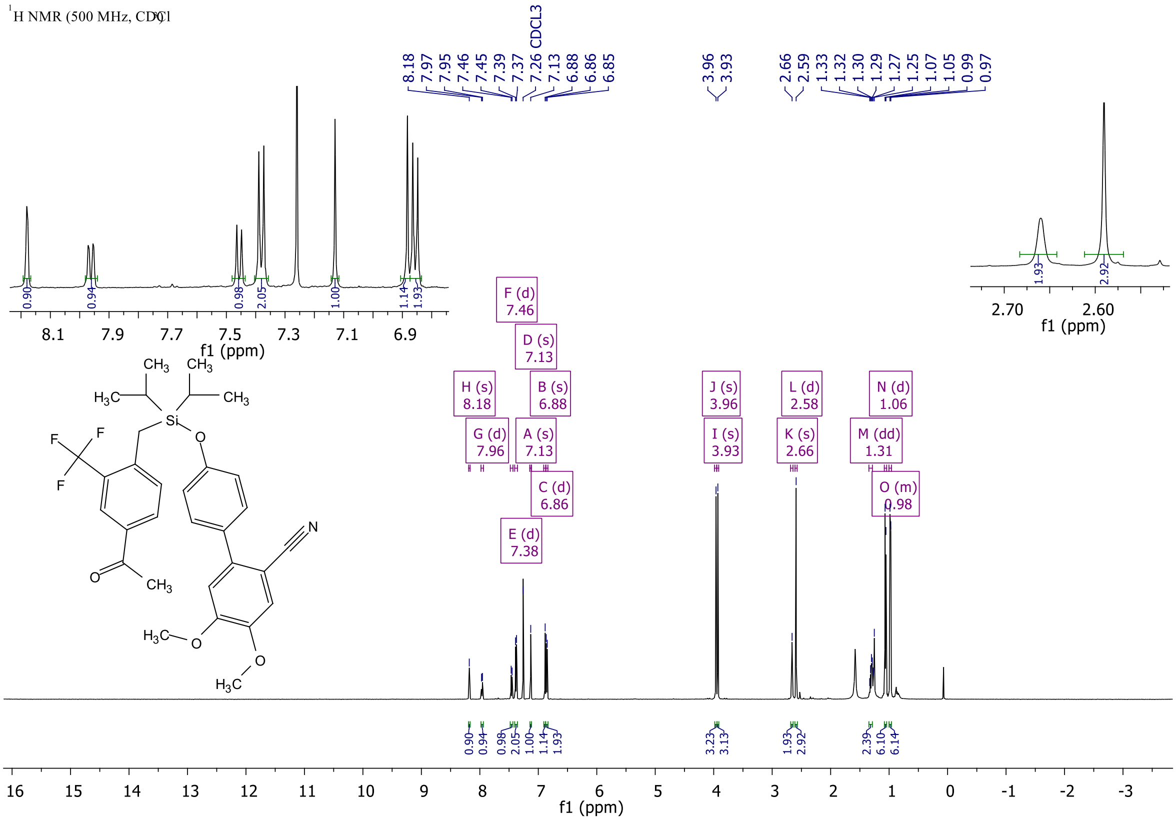

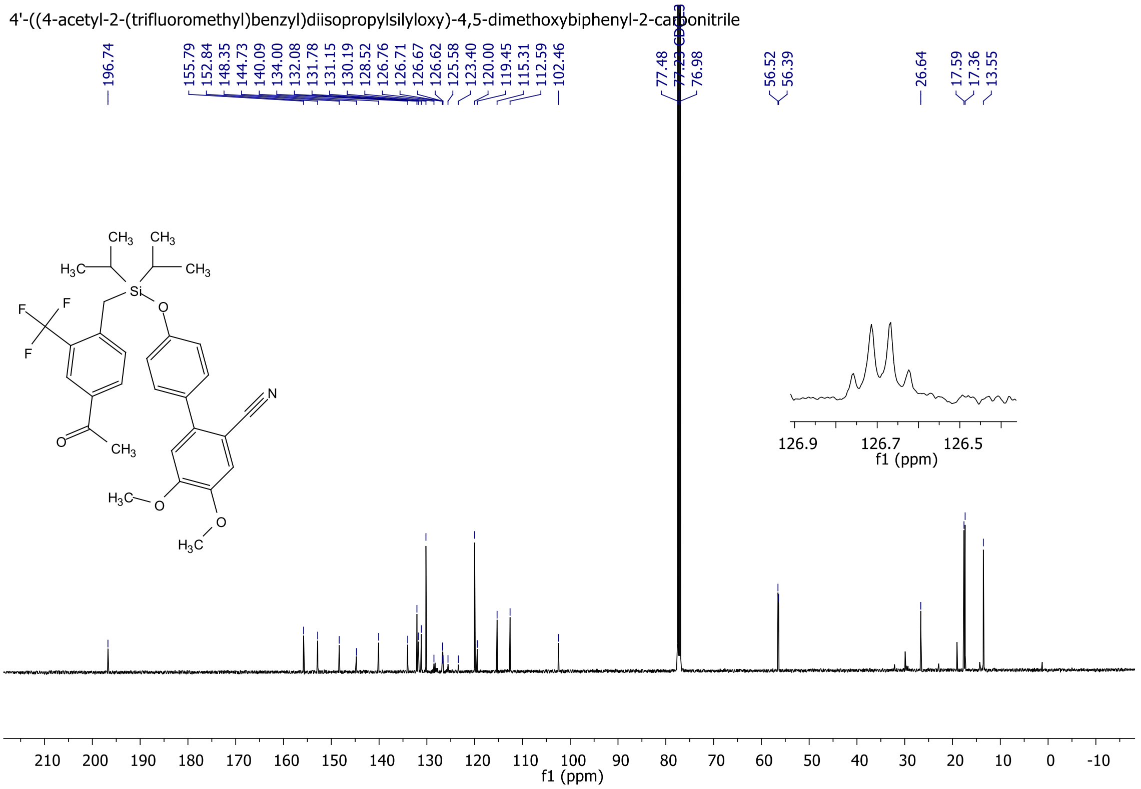

Characterizations: EWD and EDG Substituted Arene Scope

Supplementary Figure 49: ^{1}H and ^{13}C NMR (500 MHz, $CDCl_3$) of 4'-(((4-acetyl-2,3-difluoro-6-methoxybenzyl)diisopropylsilyl)oxy)-4,5-dimethoxy-[1,1'-biphenyl]-2-carbonitrile (4a):

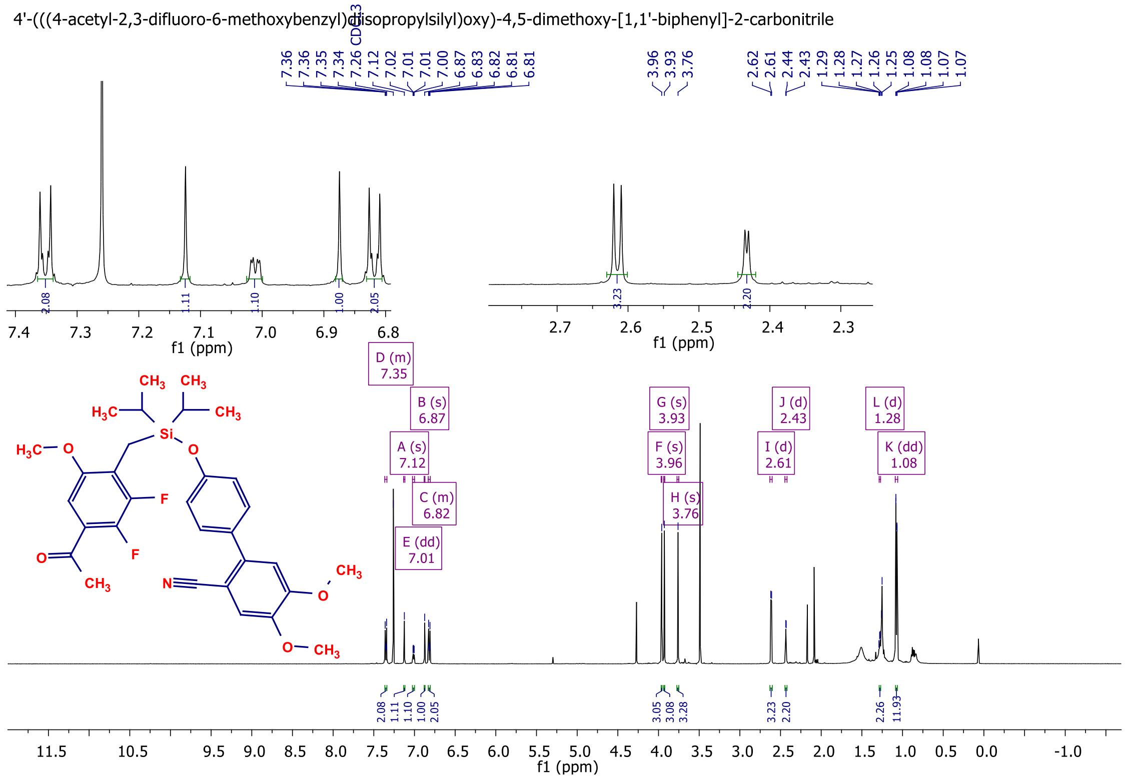

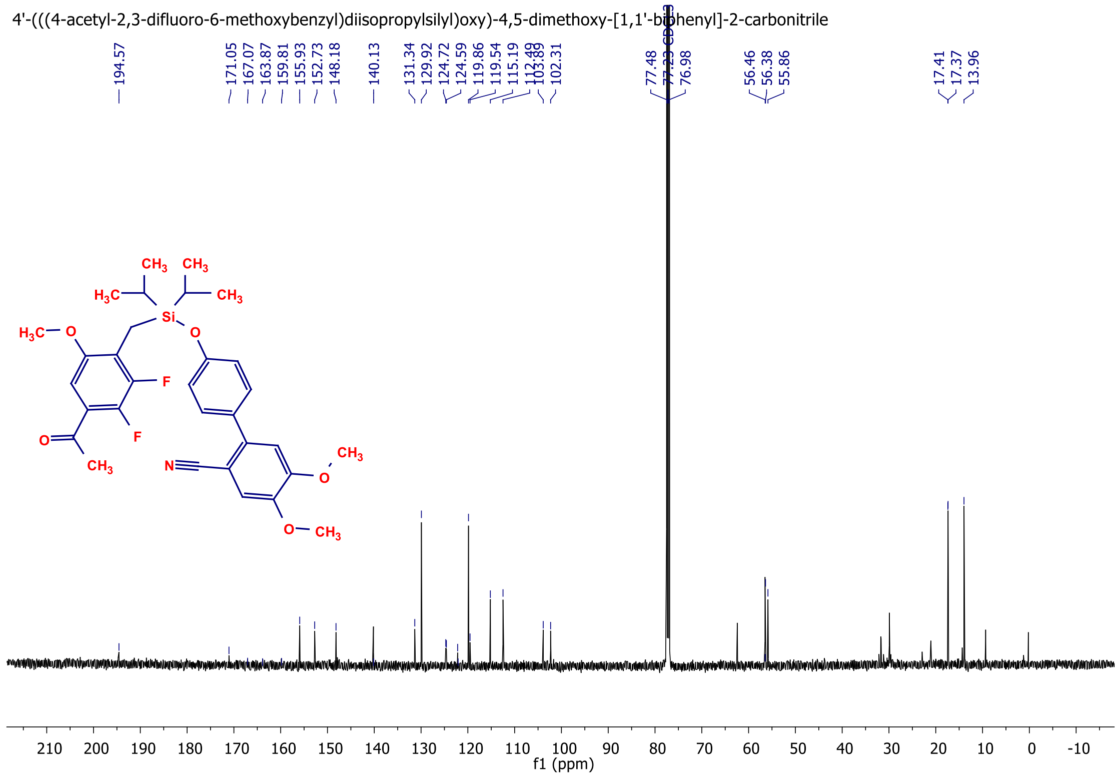

Supplementary Figure 50: ^{1}H and ^{13}C NMR (500 MHz, $CDCl_3$) of 4'-(((4-acetyl-2-fluoro-6-methoxybenzyl)diisopropylsilyl)oxy)-4,5-dimethoxy-[1,1'-biphenyl] -2-carbonitrile (4b):

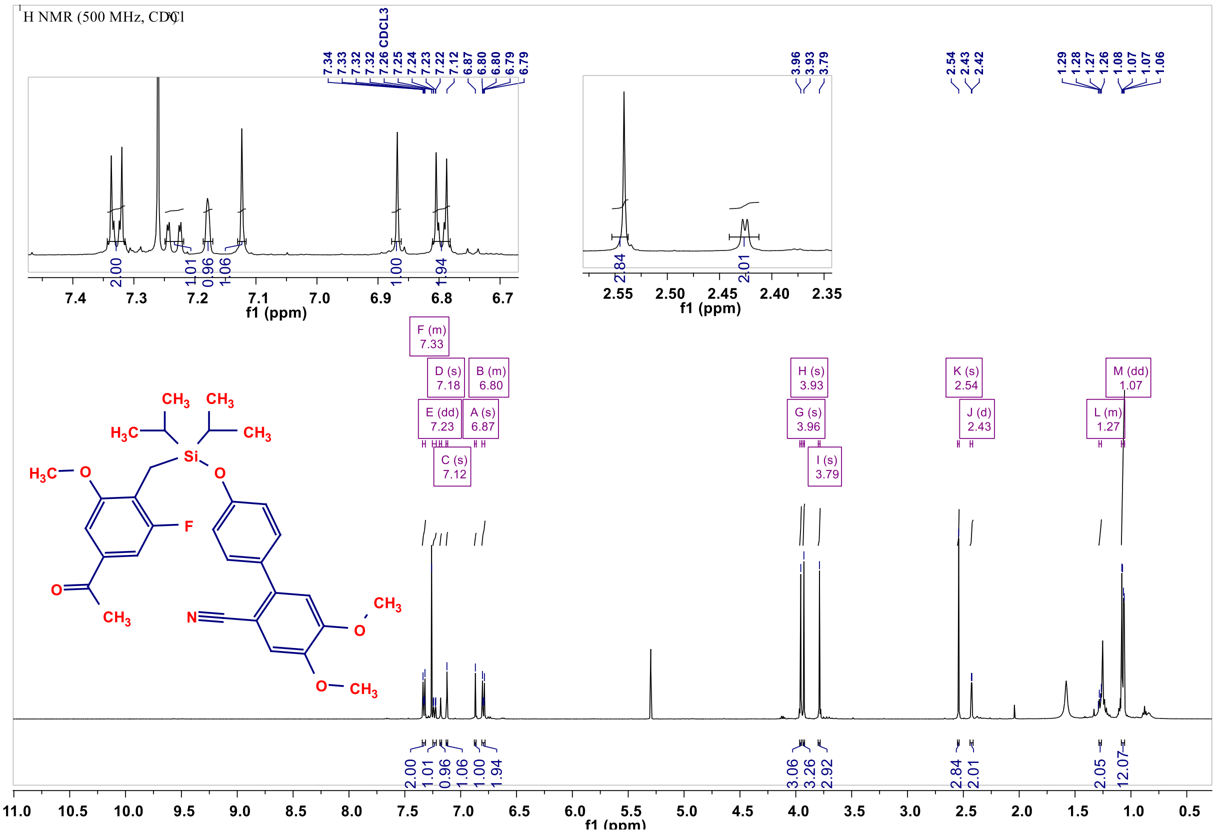

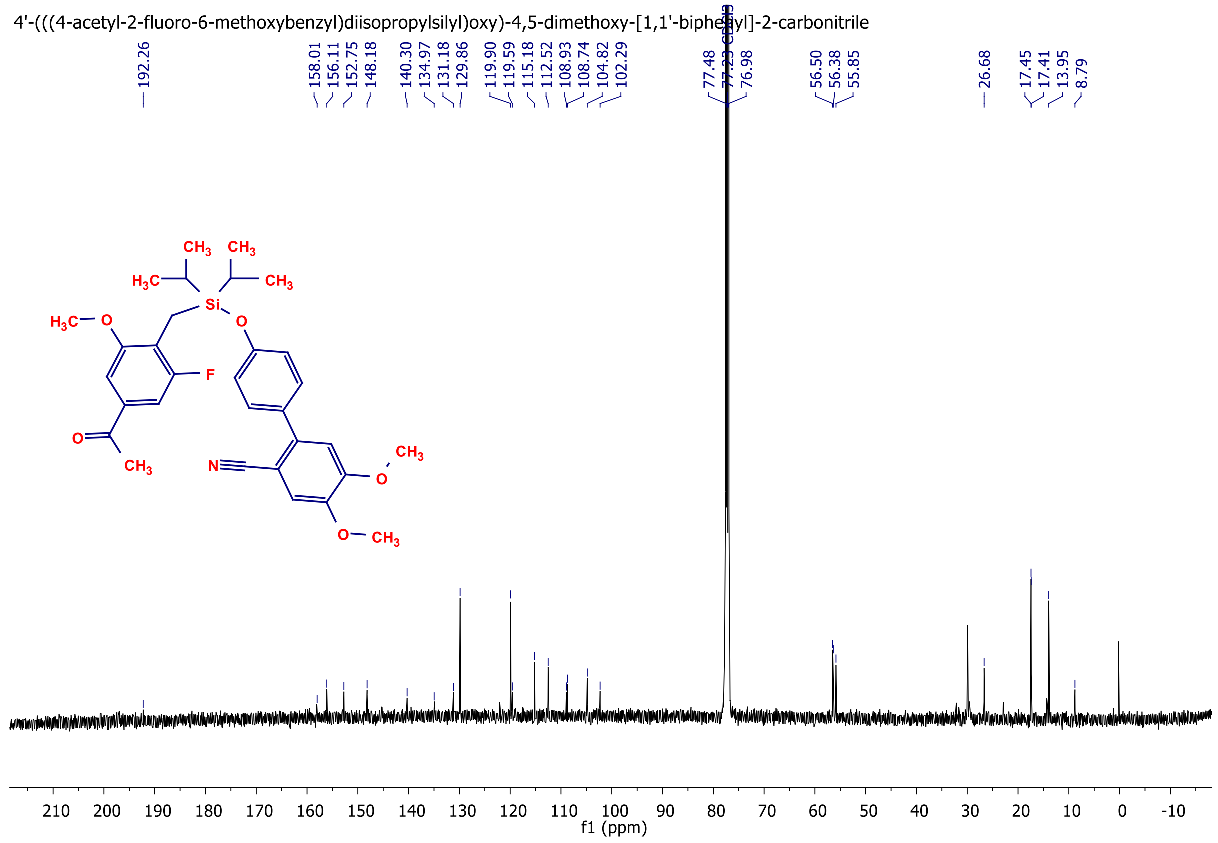

Supplementary Figure 51: ^{1}H and ^{13}C NMR (400 MHz, $CDCl_3$) of 4'-(((4-acetyl-5-fluoro-2-methoxybenzyl)diisopropylsilyl)oxy)-4,5-dimethoxy-[1,1'-biphenyl]-2-carbonitrile (4c):

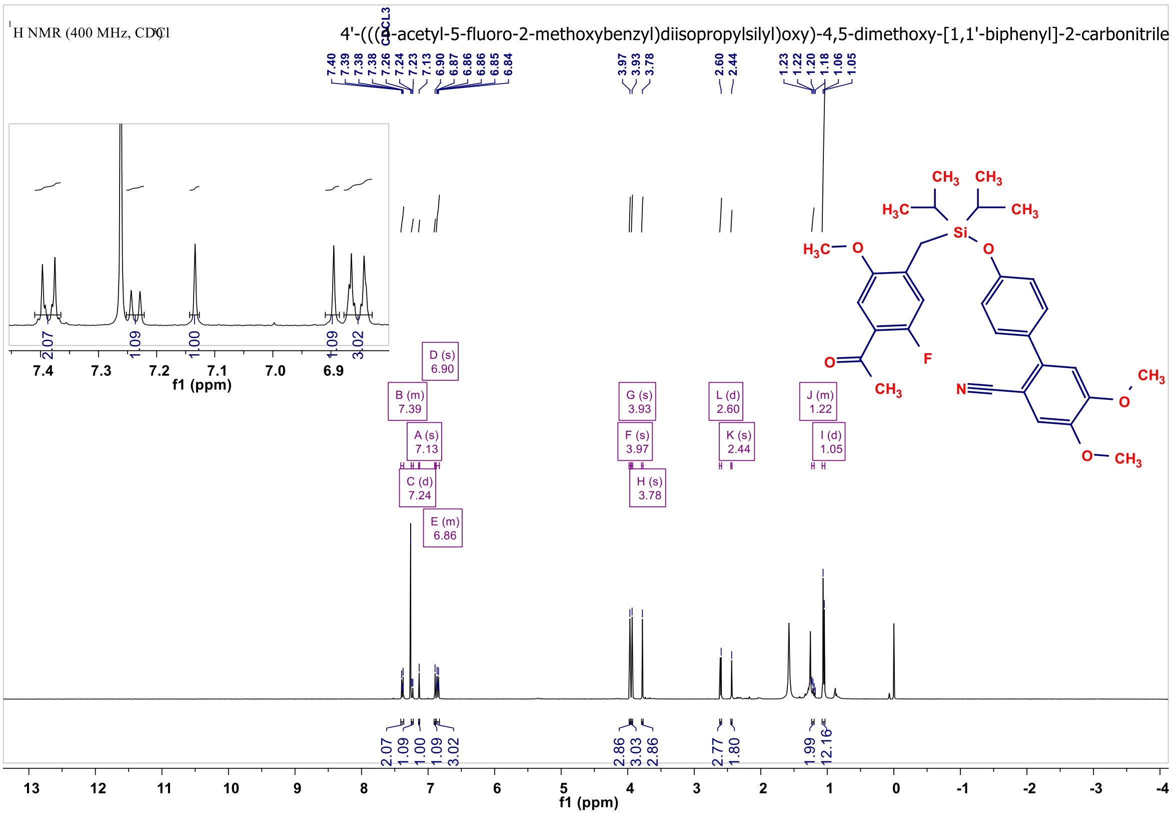

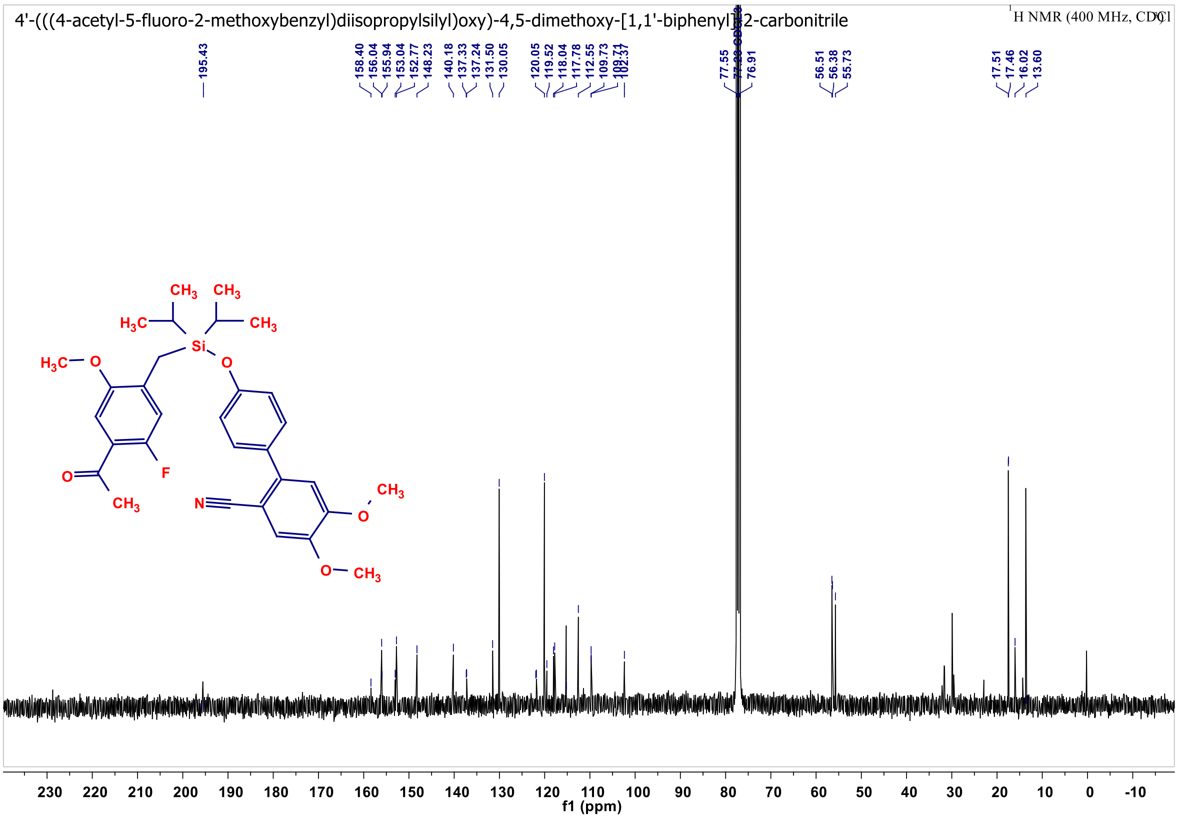

Supplementary Figure 52: ^{1}H and ^{13}C NMR (400 MHz, $CDCl_3$) of 4'-(((4-acetyl-2-methoxy-6-(trifluoromethyl)benzyl)diisopropylsilyl)oxy)-4,5-dimethoxy-[1,1'-biphenyl]-2-carbonitrile (4d):

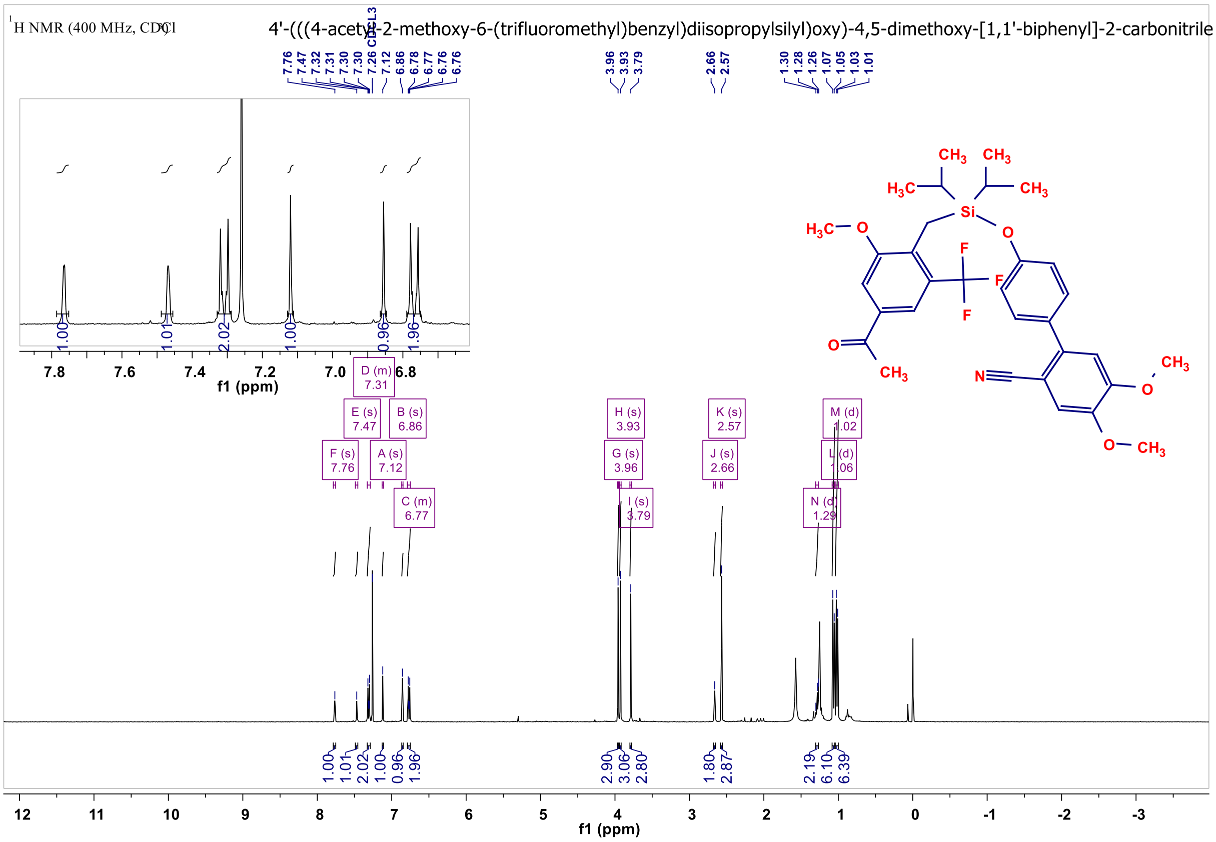

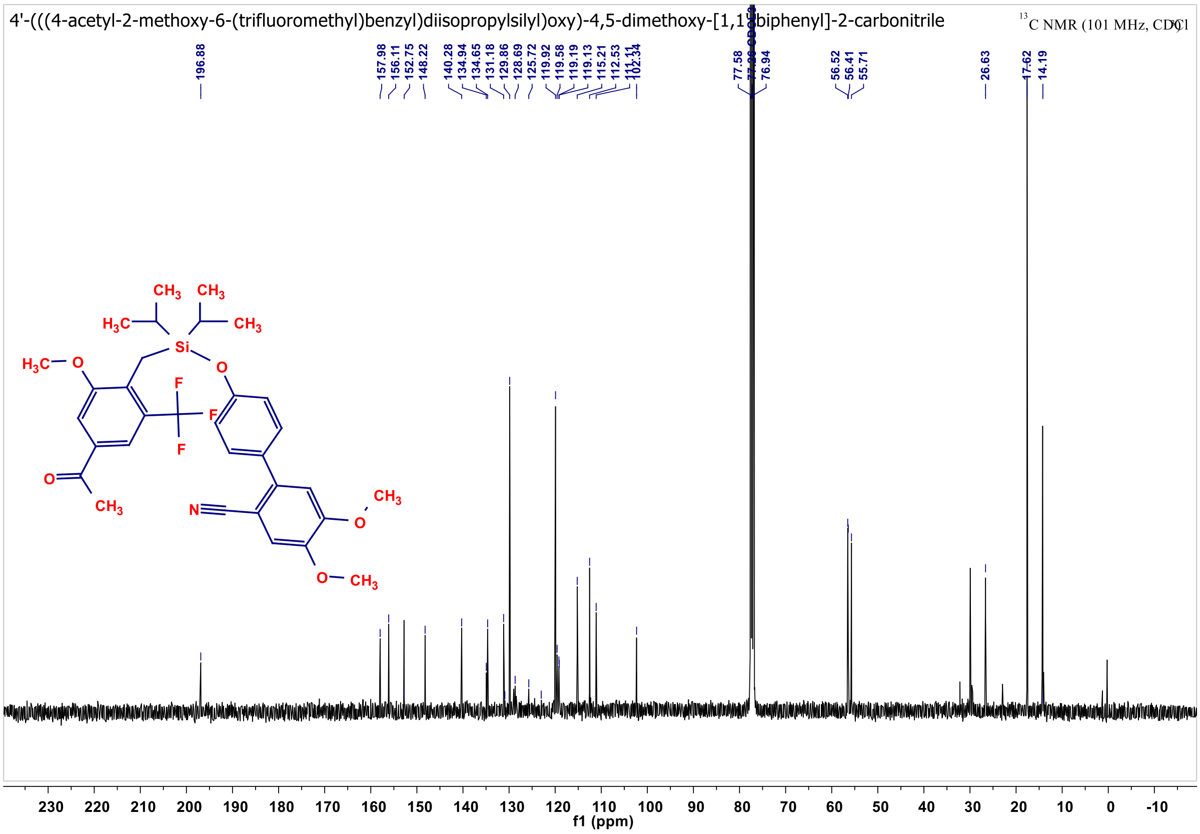

Supplementary Figure 53: ^{1}H and ^{13}C NMR (500 MHz, $CDCl_3$) of 4'-(((4-acetyl-3-fluoro-2-methylbenzyl)diisopropylsilyl)oxy)-4,5-dimethoxy-[1,1'-biphenyl]-2-carbonitrile (4e):

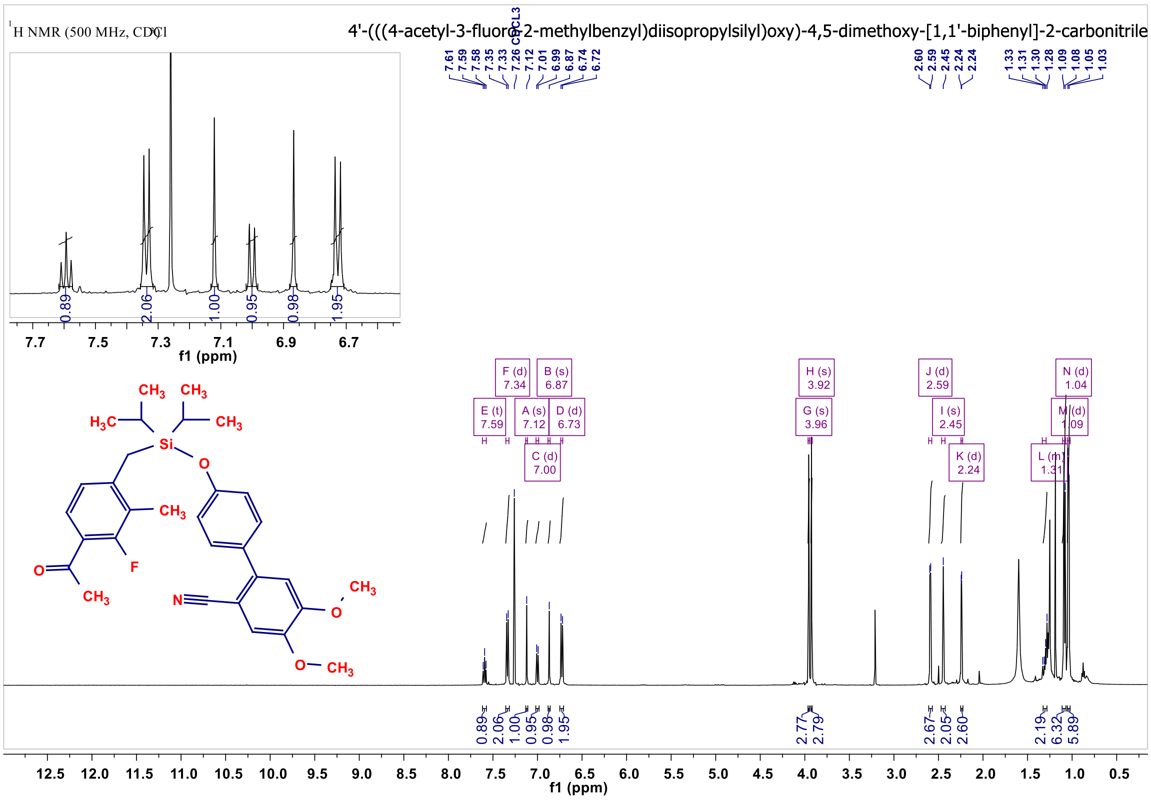

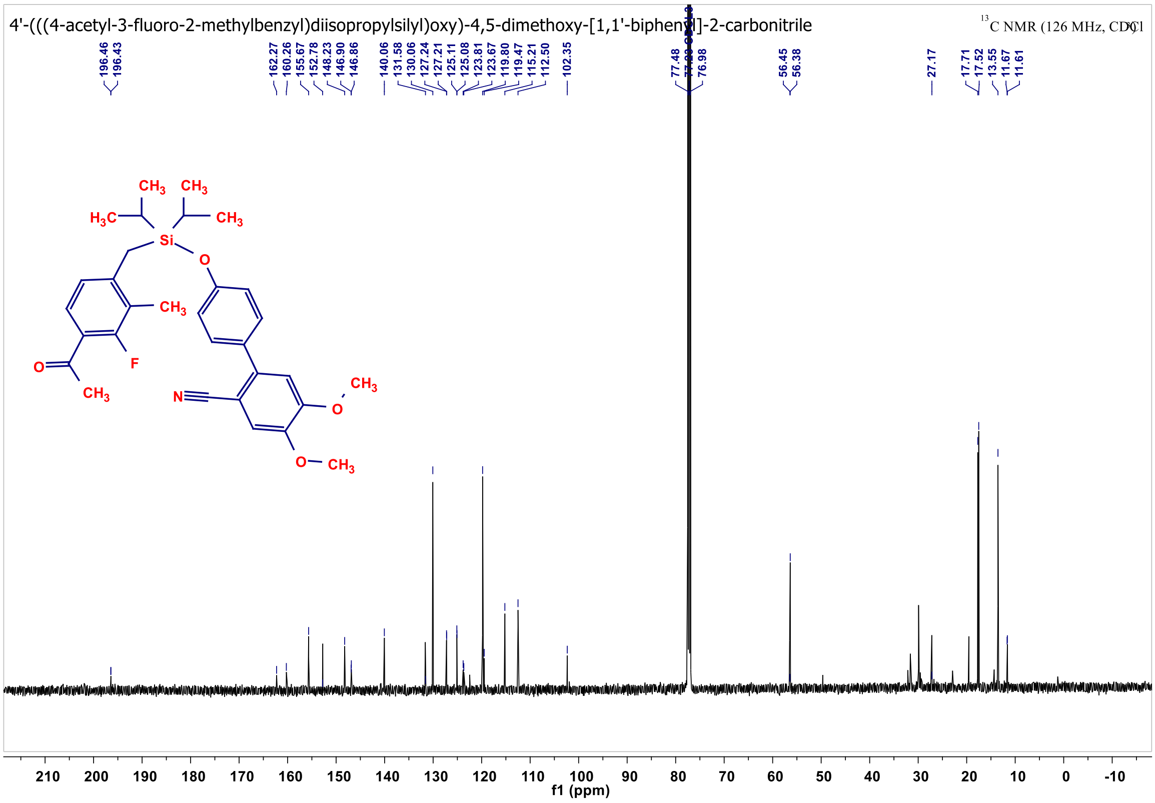

Supplementary Figure 54: ^{1}H and ^{13}C NMR (500 MHz, $CDCl_3$) of 4'-(((4-acetyl-3-fluoro-2-(trifluoromethyl)benzyl)diisopropylsilyl)oxy)-4,5-dimethoxy-[1,1'-biphenyl]-2-carbonitrile (4f):

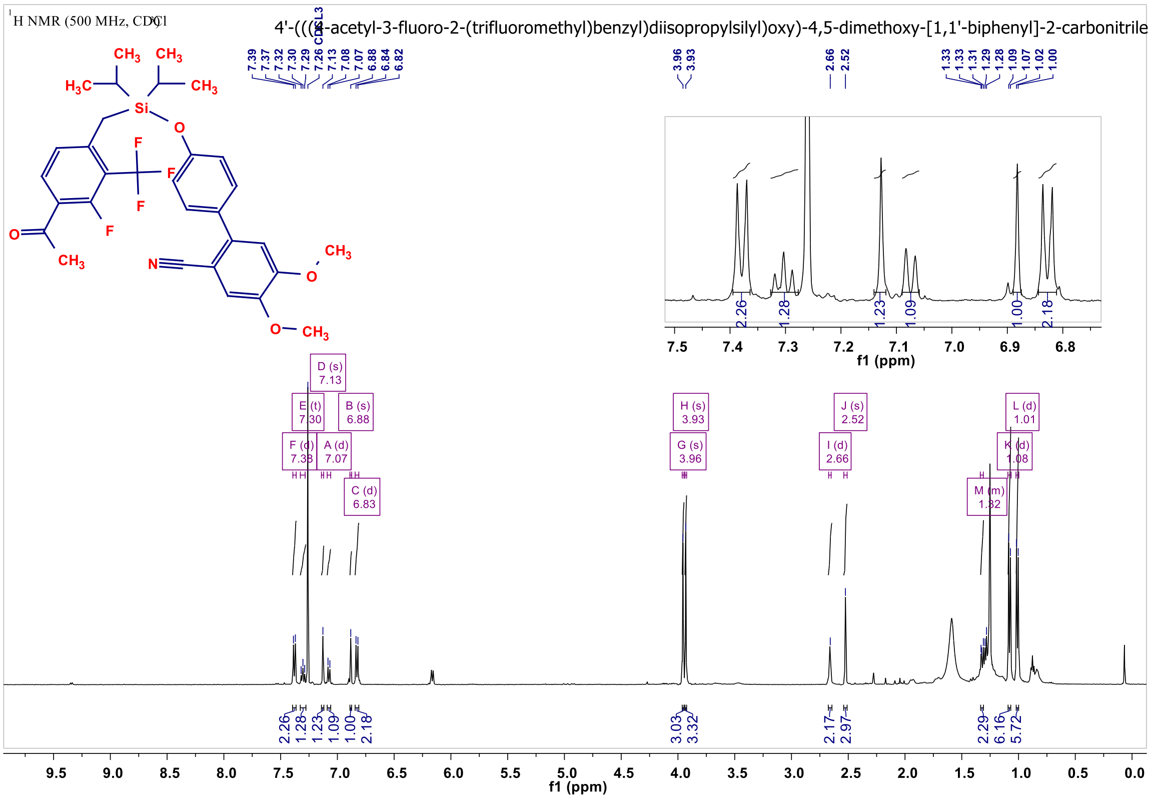

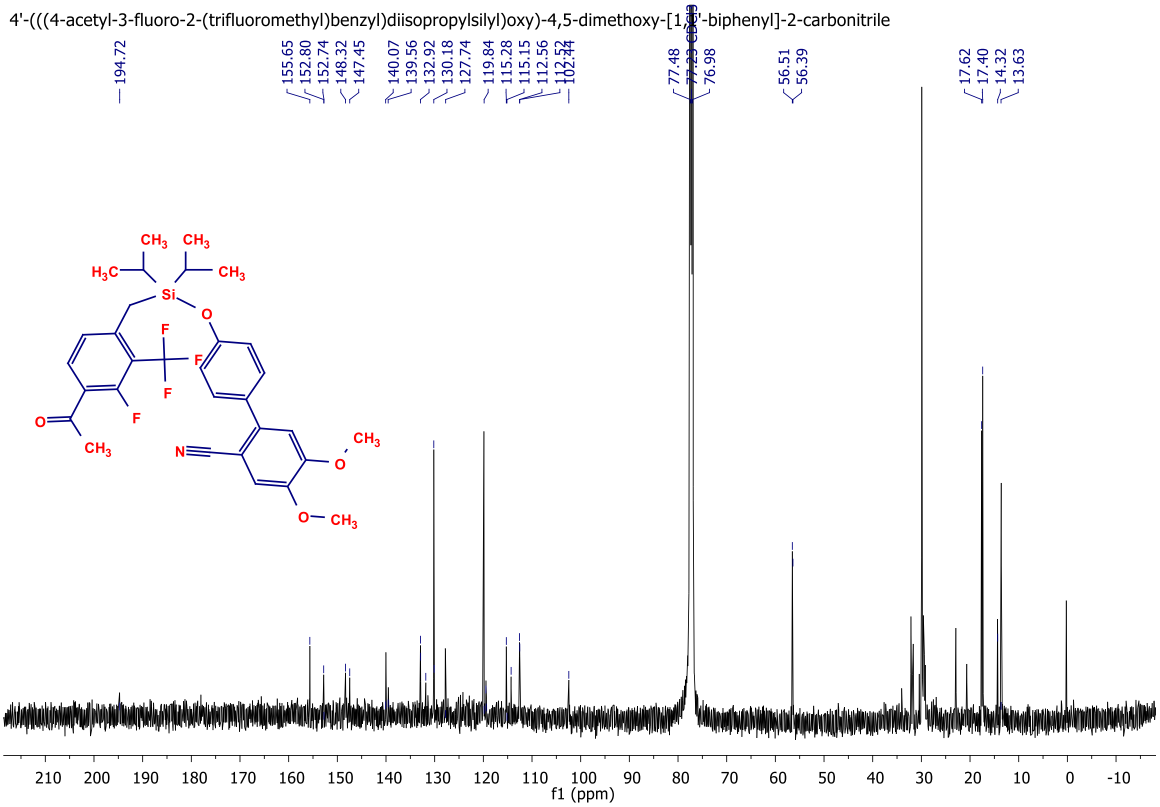

Supplementary Figure 55: ^{1}H and ^{13}C NMR (400 MHz, $CDCl_3$) of 4'-(((4-acetyl-5-fluoro-2-(trifluoromethoxy)benzyl)diisopropylsilyl)oxy)-4,5-dimethoxy-[1,1'-biphenyl]-2-carbonitrile (4g):

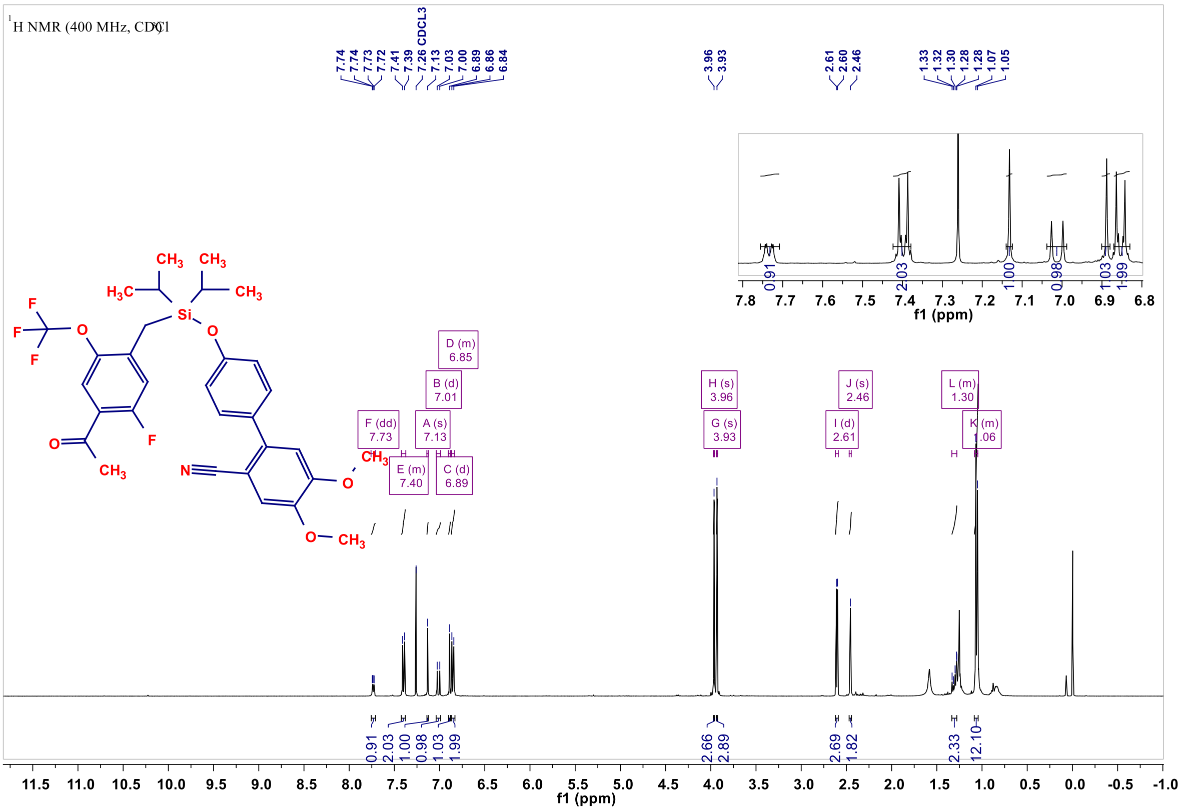

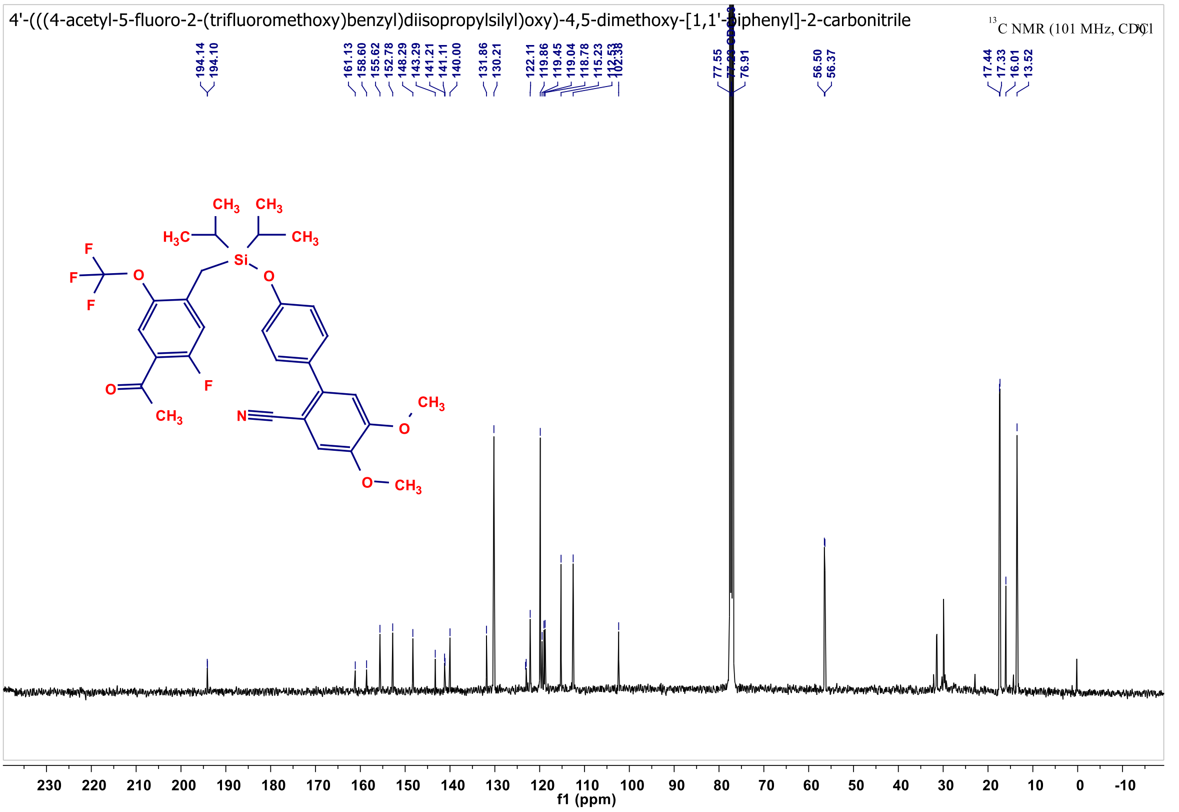

Supplementary Figure 56: ^{1}H and ^{13}C NMR (500 MHz, $CDCl_3$) of 4'-(((4-acetyl-2-chloro-5-(difluoromethoxy)benzyl)diisopropylsilyl)oxy)-4,5-dimethoxy-[1,1'-biphenyl]-2-carbonitrile (4h):

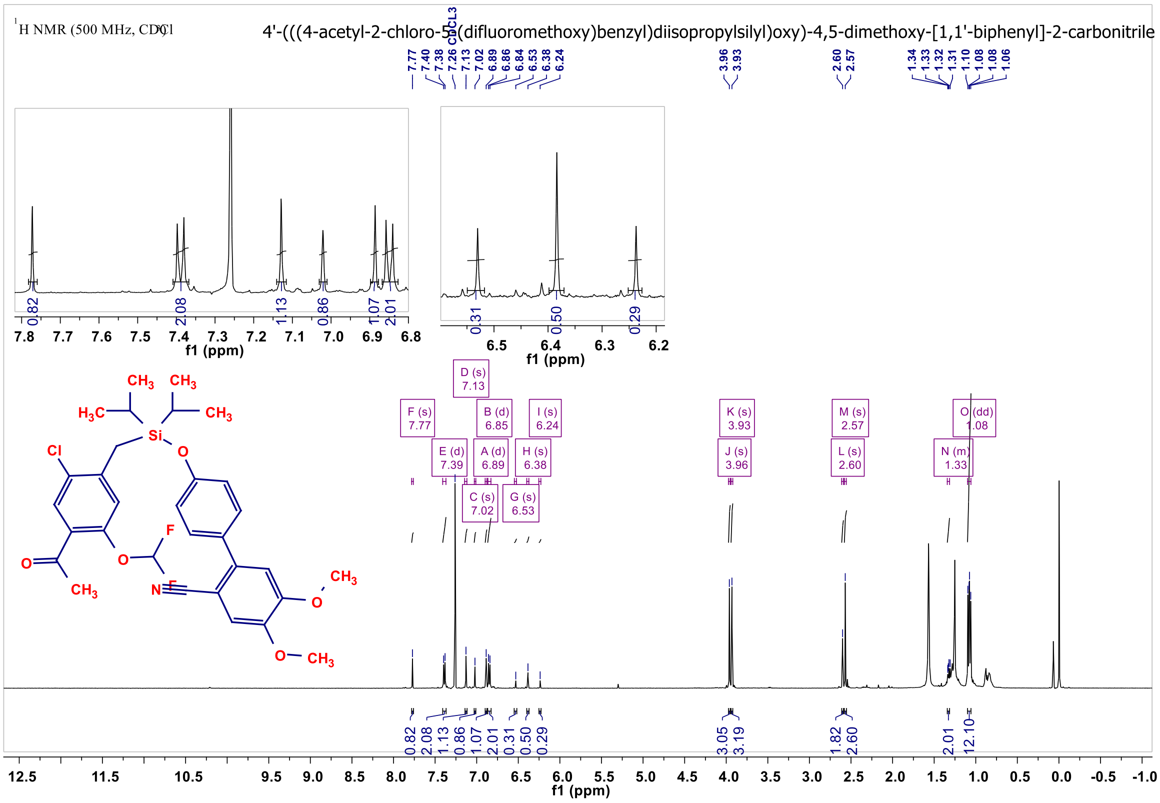

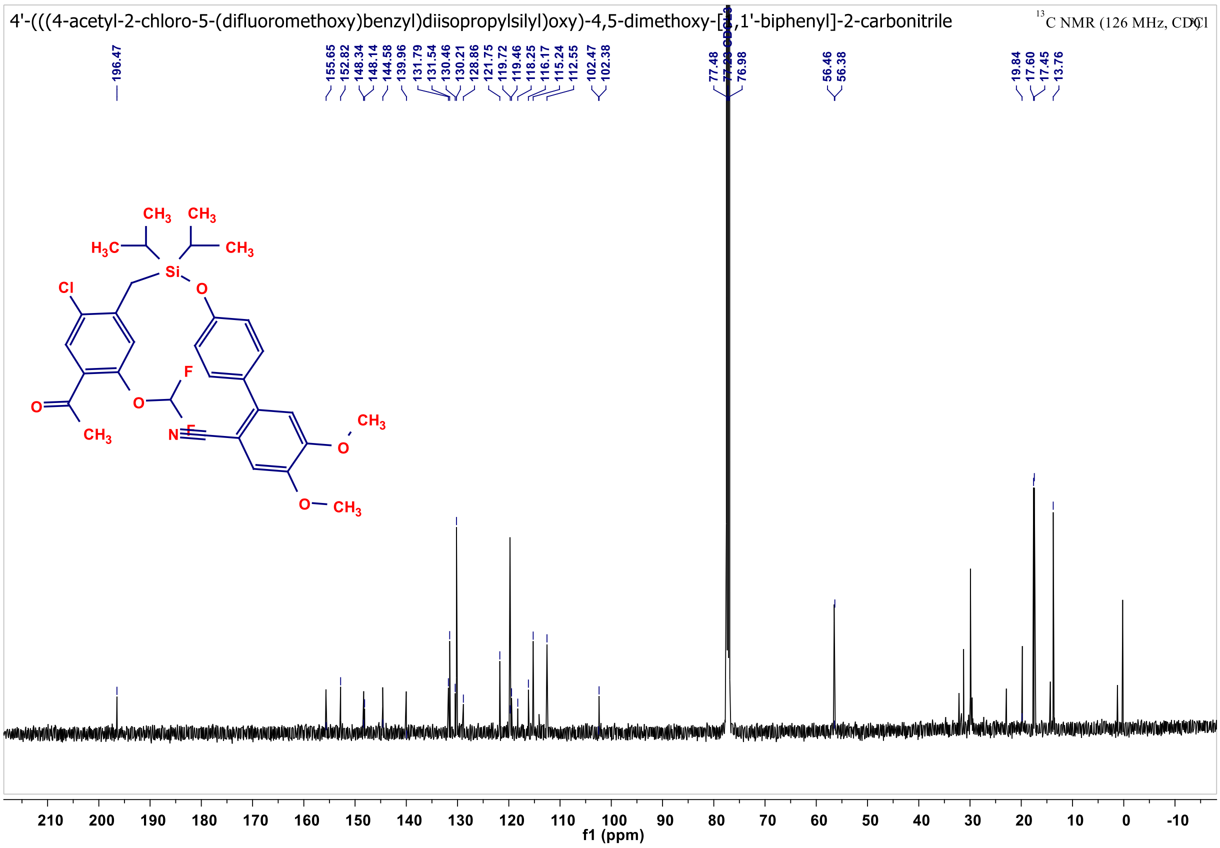

Supplementary Figure 57: ^{1}H and ^{13}C NMR (500 MHz, $CDCl_3$) of 4'-((4-acetyl-3-(trifluoromethyl)benzyl)diisopropylsilyloxy)-4,5-dimethoxybiphenyl-2-carbonitrile (4i):

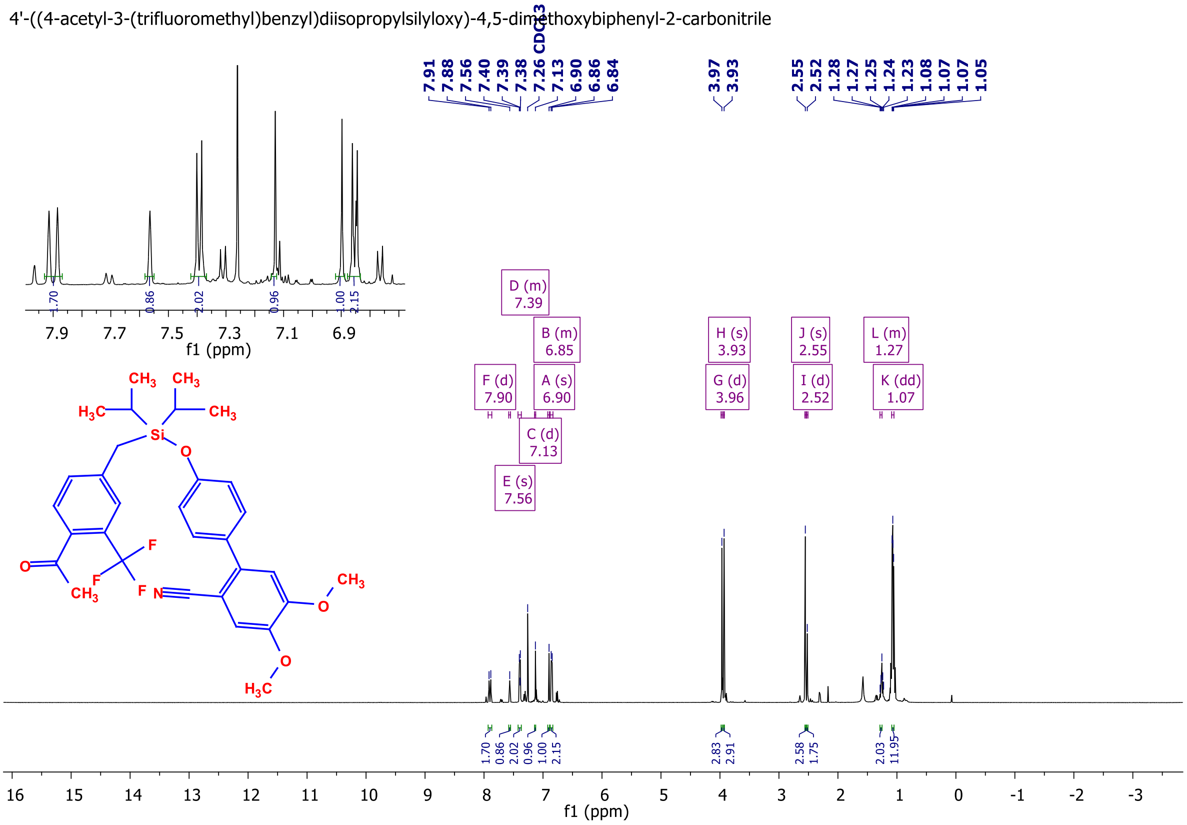

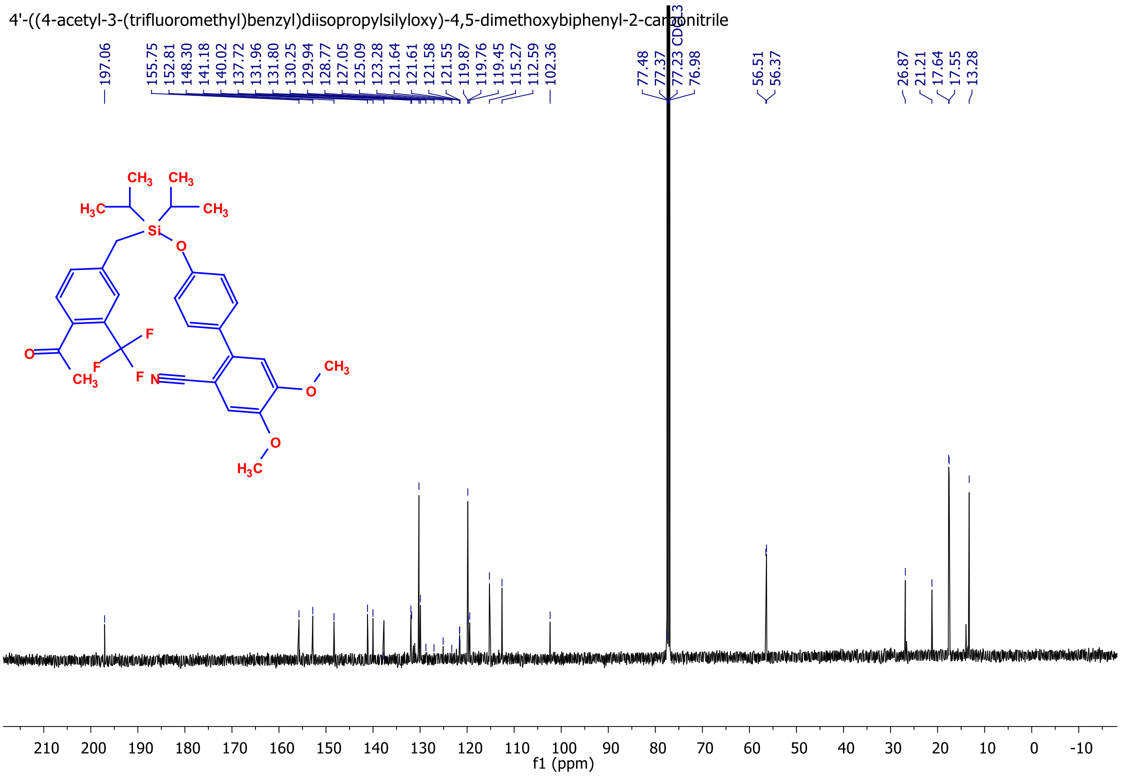

Supplementary Figure 58: ^{1}H and ^{13}C NMR (400 MHz, $CDCl_3$) of 4'-((4-acetyl-5-fluoro-2-methylbenzyl)diisopropylsilyloxy)-4,5-dimethoxybiphenyl-2-carbonitrile (4j):

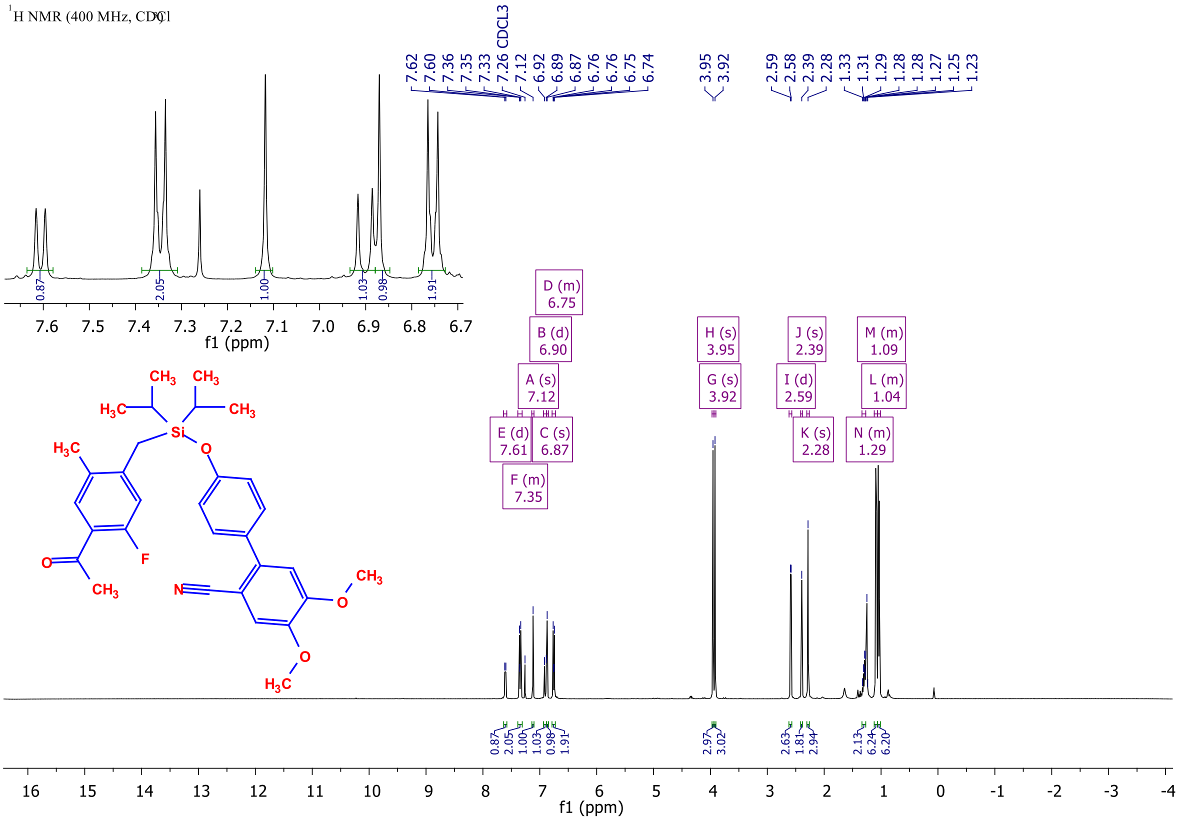

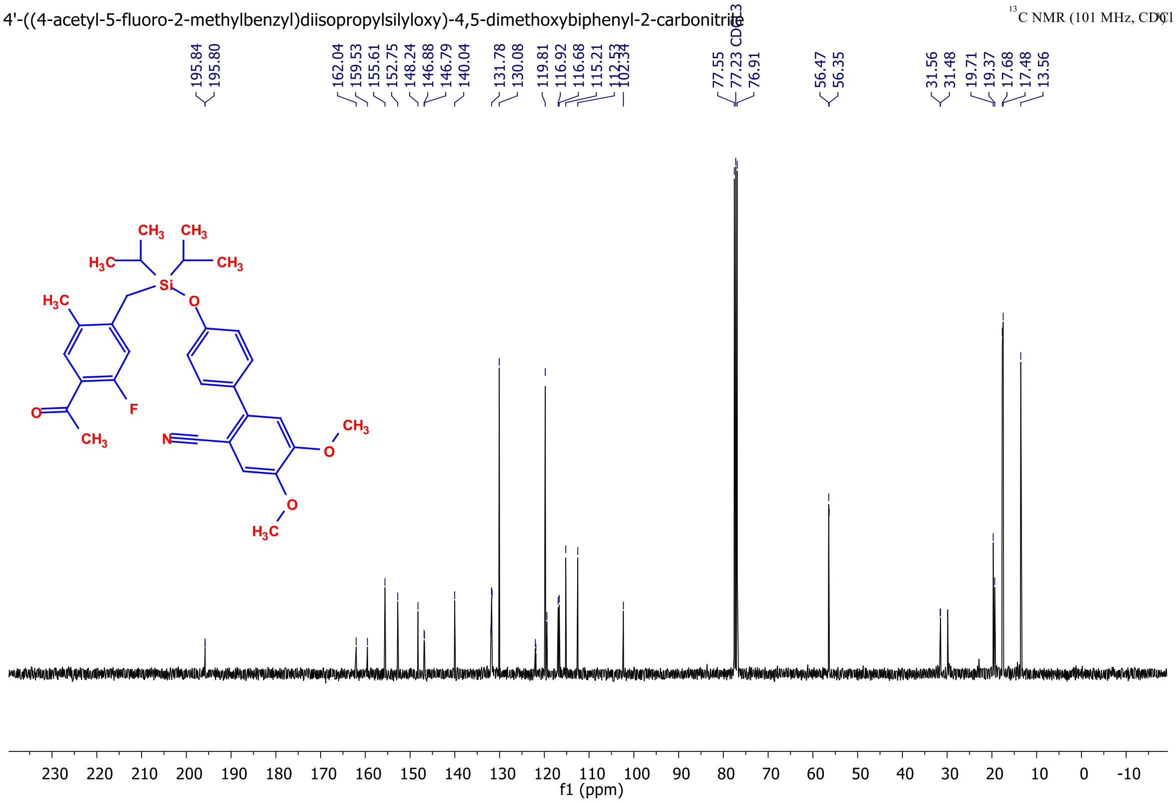

Supplementary Figure 59: ^{1}H and ^{13}C NMR (500 MHz, $CDCl_3$) of 4'-((1-(4-acetylphenyl)ethyl)diisopropylsilyloxy)-4,5-dimethoxybiphenyl-2-carbonitrile (4k):

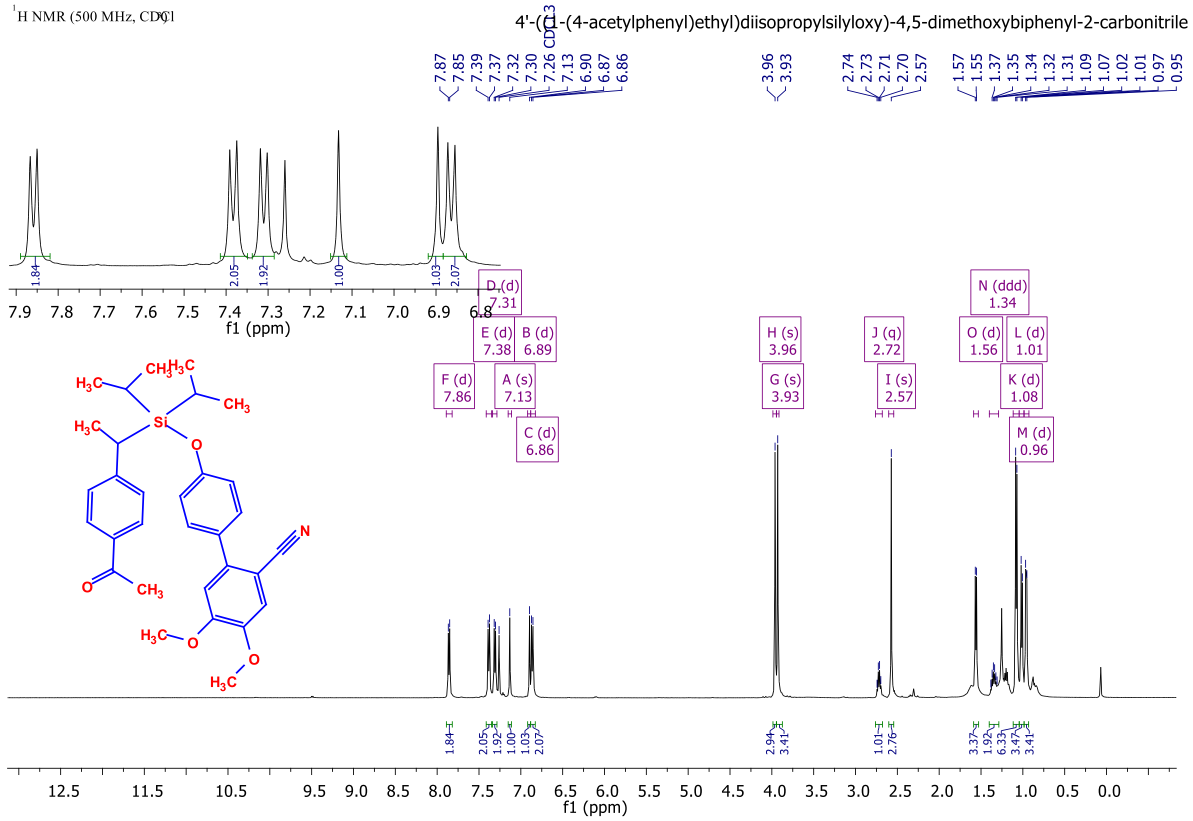

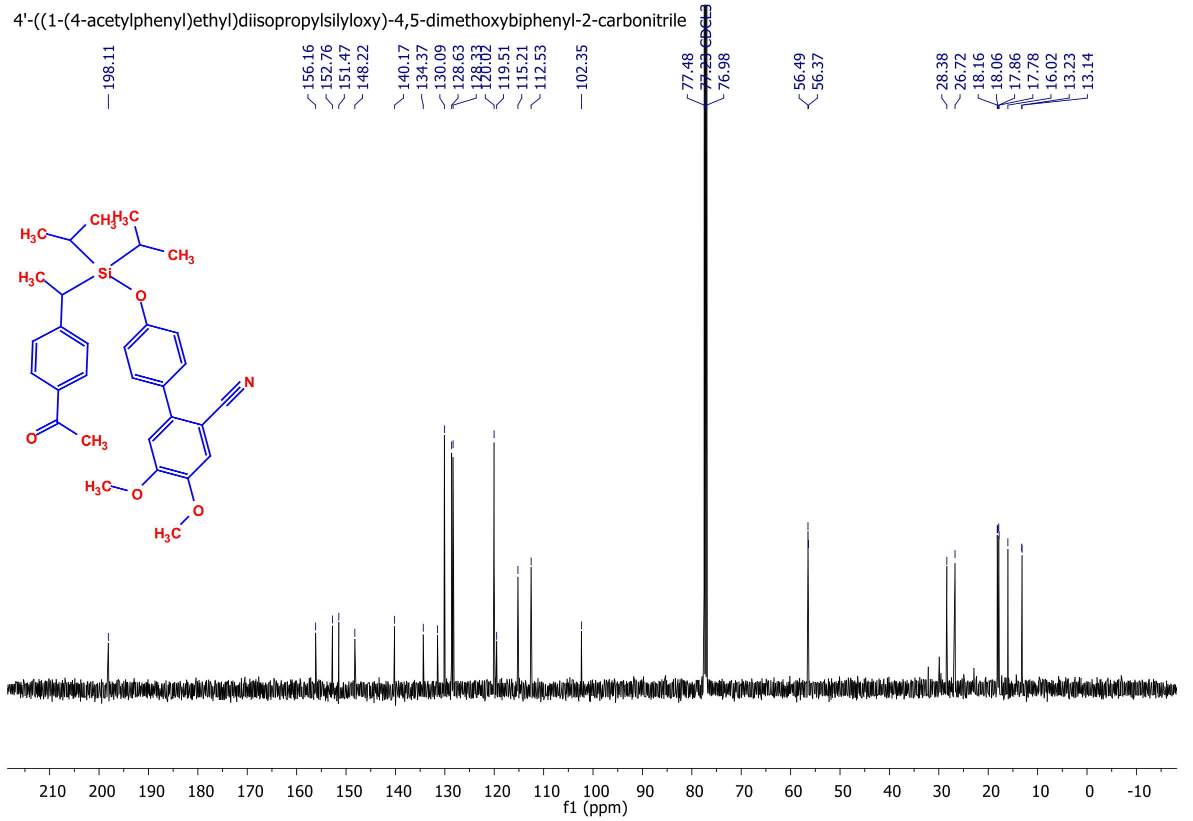

Supplementary Figure 60: ^{1}H and ^{13}C NMR (500 MHz, $CDCl_3$) of 4'-(((4-acetylphenyl)(4-chlorophenyl)methyl)diisopropylsilyloxy)-4,5-dimethoxybiphenyl-2-carbonitrile (4l):

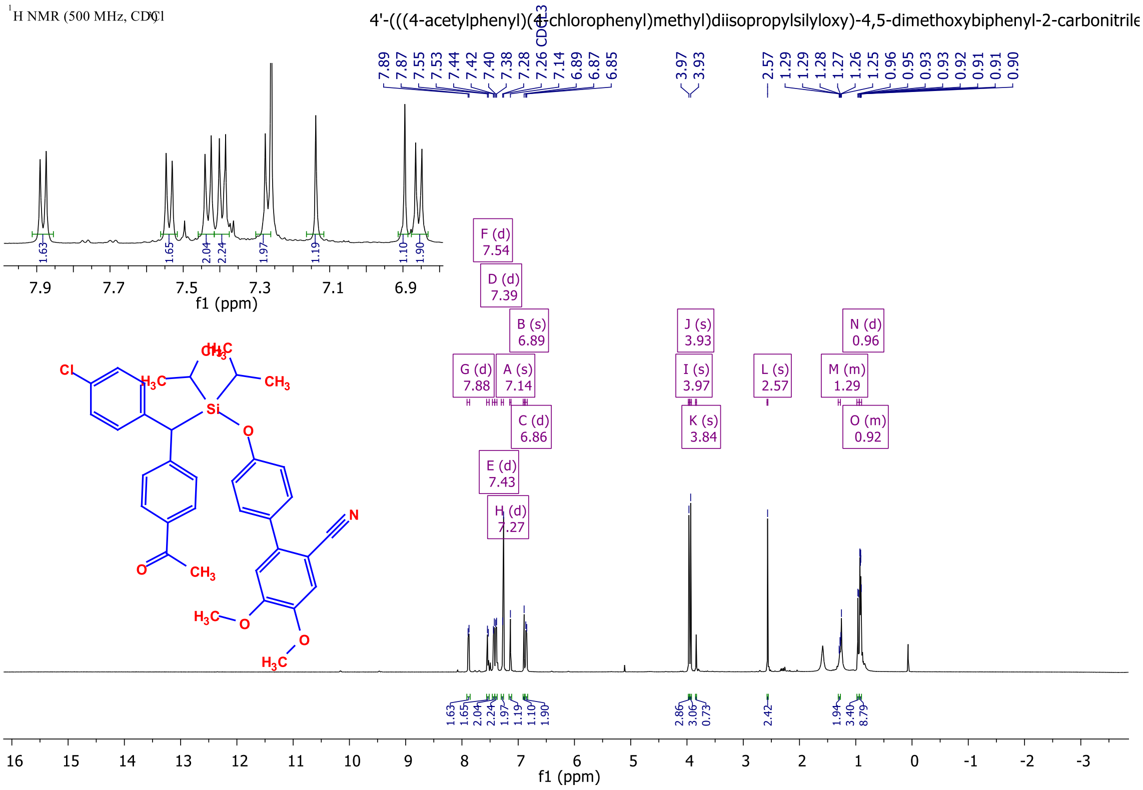

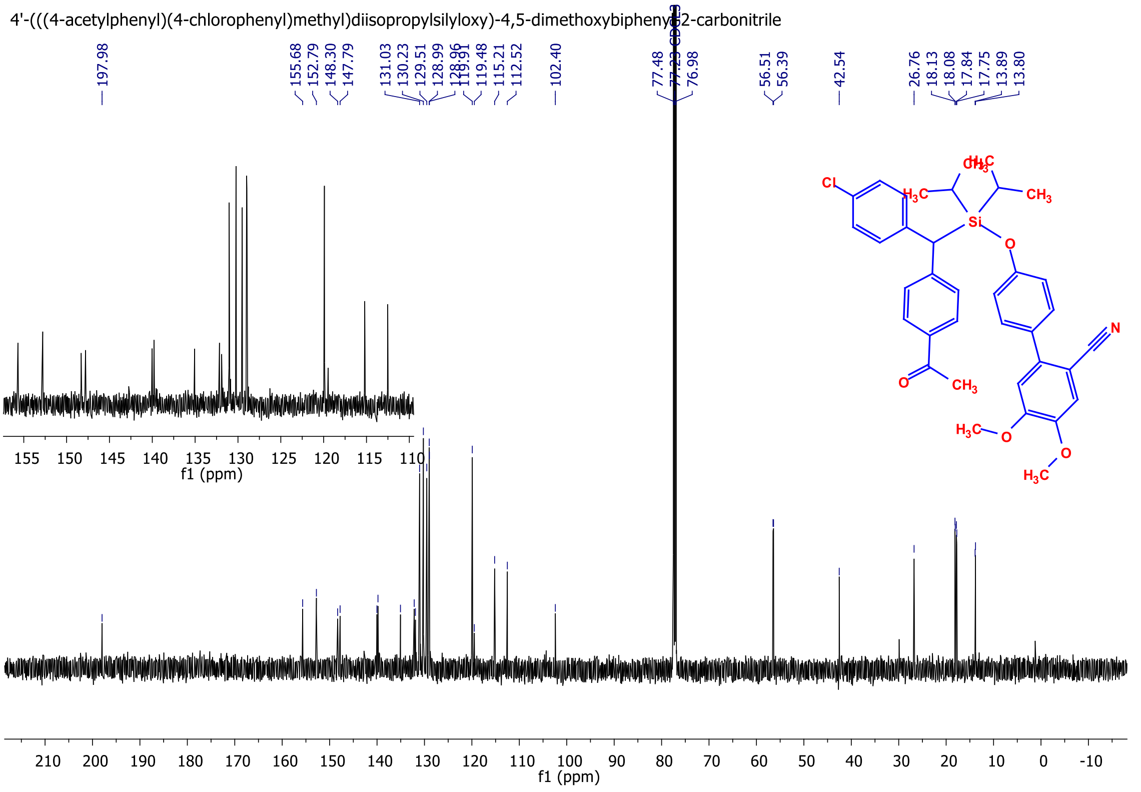

Supplementary Figure 61: ^{1}H and ^{13}C NMR (500 MHz, $CDCl_3$) of 4'-(((4-acetylphenyl)(phenyl)methyl)diisopropylsilyloxy)-4,5-dimethoxybiphenyl-2-carbonitrile (4m):

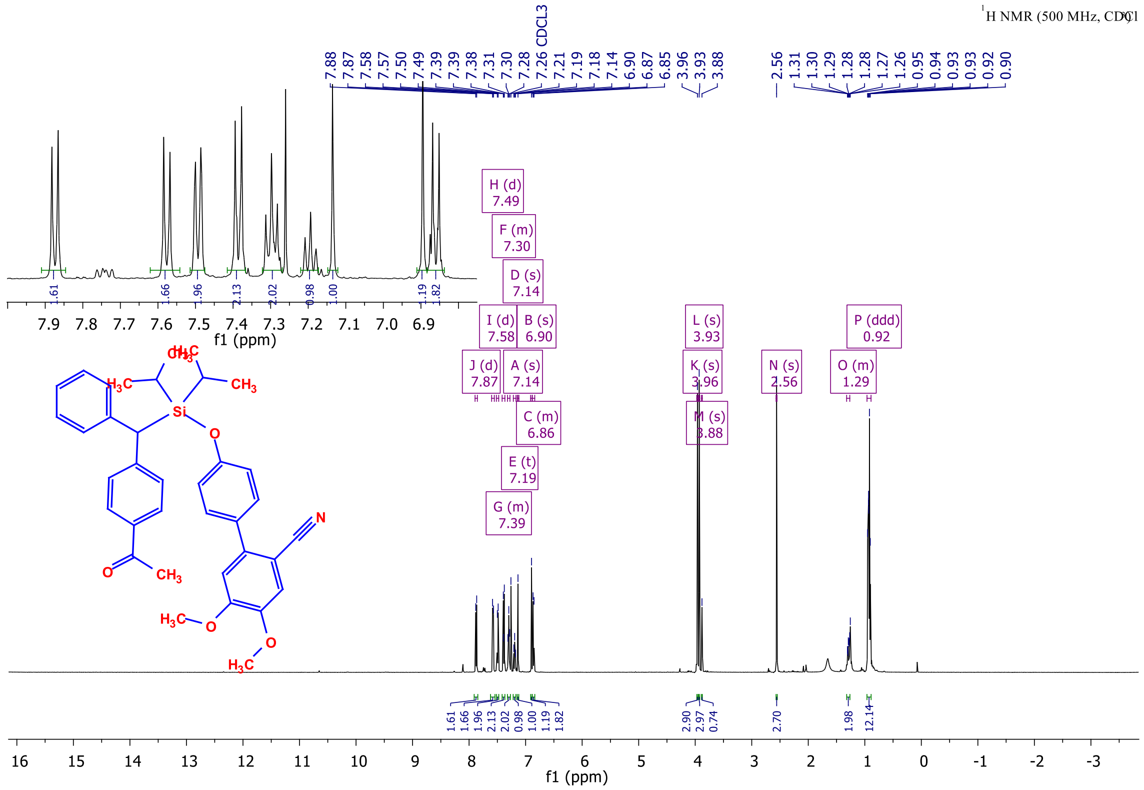

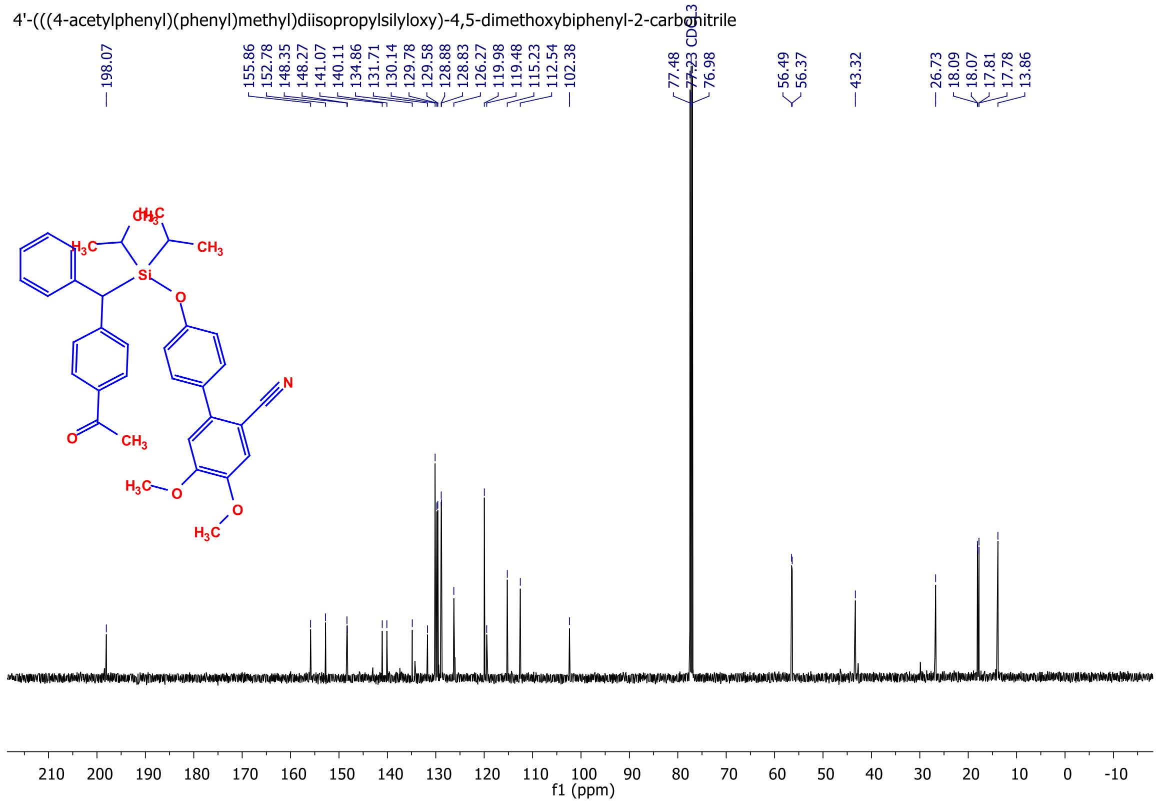

Characterizations: Vinyl Ether Variation Scope

Supplementary Figure 62: ^{1}H and ^{13}C NMR (500 MHz, $CDCl_3$) of 4'-((diisopropyl(4-propionylbenzyl)silyl)oxy)-4,5-dimethoxy-[1,1'-biphenyl]-2-carbonitrile (1q):

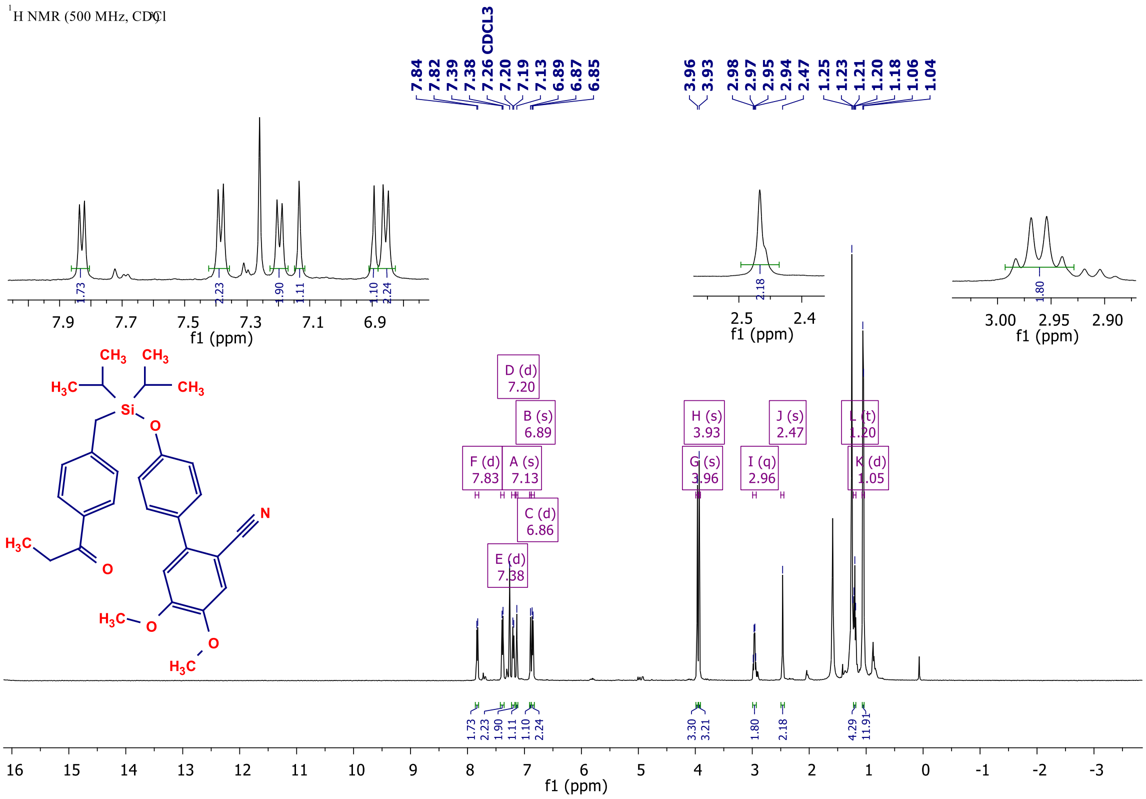

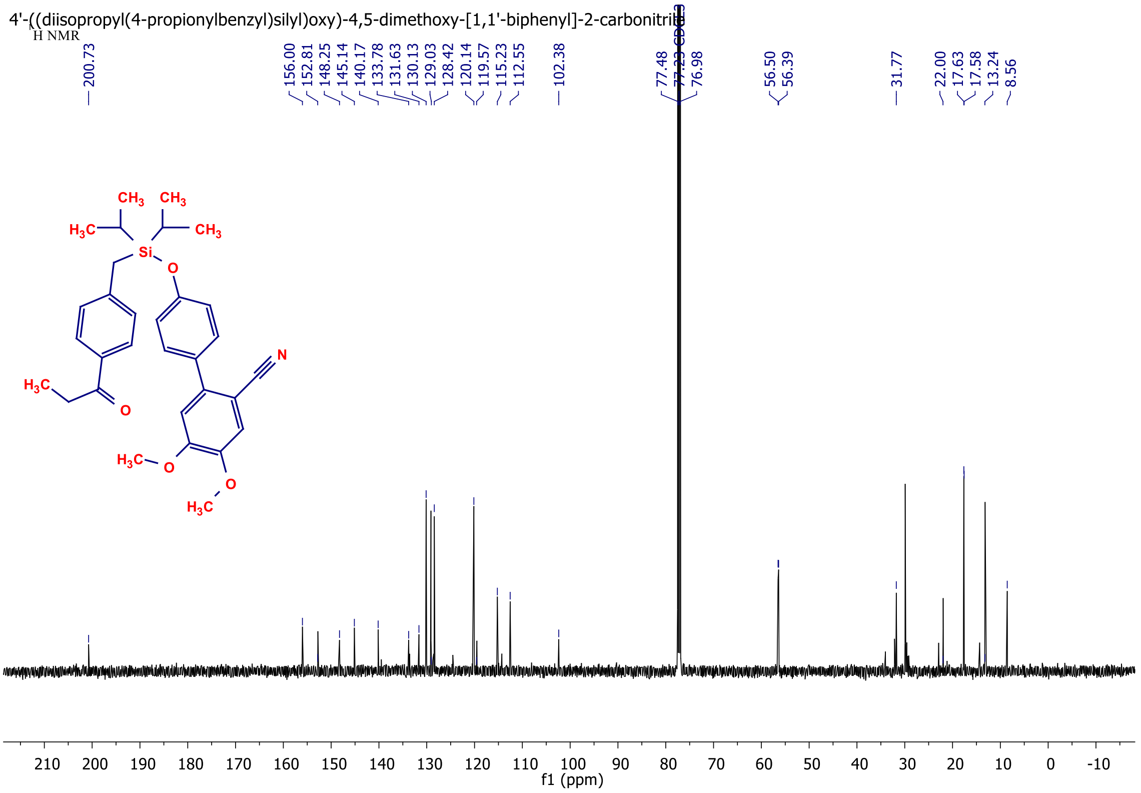

Supplementary Figure 63: ^{1}H and ^{13}C NMR (400 MHz, $CDCl_3$) of 4'-(((4-(2-(4-fluoro-2-methylphenyl)acetyl)benzyl)diisopropylsilyl)oxy)-4,5-dimethoxy-[1,1'-biphenyl]-2-carbonitrile (1r):

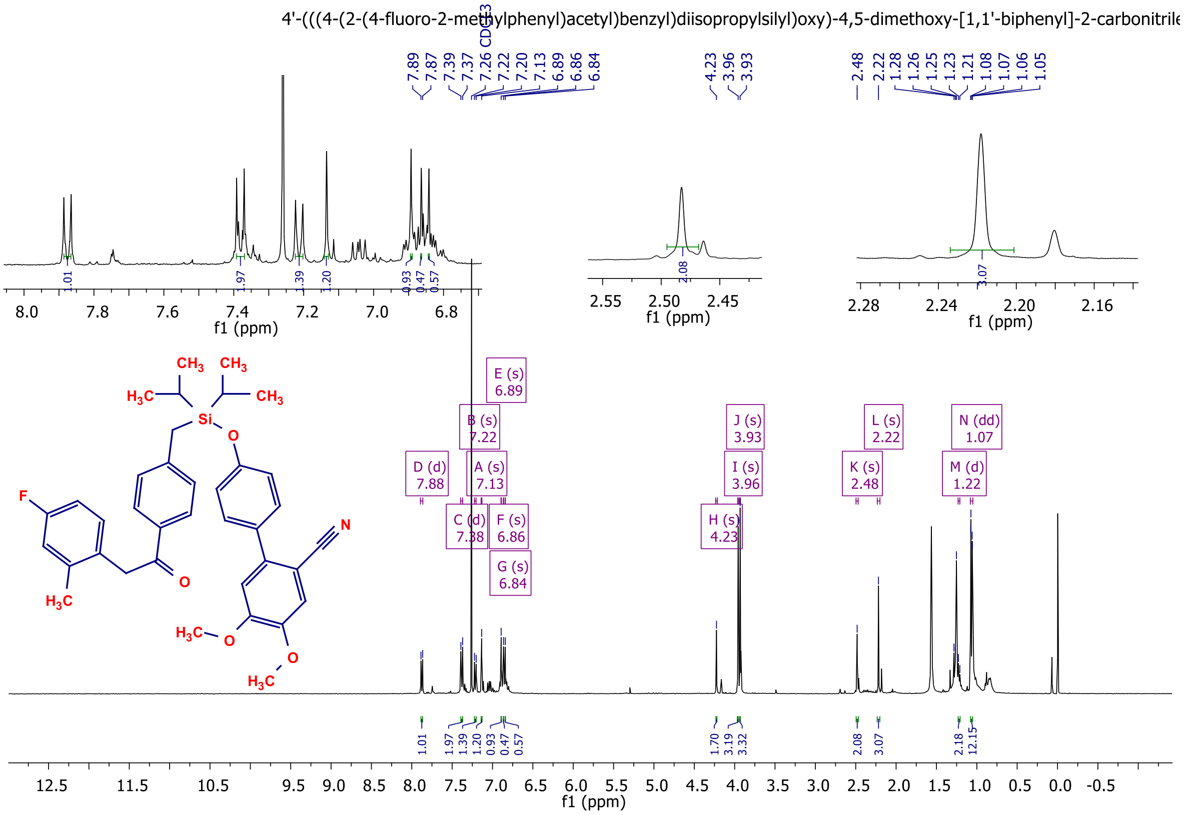

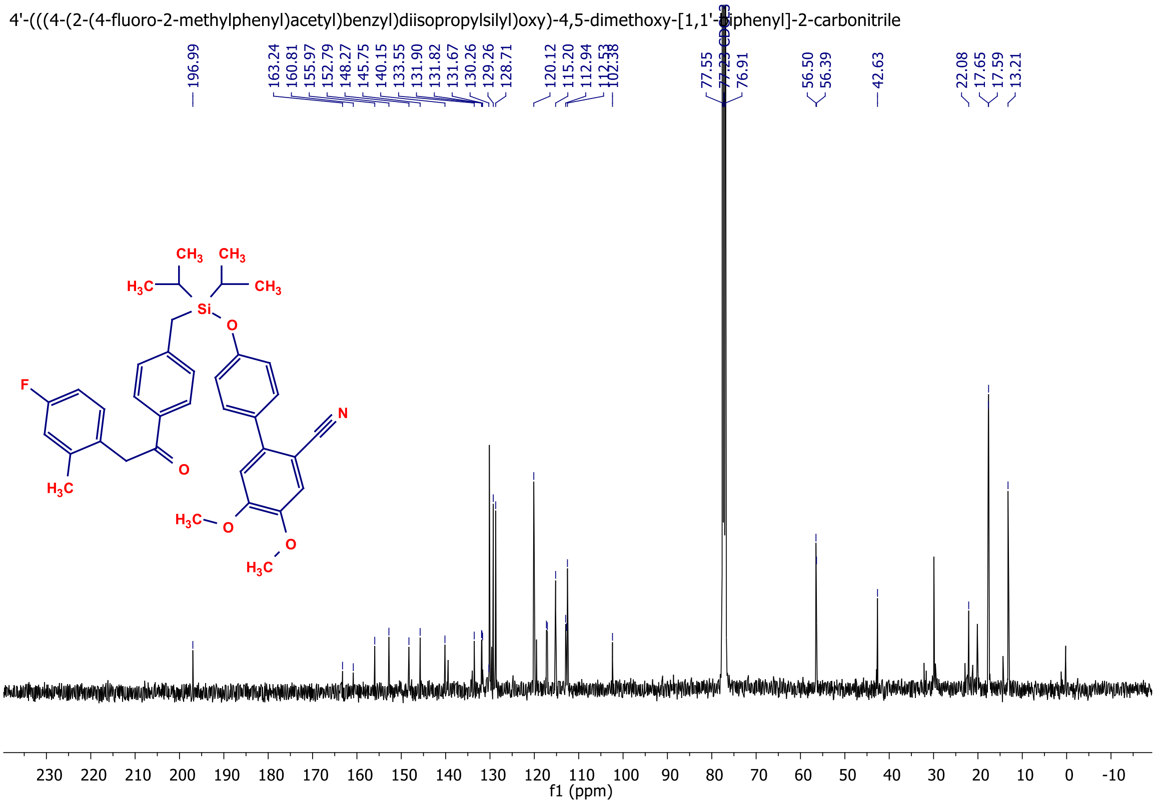

Supplementary Figure 64: ^{1}H and ^{13}C NMR (500 MHz, $CDCl_3$) of 4'-(((4-(2-(3,4-dichlorophenyl)acetyl)benzyl)diisopropylsilyl)oxy)-4,5-dimethoxy-[1,1'-biphenyl]-2-carbonitrile (1s):

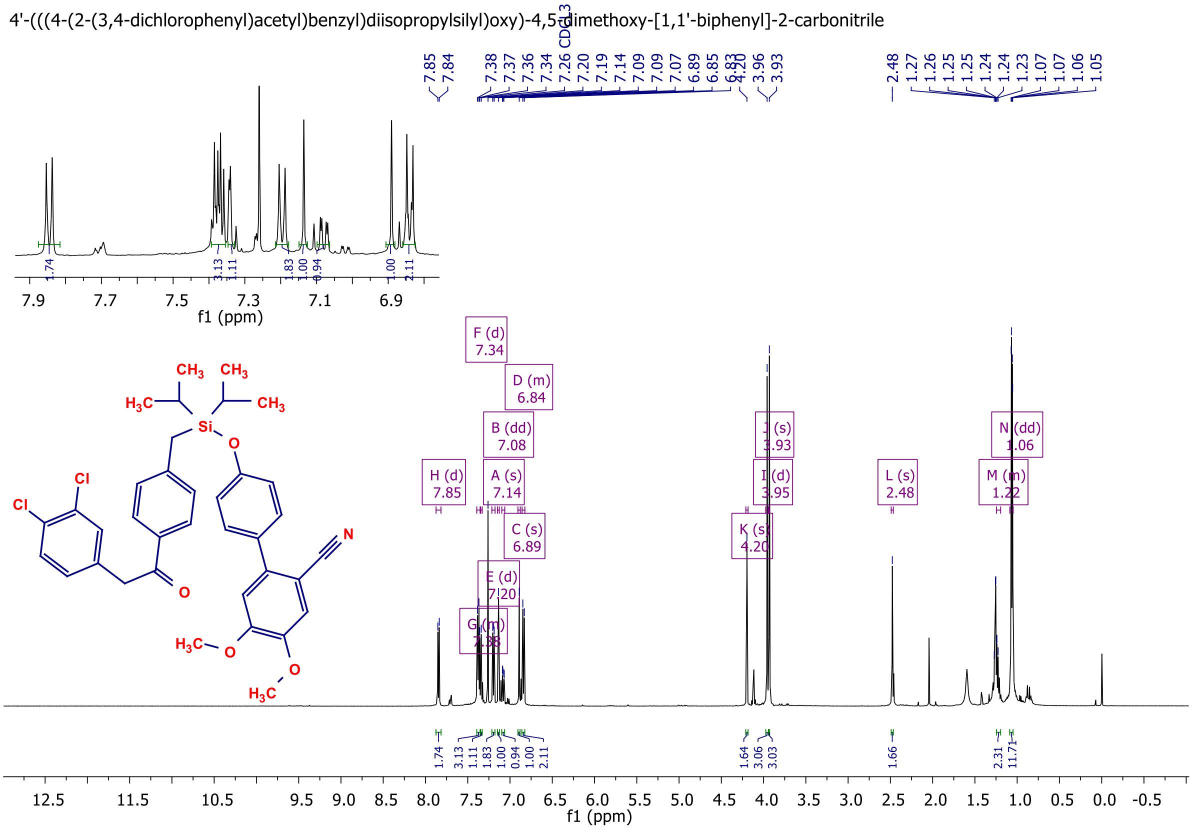

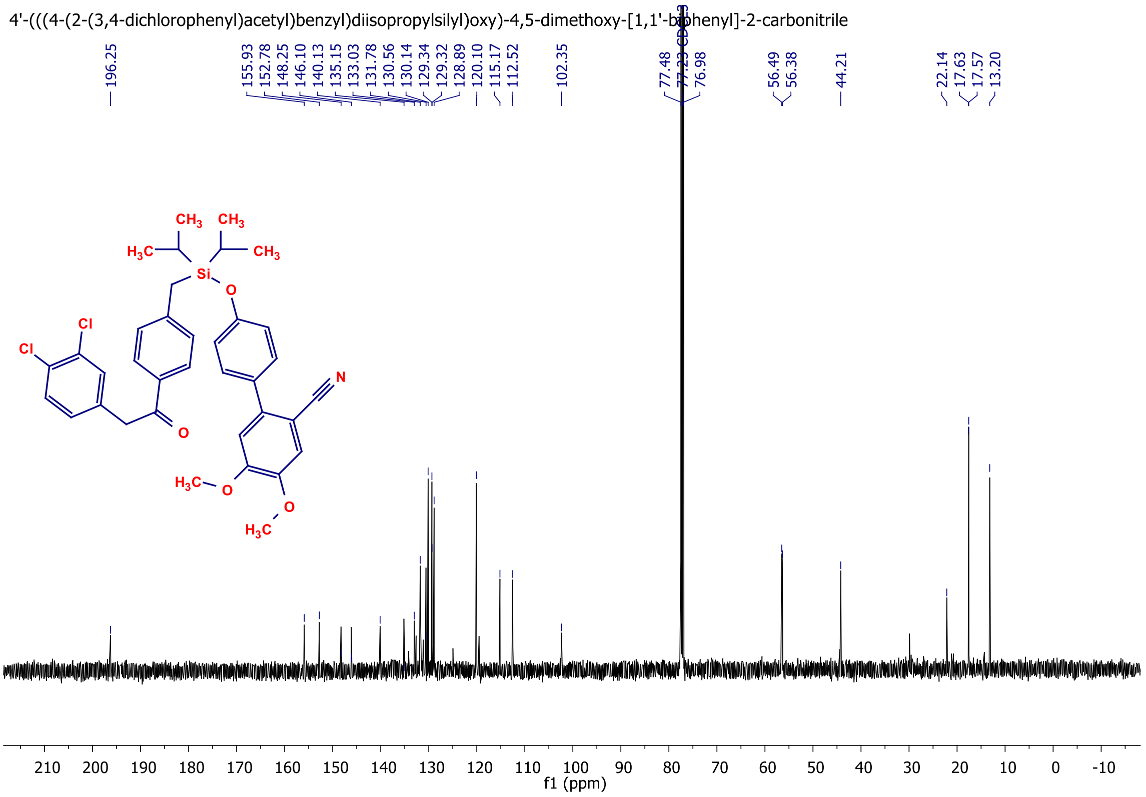

Supplementary Figure 65: ^{1}H and ^{13}C NMR (500 MHz, $CDCl_3$) of 4'-(((4-(2-(2-chlorophenyl)acetyl)benzyl)diisopropylsilyl)oxy)-4,5-dimethoxy-[1,1'-biphenyl]-2-carbonitrile (1r):

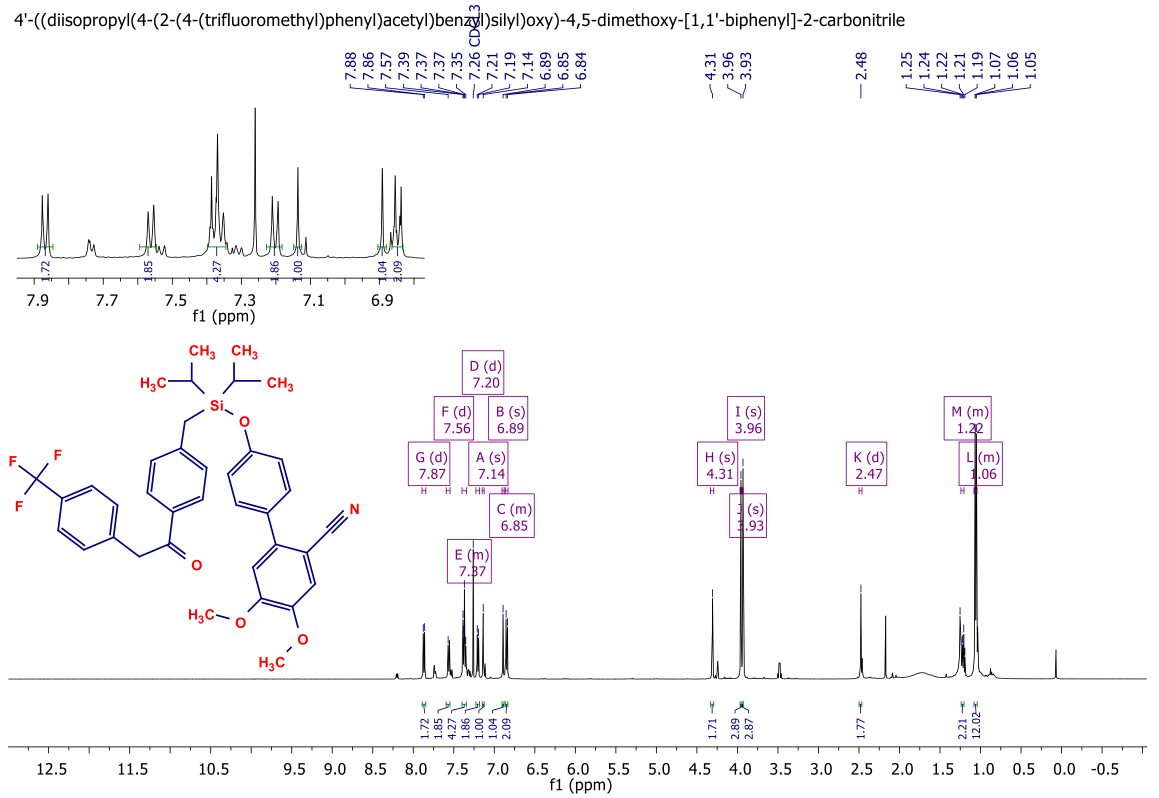

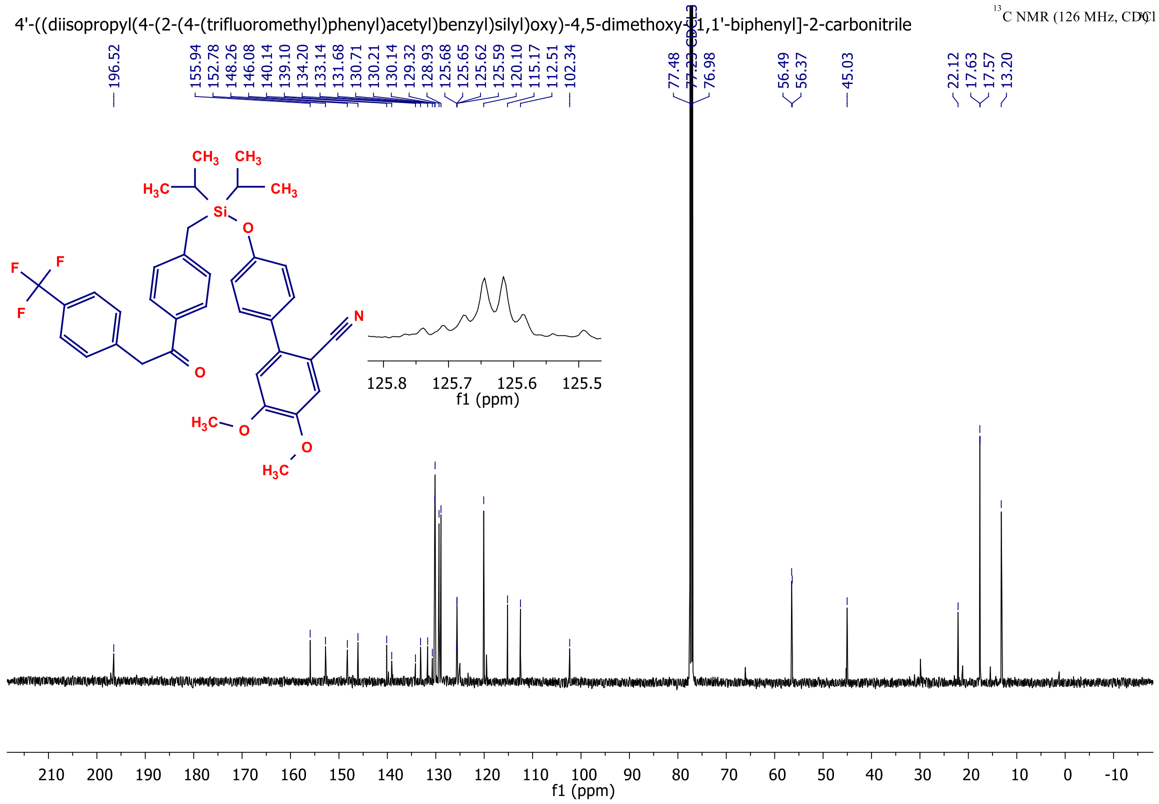

Supplementary Figure 66: ^{1}H and ^{13}C NMR (500 MHz, $CDCl_3$) of 4'-(((4-(2-(2-chlorophenyl)acetyl)benzyl)diisopropylsilyl)oxy)-4,5-dimethoxy-[1,1'-biphenyl]-2-carbonitrile (1r):

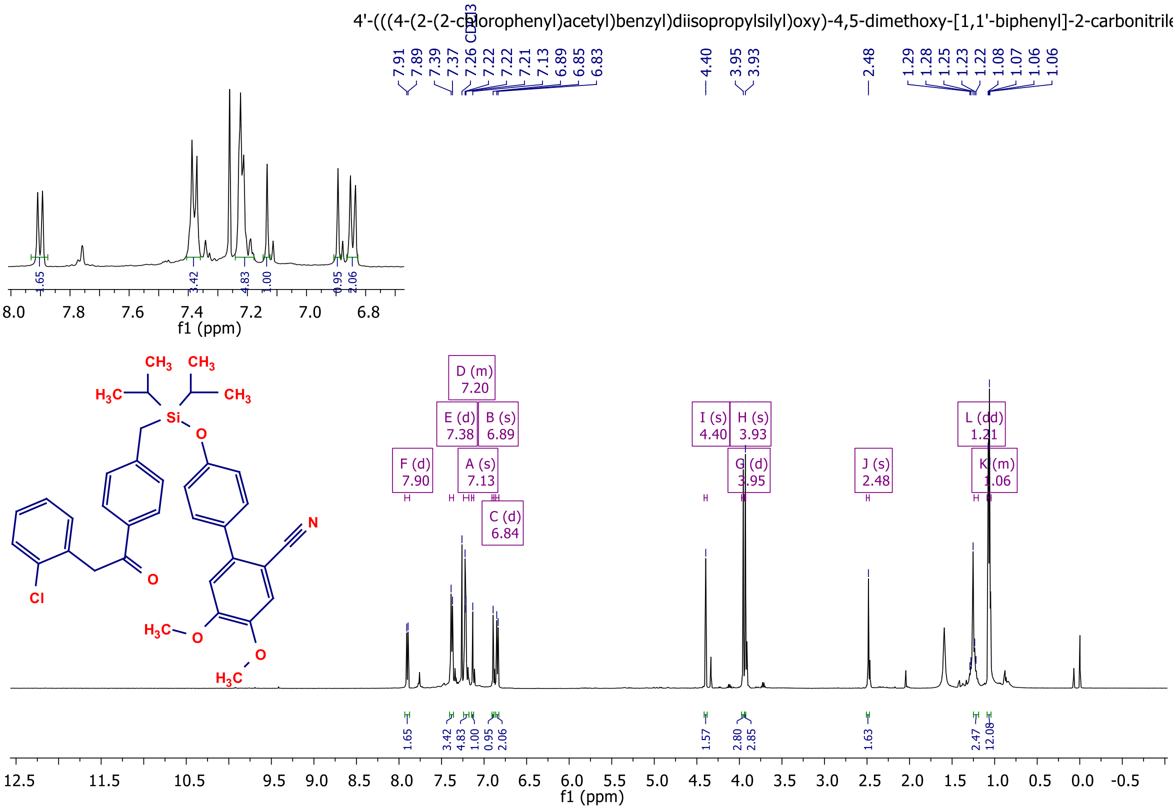

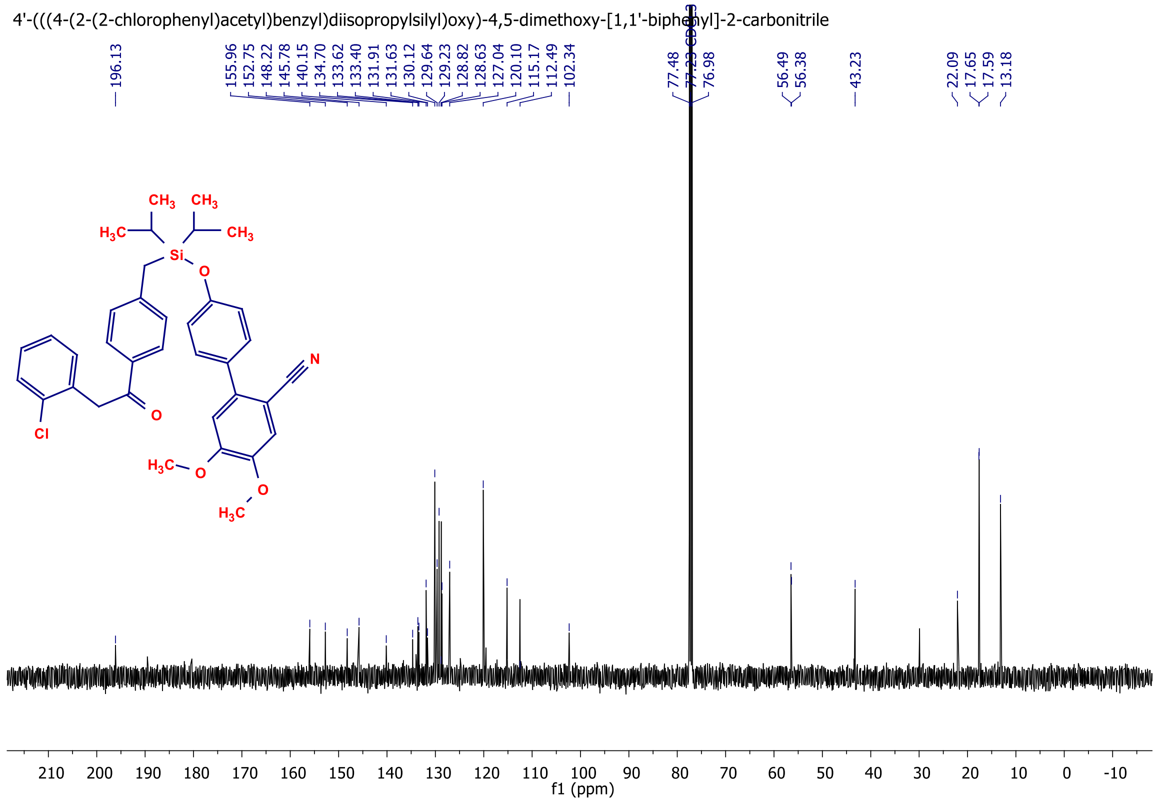

Supplementary Figure 67: ^{1}H and ^{13}C NMR (500 MHz, $CDCl_3$) of 4'-(((2-fluoro-4-propionylbenzyl)diisopropylsilyl)oxy)-4,5-dimethoxy-[1,1'-biphenyl]-2-carbonitrile (1v):

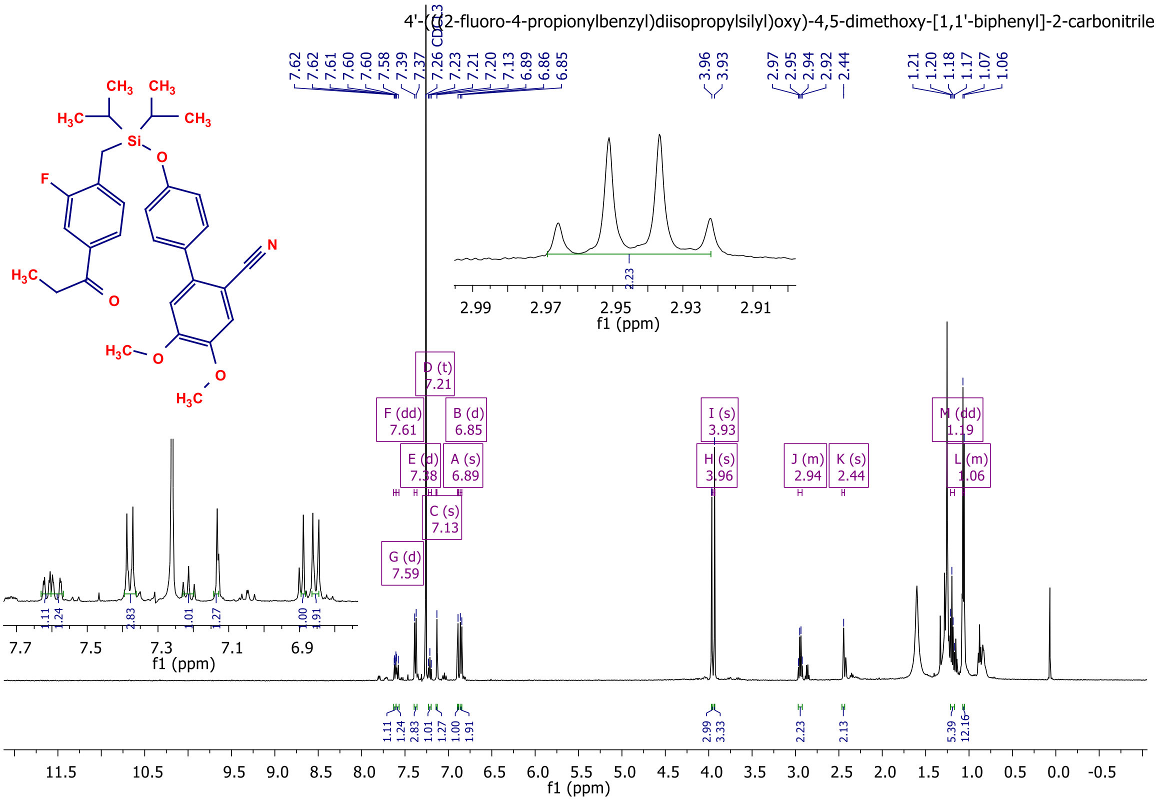

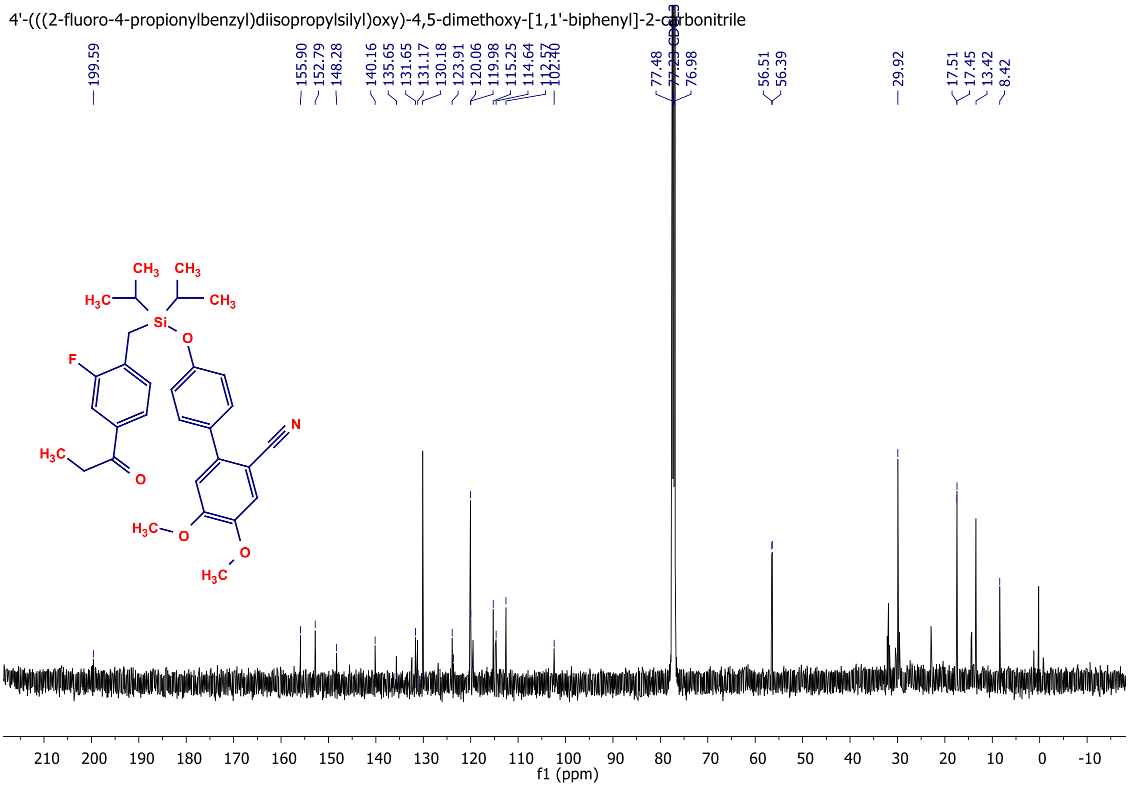

Supplementary Figure 68: ^{1}H and ^{13}C NMR (500 MHz, $CDCl_3$) of -(((4-(2-(2,4-dimethylphenyl)acetyl)benzyl)diisopropylsilyl)oxy)-4,5-dimethoxy-[1,1'-biphenyl]-2-carbonitrile (1w):

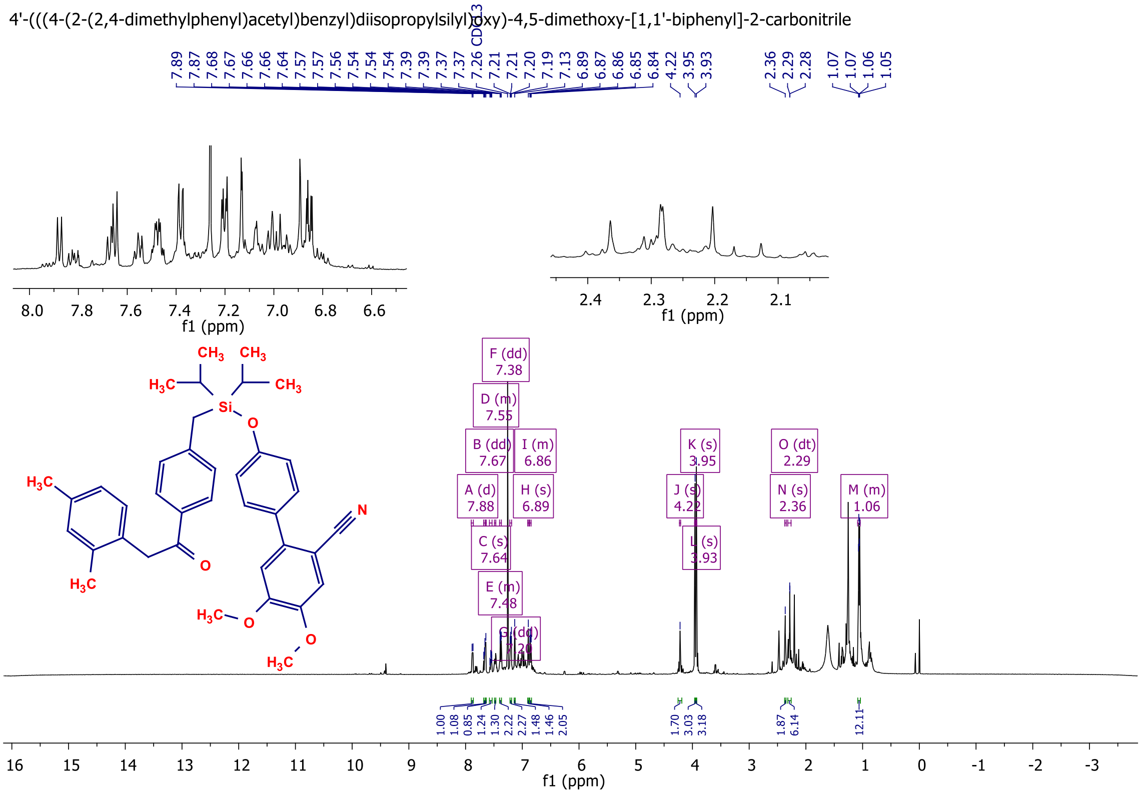

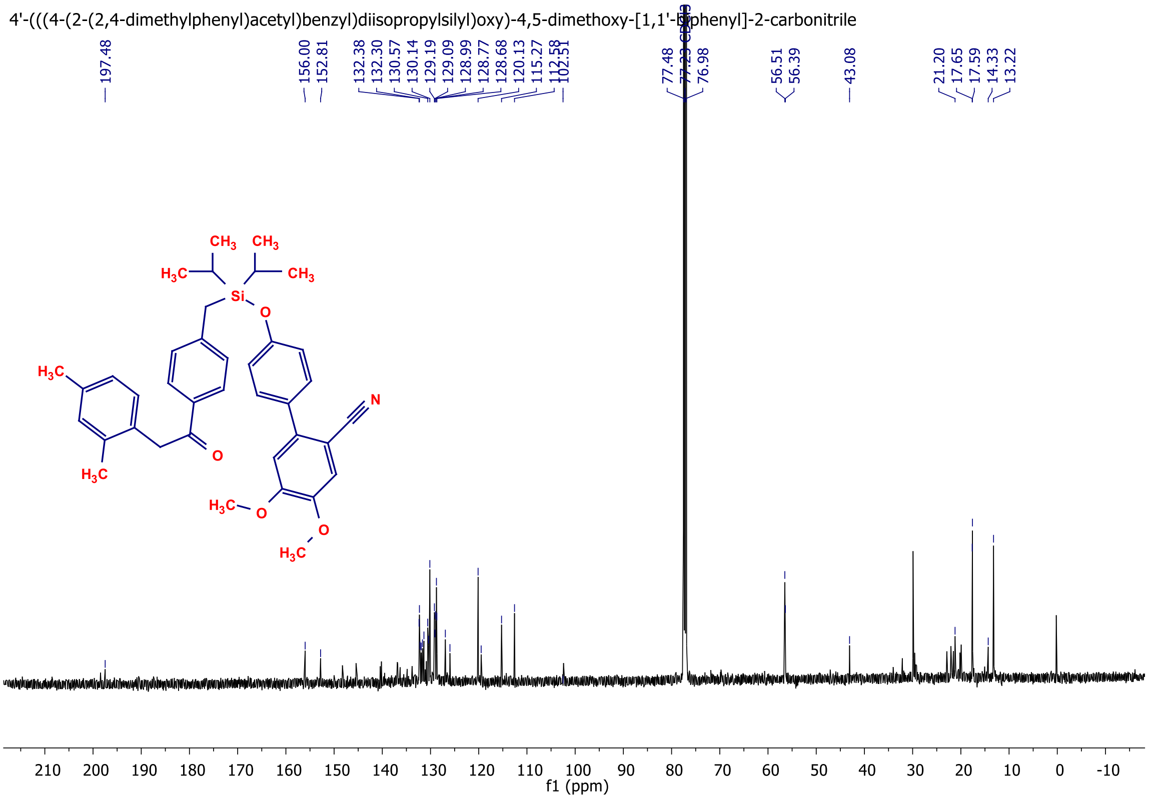

Supplementary Figure 69: ^{1}H and ^{13}C NMR (500 MHz, $CDCl_3$) of 4'-(((4-(2-(4-chlorophenyl)acetyl)benzyl)diisopropylsilyl)oxy)-4,5-dimethoxy-[1,1'-biphenyl]-2-carbonitrile (1x):

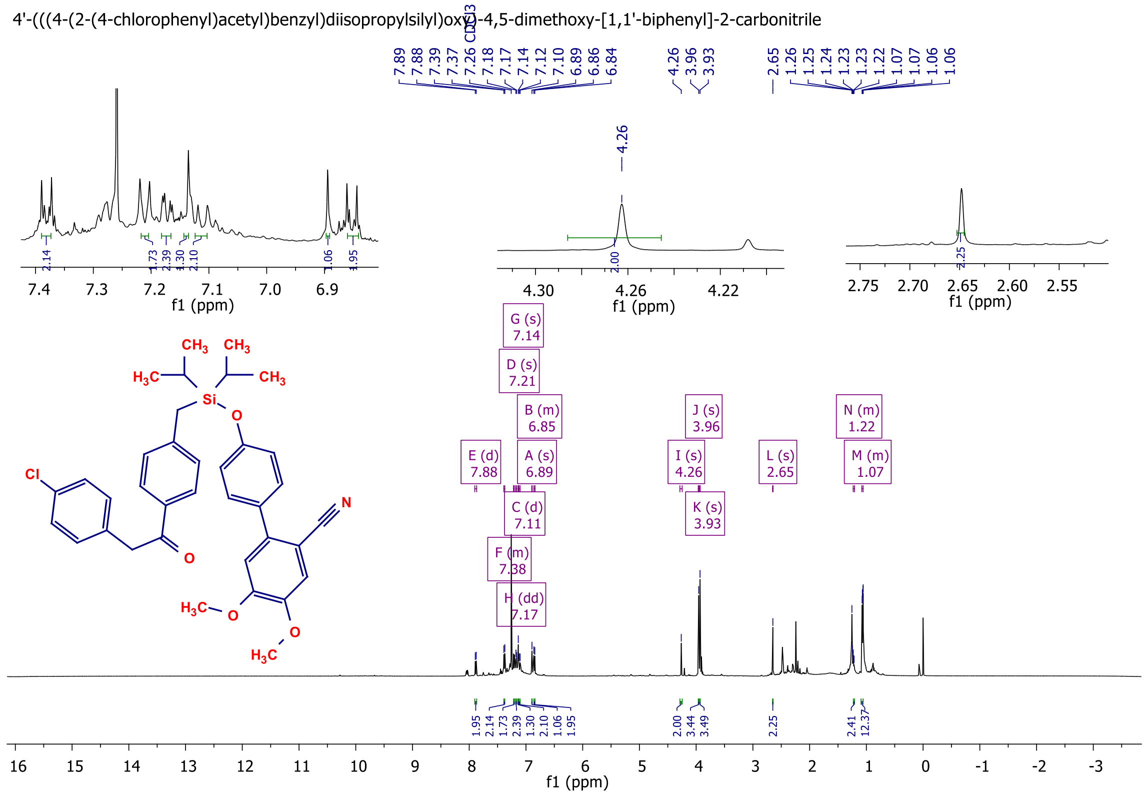

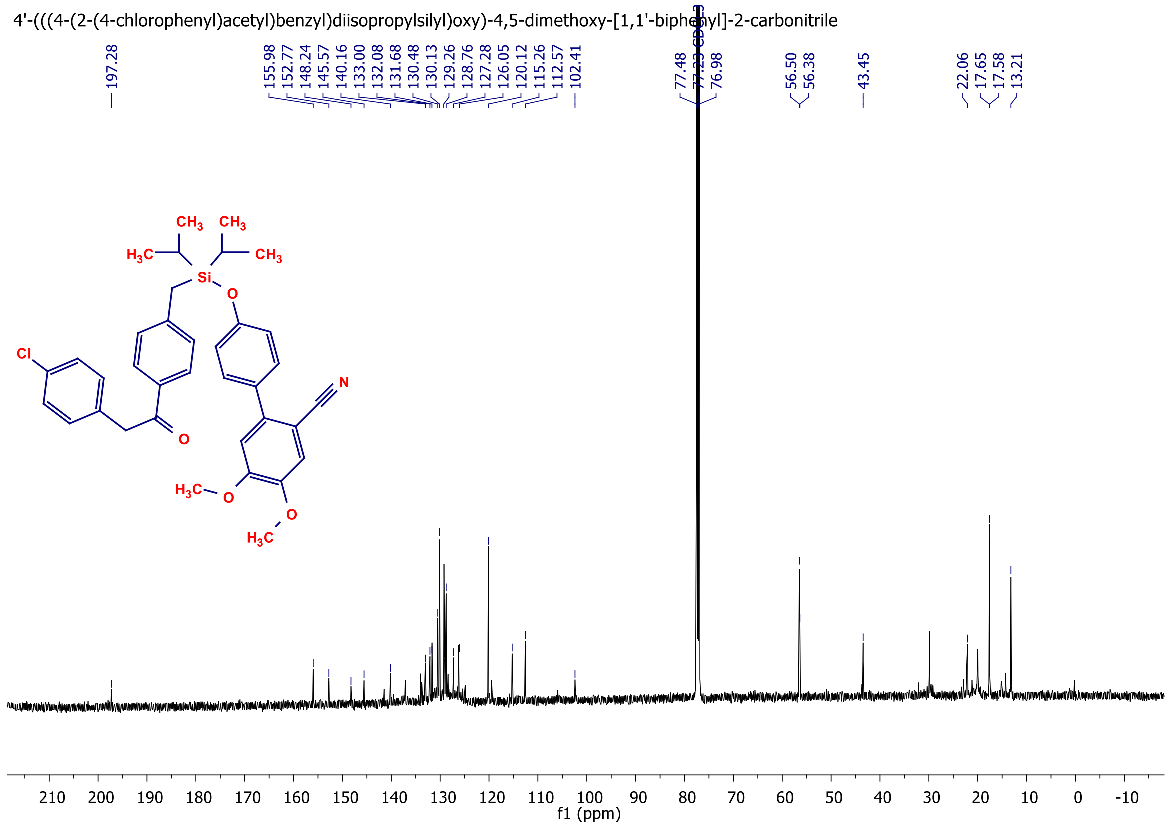

Supplementary Figure 70: ^{1}H (500 MHz, $CDCl_3$) of 4'-((diisopropyl(4-(2-(o-tolyl)acetyl)benzyl)silyl)oxy)-4,5-dimethoxy-[1,1'-biphenyl]-2-carbonitrile (1y):

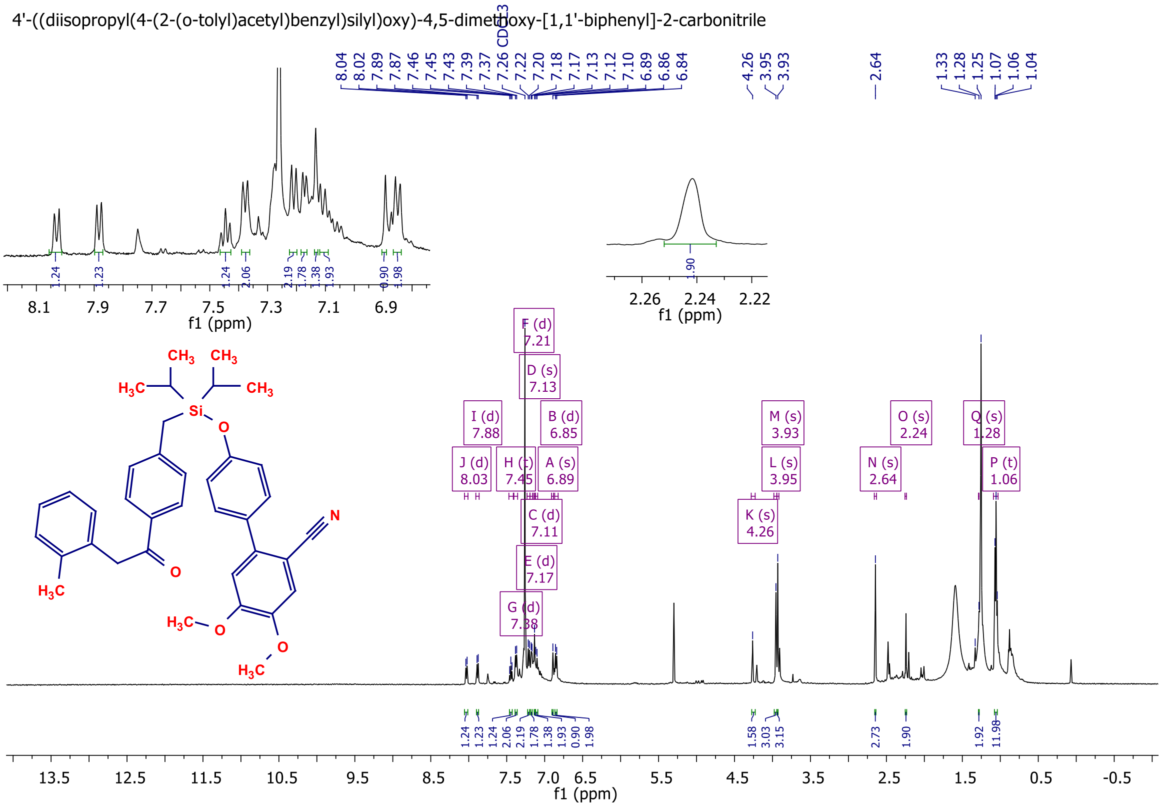

Supplementary Figure 71: ^{1}H and ^{13}C NMR (500 MHz, $CDCl_3$) of 4'-(((4-(2-(2,4-dichlorophenyl)propanoyl)benzyl)diisopropylsilyl)oxy)-4,5-dimethoxy-[1,1'-biphenyl]-2-carbonitrile (1z):

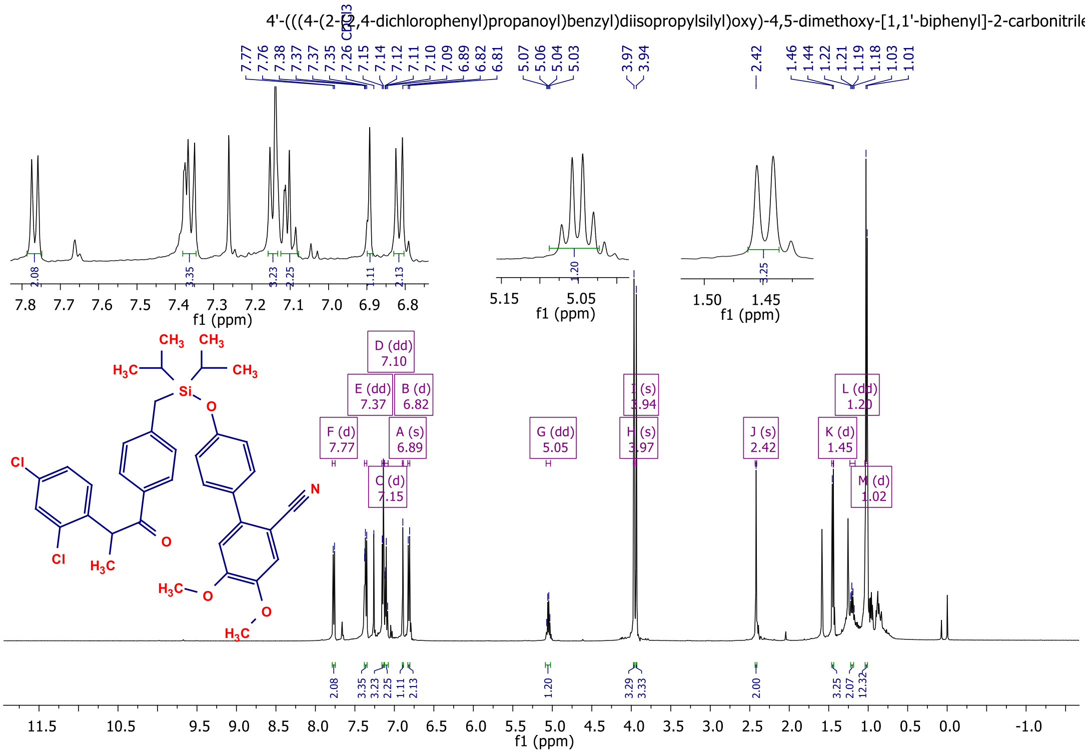

4'-(((4-(2-(2,4-dichlorophenyl)propanoyl)benzyl)diisopropylsilyl)oxy)-4,5-dimethoxy-[1,1'-biphenyl]-2-carbonitrile

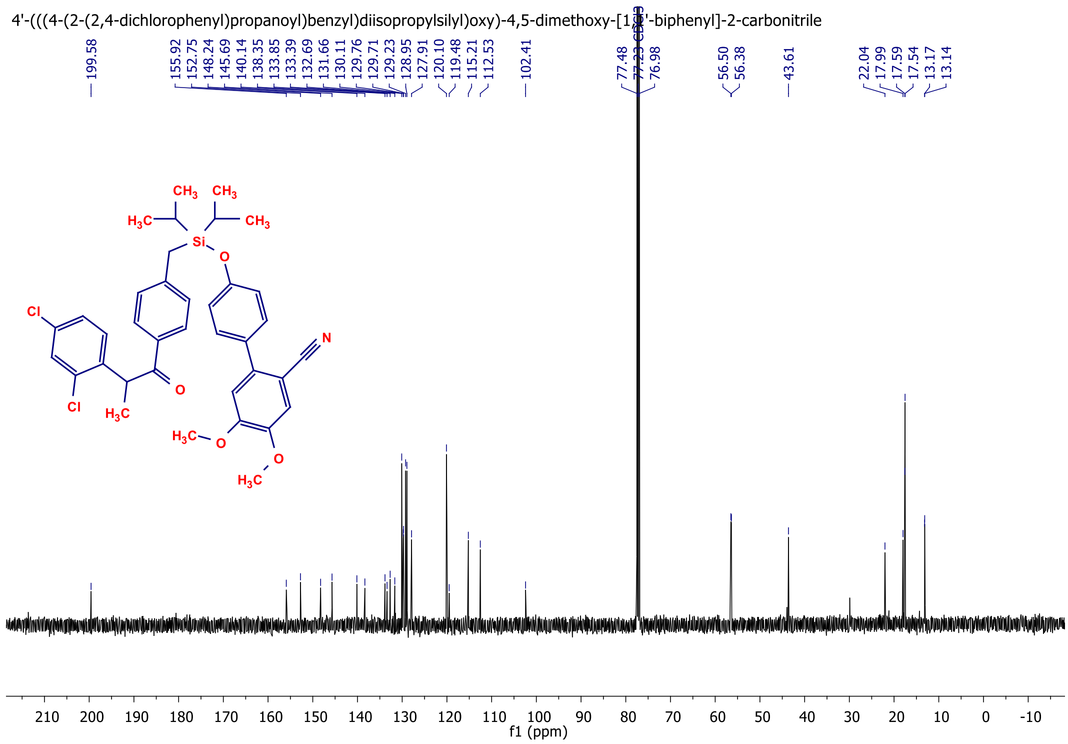

Applications:

Supplementary Figure 72: ^{1}H and ^{13}C NMR (500 MHz, $CDCl_3$) of 1-(4-benzylphenyl)ethan-1-one (5a):

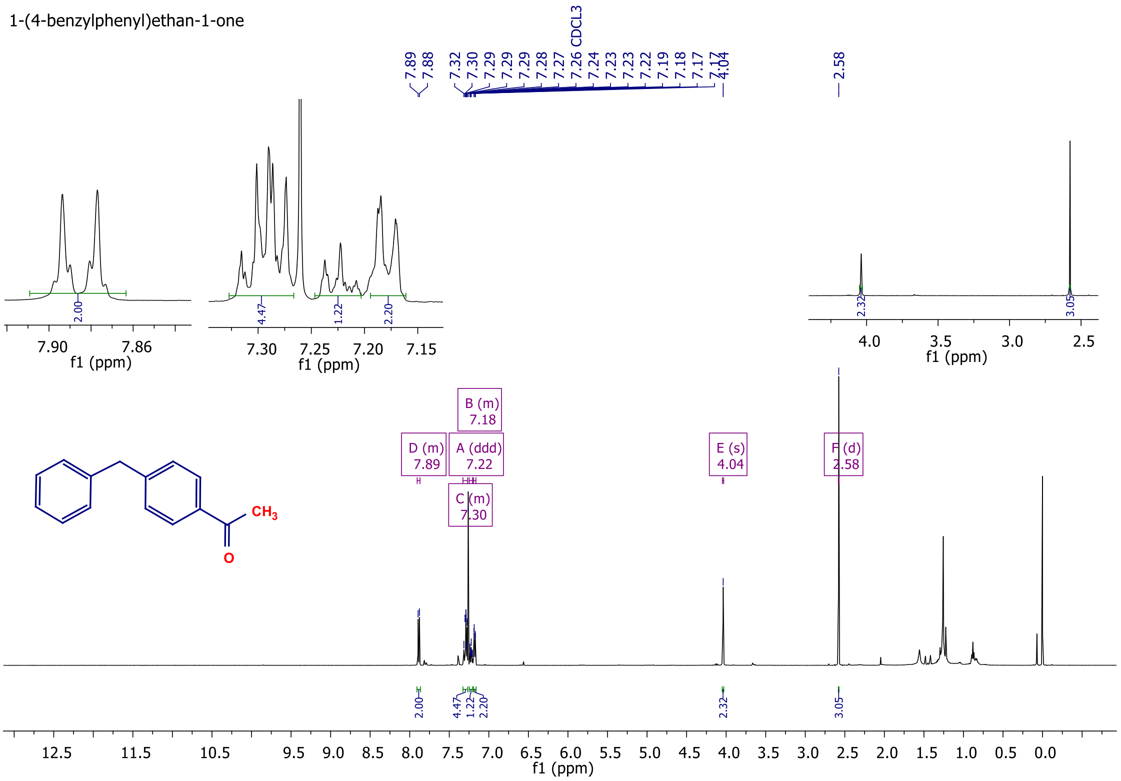

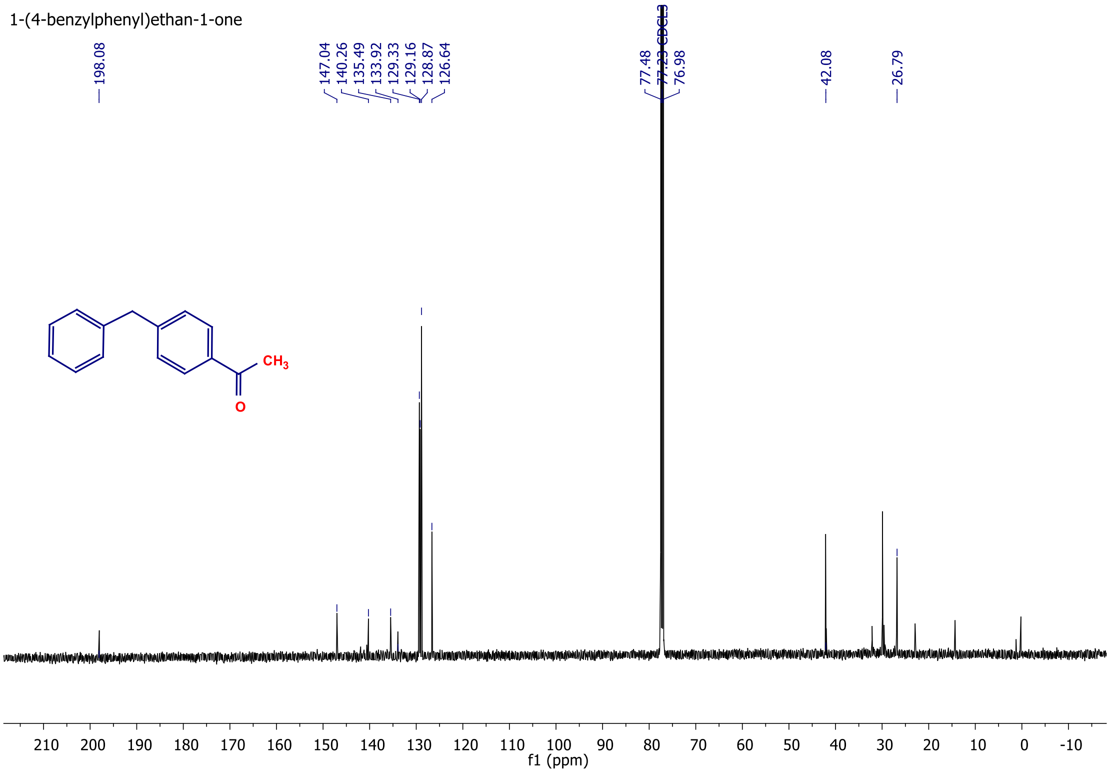

Supplementary Figure 73: ^{1}H and ^{13}C NMR (500 MHz, $CDCl_3$) of 2,2-dibromo-1-(p-tolyl)propan-1-one (5c):

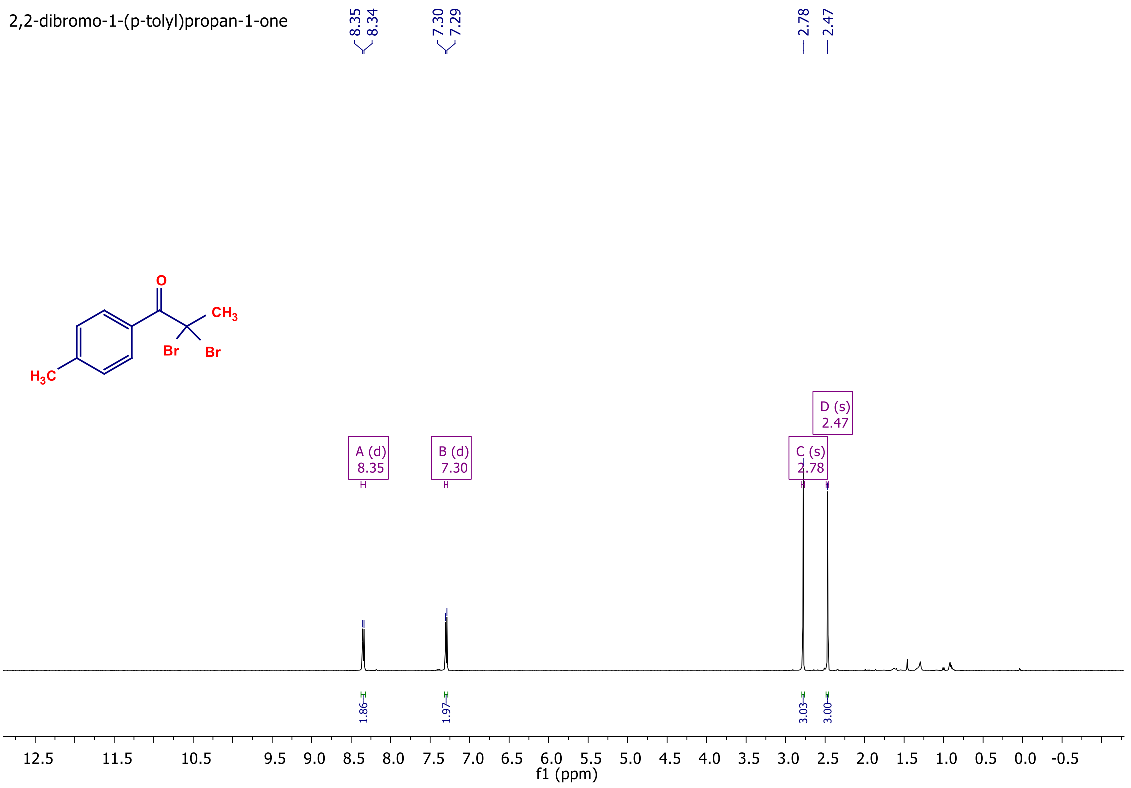

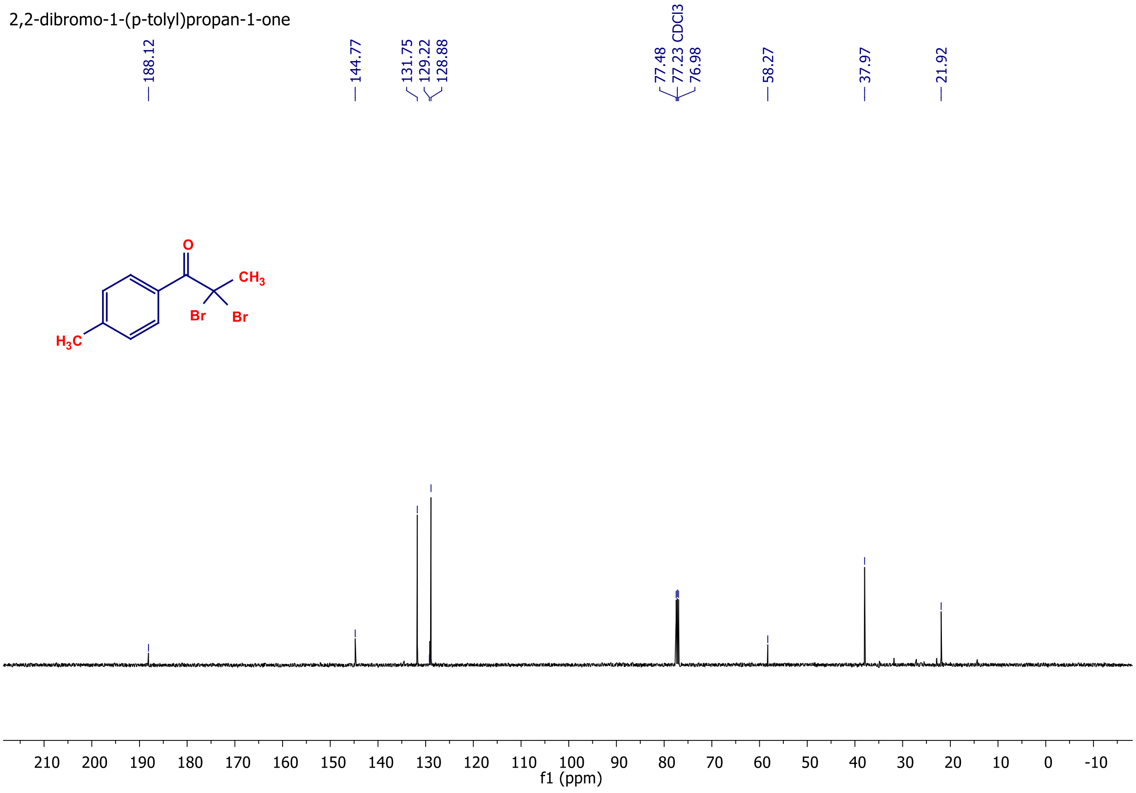

Supplementary Figure 74: ^{1}H and ^{13}C NMR (400 MHz, $CDCl_3$) of 2-hydroxy-1-(p-tolyl)propan-1-one (5e):

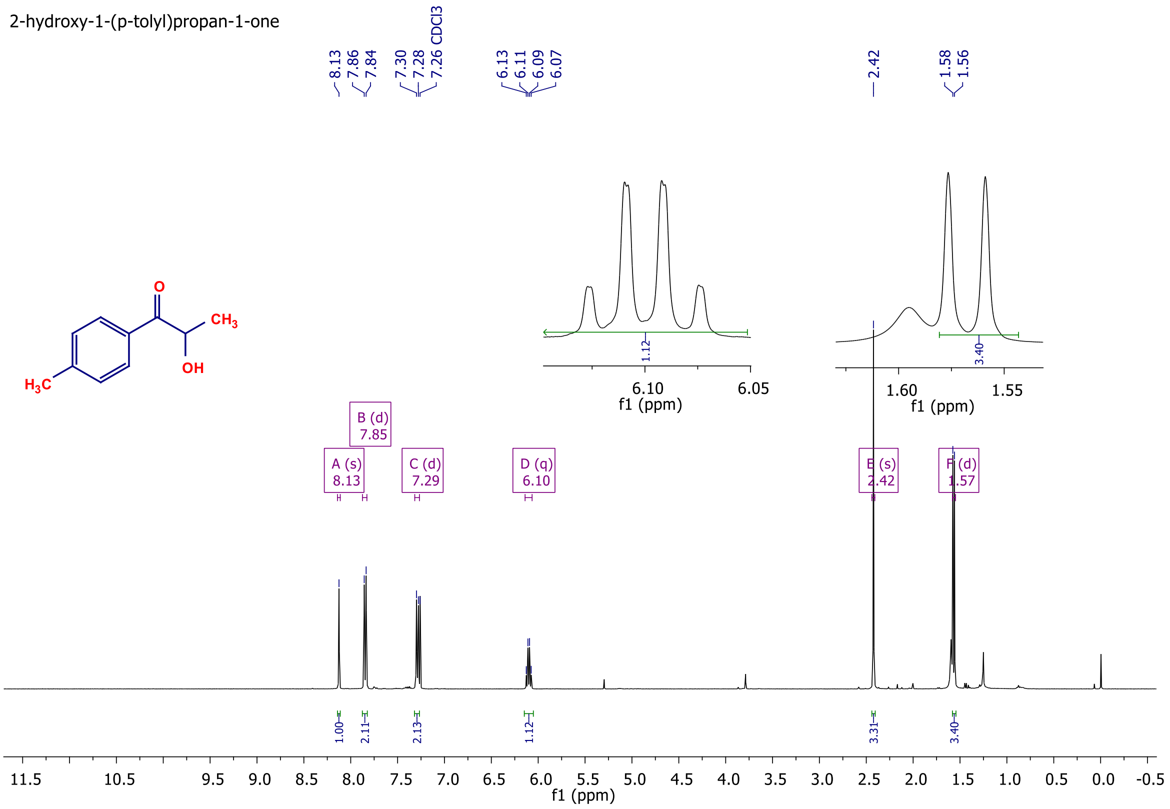

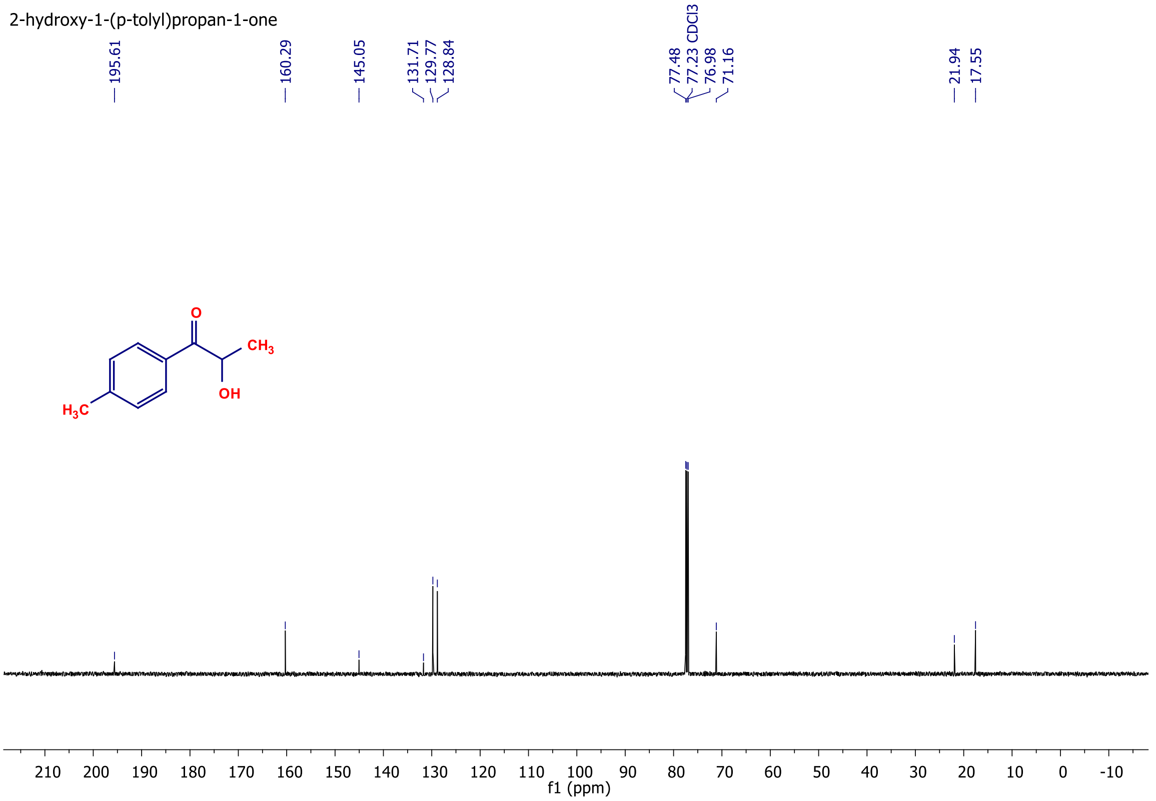

Supplementary Figure 75: ^{1}H and ^{13}C NMR (400 MHz, $CDCl_3$) of 2-bromo-1-(p-tolyl)propan-1-one (5f):

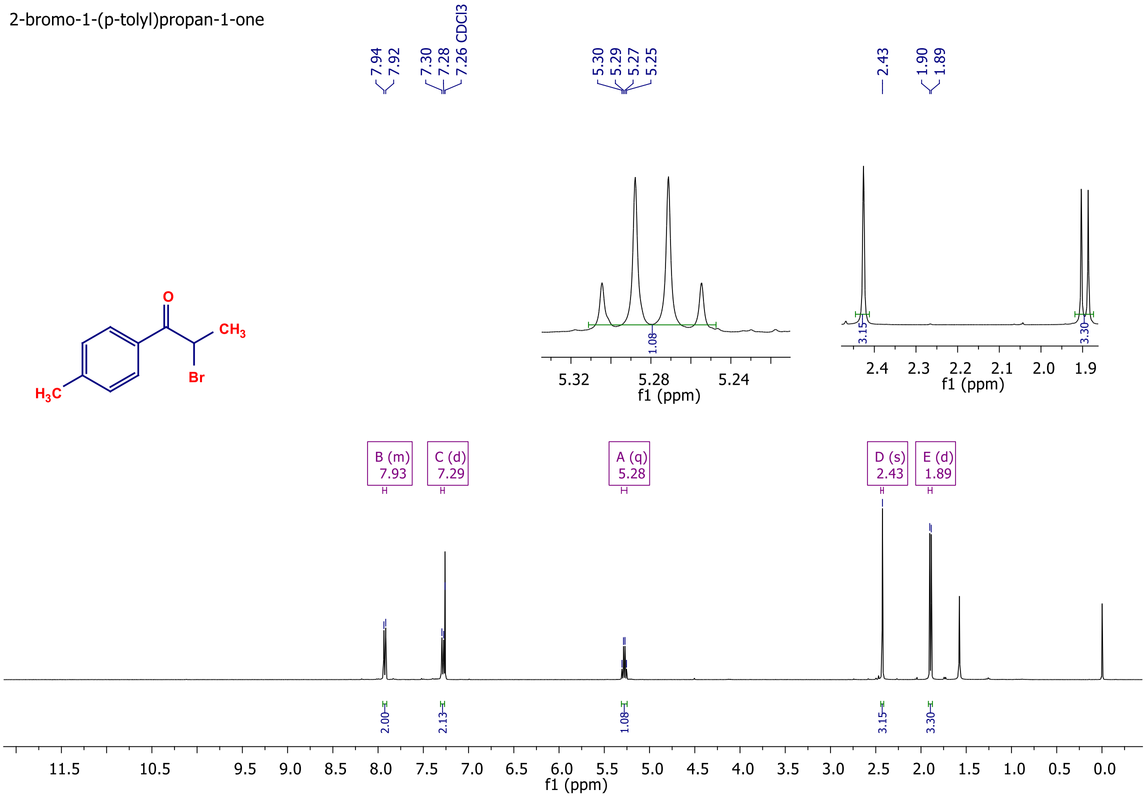

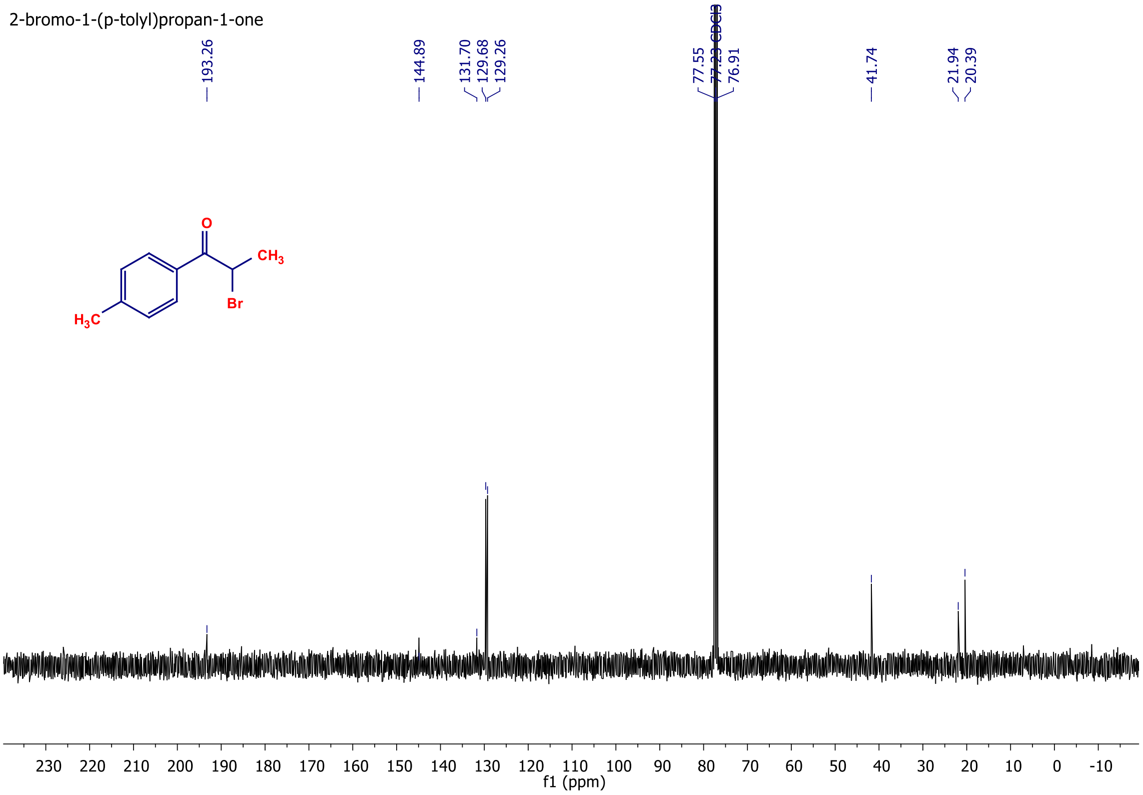

Supplementary Figure 76: ^{1}H and ^{13}C NMR (500 MHz, $CDCl_3$) of 3,5-dimethyl-2,4-di-p-tolylpyridine (5g:)

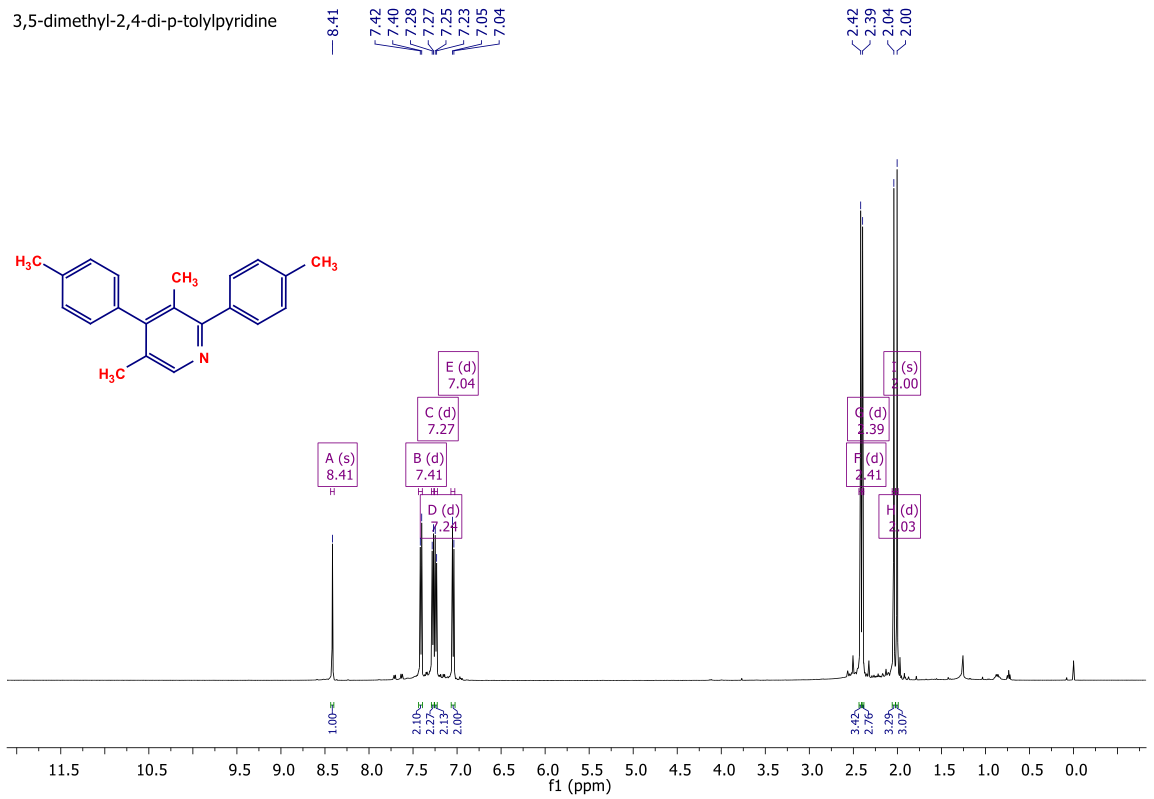

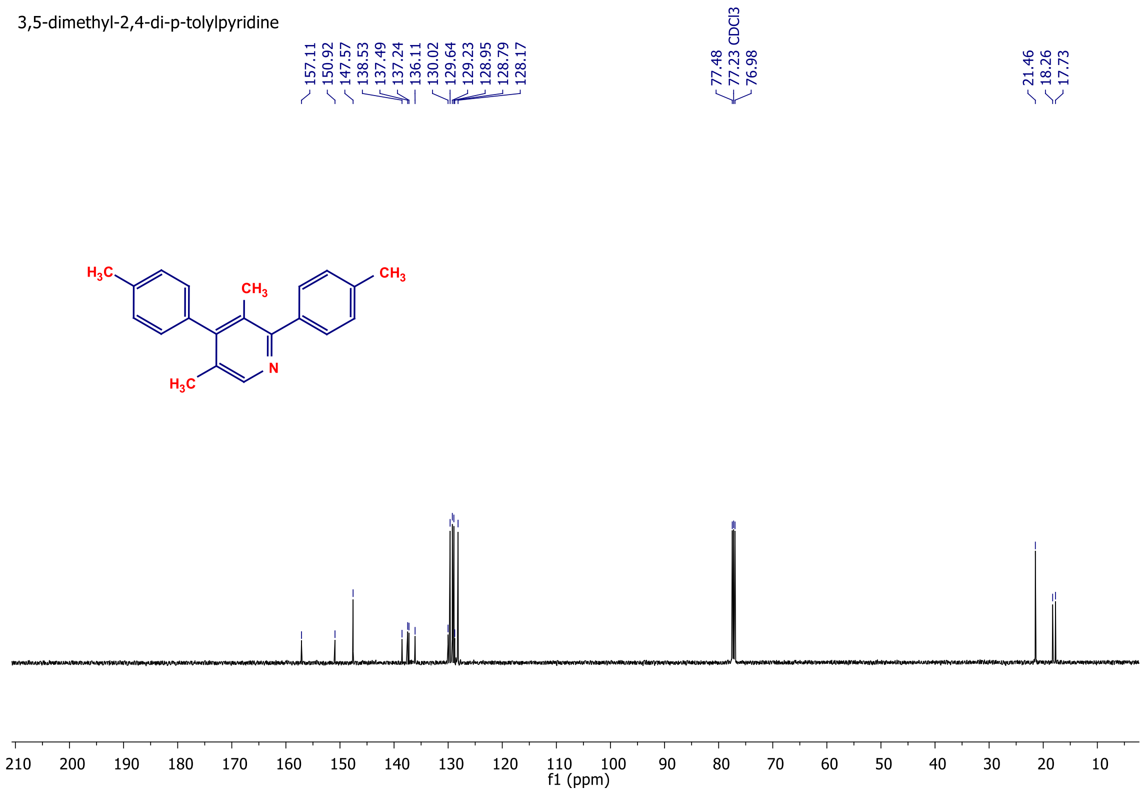

Supplementary References:

1 Maji, A. *et al.* Experimental and Computational Exploration of para-Selective Silylation with a Hydrogen-Bonded Template. *Angew. Chem. Int. Ed.* **56**, 14903-14907, doi:10.1002/anie.201708449 (2017).

2 Gaussian 09, R. D. *et al. Gaussian, Inc., Wallingford CT* (2009).

3 Becke, A. D. Density-functional exchange-energy approximation with correct asymptotic behavior. *Phys. Rev. A* **38**, 3098−3100, doi:https://doi.org/10.1103/PhysRevA.38.3098 (1988).

4 Lee, C., Yang, W. & Parr, R. G. Development of the Colle-Salvetti correlation-energy formula into a functional of the electron density. *Phys. Rev. B* **37**, 785−789, doi: https://doi.org/10.1103/PhysRevB.37.785 (1988).

5 Zhao, Y. & Truhlar, D. G. The M06 suite of density functionals for main group thermochemistry, thermochemical kinetics, noncovalent interactions, excited states, and transition elements: two new functionals and systematic testing of four M06-class functionals and 12 other functionals. *Theor. Chem. Acc.* **120**, 215–241, doi; https://doi.org/10.1007/s00214-007-0310-x (2008).

6 Marenich, A. V., Cramer, C. J. & Truhlar, D. G. Universal Solvation Model Based on Solute Electron Density and on a Continuum Model of the Solvent Defined by the Bulk Dielectric Constant and Atomic Surface Tensions. *J. Phys. Chem. B,* **113**, 6378–6396, doi: 10.1021/jp810292n (2009).

7 Legault, C. Y. *CYLview, 1.0b, Université de Sherbrooke*. (2009).

8 Cheng, G. *et al.* Role of *N*-Acyl Amino Acid Ligands in Pd(II)-Catalyzed Remote C–H Activation of Tethered Arenes. *J. Am. Chem. Soc.* **136**, 894−897, doi: 10.1021/ja411683n (2014).
