# Supplementary material for: H-bonded reusable template assisted para-selective ketonisation using soft electrophilic vinyl ethers
Source: Nat Commun. 2018 Sep 4;9:3582. doi: 10.1038/s41467-018-06018-2 (PMC6123475; doi:10.1038/s41467-018-06018-2)
Supplement: Supplementary file 3 — Supplementary Data 2 [file 41467_2018_6018_MOESM3_ESM.pdf]

Supplementary Data

H-Bonded Reusable Template-Assisted *Para*-Selective Ketonisation Using Soft Electrophilic Vinyl Ethers

Maji et al.

Cartesian Coordinates:

```
 5
B3LYP electronic energy:         -2295.85867899 a.u.
B3LYP enthalpy:                  -2295.158751 a.u.
B3LYP free energy:               -2295.276747 a.u.
M06 SCF energy in solution:      -2295.13848516 a.u.
M06 enthalpy in solution:        -2294.438557 a.u.
M06 free energy in solution:     -2294.556553 a.u.

Cartesian coordinates
ATOM       X           Y           Z
Pd       2.203135   -0.794580   -0.295822
C        0.408802   -1.590722   -0.562273
C       -0.017252   -2.601328    0.306187
C       -1.288872   -3.164366    0.156208
C       -2.157723   -2.743099   -0.861957
C       -1.695160   -1.763102   -1.751192
C       -0.432865   -1.183872   -1.602306
H        0.636974   -2.960373    1.096092
H       -1.604134   -3.948896    0.842383
H       -2.340525   -1.428146   -2.560905
C       -3.565706   -3.283199   -0.973710
H       -3.592947   -4.349226   -0.708373
H       -3.911524   -3.220970   -2.014132
Si      -4.867180   -2.399808    0.109635
C       -6.625109   -2.873460   -0.462235
H       -7.297379   -2.622174    0.373029
C       -4.577446   -2.735219    1.967751
H       -3.510313   -2.532098    2.144833
C       -7.083262   -2.059679   -1.688674
H       -6.443357   -2.254381   -2.559330
H       -8.107919   -2.334998   -1.972957
H       -7.059356   -0.983655   -1.495564
C       -6.770673   -4.384529   -0.743634
H       -6.506106   -5.006130    0.118517
H       -7.806565   -4.626602   -1.015761
H       -6.136746   -4.696877   -1.582777
C       -4.825614   -4.204120    2.369353
H       -4.246741   -4.910828    1.762449
H       -4.545427   -4.368778    3.418142
H       -5.884565   -4.472996    2.274211
C       -5.396034   -1.784851    2.864619
H       -5.178692   -1.967466    3.925576
H       -5.180013   -0.731694    2.656094
H       -6.474498   -1.934306    2.725524
O       -4.822143   -0.724414   -0.175088
C       -3.915525    0.279772   -0.059632
C       -2.868364    0.276944    0.871652
C       -4.068764    1.387550   -0.907589
C       -1.967636    1.336718    0.914393
H       -2.739937   -0.561231    1.545731
C       -3.169109    2.445868   -0.851605
```

H	-4.887694	1.388379	-1.620095
C	-2.087137	2.434386	0.046793
H	-1.166046	1.311720	1.646741
H	-3.283828	3.278521	-1.540801
C	-1.113707	3.551833	0.071637
C	-1.569097	4.881643	0.103365
C	0.282816	3.348992	0.053865
C	-0.695468	5.966456	0.131220
H	-2.638333	5.050767	0.135578
C	1.183059	4.442270	0.091909
C	0.712252	5.743562	0.133345
H	2.246789	4.240613	0.073524
C	0.845255	2.048152	-0.075060
N	1.358064	1.013696	-0.200392
O	1.482506	6.858199	0.173089
O	-1.072198	7.262276	0.172905
C	-2.461470	7.568643	0.173301
H	-2.961444	7.153836	1.057372
H	-2.521962	8.657379	0.198158
H	-2.952788	7.194891	-0.733679
C	2.894129	6.693702	0.178493
H	3.309369	7.701557	0.219617
H	3.227742	6.125053	1.056233
H	3.242700	6.192534	-0.733845
H	-0.119122	-0.409542	-2.296482
C	5.689153	-2.807309	-0.060085
C	4.434895	-1.957921	-0.128407
O	3.312596	-2.558363	-0.282451
O	4.490089	-0.700055	-0.038805
H	5.757448	-3.404461	-0.978864
H	5.582312	-3.524787	0.763961
N	6.847823	-1.964257	0.115426
H	6.697018	-0.965195	0.157475
C	8.097286	-2.485577	0.198561
O	8.370356	-3.673513	0.148368
O	9.016738	-1.488971	0.348543
C	10.369320	-1.942853	0.446044
H	10.972513	-1.040261	0.558260
H	10.501327	-2.598069	1.312118
H	10.665466	-2.488186	-0.454882

vinyl-methyl-ether

B3LYP SCF energy: -193.10696528 a.u.
B3LYP enthalpy: -193.015982 a.u.
B3LYP free energy: -193.050191 a.u.
M06 SCF energy in solution: -193.03568264 a.u.
M06 enthalpy in solution: -192.944699 a.u.
M06 free energy in solution: -192.978908 a.u.
Three lowest frequencies (cm-1): 46.5019 168.0803 316.0482

Cartesian coordinates

ATOM	X	Y	Z
C	-0.614684	0.352909	-0.044745

H	-0.364455	1.412863	-0.116138
C	-1.874592	-0.073417	0.048697
H	-2.689710	0.640448	0.042100
H	-2.111161	-1.129890	0.120906
O	0.447061	-0.499897	-0.085205
C	1.721656	0.107700	0.056756
H	2.462099	-0.681426	-0.089360
H	1.880075	0.891623	-0.697504
H	1.852379	0.542398	1.057388

6
B3LYP SCF energy: -2488.98612970 a.u.
B3LYP enthalpy: -2488.192560 a.u.
B3LYP free energy: -2488.336316 a.u.
M06 SCF energy in solution: -2488.20621525 a.u.
M06 enthalpy in solution: -2487.412646 a.u.
M06 free energy in solution: -2487.556402 a.u.
Three lowest frequencies (cm-1): 9.4566 18.0622 21.3487

Cartesian coordinates

ATOM	X	Y	Z
C	-1.156309	-2.344535	-0.119149
C	-2.131761	-2.689866	0.827425
C	-3.479150	-2.371548	0.621209
C	-3.900544	-1.699015	-0.535282
C	-2.925725	-1.386478	-1.493732
C	-1.579672	-1.700455	-1.290634
H	-1.849457	-3.198268	1.747294
H	-4.211784	-2.654912	1.375584
H	-3.220270	-0.872292	-2.406877
C	-5.344638	-1.297280	-0.740496
H	-6.017146	-2.091725	-0.386582
H	-5.550310	-1.186641	-1.813585
Si	-5.919449	0.317786	0.104220
C	-7.608965	0.844428	-0.611540
H	-8.043319	1.547672	0.116351
C	-5.952191	0.143089	2.006311
H	-4.969244	-0.263905	2.287668
C	-7.473839	1.592909	-1.952569
H	-7.031157	0.952125	-2.726500
H	-8.460282	1.907723	-2.319051
H	-6.843493	2.481443	-1.859744
C	-8.589047	-0.339314	-0.760700
H	-8.753060	-0.878272	0.178688
H	-9.568268	0.014279	-1.109982
H	-8.228353	-1.065809	-1.499587
C	-7.011507	-0.857513	2.512791
H	-6.920113	-1.843898	2.042221
H	-6.915841	-1.003079	3.596946
H	-8.028961	-0.492967	2.327477
C	-6.118910	1.505241	2.709909
H	-6.089861	1.385996	3.801309
H	-5.332420	2.214310	2.430996

H	-7.082819	1.966827	2.460908
O	-4.909520	1.610057	-0.330575
C	-3.597733	1.938960	-0.185442
C	-2.799823	1.492195	0.875939
C	-3.037580	2.799321	-1.141521
C	-1.458718	1.858075	0.944232
H	-3.215087	0.844052	1.638286
C	-1.696285	3.159321	-1.064125
H	-3.664233	3.153893	-1.953977
C	-0.871021	2.677253	-0.032640
H	-0.863137	1.500990	1.778887
H	-1.270077	3.790471	-1.839648
C	0.579267	2.987947	-0.014727
C	1.039160	4.301701	-0.206738
C	1.555551	1.983912	0.149747
C	2.398190	4.613286	-0.231454
H	0.303650	5.089549	-0.314610
C	2.938597	2.280761	0.118428
C	3.370973	3.583550	-0.069250
H	3.645003	1.464953	0.212222
C	1.197194	0.614470	0.283656
N	0.973379	-0.518322	0.386356
O	4.663743	3.980378	-0.119050
O	2.901474	5.856485	-0.398020
C	1.998022	6.940934	-0.564816
H	1.349052	7.059739	0.312016
H	2.621751	7.828673	-0.678367
H	1.378924	6.812340	-1.461743
C	5.673370	2.981380	0.005359
H	6.622770	3.514892	-0.050797
H	5.603921	2.457341	0.965923
H	5.618657	2.249290	-0.807964
Pd	0.807213	-2.582117	0.260438
H	-0.853673	-1.407785	-2.046275
C	5.080662	-1.954887	0.966253
C	3.728019	-2.057658	0.238520
O	2.851365	-2.757047	0.862879
O	3.589673	-1.477207	-0.853236
H	5.574176	-2.936813	0.947553
H	4.914389	-1.706862	2.019069
N	5.879096	-0.937613	0.312348
H	5.532961	-0.656760	-0.598650
C	7.143162	-0.642137	0.698369
O	7.722210	-1.092023	1.672448
O	7.694660	0.280230	-0.156965
C	9.047459	0.628899	0.149714
H	9.344843	1.349400	-0.614650
H	9.122553	1.076384	1.145226
H	9.697101	-0.250540	0.112927
C	0.894565	-4.471156	-1.053635
H	0.096438	-4.269736	-1.770589
C	0.622756	-4.729085	0.285188
H	-0.402504	-4.937713	0.572654
H	1.418621	-5.114977	0.916090
O	2.106790	-4.686963	-1.568014
C	2.430415	-3.980268	-2.777654

H	3.261034	-4.521934	-3.231970
H	1.572027	-3.976503	-3.461936
H	2.733358	-2.960946	-2.520781

TS1

B3LYP SCF energy: -2488.95514615 a.u.
B3LYP enthalpy: -2488.163200 a.u.
B3LYP free energy: -2488.306470 a.u.
M06 SCF energy in solution: -2488.17971477 a.u.
M06 enthalpy in solution: -2487.387769 a.u.
M06 free energy in solution: -2487.531039 a.u.
Three lowest frequencies (cm-1): -337.2936 7.8672 13.4165
Imaginary frequency: -337.2936 cm-1

Cartesian coordinates

ATOM	X	Y	Z
C	-1.239486	-2.694029	-0.403254
C	-2.162328	-2.725182	0.662002
C	-3.374729	-2.044196	0.592940
C	-3.726587	-1.298068	-0.547659
C	-2.800706	-1.250839	-1.604218
C	-1.596285	-1.954945	-1.546167
H	-1.937405	-3.311723	1.551329
H	-4.074632	-2.120356	1.421878
H	-3.045153	-0.683896	-2.499956
C	-5.091772	-0.662677	-0.685093
H	-5.860231	-1.429468	-0.503516
H	-5.241018	-0.342264	-1.724748
Si	-5.639542	0.820793	0.397180
C	-7.371396	1.374190	-0.183673
H	-7.768280	1.996979	0.633380
C	-5.583893	0.415897	2.262996
H	-4.562234	0.054895	2.455353
C	-7.316156	2.255432	-1.447768
H	-6.928548	1.696531	-2.309837
H	-8.321826	2.605219	-1.716904
H	-6.676269	3.130398	-1.305191
C	-8.352562	0.201559	-0.394484
H	-8.459150	-0.430471	0.493649
H	-9.351997	0.578265	-0.648789
H	-8.033524	-0.443244	-1.223050
C	-6.549124	-0.709329	2.690049
H	-6.422486	-1.624994	2.099422
H	-6.388647	-0.975385	3.743090
H	-7.595827	-0.396925	2.595433
C	-5.807757	1.673189	3.128419
H	-5.700019	1.435017	4.194903
H	-5.096784	2.471143	2.889884
H	-6.817693	2.079182	2.988555
O	-4.686825	2.182975	0.083738
C	-3.337672	2.398447	0.082705
C	-2.509947	1.991570	1.135347
C	-2.783292	3.094604	-0.998804

C	-1.139104	2.223490	1.071196
H	-2.932641	1.493439	2.000507
C	-1.411851	3.326341	-1.050995
H	-3.437455	3.424601	-1.799927
C	-0.560167	2.870672	-0.030893
H	-0.510318	1.895634	1.893421
H	-0.987431	3.828707	-1.916225
C	0.911687	3.012191	-0.148950
C	1.504359	4.251099	-0.439607
C	1.762738	1.897779	-0.019359
C	2.884644	4.386905	-0.586574
H	0.862242	5.119263	-0.528169
C	3.161841	2.013685	-0.179720
C	3.730456	3.247990	-0.457397
H	3.757412	1.109726	-0.126039
C	1.237609	0.591957	0.207137
N	0.866381	-0.494133	0.380158
O	5.053890	3.476164	-0.631047
O	3.521430	5.551962	-0.850768
C	2.744504	6.731259	-0.999181
H	2.189096	6.964724	-0.081656
H	3.457919	7.530816	-1.204563
H	2.041071	6.646247	-1.837578
C	5.938724	2.363507	-0.528715
H	6.939218	2.764974	-0.691680
H	5.889206	1.898662	0.462259
H	5.718511	1.608417	-1.292607
Pd	0.822460	-2.605630	0.092049
H	-0.930438	-1.942460	-2.405992
C	5.105142	-2.260687	0.983646
C	3.757585	-2.044000	0.266225
O	2.814456	-2.773114	0.746868
O	3.693039	-1.230504	-0.668849
H	5.371220	-3.323751	0.924699
H	4.998292	-2.022323	2.047954
N	6.106335	-1.418975	0.365609
H	5.798543	-0.938760	-0.471767
C	7.279932	-1.106717	0.958524
O	7.704112	-1.563950	2.006975
O	7.956618	-0.176735	0.205420
C	9.241931	0.173333	0.724065
H	9.658075	0.893070	0.016345
H	9.157064	0.623055	1.717895
H	9.889419	-0.705727	0.793460
C	-0.557309	-4.590012	-0.812276
H	-1.290619	-5.035057	-0.139799
C	0.821348	-4.574445	-0.401714
H	1.045367	-5.109453	0.522672
H	1.552393	-4.722468	-1.197065
O	-0.805937	-4.791287	-2.126796
C	-2.166860	-5.024187	-2.491929
H	-2.150706	-5.332848	-3.538135
H	-2.599830	-5.826126	-1.880229
H	-2.759841	-4.110414	-2.383088

7
B3LYP SCF energy: -2489.00052534 a.u.
B3LYP enthalpy: -2488.205727 a.u.
B3LYP free energy: -2488.351450 a.u.
M06 SCF energy in solution: -2488.21186039 a.u.
M06 enthalpy in solution: -2487.417062 a.u.
M06 free energy in solution: -2487.562785 a.u.
Three lowest frequencies (cm-1): 6.1938 11.2742
14.2923

Cartesian coordinates

ATOM	X	Y	Z
C	-1.642285	-0.197652	2.250677
C	-2.462200	-0.825392	1.306180
C	-2.255805	-2.159148	0.949622
C	-1.226109	-2.914647	1.530353
C	-0.401443	-2.276666	2.470517
C	-0.607114	-0.945094	2.827209
H	-3.277463	-0.266232	0.852810
H	-2.926369	-2.627535	0.231897
H	0.399376	-2.839984	2.944948
C	-1.030697	-4.375202	1.188528
H	-2.007507	-4.866307	1.075733
H	-0.534859	-4.887539	2.024028
Si	-0.024784	-4.799646	-0.374679
C	0.349009	-6.671413	-0.397652
H	0.655340	-6.904843	-1.429271
C	-0.889291	-4.199092	-1.967442
H	-1.038227	-3.117131	-1.830559
C	1.521273	-7.053155	0.528302
H	1.283755	-6.849469	1.580787
H	1.739906	-8.126715	0.449650
H	2.431848	-6.500282	0.281429
C	-0.887490	-7.535554	-0.070204
H	-1.733882	-7.339240	-0.737198
H	-0.644357	-8.602841	-0.157050
H	-1.231105	-7.367368	0.958022
C	-2.288302	-4.814284	-2.183103
H	-2.944949	-4.685468	-1.314841
H	-2.785365	-4.342807	-3.040899
H	-2.227984	-5.887648	-2.399129
C	-0.016859	-4.408452	-3.222296
H	-0.503006	-3.982033	-4.109753
H	0.970492	-3.943547	-3.128556
H	0.140633	-5.475838	-3.422143
O	1.523439	-4.117256	-0.248733
C	2.103164	-2.890617	-0.280944
C	1.660078	-1.847893	-1.105911
C	3.242241	-2.690996	0.514168
C	2.339511	-0.633366	-1.124597
H	0.794594	-1.984433	-1.742218
C	3.911393	-1.473000	0.491482
H	3.581574	-3.502325	1.150506
C	3.471563	-0.413901	-0.323265
H	1.991592	0.144381	-1.797318

H	4.774460	-1.329928	1.136354
C	4.199237	0.876816	-0.343797
C	5.604227	0.890388	-0.407393
C	3.545626	2.126812	-0.298963
C	6.336573	2.074773	-0.436001
H	6.119393	-0.060351	-0.466331
C	4.276888	3.338910	-0.337425
C	5.659621	3.328291	-0.407470
H	3.733429	4.274720	-0.298562
C	2.132981	2.218159	-0.153184
N	0.983664	2.328916	-0.029321
O	6.454526	4.425464	-0.450900
O	7.683048	2.150513	-0.506845
C	8.433222	0.942342	-0.538171
H	8.189541	0.343482	-1.424562
H	9.479501	1.247461	-0.581784
H	8.263563	0.343767	0.365511
C	5.831036	5.702205	-0.434676
H	6.643034	6.428822	-0.486046
H	5.167114	5.834776	-1.298815
H	5.260334	5.858139	0.490102
Pd	-0.996564	2.417843	0.101851
H	0.019450	-0.483944	3.585764
C	-4.421683	2.858812	-2.015876
C	-3.089753	2.688280	-1.307801
O	-2.020788	2.697513	-1.964424
O	-3.081748	2.545535	-0.026250
H	-4.522584	2.074215	-2.774700
H	-4.390307	3.811630	-2.562331
N	-5.524865	2.807046	-1.082646
H	-5.304754	2.800051	-0.096097
C	-6.788325	2.531787	-1.498354
O	-7.137301	2.400169	-2.659745
O	-7.624945	2.428469	-0.426336
C	-8.988476	2.158132	-0.763135
H	-9.402770	2.955595	-1.386937
H	-9.078720	1.209371	-1.300194
H	-9.519376	2.107057	0.188955
C	-1.881860	1.246905	2.672311
H	-2.885932	1.549691	2.339999
C	-0.855308	2.230963	2.116094
H	-1.061625	3.240491	2.492760
H	0.159208	1.934371	2.396013
O	-1.804622	1.393529	4.099018
C	-2.907851	0.837609	4.788155
H	-2.755314	1.045127	5.851073
H	-3.856659	1.297789	4.468132
H	-2.981314	-0.250045	4.643070

TS2

B3LYP SCF energy: -2488.96741127 a.u.
B3LYP enthalpy: -2488.177847 a.u.
B3LYP free energy: -2488.321658 a.u.
M06 SCF energy in solution: -2488.19203068 a.u.

M06 enthalpy in solution: -2487.402466 a.u.
M06 free energy in solution: -2487.546277 a.u.
Three lowest frequencies (cm-1): -561.4742 5.6122
9.7266
Imaginary frequency: -561.4742 cm-1

Cartesian coordinates

ATOM	X	Y	Z
C	-2.138214	-2.228198	-1.961752
C	-2.348597	-2.525186	-0.608306
C	-3.390672	-1.913396	0.090045
C	-4.241375	-0.984719	-0.528773
C	-4.049257	-0.731148	-1.895188
C	-3.020770	-1.346365	-2.602860
H	-1.671381	-3.200778	-0.090824
H	-3.530103	-2.151359	1.142673
H	-4.702460	-0.028037	-2.406286
C	-5.290310	-0.230789	0.247386
H	-5.732796	-0.868450	1.024112
H	-6.113522	0.055106	-0.420012
Si	-4.668137	1.373923	1.093716
C	-6.116229	2.609070	1.214839
H	-5.766367	3.421545	1.870097
C	-3.818880	1.013051	2.766411
H	-2.966808	0.359262	2.529616
C	-6.456068	3.233796	-0.153446
H	-6.824111	2.478291	-0.860211
H	-7.248630	3.986680	-0.047470
H	-5.586181	3.716046	-0.608579
C	-7.381479	1.998191	1.852154
H	-7.202772	1.612162	2.861173
H	-8.177385	2.751003	1.926379
H	-7.777165	1.172470	1.247231
C	-4.704596	0.245335	3.769242
H	-5.112816	-0.681460	3.349445
H	-4.122654	-0.029384	4.658575
H	-5.548286	0.854063	4.115143
C	-3.251240	2.292529	3.415112
H	-2.705877	2.048996	4.336397
H	-2.557615	2.820064	2.751762
H	-4.051443	2.992068	3.688576
O	-3.573212	2.153774	0.058717
C	-2.225691	2.271327	-0.076750
C	-1.314520	1.273894	0.296458
C	-1.735230	3.457114	-0.645310
C	0.055182	1.477318	0.135836
H	-1.668524	0.332546	0.700482
C	-0.368542	3.641609	-0.820123
H	-2.443819	4.219585	-0.953611
C	0.562244	2.660474	-0.427860
H	0.731379	0.699094	0.474043
H	-0.017099	4.556959	-1.288525
C	2.017992	2.906658	-0.585665
C	2.536882	4.181221	-0.287804
C	2.940231	1.931799	-1.027060
C	3.888872	4.488661	-0.415333

H	1.852615	4.933864	0.083033
C	4.319034	2.227381	-1.149954
C	4.804386	3.489397	-0.852002
H	4.986845	1.446189	-1.490927
C	2.521682	0.622000	-1.396519
N	2.192709	-0.453069	-1.690422
O	6.101287	3.874898	-0.935808
O	4.438848	5.691202	-0.137578
C	3.590580	6.742693	0.306130
H	3.102569	6.489552	1.255640
H	4.242824	7.604841	0.451385
H	2.827051	6.983846	-0.444137
C	7.059275	2.908900	-1.346099
H	8.021901	3.421868	-1.331022
H	7.089106	2.055836	-0.655932
H	6.854932	2.547806	-2.362556
Pd	1.016496	-2.182149	-1.684244
H	-2.885281	-1.121239	-3.657315
C	2.012537	-4.197252	2.079962
C	1.310220	-3.776327	0.780511
O	0.266262	-4.367937	0.475119
O	1.896702	-2.828012	0.125664
H	2.240377	-5.268440	2.005488
H	1.303603	-4.090664	2.909241
N	3.207938	-3.417992	2.325501
H	3.467461	-2.755169	1.607170
C	3.832517	-3.416067	3.525467
O	3.534442	-4.103528	4.489512
O	4.872782	-2.524257	3.517487
C	5.605520	-2.466426	4.741656
H	4.962644	-2.159255	5.572329
H	6.045981	-3.438514	4.983529
H	6.391278	-1.725981	4.578542
C	-0.974204	-2.768484	-2.765725
H	-0.067317	-3.347157	-1.582138
C	-0.098545	-1.829995	-3.408091
H	0.418378	-2.177686	-4.301841
H	-0.386417	-0.782084	-3.403916
O	-1.126787	-3.962691	-3.422789
C	-1.622634	-5.071741	-2.665199
H	-1.635515	-5.916577	-3.355218
H	-0.954091	-5.281979	-1.821059
H	-2.635126	-4.879727	-2.293767

8
B3LYP SCF energy: -2488.97625570 a.u.
B3LYP enthalpy: -2488.184447 a.u.
B3LYP free energy: -2488.330048 a.u.
M06 SCF energy in solution: -2488.19583874 a.u.
M06 enthalpy in solution: -2487.404030 a.u.
M06 free energy in solution: -2487.549631 a.u.
Three lowest frequencies (cm-1): 5.9600 8.7709
18.7008

Cartesian coordinates

ATOM	X	Y	Z
C	-1.215802	-2.269513	-2.055332
C	-1.392550	-2.627232	-0.707924
C	-2.599677	-2.353061	-0.064929
C	-3.660304	-1.713737	-0.727699
C	-3.451917	-1.316568	-2.056679
C	-2.253721	-1.591743	-2.712835
H	-0.580070	-3.101746	-0.162132
H	-2.723825	-2.665392	0.970130
H	-4.249475	-0.810168	-2.595311
C	-4.996327	-1.486000	-0.059107
H	-5.263913	-2.365321	0.543761
H	-5.773400	-1.406421	-0.830662
Si	-5.216179	0.034296	1.082792
C	-7.054575	0.541400	1.101263
H	-7.172366	1.202991	1.973944
C	-4.522440	-0.300277	2.832309
H	-3.507365	-0.700540	2.691021
C	-7.451100	1.350877	-0.149773
H	-7.352948	0.750277	-1.063760
H	-8.500231	1.669029	-0.084840
H	-6.829943	2.242287	-0.271996
C	-8.014566	-0.653567	1.285012
H	-7.811897	-1.229376	2.193884
H	-9.053620	-0.303782	1.344688
H	-7.959552	-1.346715	0.436179
C	-5.315395	-1.372757	3.608364
H	-5.425404	-2.308031	3.046255
H	-4.806440	-1.617254	4.549704
H	-6.319702	-1.019089	3.868950
C	-4.403726	0.990952	3.667458
H	-3.971084	0.775772	4.653370
H	-3.769042	1.739462	3.182132
H	-5.387016	1.447695	3.838421
O	-4.463238	1.393140	0.401535
C	-3.190771	1.850517	0.269854
C	-2.071071	1.194210	0.796311
C	-3.008990	3.055601	-0.425418
C	-0.799644	1.730010	0.621475
H	-2.182015	0.259501	1.331109
C	-1.733000	3.578352	-0.602791
H	-3.880379	3.558306	-0.833210
C	-0.598777	2.921972	-0.091625
H	0.045744	1.211636	1.062404
H	-1.610288	4.495175	-1.173460
C	0.761110	3.471036	-0.299307
C	1.008276	4.842820	-0.112609
C	1.851684	2.665330	-0.686174
C	2.273296	5.399527	-0.285646
H	0.184941	5.469520	0.207119
C	3.144433	3.215444	-0.855069
C	3.368653	4.568074	-0.657699
H	3.953169	2.559087	-1.151143
C	1.688964	1.279083	-0.980552
N	1.600395	0.150676	-1.242289

O	4.563462	5.194508	-0.790306
O	2.575035	6.704906	-0.110914
C	1.536473	7.598818	0.269077
H	1.108681	7.324530	1.241526
H	2.005872	8.580707	0.342267
H	0.739706	7.629364	-0.484787
C	5.694139	4.407221	-1.139721
H	6.536280	5.099555	-1.177588
H	5.890952	3.632771	-0.387229
H	5.565708	3.935335	-2.122625
Pd	1.866036	-1.975915	-1.714142
H	-2.134035	-1.307504	-3.754864
C	3.395918	-3.235684	2.135863
C	2.526100	-3.029378	0.887293
O	1.449854	-3.631460	0.820074
O	3.024431	-2.217137	0.007863
H	3.607536	-4.308420	2.227809
H	2.800429	-2.963362	3.015800
N	4.620152	-2.464418	2.077706
H	4.783338	-1.936207	1.231318
C	5.580689	-2.568930	3.024613
O	5.512523	-3.241988	4.040970
O	6.661732	-1.796306	2.691667
C	7.730614	-1.843355	3.637478
H	7.406419	-1.479405	4.617347
H	8.109978	-2.863200	3.752917
H	8.509575	-1.194378	3.232247
C	-0.021148	-2.681900	-2.865420
H	2.296653	-3.393876	-2.071598
C	0.784807	-1.786966	-3.578295
H	1.375722	-2.177712	-4.402014
H	0.496925	-0.741905	-3.626810
O	0.062878	-3.982798	-3.247283
C	-0.217536	-5.030944	-2.311645
H	-0.065136	-5.956615	-2.868536
H	0.479515	-4.976758	-1.468254
H	-1.249069	-4.982411	-1.949537
